# Proteome of Stored RBC Membrane and Vesicles from Heterozygous Beta Thalassemia Donors

**DOI:** 10.3390/ijms22073369

**Published:** 2021-03-25

**Authors:** Vassilis L. Tzounakas, Alkmini T. Anastasiadi, Monika Dzieciatkowska, Dimitrios G. Karadimas, Konstantinos Stamoulis, Issidora S. Papassideri, Kirk C. Hansen, Angelo D’Alessandro, Anastasios G. Kriebardis, Marianna H. Antonelou

**Affiliations:** 1 Department of Biology, School of Science, National and Kapodistrian University of Athens (NKUA), 15784 Athens, Greece; tzounak@biol.uoa.gr (V.L.T.); alkanast@biol.uoa.gr (A.T.A.); dimitriskrd@biol.uoa.gr (D.G.K.); ipapasid@biol.uoa.gr (I.S.P.); 2 Department of Biochemistry and Molecular Genetics, School of Medicine–Anschutz Medical Campus, University of Colorado, Aurora, CO 80045, USA; monika.dzieciatkowska@ucdenver.edu (M.D.); KIRK.HANSEN@CUANSCHUTZ.EDU (K.C.H.); ANGELO.DALESSANDRO@UCDENVER.EDU (A.D.); 3 Hellenic National Blood Transfusion Centre, Acharnes, 13677 Athens, Greece; kostas.stamoulis@gmail.com; 4 Laboratory of Reliability and Quality Control in Laboratory Hematology (HemQcR), Department of Biomedical Sciences, School of Health & Welfare Sciences, University of West Attica (UniWA), 12243 Egaleo, Greece

**Keywords:** RBC storage lesion, RBC membrane proteome, RBC shape modifications, extracellular vesicles proteome, beta thalassemia trait donors, donor variation effect, network analysis

## Abstract

Genetic characteristics of blood donors may impact the storability of blood products. Despite higher basal stress, red blood cells (RBCs) from eligible donors that are heterozygous for beta-thalassemia traits (βThal^+^) possess a differential nitrogen-related metabolism, and cope better with storage stress compared to the control. Nevertheless, not much is known about how storage impacts the proteome of membrane and extracellular vesicles (EVs) in βThal^+^. For this purpose, RBC units from twelve βThal^+^ donors were studied through proteomics, immunoblotting, electron microscopy, and functional ELISA assays, versus units from sex- and aged-matched controls. βThal^+^ RBCs exhibited less irreversible shape modifications. Their membrane proteome was characterized by different levels of structural, lipid raft, transport, chaperoning, redox, and enzyme components. The most prominent findings include the upregulation of myosin proteoforms, arginase-1, heat shock proteins, and protein kinases, but the downregulation of nitrogen-related transporters. The unique membrane proteome was also mirrored, in part, to that of βThal^+^ EVs. Network analysis revealed interesting connections of membrane vesiculation with storage and stress hemolysis, along with proteome control modulators of the RBC membrane. Our findings, which are in line with the mild but consistent oxidative stress these cells experience in vivo, provide insight into the physiology and aging of stored βThal^+^ RBCs.

## 1. Introduction

The intrinsic characteristics of blood donors might impact the storage capacity of red blood cells (RBCs). Both environmental (alcohol consumption [1], smoking [2]) and genetic (sex [3], ethnicity [4], hemoglobin (Hb) mutation [5]) factors have been shown to affect hemolysis, redox, and metabolic parameters of stored RBCs, as well as their post-transfusion recovery. Beta-thalassemia trait (βThal^+^) is a heterogeneous group of genetic defects in the beta-globin gene, leading to decreased beta-globin synthesis, ineffective erythropoiesis, excess and precipitation of alpha-globin chains, and oxidative stress.

Individuals with beta thalassemia traits consist a non-negligible proportion of blood donors in several geographical areas, including the Mediterranean. It was recently shown that stored RBCs from these subjects cope better with storage stress with respect to hemolysis and redox variables, and have a differentiated metabolism, especially with respect to purine oxidation, arginine metabolism, and the hexosamine pathway [5].

Despite a lack of information regarding the storability of βThal^+^ RBCs, a significant amount of studies have reported differences in the protein composition of the RBC membrane between thalassemic subjects (minor or major) and controls in vivo. The genetic regulation of redox balance in βThal^+^ subjects consists of interesting modifications in the transcript levels of several redox regulators that could be associated with changes in the erythrocyte proteome [6]. It has been shown that an excess of Hb chains can bind to spectrin, generating a spectrin-globin complex that increases the rigidity of the RBC membrane [7]. Moreover, several structural and functional alterations have been observed in the most abundant membrane protein with a critical role in gas transport and RBC structure and metabolism, band 3: extensive phosphorylation [8], cleavage by caspase-3 [9], and increased anion exchange [10]. The latter strongly contributes to the unique surface to volume ratio of RBCs in thalassemia, that in turn enhances their resistance to osmotic stress [11]. Variations in the levels of membrane proteins, including spectrin, flotillin-1, and p55, have emerged from proteomics analysis of HbE/beta-thalassemic erythrocytes [12].

Another interesting aspect of RBC physiology in beta thalassemia is the membrane vesiculation profile. There is evidence that thalassemic subjects exhibit higher levels of extracellular vesicles (EVs) in vivo, a great part of which are released from erythrocytes [13,14,15], and present elevated levels of antigens involved in coagulation [13]. Such EVs have been found to be enriched in antioxidant and chaperone proteins, like HSP70 (beta thalassemia intermedia patients) [13], but containing lower levels of free Hb-scavenging plasma proteins and immunoglobulin chains (HbE/beta-thalassemic patients) [16,17].

Nonetheless, apart from our recent preliminary proteomics study that suggested differences in volume and calcium homeostasis in stored RBCs from βThal^+^ donors compared to controls [5], not much is known about the membrane proteome of βThal^+^ RBCs during storage. Contemporary proteomics approaches that are based on mass spectrometry analysis enable us to effectively analyze protein molecular targets [18] in a sensitive and very high-throughput manner [19]. By applying this technology in combination with classic biochemical and physiological assays, we currently present the first comprehensive report of changes in protein levels of the RBC membrane and EVs from βThal^+^ donors compared to a control throughout storage at blood bank conditions.

## 2. Results

### 2.1. The Proteome of βThal^+^ RBC Membrane during Storage

**Figure 1:**
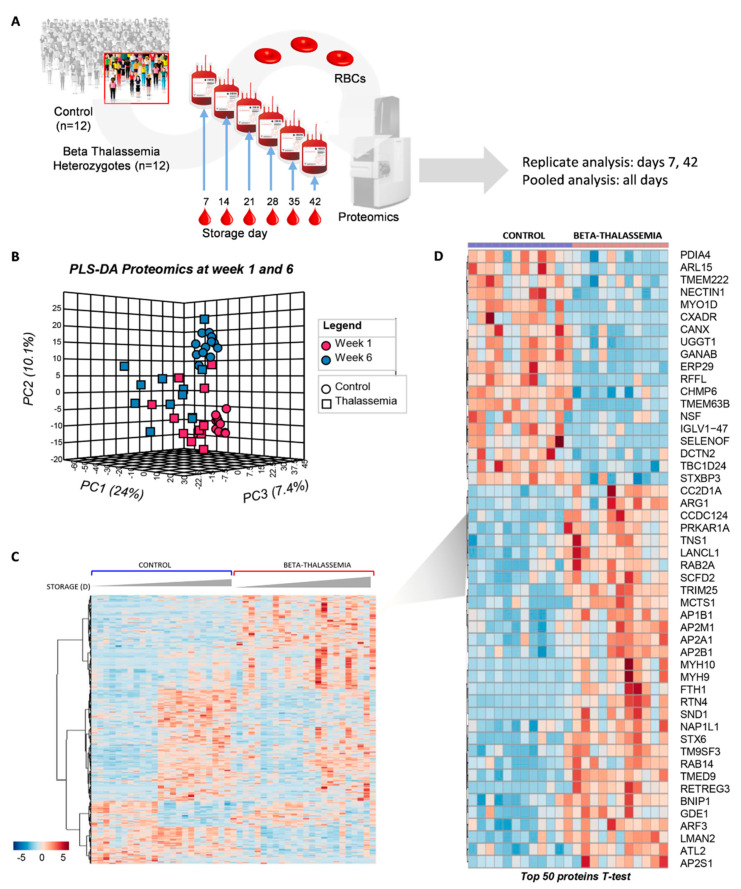
Proteomics analysis of the stored RBC membrane. (**A**) Isolated membranes from *n* = 12 βThal^+^ and *n* = 12 control RBC units were analyzed by proteomics tools in single replicates (day 7 and day 42 samples) or in pooled samples (days 7–42 at weekly intervals). (**B**) Principal component and discriminant analysis of day 7 and day 42 samples. (**C**) Heat map showing differentially expressed proteins between the two groups at early and late storage. (**D**) Heat map showing the top 50 proteins differing between βThal^+^ and control membranes at early storage.

Proteomics analysis was performed both on single biological replicates (young, day 7; old, day 42) or pooled (from day 7 to day 42 on a weekly basis) membrane samples of stored RBCs from the control and βThal^+^ groups (*n* = 12; Figure 1A; Tables S1 and S2). Partial least square-discriminant analysis (PLS-DA) separated the two groups at both early (week 1) and late (week 6) storage across principal component 1 (PC1), explaining ~24% of the total variance (Figure 1B). Several proteins exhibited different expression levels in the membrane of young and old βThal^+^ vs. control RBCs (Figure 1C). The top 50 between-group differences concerning the young RBCs, as determined by an independent *t*-test, are highlighted in the heat map in Figure 1D.

**Figure 2:**
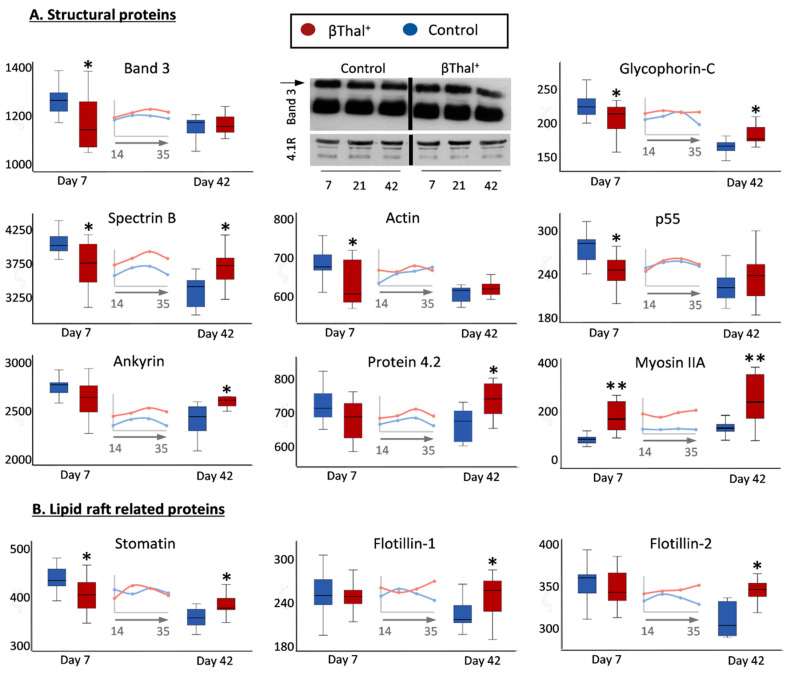
Proteomics analysis of stored RBC membrane: structural proteins. Levels of integral and peripheral membrane proteins (**A**) as well as of lipid raft-related components (**B**) in βThal^+^ and control RBCs throughout storage. Inserts: pooled analysis of membrane samples at weekly intervals. Representative immunoblots of band 3 monomers and dimers (arrow) are shown (*n* = 7). 4.1R protein was used as internal loading control. (*) *p* < 0.05 βThal^+^ vs. controls; (**) *p* < 0.05 and fold > 1.25 βThal^+^ vs. controls.

**Figure 3:**
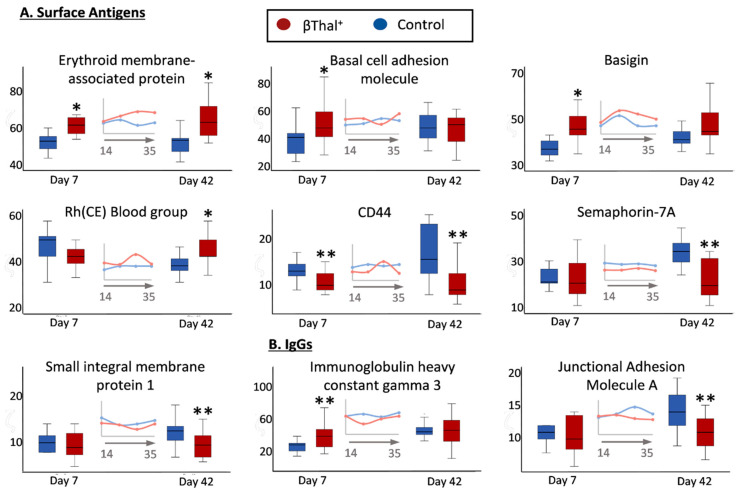
Proteomics analysis of stored RBC membrane: surface antigens and immunoglobulins. Levels of integral protein carriers of surface antigens (**A**) and of immunoglobulins (**B**) in βThal^+^ and control RBCs throughout storage. Inserts: pooled analysis of membrane samples at weekly intervals. (*) *p* < 0.05 βThal^+^ vs. controls; (**) *p* < 0.05 and fold > 1.25 βThal^+^ vs. controls.

**Figure 4:**
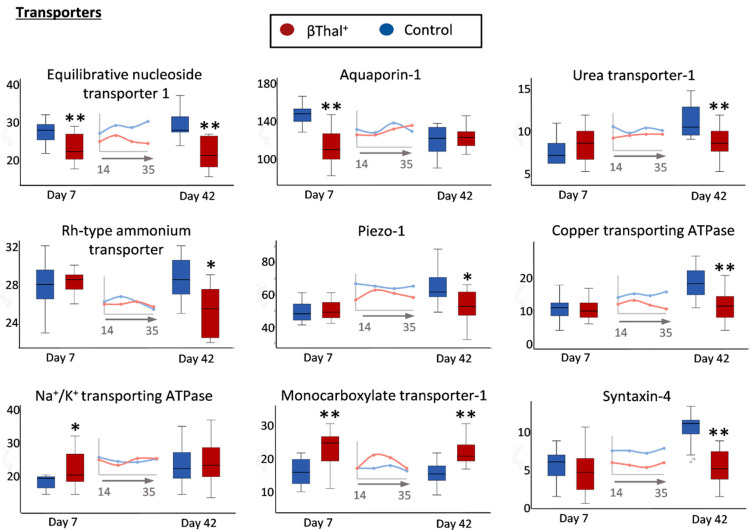
Proteomics analysis of stored RBC membrane: transporters, channels, and pumps. Levels of integral proteins involved in transport across the membrane in βThal^+^ and control RBCs throughout storage. Inserts: pooled analysis of membrane samples at weekly intervals. (*) *p* < 0.05 βThal^+^ vs. controls; (**) *p* < 0.05 and fold > 1.25 βThal^+^ vs. controls.

**Figure 5:**
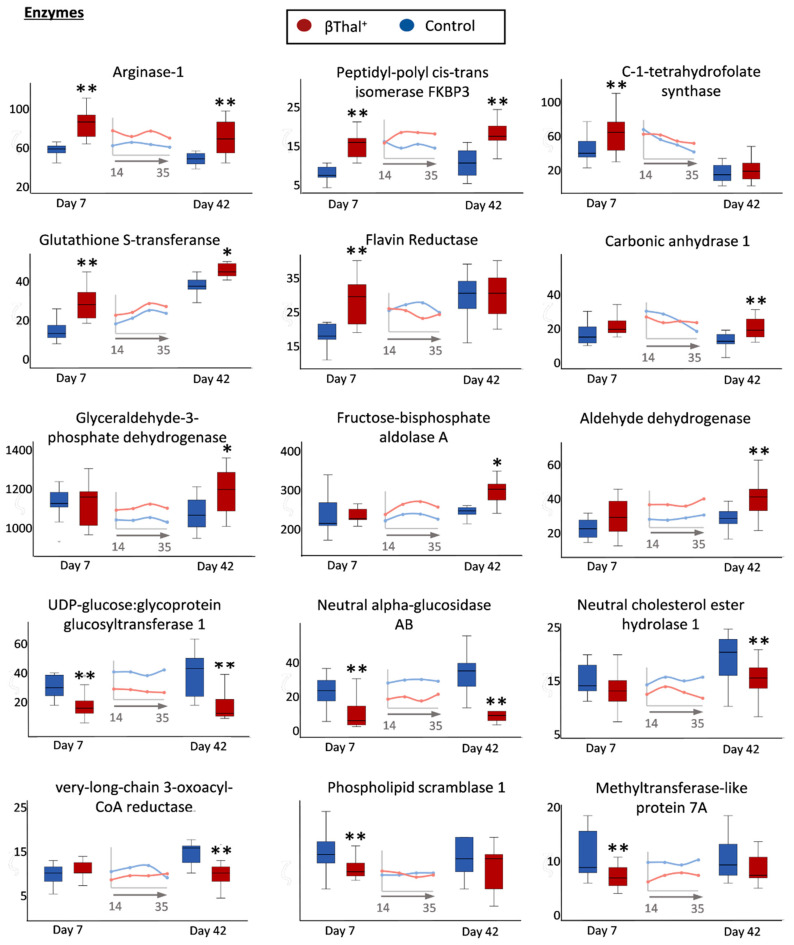
Proteomics analysis of stored RBC membrane: proteins with enzymatic activity. Levels of integral membrane enzymes or cytosolic membrane-bound enzymes in βThal^+^ and control RBCs throughout storage. Inserts: pooled analysis of membrane samples at weekly intervals. (*) *p* < 0.05 βThal^+^ vs. controls; (**) *p* < 0.05 and fold > 1.25 βThal^+^ vs. controls.

**Figure 6:**
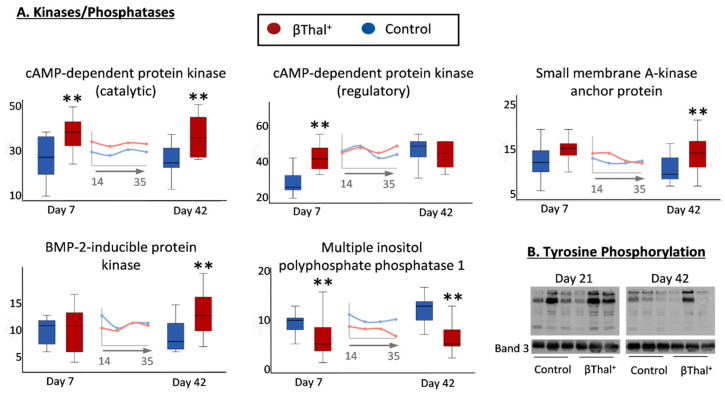
Proteomics analysis of stored RBC membrane: protein phosphorylation. (**A**) Levels of membrane-bound cytosolic protein kinases and phosphatases in βThal^+^ and control RBCs throughout storage. Inserts: pooled analysis of membrane samples at weekly intervals. (*) *p* < 0.05 βThal^+^ vs. controls; (**) *p* < 0.05 and fold > 1.25 βThal^+^ vs. controls. (**B**) Representative immunoblots showing variation in the levels of protein Tyrosine phosphorylation in βThal^+^ and control RBC membrane samples treated with phosphatase inhibitors (*n* = 3 per group) at middle and late storage. Band 3 was used as the internal loading control.

**Figure 7:**
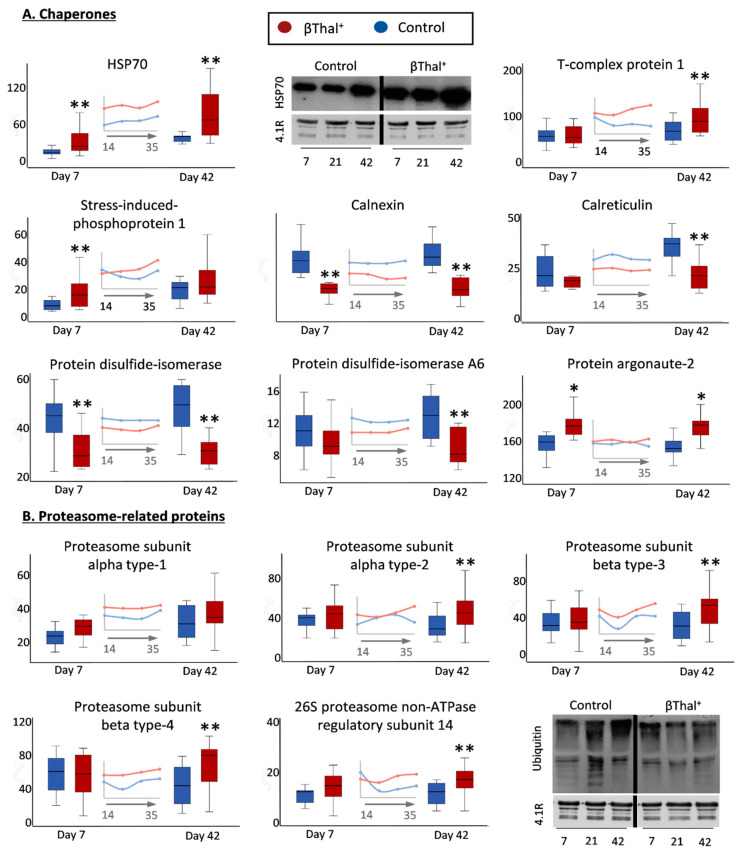
Proteomics analysis of stored RBC membranes: “repair or destroy” group of proteins. Variation in the membrane levels of selected chaperones (**A**) and of 20S/26S proteasome subunits (**B**) in βThal^+^ and control RBCs throughout storage. Inserts: pooled analysis of membrane samples at weekly intervals. (*) *p* < 0.05 βThal^+^ vs. controls; (**) *p* < 0.05 and fold > 1.25 βThal^+^ vs. controls. Representative immunoblots showing variation in the levels of heat shock protein-70 (HSP70) and of ubiquitinylated proteins at early, middle, and late storage are also shown (*n* = 7 per group). Protein 4.1R was used as internal loading control.

The majority of the differently expressed proteins belonged to seven functional clusters: key structural proteins (including lipid raft-associated components), antigens and immunoglobulins, transporters, metabolic enzymes, kinases/phosphatases, and stress response proteins (including chaperones, proteasome subunits, and proteasome-related components) (Figure 2, Figure 3, Figure 4, Figure 5, Figure 6 and Figure 7).

#### 2.1.1. Major Structural Proteins and Lipid Raft-Associated Components

Concerning the major structural proteins, lower content of integral (band 3, glycophorin C), skeletal (spectrin, actin, p55) and raft-associated (stomatin, p55) components were detected in the membrane of βThal^+^ RBCs compared to the control at early storage (Figure 2). In striking contrast, numerous myosin proteoforms (e.g., non-muscle myosin IIA, Figure 2A; myosin IIB, 37 ± 9 vs. 146 ± 100 A.U., control vs. βThal^+^, *p* = 0.001, day 42) were found in great excess. For the following storage time, no significant reduction in prominent membrane components (glycophorin C, spectrin, ankyrin, stomatin, etc.) was observed in βThal^+^ samples, resulting in higher membrane levels at late storage compared to the control RBCs. Normal levels of membrane-bound globin chains (e.g., day 42: 184 ± 48 vs. 179 ± 54 A.U., control vs. βThal^+^) and band 3 oligomers (Figure 2A) were detected in βThal^+^ samples by both proteomics and immunoblotting analyses.

#### 2.1.2. Blood Group Surface Antigens

In the category of membrane proteins carrying blood group antigens, several components were increased (e.g., erythroid membrane-associated protein-ERMAP/Scianna blood group; basal cell adhesion molecule-BCAM/Lutheran antigen; basigin/Ok blood group; Rh-CE glycoprotein) or decreased (CD44, AnWj and In antigens; Semaphorin 7A, John-Milton Hagen blood group antigen; small integral membrane protein-1, SMIM/Vel blood group antigen) in βThal^+^ samples compared to the control (Figure 3A). With a few exceptions (e.g., IGHG3 Figure 3B), control levels of membrane-bound immunoglobulin proteoforms were detected in the stored βThal^+^ RBCs. Notably, the junctional adhesion molecule A (F11 receptor), a member of the immunoglobin superfamily and potential platelet receptor, was detected at lower levels in old stored βThal^+^ RBCs (Figure 3B).

#### 2.1.3. Transport Across the Membrane: Pumps, Channels, and Transporters

Compared to control RBCs, the βThal^+^ RBCs had lower levels of many transmembrane transporters, pumps and channels playing significant roles in metabolite influx/efflux and volume regulation, either throughout storage (equilibrate nucleoside transporter 1, ENT1) or at the early (aquaporin-1) or later period of it (N_2_ metabolism-associated urea and ammonium transporters; Piezo-1; copper transporting ATPase) (Figure 4). A trend for lower levels of the Gardos ion channel (KCNN4, 16.3 ± 1.5 vs. 14.1 ± 3.2, *p* = 0.052) was also detected in young stored βThal^+^ RBCs. On the contrary, there was an increased expression of Na^+^/K^+^ ATPase (in young stored RBCs) and of monocarboxylate transporter-1 (SLC16A1). Syntaxin-4 had low levels in βThal^+^ samples when compared to controls (Figure 4).

#### 2.1.4. Enzymes

The βThal^+^ RBC repertoire of membrane-bound enzymes was also strikingly different compared to the controls (Figure 5). Enzymes participating in the urea cycle, purine synthesis, and amino acid metabolism (arginase-1, peptidyl-poly cis-trans isomerase, C-1 tetrahydrofolate synthase) in addition to NADPH-dependent redox systems (flavin reductase, glutathione transferase) had significantly increased levels at early storage or throughout it. Carbonic anhydrase and glycolysis-associated enzymes (e.g., glyceraldehyde-3-phosphate dehydrogenase) were selectively increased in old stored βThal^+^ RBCs. On the other side, extremely low levels of enzymes involved in protein glycosylation (UDP-glucose:glycoprotein glucosyltransferase), as well as in carbohydrate (neutral alpha-glucosidase) and lipid (neutral cholesterol ester hydrolase 1, very long chain 3-oxoacyl-CoA reductase) catabolism, were detected throughout storage or in old stored βThal^+^ RBCs. Notably, downregulation of phospholipid scramblase 1 (participating in the deregulation of membrane phospholipid asymmetry) and of methyltransferase-like protein 7A (a probable sensor of oxidative stress in RBCs), was also detected in young stored βThal^+^ samples.

It is worth mentioning that, while several cytosolic kinases exhibited higher membrane binding in the βThal^+^ stored RBCs, the phosphatases demonstrated the opposite trend (Figure 6A). Consequently, higher protein tyrosine phosphorylation was detected by immunoblotting analysis in the βThal^+^ RBC membrane compared to the control at middle and late storage (Figure 6B).

#### 2.1.5. “Repair or Destroy” Proteins

Finally, several proteins with significant differences in stored βThal^+^ RBCs compared to the controls fall in the group of “repair or destroy” proteins, including chaperones and proteasome molecules participating in stress responses (Figure 7A). Indeed, significant enrichment of βThal^+^ vs. the control membrane in molecular chaperones, co-chaperones, and protein partners was evident at individual storage times (e.g., stress-induced phosphoprotein 1, STIP1, week 1; T-complex proteins, week 6) or throughout storage (Hsp70; DNAJ 21 ± 7 vs. 37 ± 14 A.U., control vs. βThal^+^, *p* = 0.002, day 42 samples) by proteomics and immunoblotting analyses. In contrast, the calcium-binding chaperones calnexin and calreticulin, as well as several protein disulfide isomerases, were less abundant in the βThal^+^ membrane (Figure 7A). The membrane association of proteasome subunits, both catalytic and regulatory, was greater in the βThal^+^ group at late storage (Figure 7B), but protein ubiquitinylation exhibited the opposite pattern (day 42: 19 ± 5 vs. 14 ± 4, controls vs. βThal^+^, *p* = 0.006; see representative immunoblots in Figure 7B). Apart from these protein groups, the membrane of stored βThal^+^ RBCs was further characterized by the overexpression of protein argonaute-2 (AGO2) implicated in miRNA binding (Figure 7A) and of AP-2 complex members involved in the internalization of transferrin receptor in reticulocytes [20]. Finally, different levels of small GTPases (mostly enrichment) were detected in stored βThal^+^ RBC membrane (Table S1)**.**

### 2.2. RBC Shape and Membrane Vesiculation

**Figure 8:**
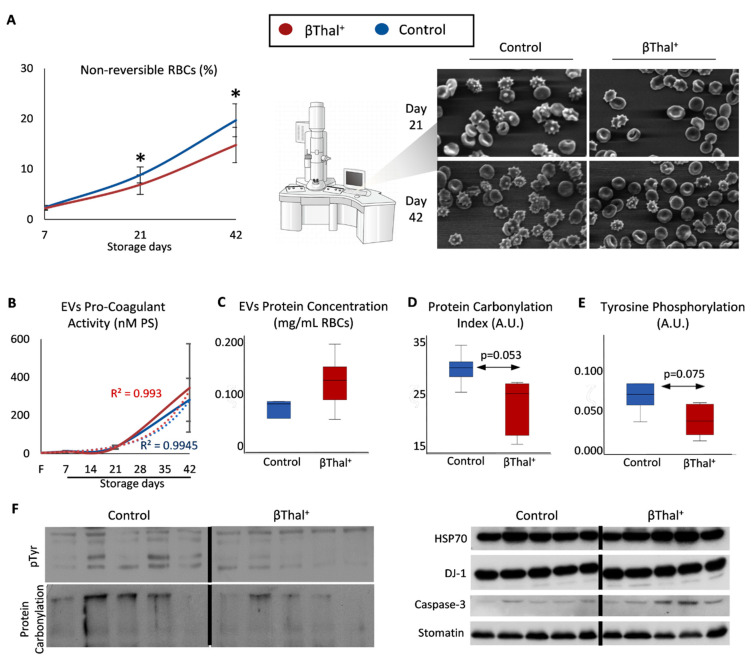
βThal^+^ RBC shape modifications and storage EV characteristics. (**A**) Percentage of non-reversible shape modifications in βThal^+^ and control RBC units throughout storage and representative micrographs captured by scanning electron microscopy (*n* = 8 per group; magnification: 1000×). (**B**–**F**) EV analysis (*n* = 5 per group, except procoagulant activity: *n* = 8 per group): procoagulant activity (**B**), total protein concentration (**C**), protein carbonylation (**D**), and protein tyrosine phosphorylation (**E**) levels in βThal^+^ and control EVs. (**F**) Representative immunoblots of individual proteins or protein modifications showing a trend for different expression levels in βThal^+^ vs. control EVs. Stomatin was used as the internal loading control. (*) *p* < 0.05 βThal^+^ vs. controls; F: fresh blood.

We then examined whether the abovementioned variation of the RBC membrane proteome was associated with typical storage-induced changes in cellular shape and the degree of membrane vesiculation. To this purpose, we first proceeded to a morphological evaluation of stored RBCs by scanning electron microscopy. Lower levels of irreversible shape modifications (e.g., spherocytes) were observed in βThal^+^ vs. the control units at middle and late storage (Figure 8A). Following sequential isolation of supernatants and then of EVs accumulated into them up to late storage, we found equal levels of procoagulant activity (Figure 8B), but a slightly higher (*p* = 0.198) concentration of total vesicular proteins per volume unit of packed RBCs (Figure 8C). Of note, a trend (*p* = 0.053–0.150) for lower levels of post-translational modifications, such as protein carbonylation and phosphorylation (Figure 8D–F, left panel), but overexpression of molecular chaperones (e.g., HSP70, DJ-1) and caspase-3 (Figure 8F, right panel), was detected by immunoblotting in EVs collected from βThal^+^ units compared to the control.

**Figure 9:**
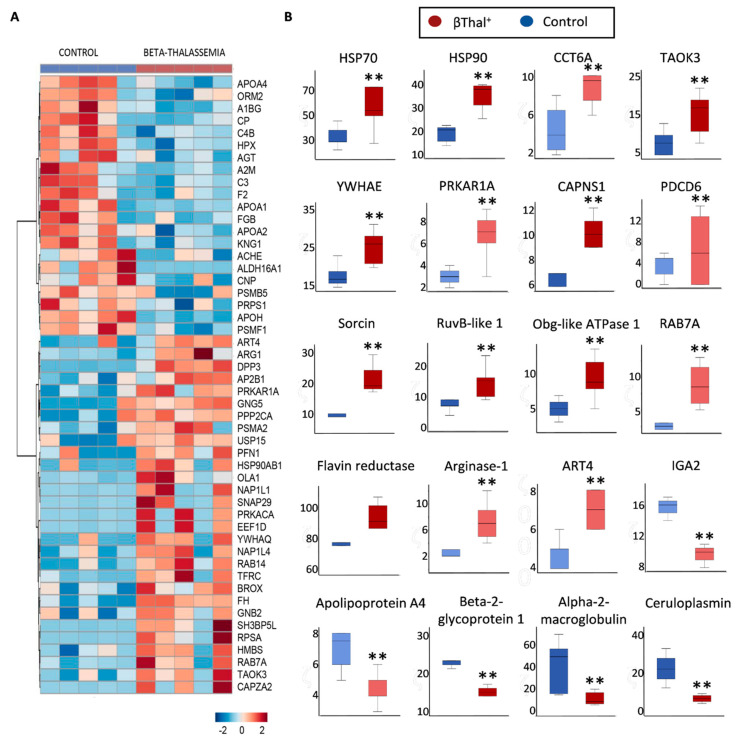
Proteomics analysis of storage EVs. (**A**) Heat map for proteins differing between βThal^+^ and control vesicles (*n* = 5 per group). (**B**) Representative boxplots showing statistically significant variation in the levels of specific EV proteins between the two groups. Light and dark colors indicate proteins of low and high abundance, respectively. (**) *p* < 0.05 and fold > 1.25 βThal^+^ vs. controls. Abbreviations: CCT6A: T-complex protein 1 subunit zeta; TAOK3: serine/threonine-protein kinase; YWHAE: 14-3-3 protein epsilon; PRKAR1A: cAMP-dependent protein kinase type I-alpha regulatory subunit; CAPNS1: calpain small subunit 1; PDCD6: programmed cell death protein 6; ART4: ecto-ADP-ribosyltransferase 4; IGA2: immunoglobulin alpha-2 heavy chain.

Individual proteomics analysis of EVs (*n* = 5 per group) (Table S3) revealed enrichment in plasma proteins (e.g., complement, IgGs) and in several RBC cytosolic proteins, including Hb subunits, carbonic anhydrases, NADPH-dependent redox systems (catalase), and flavin reductase, compared to the RBC membrane of origin in both groups (Table S4). Moreover, there were significant between-group differences, the top of which are shown in the heat map of Figure 9A. The βThal^+^ EVs exhibited greater levels of molecular chaperones (HSPs, T-complex proteins), kinases (e.g., TAO kinase 3), calcium-related proteins (calpain, sorcin etc.), ATPases (e.g., Obg like ATPase 1), and enzymes like arginase-1 and flavin reductase (Figure 9B). Nevertheless, apolipoproteins, IgGs, and ceruloplasmin were less evident in βThal^+^ EVs when compared to the controls (Figure 9B). Some interesting trends for higher (e.g., carbonic anhydrase, transferrin receptor, proteasome subunits) or lower (e.g., complement C3, hemopexin, coagulation factor V) levels of EV proteins in βThal^+^ vs. the controls are shown in Table S5. Notably, (a) traces of non-muscle myosin IIA were detected only in βThal^+^-derived EVs and (b) AHSP and ALIX (an exosome marker) were present in both groups’ EVs (e.g., ALIX: 22 ± 13 vs. 16 ± 11 A.U., βThal^+^ vs. control), while absent in RBC membranes.

### 2.3. RBCs vs. EVs Networks

To find out RBC parameters (if any) that exhibited statistically significant correlations with EV phenotypes, we proceeded to compare βThal^+^ vs. control RBC EVs via network analysis for late storage. Though EVs collected from the supernatant of day 42 units were the cumulative pool of vesicles released throughout storage, the exponential pattern of EV release with the storage time suggests that the old stored RBCs contributed the most to the final pool.

**Figure 10:**
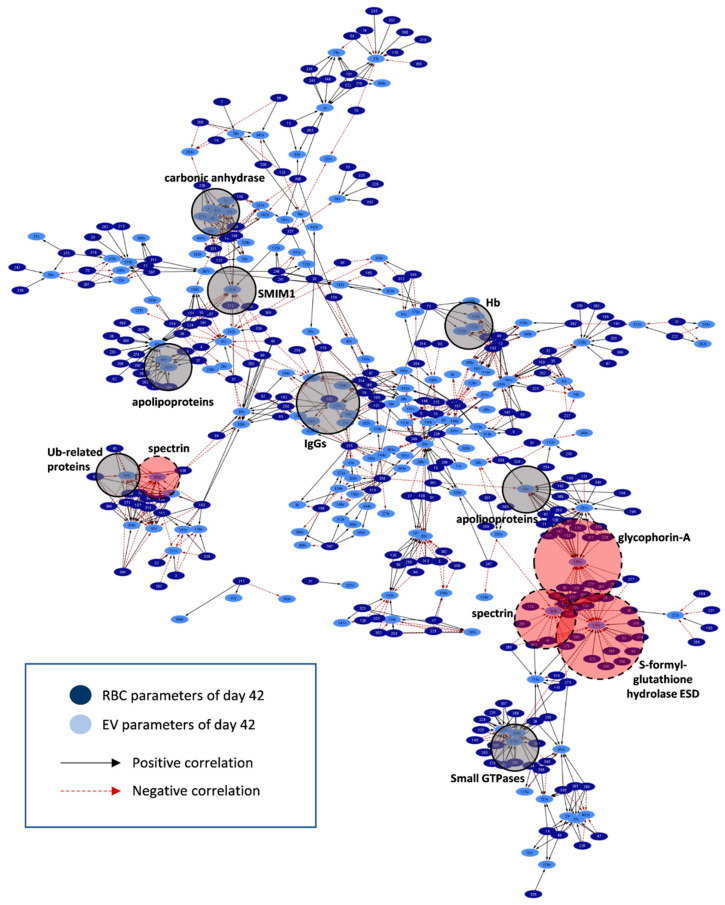
Network connecting RBC to EV parameters in control RBC units on day 42 of storage. Interactome based on the correlation coefficient R values between parameters of stored RBCs and EVs in late storage. The length of each edge is inversely proportional to the R value (the shorter the edge, the higher the R value). All connections are statistically significant at *p* < 0.01. Solid light grey circles focus on high connectivity hubs found in both donor groups. Dashed red circles highlight representative high connectivity hubs that predominate in the control network. For abbreviations, see Table S6.

**Figure 11:**
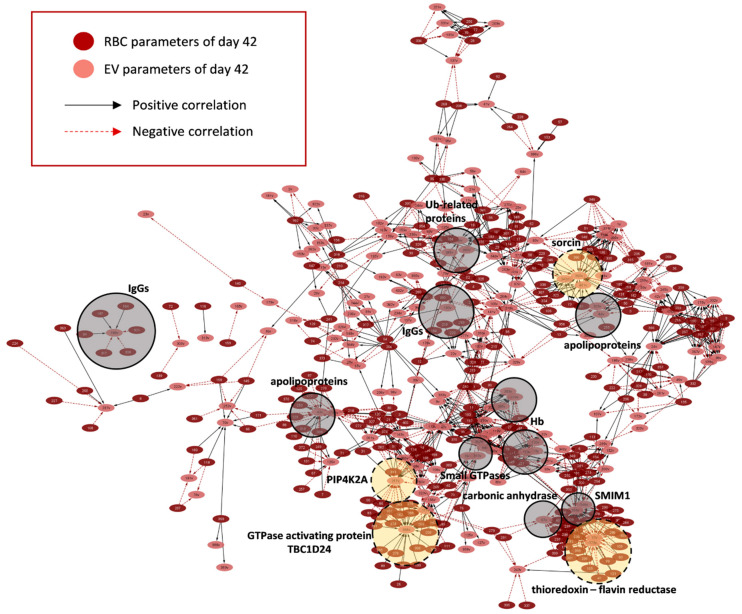
Network connecting RBC to EV parameters in βThal^+^ RBC units on day 42 of storage. Interactome based on the correlation coefficient R values between parameters of stored RBCs and EVs in late storage. The length of each edge is inversely proportional to the R value (the shorter the edge, the higher the R value). All connections are statistically significant at *p* < 0.01. Solid light grey circles focus on high connectivity hubs found in both donor groups. Dashed yellow circles highlight representative high connectivity hubs that predominate in the βThal^+^ network. For abbreviations, see Table S6.

More than 1000 statistically significant (*p* < 0.01) correlations were detected in the control (~1000) and βThal^+^ (~1350) RBC-EV networks (Figure 10 and Figure 11, respectively; Table S6). Variation in the RBC membrane-bound small GTPases (90 vs. 140 connections, βThal^+^ vs. control), proteasome subunits, activators (but not ubiquitin-related proteins; 163 vs. 100 connections, βThal^+^ vs. control), and IgGs (70 vs. 28 connections, βThal^+^ vs. control, respectively) were more interconnected to the target EV phenotypes that are common (e.g., RBC IgGs connections to EV Hb, acetylcholinesterase, and superoxide dismutase) or different between groups (e.g., RBC proteasome connections to Hb concentration exclusively in βThal^+^ EVs) (Table S7). Even though the chaperone proteoforms prevailed in the RBC membrane over proteasome subunits, the latter were significantly more interconnected to EV features, including EV Hb, complement, redox metabolism, and protein tyrosine phosphorylation. Binding of small GTPases to the RBC membrane was further related to the vesicular content of heat shock proteins, IgGs and Alix in both groups, but also with the caspase 3 and total EV protein concentration in βThal^+^ units. Finally, the IgGs load of the membrane in the stored RBCs was strongly connected to the peroxiredoxin protein kinases and protein tyrosine phosphorylation levels in βThal^+^ units.

Without exception, (a) all of the skeletal proteins, (b) components participating in the vertical linkage of cytoskeleton to the lipid bilayer, (c) lipid-raft associated proteins, and (d) Ca^2+^-regulated proteins of the stored RBCs correlated significantly to EV phenotypes, in accordance with the established mechanisms of EV biogenesis. On the other hand, the release of vesicles by the βThal^+^ RBCs seemed to be further connected to variation in the parameters not participating at all (e.g., storage, osmotic and oxidative hemolysis, lipid peroxidation, Hb concentration) or showing significantly fewer connections (e.g., MCV, thioredoxins, Ca^2+^-transporting ATPase, antioxidant capacity of the supernatant) in the control network. Notably, Hb concentration in EVs was negatively related to hemolysis parameters of stored RBCs. The kinases contributed significantly more connections compared to the phosphatases in the networks, however, significantly more phosphatases were detected in the βThal^+^ RBC/EV one (Table S7).

Regarding the “targets” of the RBC features in EVs, apolipoprotein proteoforms absorbed by the residual plasma of the unit constituted the highest connectivity EV hub (where the hub is a parameter with > 15 connections to RBC parameters) in both networks (Table S8). Six additional smaller EV hubs (including EV Hb, carbonic anhydrase, IgGs, and small GTPases) were found in common in the two networks, though at variable connectivity degrees (e.g., twice the number of Hb connections in the βThal^+^ vs. control EVs). As expected, certain EV hubs characterized each network individually. Redox and stress-associated EV components (e.g., flavin reductase, thioredoxin, peroxiredoxins, α-hemoglobin stabilizing protein, complement regulators) characterized the βThal^+^ network as opposed to major membrane components (spectrins, glycophorin A), glutathione hydrolase, heat shock proteins, and complement proteoforms found predominantly among the hubs of the control network. The exosomal ALIX protein correlated with the RBC membrane content of small GTPases Rab and redox homeostasis parameters, such as supernatant antioxidant capacity, lipid peroxidation (in βThal^+^), and glutathione transferase (in control).

The levels of Hb release through vesicles were found to be significantly related to calcium-related factors in both RBC groups, but also to proteasome components and hemolysis levels (as mentioned above) in βThal^+^ RBCs. Those of complement regulators (βThal^+^ hub) in EVs were correlated to RBC PS percentage, storage hemolysis, proteasome and IgG proteoforms. The vesicular α-hemoglobin stabilizing protein (AHSP), flavin reductase, and clusterin levels were found to be highly interconnected with the major RBC membrane proteins, Hb, proteasome subunits, lipid peroxidation, and antioxidant capacity of the supernatant only in the βThal^+^ network (Table S8).

## 3. Discussion

Here, we report the first proteomic dataset comparing the RBC membrane and EVs from βThal^+^ donors vs. control throughout storage in leuko-reduced CPD-SAGM units. The data highlight beta-thalassemia signatures on RBCs and how they are modified during ex vivo aging. Several modifications have not been reported so far in beta-thalassemia minor. Their detection was based on an advanced proteomics analysis of single replicates (*n* = 12 per group) and validation by targeted immunoblotting experiments. Of note, proteomic findings are consistent with the physiological features of the same RBC units, such as RBC morphology and membrane vesiculation (current work), as well as hemolysis and metabolism data [5]. The findings are relevant to transfusion medicine and beyond, by providing the basis for a more detailed exploration of RBC physiology and aging processes, in vivo and ex vivo, in beta-thalassemia minor.

The membrane composition of young stored βThal^+^ RBCs was more indicative of the thalassemic background compared to old stored cells, as it is closer to the in vivo status. Indeed, lower content of integral and skeletal proteins has been reported in the RBCs of HbE/βThal patients [12], suggesting changes in the architecture of the skeleton and its adherence to the membrane. Though detected in simple heterozygous state, these shortcomings are not associated with deformability defects, because the mechanical and osmotic hemolysis of βThal^+^ RBCs were found to be better than the average control [5]. Upregulation of myosin levels may account for these phenomenally paradoxical findings. As a probable result of different reticulocyte maturation in beta thalassemia [21], it has been considered a cellular volume regulator in the microcytic βThal^+^ RBCs [22]. βThal^+^ membrane enrichment in several non-muscle myosin proteoforms might contribute to structural integrity, deformability, and resistance to stress [23], including storage-induced stress, since the expression of structural proteins did not reduce with storage time, as opposed to control RBCs. Of note, both regulators of myosin activity, namely kinases/protein phosphorylation and calmodulin, are also overexpressed in the membrane of βThal^+^ RBCs [24]. Stomatin, which was observed for the first time to be downregulated in βThal^+^ RBCs, followed the same pattern of variation during storage (no significant reduction), resulting (along with flotillins and synexin) in the enrichment of old stored βThal^+^ RBCs in lipid raft components that regulate the lateral organization of the membrane. On the other side, no higher membrane binding of Hb (a typical feature of thalassemic RBCs [25]) was found in stored βThal^+^ RBCs. All of these protein diversifications may account for the better maintenance of stored RBC morphology in βThal^+^ compared to the control.

Several deregulated proteins in βThal^+^ RBCs fall in the group of molecular chaperones, proteasome proteins, redox regulators, and free radical scavengers, reflecting proteotoxic stress and activated antioxidant defense. Among these, glutathione transferase, flavin reductase, and aldehyde dehydrogenase were included. The potentially toxic unstable free α-globin chains in βThal^+^ RBCs can be eliminated by functionally interconnected protein quality control pathways [26]. It has been suggested that chaperones may be involved in α-globin refolding or targeting for degradation to proteasome [12,27,28,29], and, consequently, numerous molecular chaperones are upregulated in βThal^+^ trait erythroblasts in mice.

Upregulation of proteome stress markers in the βThal^+^ RBC membrane at early storage is probably related to higher baseline levels, but many of them were further upregulated later on. Thus, overexpression is the result of both thalassemia- and storage-related [30] proteome and oxidative stresses that are, however, successfully treated, as judged by the low levels of oxidative modifications in βThal^+^ RBC membrane proteins and lipids [5]. Moreover, the function of those chaperones is probably unrelated to α-globin chain toxicity, because normal levels of Hb and α-Hb stabilizing chaperone AHSP [12] were found in the membrane and EVs, respectively. Of note, in platelets, heat shock proteins form large complexes with myosin-targeting subunits [31]. Finally, the protein argonaute-2 that seemed to be an abundant protein of the stored RBC membrane, especially in βThal^+^ RBCs, forms functional complexes with several miRNAs that are differentially expressed in relation to the storage lesion [32].

Downregulation of several transporters in the membrane of βThal^+^ RBCs may be associated with the low levels of the scaffolding protein stomatin [33]. Some of them participate in the regulation of erythropoiesis [34] and in the membrane response to hypoxia [35]. Notably, most of them transport substances directly or indirectly involved in N_2_ metabolism. ENT1, for instance, is a key purinergic component that mediates nucleoside (especially adenosine) uptake by RBCs, while the mechanically activated cation channel piezo-1 modifies the kinetics of ATP release under shear conditions in the vasculature [36]. ENT1 levels are regulated post-translationally in response to exposure to hypoxia, and ultimately contribute to RBC metabolic reprogramming in response to oxidant/hypoxic stress, such as at high altitude or in models of intrauterine growth restriction [37,38]. In the same context, enzymes participating in the urea cycle, purine synthesis, and amino acid metabolism had significantly increased levels in βThal^+^ vs. control RBCs at early storage or throughout it (see below). Recent metabolomics analyses revealed alterations in the purine oxidation pathway in circulating and stored βThal^+^ RBCs [5], while malaria parasites that are purine auxotrophic import purines via their ENT1 homologue [39]. On the other side, while upregulation of monocarboxylate transporter and Na^+^/K^+^ ATPase is suggestive of modified fluxes, previously seen in sickle cell trait and disease [40,41], in βThal^+^ RBCs, it is not associated with increased K^+^ and lactate efflux, nor with RBC dehydration [5], probably due to the mild excess/precipitation of free α-globin chains, and thus, to manageable levels of oxidative stress [42].

Among the enzymes significantly upregulated in the membrane of βThal^+^ RBCs throughout storage was arginase-1. As the final enzyme in the urea cycle, it is responsible for the hydrolysis of L-arginine to urea and L-ornithine. Arginase-1 competes with the nitric oxide synthase (NOS) that also uses L-arginine as a substrate to produce and release the cardiovascular protective nitric oxide (NO) in the bloodstream [43]. Comparing the two enzymes, NOS is more redox-sensitive than arginase [44], and may be readily inactivated during storage. Increased arginase/NOS activity was indeed observed in human (and non-human primate) RBCs as a function of storage duration through arginine tracing experiments [45]. Thalassemia is characterized by dysregulated arginine metabolism and increased arginase activity in plasma, probably leading to low NO bioavailability and cardiovascular dysfunction [46,47]. Moreover, increased arginase expression and activity (at the expense of NO synthesis) were detected in RBCs from patients with diabetes [48] and in the plasma of recipients of aged stored autologous RBCs [49]. Most of the RBC arginase-1 is bound to the membrane in vivo through association with flotillin-1 [50], and this binding increases its enzymatic activity. The currently reported proteomics analysis is consistent with the modulation of arginine and glutamine (an arginine precursor) metabolism recently observed in fresh and stored βThal^+^ RBCs, including ornithine to arginine and citrulline to arginine ratios [5]. These markers of arginase activity in stored RBCs are indicative of the capacity of transfused RBCs to respond to acetylcholine-induced vasodilation [49]. The low propensity of stored βThal^+^ RBCs to hemolysis [5] and the low levels of arginase-1 in both storage EVs and RBC units’ supernatant (manuscript in preparation) suggest that upregulation of the enzyme in βThal^+^ RBCs is not associated with increased arginase-1 release. For the βThal^+^ RBCs per se, however, arginine consumption [5] due to increased expression of arginase-1 would repress the NOS-mediated NO synthesis. Interestingly, in pathologies characterized by increased RBC arginase-1 activity, like diabetes, there is also increased oxidative stress and ROS generation. Moreover, the detrimental effects of arginase-1 enriched RBCs on vascular cells (upregulation of arginase-1 and dysfunction) are ROS-dependent [48]. In contrast, stored βThal^+^ RBCs show lower oxidative defects in lipids and proteins, lower ROS generation at late storage, and an enhanced antioxidant and “repair or destroy” arsenal in their membrane.

Increased arginase activity may be the result of mild, sustained oxidative stress in βThal^+^ traits, as observed in normal aging [51], and a compensatory defense mechanism against the NOS-associated oxidative/nitrosative stress. In either case, it may contribute to the resistance of βThal^+^ RBCs to malaria infection, since the host arginine (intracellular and introduced by RBC amino acid transporters) is metabolized by the parasite to sustain its metabolic needs [52,53].

In fact, this feature is only a part of the general context of proteome diversifications potentially related to βThal^+^ RBC resistance to malaria infection. First of all, the proteome of the βThal^+^ membrane is characterized by downregulation of several parasite receptors, including band 3, glycophorin C, CD44, complement receptor-1 (CR1), and semaphorin-7A [54,55]. The prevalence of the O blood group among our donors [5,56] and variation in other determinants of malaria risk, including the globin chains per se, redox regulators, the protein GNAS [57], and several kinases—potentially involved in protein hyperphosphorylation and thereby membrane destabilization observed in malaria protection [58]—probably work in the same direction. For instance, piezo-1 and beta-spectrin hyperphosphorylation by host kinases is an early RBC response to merozoite attachment on RBCs before parasite entry [59]. Increased activation of protein kinases and protein phosphorylation has been previously reported in erythroblasts of patients with β0-thalassaemia/Hb E disease [60]. Upregulation of argonaute-2 may be also related to the innate resistance of βThal^+^ RBCs to malaria infection; the transfer of argonaute-2 from malaria-infected RBCs to recipient cells through EVs downregulates the expression of essential malaria antigens [61].

The modified expression of membrane receptors, antigens, and IgGs in stored βThal^+^ RBCs compared to the control may be clinically relevant. Some of them, like the ERMAP-related Scianna blood group antigens [62], the SMIM-1-related Vel blood group [63], the CD44 receptor (AnWj blood group antigen) [64], and semaphorin-7A, are clinically involved in the immunohematology of hemolytic transfusion reactions, adhesion processes, macrophage responses, and erythrophagocytosis. For example, Semaphorin-7A, a ligand for the platelet receptor glycoprotein Ib, enhances thrombo-inflammation in myocardial ischemia-reperfusion injury [65]. Moreover, SMIM-1, which is implicated in the physiology of erythroid cells [66], carries a high-frequency blood group antigen able to induce aggressive hemolytic activity [63]. Finally, the AnWj blood group antigen on the CD44 protein is the RBC receptor of *Haemophilus influenzae*. Apparently, deficiency in these surface molecules renders βThal^+^ RBCs a safer choice for transfusion therapy.

The proteomics profiles of EVs accumulated in βThal^+^ and control RBC units throughout storage were also highly revealing. As expected [67], both groups’ EVs were enriched in Hb, complement, and carbonic anhydrase and redox proteoforms, including catalase and peroxiredoxins. Of note, the exosome-related ALIX protein was also detected in EVs, suggesting either the presence of exosomes in the residual plasma of the units or the participation of ALIX in the membrane vesiculation of stored RBCs. Both contingencies deserve further investigation. In terms of between-group variance, the composition of the βThal^+^ EVs reflected, in part, that of the RBCs of origin, since there were several upregulated (chaperones/proteasome, flavin reductase, arginase-1, etc.) or downregulated (e.g., CD44) components. Moreover, porphobilinogen deaminase, sorcin, transferrin receptor, and myosin proteoforms are selectively sorted to βThal^+^ EVs. Myosin and biliverdin reductase were identified as thalassemia-specific components in the plasma microparticles of β-thal/HbE patients [17]. According to our results, biliverdin reductase is a minor component of storage EVs, independently of the thalassemia background.

The release of slightly more vesicular proteins by βThal^+^ RBCs showing better preservation of cell morphology under storage seems paradoxical, but it is plasmatic, because the volume unit of the microcytic βThal^+^ RBCs contains more cells than the control. As opposed to the high levels of PS^+^ microparticles in the plasma of β-thal/HbE patients [17], the βThal^+^ storage EVs exhibited normal levels of procoagulant activity. Moreover, they contained lower levels of protein stress markers (carbonylation, Tyr-phosphorylation) and removal signals (IgGs, complement), suggesting prolonged circulation time compared to control EVs. Finally, they contain lower levels of plasma components exerting antioxidant (e.g., apolipoprotein A-IV and hemopexin implicated in free heme detoxification) and anti-coagulation (beta-2 glycoprotein, a2-macroglobulin) activities, in addition to coagulation factor V and the oxireductase ceruloplasmin that is involved in iron metabolism and transfer across the membrane.

Network analysis revealed interesting connections of membrane vesiculation with hemolysis and the membrane proteome of βThal^+^ RBCs. The degree of protein (mainly Hb) release by βThal^+^ RBCs was positively related not only to the membrane levels of IgGs and lipid raft/Ca^2+^-associated proteins, as expected, but also to variation in the erythroid cell receptor ERMAP that is upregulated in βThal^+^ RBCs. On the contrary, protein loss through membrane vesiculation exhibited negative correlations with the membrane levels of proteasome, regulators of redox activity, small GTPases, and especially with the oxidative and storage hemolysis of parental βThal^+^ RBCs, highlighting the homeostatic role of vesiculation in membrane modulation and damage control. The removal of potentially dangerous compounds and activators of hemolysis by RBCs renders them less susceptible to storage and oxidative hemolysis. The currently reported networks contain hundreds of potentially informative correlations between EV and RBC features that deserve investigation by targeted studies. Some of them are expected to shed light on the emerging role of membrane proteasomes in the physiology of RBCs.

## 4. Materials and Methods

### 4.1. Biological Samples

Twenty-four (12 βThal^+^ and 12 control) blood units of packed RBCs in citrate-phosphate-dextrose (CPD)/saline-adenine-glucose-mannitol (SAGM) were stored for 42 days at 4 °C, and were sampled aseptically on a weekly basis. The beta-thalassemia trait was confirmed by Hb electrophoresis and molecular identification of mutations. The study was approved by the Ethics Committee of the Department of Biology, School of Science, NKUA. Investigations were carried out upon donor consent, in accordance with the principles of the Declaration of Helsinki.

### 4.2. Isolation of Membranes and Extracellular Vesicles

Hypotonic lysis was performed to isolate RBC membranes. Briefly, RBCs were lysed with hypotonic sodium phosphate buffer containing protease inhibitors, and the precipitated membranes were washed to remove the excess Hb. To evaluate the extent of tyrosine phosphorylation, stored RBCs (*n* = 3 for each donor group) were treated with 2.0–2.5 mmol/L ortho-vanadate, a known tyrosine phosphatase inhibitor, for 2 h at 37 °C, as extensively described before [68]. Untreated RBCs were used as controls, and membrane isolation was performed as described above.

At late storage, vesicles were isolated from the supernatant of the RBC units (*n* = 5 of each donor group) by high-speed centrifugation at 4 °C. Firstly, RBCs were centrifuged at 2000× *g*. Their supernatants were ultra-centrifuged at 37,000× *g* for 1 h, after passing through sterile 0.8 μm nitrocellulose filters (Millipore, Carrigtwohill, County Cork, Ireland). The pellet of vesicles was then resuspended in PBS and washed under the same conditions. Protease inhibitors were added to vesicles and their protein concentration was determined by the Bradford protein assay (Bio-Rad, Hercules, CA). The total vesicular protein per RBC unit volume was used to assess vesiculation, as previously described [67].

### 4.3. Western Blot Analysis

Equal concentrations (12–25 μg) of isolated RBC membranes (*n* = 7 per group) or vesicles (*n* = 5 per group) were loaded in Laemmli gels and transferred to nitrocellulose membranes. Primary monoclonal and polyclonal antibodies for a variety of proteins were used, along with species-specific secondary antibodies conjugated with HRP. Antibodies to the following proteins were used: band 3 (B 9277) from Sigma-Aldrich (Munich, Germany); ubiquitin (BML-PW8810) from Enzo Life Sciences (New York, NY, USA); HSP70 (sc-1060R) from Santa Cruz Biotechnology (Santa Cruz, CA, USA); and caspase-3 (#9662) and DJ-1 (#5933) from Cell Signaling Technology (Danvers, MA, USA). Antibodies against stomatin and 4.1R were kindly provided by Prof. R. Prohaska (Institute of Medical Biochemistry, University of Vienna, Austria) and Prof. J. Delaunay (Laboratoire d’ Hématologie, d’ Immunologie et de Cytogénétique, Hopital de Bicetre, Le Kremlin-Bicetre, France), respectively. The immunoblots were developed through chemiluminescence, and the bands were quantified by scanning densitometry (Gel Analyzer v.1.0, Athens, Greece). To estimate oxidative modifications of membrane and vesicular proteins, the Oxyblot kit was used, per the manufacturer’s specifications (Oxyblot, Millipore, Chemicon, Temecula, CA, USA). The proteome carbonylation index (PCI) was then calculated [68].

### 4.4. Procoagulant Activity of Extracellular Vesicles

A functional ELISA assay kit (Zymuphen MP-activity, Hyphen BioMed, Neuville-sur-Oise, France) was used to estimate the procoagulant activity of extracellular vesicles (*n* = 8 per group), per the manufacturer’s specifications and as previously described [69].

### 4.5. Scanning Electron Microscopy

Samples of RBCs (*n* = 8 per group) were firstly fixed with 2% glutaraldehyde and then with 1% osmium tetroxide in a 0.1 mol/L sodium cacodylate buffer, with a pH of 7.4. Following dehydration in a gradient of ethanol concentrations, the cells were coated with gold-palladium (Tousimis Samsputter-2a, Rockville, Maryland, USA) and were microscopically observed (Philips SEM515). Electron micrographs (magnification ×1000) were taken at randomly chosen fields, and the cell shapes were characterized as irreversible or not. A blind evaluation of at least 2000 cells was performed for each sample.

### 4.6. Proteomics Analysis

Samples (200 ng each) were loaded onto individual Evotips for desalting and then washed with 20 μL of 0.1% formic acid, followed by the addition of 100 μL of storage solvent (0.1% formic acid) to keep the Evotips wet until analysis. The Evosep One system was coupled to a timsTOF Pro mass spectrometer (Bruker Daltonics, Bremen, Germany). Data were collected over an m/z range of 100−1700 for MS and MS/MS on the timsTOF Pro instrument using an accumulation and ramp time of 100 ms. Post-processing was performed with PEAKS studio (Version X+, Bioinformatics Solutions, Waterloo, ON, USA). Graphs and statistical analyses were prepared with GraphPad Prism 8.0 (GraphPad Software, La Jolla, CA, USA) and GENE E (Broad Institute, Cambridge, MA, USA) [70].

### 4.7. Statistical Analysis and Network Preparation

Statistical analysis was performed by using the statistical package SPSS Version 22.0 (IBM Hellas, Athens, Greece, administered by NKUA). After testing for normal distribution and the presence of outliers (Shapiro−Wilk test and detrended normal Q–Q plots), the independent *t*-test was used for the evaluation of differences between groups. Correlation between the parameters of stored RBC and EVs was assessed with Pearson’s or Spearman’s tests. The R values were used for the construction of biological networks (Cytoscape 3.7.2, San Diego, CA, USA). Significance was accepted at *p* < 0.05 or *p* < 0.01 (in the case of correlation analysis).

## 5. Conclusions

The RBCs of eligible βThal^+^ donors are characterized by a low percentage of irreversible spherocytic modifications compared to the control. Their membrane proteome contains several beta thalassemia signatures related to the expression of structural, lipid raft, chaperoning, proteasome, redox, transport, antigenic, and enzyme components, including the upregulation of myosin, arginase-1, glutathione transferase, and protein kinases, but downregulation of transporters involved in nitrogen, purine, and amino acid metabolism. Some of them are reported for the first time in βThal^+^ RBCs. The overall picture is that of an efficient cellular response to a mild alpha-globin excess and oxidative/proteome stress functionally connected to resistance to malaria infection. Storage has a balancing (e.g., Na^+^/K^+^ transporter) or augmenting (e.g., HSPs) effect on these modifications, or produces new ones, including excess of skeletal proteins. The βThal^+^ EVs have normal procoagulant activity, and their composition is related in part to that of parental RBCs. Network analysis revealed interesting connections of membrane vesiculation with proteome control modulators of the RBC membrane, as well as with hemolysis of βThal^+^ RBCs. Our findings shed light on the donor variation effect on RBC storability and give hints for potential post-transfusion implications. Moreover, they might provide the basis for a more detailed exploration of RBC physiology and aging processes that occur in beta thalassemia carriers who are eligible for blood donation. Functional aspects of the presently identified variations, including proteostasis, degradome, and other important modifications that fill out the protein profile of βThal^+^ RBCs during storage, are currently underway in our labs.

## Data Availability

The data presented in this study are available online at www.mdpi.com/xxx/s1 (Supplementary Tables S1–S3).

## Supplementary Materials

Supplementary tables are available online at https://www.mdpi.com/1422-0067/22/7/3369/s1.
