# Supplementary material for: Proteome of Stored RBC Membrane and Vesicles from Heterozygous Beta Thalassemia Donors
Source: Int J Mol Sci. 2021 Mar 25;22(7):3369. doi: 10.3390/ijms22073369 (PMC8037027; doi:10.3390/ijms22073369)
Supplement: Supplementary file 1 [file ijms-22-03369-s001.zip › Supplementary Table S4.pdf]

Table S4: Major RBC components of storage EVs and their relation to membrane values

	EVs (A.U.)		p-value	EV/membrane ratio*	
Protein	βThal⁺	Control	(βThal⁺ vs. C)	βThal⁺	Control
**Hb alpha chains**	1,187±159	1,125±137	0.526	**6.630**	**6.125**
**Hb beta chains**	975±56	978±114	0.965	**9.559**	**8.969**
Stomatin	354±145	340±93	0.858	0.930	0.956
Band 3	297±134	245±51	0.448	0.254	0.212
Ankyrin	237±29	216±21	0.228	0.091	0.090
**Valosin containing protein**	189±18	188±11	0.853	**1.333**	**1.460**
**Carbonic anhydrase-1**	193±63	137±23	0.100	**9.482**	**9.935**
Peroxiredoxin-2	160±72	128±49	0.432	0.965	0.889
**Catalase**	118±20	105±31	0.467	**1.723**	**1.625**
Protein 4.1R	86±35	79±11	0.685	0.096	0.090
**Flavin reductase**	105±26	78±10	0.030	**3.488**	**2.630**

(*) day 42 of storage. Bold: ratio > 1.25
