# Supplementary material for: Proteome of Stored RBC Membrane and Vesicles from Heterozygous Beta Thalassemia Donors
Source: Int J Mol Sci. 2021 Mar 25;22(7):3369. doi: 10.3390/ijms22073369 (PMC8037027; doi:10.3390/ijms22073369)
Supplement: Supplementary file 1 [file ijms-22-03369-s001.zip › Supplementary Table S5.pdf]

Table S5: Proteins with a trend for different expression levels in βThal[+] EVs vs. control on day 42 of storage

Protein	βThal[+] EVs	Control EVs	p-value
Carbonic anhydrase 1	192.80±63.43	136.60±23.39	0.100
Complement C3	43.80±17.74	83.40±39.26	0.074
Glyceraldehyde 3-phosphate dehydrogenase	41.00±3.81	33.80±7.01	0.078
Immunoglobulin kappa constant	31.67±5.69	39.00±3.74	0.092
Transferrin receptor	41.50±16.60	8.50±4.95	0.059
Hemopexin	25.20±6.26	33.20±6.91	0.091
Porphobilinogen deaminase	16.00±6.28	9.25±1.50	0.077
Aspartate aminotransferase	10.20±2.95	6.60±1.95	0.052
Coagulation factor V	4.33±2.52	14.50±6.76	0.059
CD44 antigen	7.80±1.79	6.00±0.71	0.070
Ribose-phosphate pyrophosphokinase 1	6.40±2.07	8.50±0.58	0.093
Proteasome subunit alpha type-1	4.67±1.53	2.33±0.58	0.069
Proteasome subunit alpha type-2	7.20±2.95	3.75±1.26	0.067
