# Supplementary material for: Proteome of Stored RBC Membrane and Vesicles from Heterozygous Beta Thalassemia Donors
Source: Int J Mol Sci. 2021 Mar 25;22(7):3369. doi: 10.3390/ijms22073369 (PMC8037027; doi:10.3390/ijms22073369)
Supplement: Supplementary file 1 [file ijms-22-03369-s001.zip › Supplementary Table S6.pdf]

Supplementary Table S6. Numerical code used for the presentation of RBC and supernatant measurements, including RBC and EVs proteome data, in the biological networks shown in Figures 10 and 11.

Parameter	Abbreviation
Alpha-1B-glycoprotein (A1BG)	1
Alpha-2-macroglobulin (A2M)	2
ATP-binding cassette sub-family B member 6 (ABCB6)	3
Multidrug resistance-associated protein 1 (ABCC1)	4
Multidrug resistance-associated protein 4 (ABCC4)	5
Multidrug resistance-associated protein 5 (ABCC5)	6
ATP-binding cassette sub-family G member 2 (ABCG2)	7
Acetylcholinesterase (ACHE)	8
ATP-citrate synthase (ACLY)	9
Long-chain-fatty-acid--CoA ligase 3 (ACSL3)	10
Long-chain-fatty-acid--CoA ligase 4 (ACSL4)	11
Long-chain-fatty-acid--CoA ligase 6 (ACSL6)	12
Actin, alpha (ACTA1)	13
Actin, cytoplasmic 1 (ACTB)	14
Beta-actin-like protein 2 (ACTBL2)	15
Actin, cytoplasmic 2 (ACTG1)	16
Alpha-centractin (ACTR1A)	17
Alpha-adducin (ADD1)	18
Beta-adducin (ADD2)	19
Gamma-adducin (ADD3)	20
Protein argonaute-2 (AGO2)	21
Adenosylhomocysteinase (AHCY)	22
Alpha-2-HS-glycoprotein (AHSG)	23
Alpha-hemoglobin-stabilizing protein (AHSP)	24
Adenylate kinase isoenzyme 1 (AK1)	25
Delta-aminolevulinic acid dehydratase (ALAD)	26
Serum albumin (ALB)	27
Aldehyde dehydrogenase family 16 member A1 (ALDH16A1)	28
Retinal dehydrogenase 1 (ALDH1A1)	29
Fructose-bisphosphate aldolase A (ALDOA)	30
Fructose-bisphosphate aldolase C (ALDOC)	31
Ankyrin-1 (ANK1)	32
Annexin A4 (ANXA4)	33
Annexin A4 (ANXA4)	34
Annexin A7 (ANXA7)	35
AP-1 complex subunit beta-1 (AP1B1)	36
AP-2 complex subunit alpha-1 (AP2A1)	37
AP-2 complex subunit alpha-2 (AP2A2)	38
AP-2 complex subunit beta (AP2B1)	39
AP-2 complex subunit mu (AP2M1)	40
Acylamino-acid-releasing enzyme (APEH)	41

Apolipoprotein A-I (APOA1)	42
Apolipoprotein A-II (APOA2)	43
Apolipoprotein A-IV (APOA4)	44
Apolipoprotein B-100 (APOB)	45
Apolipoprotein E (APOE)	46
Beta-2-glycoprotein 1 (APOH)	47
Aquaporin-1 (AQP1)	48
ADP-ribosylation factor 1 (ARF1)	49
Arginase-1 (ARG1)	50
Atlastin-3 (ATL3)	51
Sodium/potassium-transporting ATPase subunit alpha-1 (ATP1A1)	52
Plasma membrane calcium-transporting ATPase 1 (ATP2B1)	53
Plasma membrane calcium-transporting ATPase 4 (ATP2B4)	54
Copper-transporting ATPase 1 (ATP7A)	55
Basal cell adhesion molecule (BCAM)	56
Flavin reductase (BLVRB)	57
BMP-2-inducible protein kinase (BMP2K)	58
Bisphosphoglycerate mutase (BPGM)	59
Basigin (BSG)	60
Phospholipid transfer protein (C2CD2L)	61
Small membrane A-kinase anchor protein (C2orf88)	62
Complement C3 (C3)	63
Complement C4-B (C4B)	64
C4b-binding protein alpha chain (C4BPA)	65
Intracellular calcium levels	66
Carbonic anhydrase 1 (CA1)	67
Carbonic anhydrase 2 (CA2)	68
Carbonic anhydrase 3 (CA3)	69
Calmodulin-1 (CALM1)	70
Calreticulin (CALR)	71
Cullin-associated NEDD8-dissociated protein 1 (CAND1)	72
Calnexin (CANX)	73
Calpain-1 catalytic subunit (CAPN1)	74
Calpain-5 (CAPN5)	75
Calpain small subunit 1 (CAPNS1)	76
F-actin-capping protein subunit alpha-1 (CAPZA1)	77
F-actin-capping protein subunit beta (CAPZB)	78
Caspase 3 (Immunoblot)	79
Calpastatin (CAST)	80
Catalase (CAT)	81
Coiled-coil and C2 domain-containing protein 1A (CC2D1A)	82
T-complex protein 1 subunit beta (CCT2)	83
T-complex protein 1 subunit gamma (CCT3)	84
T-complex protein 1 subunit delta (CCT4)	85

T-complex protein 1 subunit epsilon (CCT5)	86
T-complex protein 1 subunit zeta (CCT6A)	87
T-complex protein 1 subunit eta (CCT7)	88
T-complex protein 1 subunit theta (CCT8)	89
CD44 antigen (CD44)	90
Complement decay-accelerating factor (CD55)	91
Lymphocyte function-associated antigen 3 (CD58)	92
CD59 glycoprotein (CD59)	93
CD5 antigen-like (CD5L)	94
Cell division control protein 42 homolog (CDC42)	95
Complement factor B (CFB)	96
Cofilin-1 (CFL1)	97
Charged multivesicular body protein 4b (CHMP4B)	98
Clathrin heavy chain 1 (CLTC)	99
Clusterin (CLU)	100
2',3'-cyclic-nucleotide 3'-phosphodiesterase (CNP)	101
CB1 cannabinoid receptor-interacting protein 1 (CNRIP1)	102
COP9 signalosome complex subunit 2 (COPS2)	103
COP9 signalosome complex subunit 3 (COPS3)	104
COP9 signalosome complex subunit 4 (COPS4)	105
COP9 signalosome complex subunit 6 (COPS6)	106
Ceruloplasmin (CP)	107
Copine-3 (CPNE3)	108
Complement receptor type 1 (CR1)	109
Casein kinase I isoform alpha (CSNK1A1)	110
Cullin-1 (CUL1)	111
NADH-cytochrome b5 reductase 3 (CYB5R3)	112
Disheveled-associated activator of morphogenesis 1 (DAAM1)	113
Dynactin subunit 1 (DCTN1)	114
Dynactin subunit 2 (DCTN2)	115
Protein DDI1 homolog 2 (DDI2)	116
Deoxyribose-phosphate aldolase (DERA)	117
Diamide-induced intracellular ROS levels	118
Protein diaphanous homolog 1 (DIAPH1)	119
Dematin (DMTN)	120
DnaJ homolog subfamily B member 1 (DNAJB1)	121
DnaJ homolog subfamily C member 13 (DNAJC13)	122
Dynamin-2 (DNM2)	123
Proteasome adapter and scaffold protein ECM29 (ECPAS)	124
Elongation factor 1-alpha 1 (EEF1A1)	125
EH domain-binding protein 1-like protein 1 (EHBP1L1)	126
EH domain-containing protein 1 (EHD1)	127
Eukaryotic translation initiation factor 5A-1 (EIF5A)	128
Endonuclease domain-containing 1 protein (ENDOD1)	129

Alpha-enolase (ENO1) 130
Protein 4.1 (EPB41) 131
Band 4.1-like protein 2 (EPB41L2) 132
Erythrocyte membrane protein band 4.2 (EPB42) 133
Epoxide hydrolase 1 (EPHX1) 134
Erythroid membrane-associated protein (ERMAP) 135
Endoplasmic reticulum resident protein 44 (ERP44) 136
S-formylglutathione hydrolase (ESD) 137
EV Protein Carbonylation 138
Ezrin (EZR) 139
Junctional adhesion molecule A (F11R) 140
Prothrombin (F2) 141
Fatty acid synthase (FASN) 142
Fibrinogen alpha chain (FGA) 143
Fibrinogen beta chain (FGB) 144
Fibrinogen gamma chain (FGG) 145
Peptidyl-prolyl cis-trans isomerase (FKBP3) 146
Flotillin-1 (FLOT1) 147
Flotillin-2 (FLOT2) 148
Storage Hemolysis 149
Glyceraldehyde-3-phosphate dehydrogenase (GAPDH) 150
GTPase-activating protein and VPS9 domain-containing protein 1 (GAPVD1) 151
Vitamin D-binding protein (GC) 152
Rab GDP dissociation inhibitor beta (GDI2) 153
Golgi-associated plant pathogenesis-related protein 1 (GLIPR2) 154
GMP reductase 1 (GMPR) 155
Guanine nucleotide-binding protein subunit alpha-13 (GNA13) 156
Guanine nucleotide-binding protein G(i) subunit alpha-2 (GNAI2) 157
Guanine nucleotide-binding protein G(k) subunit alpha (GNAI3) 158
Guanine nucleotide-binding protein G(q) subunit alpha (GNAQ) 159
Guanine nucleotide-binding protein G(s) subunit alpha isoforms XLas (GNAS) 160
Guanine nucleotide-binding protein G(I)/G(S)/G(T) subunit beta-1 (GNB1) 161
Guanine nucleotide-binding protein G(I)/G(S)/G(T) subunit beta-2 (GNB2) 162
Aspartate aminotransferase (GOT1) 163
G protein-coupled receptor kinase 6 (GRK6) 164
Glutathione S-transferase omega-1 (GSTO1) 165
Glycophorin-A (GYPA) 166
Glycophorin-C (GYPC) 167
Hemoglobin (g/dL) 168
Hemoglobin subunit alpha (HBA1) 169
Hemoglobin subunit beta (HBB) 170
Hemoglobin subunit delta (HBD) 171
Hemoglobin subunit gamma-1 (HBG1) 172
Hemoglobin subunit theta-1 (HBQ1) 173

Hematocrit	174
Heme-binding protein 1 (HEBP1)	175
Porphobilinogen deaminase (HMBS)	176
Haptoglobin (HP)	177
Hypoxanthine-guanine phosphoribosyltransferase (HPRT1)	178
Hemopexin (HPX)	179
Very-long-chain 3-oxoacyl-CoA reductase (HSD17B12)	180
Heat shock protein HSP 90-alpha (HSP90AA1)	181
Heat shock 70 kDa protein 1A (HSPA1A)	182
Heat shock 70 kDa protein 4 (HSPA4)	183
Endoplasmic reticulum chaperone BiP (HSPA5)	184
Heat shock cognate 71 kDa protein (HSPA8)	185
E3 ubiquitin-protein ligase (HUWE1)	186
Intercellular adhesion molecule 4 (ICAM4)	187
Immunoglobulin gamma-1 heavy chain	188
Immunoglobulin gamma-1 heavy chain	189
Immunoglobulin heavy constant alpha 1 (IGHA1)	190
Immunoglobulin heavy constant gamma 2 (IGHG2)	191
Immunoglobulin heavy constant gamma 3 (IGHG3)	192
Immunoglobulin heavy constant mu (IGHM)	193
Immunoglobulin kappa light chain	194
Immunoglobulin kappa light chain	195
Immunoglobulin kappa constant (IGKC)	196
Immunoglobulin lambda-1 light chain	197
Immunoglobulin lambda-like polypeptide 5 (IGLL5)	198
Importin-5 (IPO5)	199
Importin-7 (IPO7)	200
Inter-alpha-trypsin inhibitor heavy chain H4 (ITIH4)	201
Extracellular K^+ levels	202
Intermediate conductance calcium-activated potassium channel protein 4 (KCNN4)	203
Kell blood group glycoprotein (KEL)	204
Importin subunit beta-1 (KPNB1)	205
GTPase KRas (KRAS)	206
Glutathione S-transferase (LANCL1)	207
LanC-like protein 2 (LANCL2)	208
L-lactate dehydrogenase B chain (LDHB)	209
Galectin-3 (LGALS3)	210
Vesicular integral-membrane protein VIP36 (LMAN2)	211
Mean corpuscular fragility index (MCF)	212
MCH	213
MCHC	214
MCV	215
Membrane lipid peroxidation	216
Malate dehydrogenase (MDH1)	217

Methyltransferase-like protein 7A (METTL7A) 218
Mechanical fragility index (MFI) 219
Multiple inositol polyphosphate phosphatase 1(MINPP1) 220
55 kDa erythrocyte membrane protein (MPP1) 221
Moesin (MSN) 222
C-1-tetrahydrofolate synthase (MTHFD1) 223
Myosin-10 (MYH10) 224
Myosin-9 (MYH9) 225
Myosin regulatory light chain 12B (MYL12B) 226
Myosin light chain 4 (MYL4) 227
Unconventional myosin-XVIIIa (MYO18A) 228
Extracellular Na^+ levels 229
Nucleosome assembly protein 1-like 1 (NAP1L1) 230
Nucleosome assembly protein 1-like 4 (NAP1L4) 231
Alpha-soluble NSF attachment protein (NAPA) 232
Neutral cholesterol ester hydrolase 1 (NCEH1) 233
Nucleoside diphosphate kinase A (NME1) 234
GTPase NRas (NRAS) 235
Vesicle-fusing ATPase (NSF) 236
Obg-like ATPase 1 (OLA1) 237
Alpha-1-acid glycoprotein 1 (ORM1) 238
Alpha-1-acid glycoprotein 2 (ORM2) 239
Oxidative hemolysis 240
Protein disulfide-isomerase (P4HB) 241
Proliferation-associated protein 2G4 (PA2G4) 242
Protein/nucleic acid deglycase DJ-1 (PARK7) 243
Protein-L-isoaspartate(D-aspartate) O-methyltransferase (PCMT1) 244
Programmed cell death 6-interacting protein (PDCD6IP) 245
Protein disulfide-isomerase A3 (PDIA3) 246
Protein disulfide-isomerase A6 (PDIA6) 247
Phosphatidylethanolamine-binding protein 1 (PEBP1) 248
ATP-dependent 6-phosphofructokinase, liver type (PFKL) 249
ATP-dependent 6-phosphofructokinase, muscle type (PFKM) 250
6-phosphogluconate dehydrogenase, decarboxylating (PGD) 251
Phosphoglycerate kinase 1 (PGK1) 252
Phenylhydrazine-induced intracellular ROS levels 253
Phosphatidylinositol 4-kinase type 2-alpha (PI4K2A) 254
Phosphatidylinositol-binding clathrin assembly protein (PICALM) 255
Piezo-type mechanosensitive ion channel component 1 (PIEZO1) 256
Phosphatidylinositol 5-phosphate 4-kinase type-2 alpha (PIP4K2A) 257
Phospholipid scramblase 1 (PLSCR1) 258
Purine nucleoside phosphorylase (PNP) 259
Neuropathy target esterase (PNPLA6) 260
Peptidyl-prolyl cis-trans isomerase A (PPIA) 261

Peptidyl-prolyl cis-trans isomerase B (PPIB) 262
Serine/threonine-protein phosphatase 2A 65 kDa regulatory subunit A alpha isoform (PPP2R1A) 263
Peroxiredoxin-1 (PRDX1) 264
Peroxiredoxin-2 (PRDX2) 265
Peroxiredoxin-6 (PRDX6) 266
cAMP-dependent protein kinase catalytic subunit alpha (PRKACA) 267
cAMP-dependent protein kinase type I-alpha regulatory subunit (PRKAR1A) 268
Protein arginine N-methyltransferase 5 (PRMT5) 269
Ribose-phosphate pyrophosphokinase 1 (PRPS1) 270
Phosphoribosyl pyrophosphate synthase-associated protein 2 (PRPSAP2) 271
Externalized phosphatidylserine levels 272
Proteasome subunit alpha type-1 (PSMA1) 273
Proteasome subunit alpha type-2 (PSMA2) 274
Proteasome subunit alpha type-3 (PSMA3) 275
Proteasome subunit alpha type-4 (PSMA4) 276
Proteasome subunit alpha type-5 (PSMA5) 277
Proteasome subunit alpha type-6 (PSMA6) 278
Proteasome subunit alpha type-7 (PSMA7) 279
Proteasome subunit beta type-1 (PSMB1) 280
Proteasome subunit beta type-2 (PSMB2) 281
Proteasome subunit beta type-3 (PSMB3) 282
Proteasome subunit beta type-4 (PSMB4) 283
Proteasome subunit beta type-5 (PSMB5) 284
Proteasome subunit beta type-7 (PSMB7) 285
26S proteasome regulatory subunit 4 (PSMC1) 286
26S proteasome regulatory subunit 7 (PSMC2) 287
26S proteasome regulatory subunit 6A (PSMC3) 288
26S proteasome regulatory subunit 6B (PSMC4) 289
26S proteasome regulatory subunit 8 (PSMC5) 290
26S proteasome regulatory subunit 10B (PSMC6) 291
26S proteasome non-ATPase regulatory subunit 1 (PSMD1) 292
26S proteasome non-ATPase regulatory subunit 11 (PSMD11) 293
26S proteasome non-ATPase regulatory subunit 12 (PSMD12) 294
26S proteasome non-ATPase regulatory subunit 13 (PSMD13) 295
26S proteasome non-ATPase regulatory subunit 14 (PSMD14) 296
26S proteasome non-ATPase regulatory subunit 2 (PSMD2) 297
26S proteasome non-ATPase regulatory subunit 3 (PSMD3) 298
26S proteasome non-ATPase regulatory subunit 5 (PSMD5) 299
26S proteasome non-ATPase regulatory subunit 6 (PSMD6) 300
26S proteasome non-ATPase regulatory subunit 7 (PSMD7) 301
26S proteasome non-ATPase regulatory subunit 8 (PSMD8) 302
Proteasome activator complex subunit 1 (PSME1) 303
Proteasome activator complex subunit 2 (PSME2) 304
Polypyrimidine tract-binding protein 1 (PTBP1) 305

Phosphorylated tyrosine levels 306
Pyrroline-5-carboxylate reductase 3 (PYCR3) 307
Ras-related protein Rab-10 (RAB10) 308
Ras-related protein Rab-18 (RAB18) 309
Ras-related protein Rab-1B (RAB1B) 310
Ras-related protein Rab-21 (RAB21) 311
Ras-related protein Rab-2B (RAB2B) 312
Ras-related protein Rab-35 (RAB35) 313
Ras-related protein Rab-35 (RAB35) 314
Ras-related protein Rab-5C (RAB5C) 315
Ras-related protein Rab-7a (RAB7A) 316
Ras-related protein Rab-8A (RAB8A) 317
Ras-related protein Rab-8B (RAB8B) 318
Ras-related C3 botulinum toxin substrate 1 (RAC1) 319
UV excision repair protein RAD23 homolog A (RAD23A) 320
Ras-related protein Ral-A (RALA) 321
GTP-binding nuclear protein Ran (RAN) 322
Ras-related protein Rap-1A (RAP1A) 323
Ras-related protein Rap-1b (RAP1B) 324
Ras-related protein Rap-2a (RAP2A) 325
Ras-related protein Rap-2b (RAP2B) 326
RBC count 327
RDW 328
Radixin (RDX) 329
Ammonium transporter Rh type A (RHAG) 330
Blood group Rh(CE) polypeptide (RHCE) 331
Transforming protein RhoA (RHOA) 332
Ribonuclease inhibitor (RNH1) 333
Intracellular ROS levels 334
Protein XRP2 (RP2) 335
RuvB-like 1 (RUVBL1) 336
RuvB-like 2 (RUVBL2) 337
Phosphatidylinositide phosphatase SAC1 (SACM1L) 338
Vesicle-trafficking protein SEC22b (SEC22B) 339
Methanethiol oxidase (SELENBP1) 340
Semaphorin-7A (SEMA7A) 341
Alpha-1-antitrypsin (SERPINA1) 342
Alpha-1-antichymotrypsin (SERPINA3) 343
Antithrombin-III (SERPINC1) 344
SH3 domain-binding glutamic acid-rich-like protein (SH3BGRL) 345
SH3 domain-binding glutamic acid-rich-like protein 2 (SH3BGRL2) 346
Urea transporter 1 (SLC14A1) 347
Monocarboxylate transporter 1 (SLC16A1) 348
Equilibrative nucleoside transporter 1 (SLC29A1) 349

Solute carrier family 2, facilitated glucose transporter member 1 (SLC2A1) 350
Zinc transporter 1 (SLC30A1) 351
Solute carrier family 40 member 1 (SLC40A1) 352
Band 3 anion transport protein (SLC4A1) 353
Small integral membrane protein 1 (SMIM1) 354
Alpha-synuclein (SNCA) 355
Superoxide dismutase [Cu-Zn] (SOD1) 356
Sorbitol dehydrogenase (SORD) 357
Spectrin alpha chain, erythrocytic 1 (SPTA1) 358
Spectrin beta chain, erythrocytic (SPTB) 359
Sorcin (SRI) 360
Hsc70-interacting protein (ST13) 361
Stress-induced-phosphoprotein 1 (STIP1) 362
Erythrocyte band 7 integral membrane protein (STOM) 363
Syntaxin-7 (STX7) 364
Total antioxidant capacity of the supernatant 365
Uric acid independent antioxidant capacity of the supernatant 366
Serine/threonine-protein kinase TAO3 (TAOK3) 367
TBC1 domain family member 24 (TBC1D24) 368
Tert-butyl hydroperoxide-induced intracellular ROS levels 369
T-complex protein 1 subunit alpha (TCP1) 370
Serotransferrin (TF) 371
Transmembrane emp24 domain-containing protein 2 (TMED2) 372
Transmembrane protein 222 (TMEM222) 373
Tropomodulin-1 (TMOD1) 374
Tensin-1 (TNS1) 375
Triosephosphate isomerase (TPI1) 376
Tropomyosin alpha-1 (TPM1) 377
Tripeptidyl-peptidase 2 (TPP2) 378
Thioredoxin (TXN) 379
Uric acid dependent antioxidant capacity of the supernatant 380
Ubiquitin-like modifier-activating enzyme 1 (UBA1) 381
Polyubiquitin-B (UBB) 382
Ubiquitin-conjugating enzyme E2 N (UBE2N) 383
E2 ubiquitin-conjugating enzyme (UBE2O) 384
E3 ubiquitin-protein ligase UBR4 (UBR4) 385
UBX domain-containing protein 6 (UBXN6) 386
UDP-glucose:glycoprotein glucosyltransferase 1 (UGGT1) 387
Ubiquitin carboxyl-terminal hydrolase 14 (USP14) 388
Ubiquitin carboxyl-terminal hydrolase 15 (USP15) 389
Ubiquitin carboxyl-terminal hydrolase 5 (USP5) 390
Probable ubiquitin carboxyl-terminal hydrolase FAF-X (USP9X) 391
Vesicle-associated membrane protein 3 (VAMP3) 392
Vesicle-associated membrane protein-associated protein A (VAPA) 393

Synaptic vesicle membrane protein VAT-1 homolog (VAT1) 394
Transitional endoplasmic reticulum ATPase (VCP) 395
EVs Protein Concentration 396
Vacuolar protein sorting-associated protein 13A (VPS13A) 397
WD repeat-containing protein 81 (WDR81) 398
WD repeat-containing protein 91 (WDR91) 399
Exportin-7 (XPO7) 400
14-3-3 protein epsilon (YWHAE) 401
14-3-3 protein zeta/delta (YWHAZ) 402
