# Supplementary material for: Proteome of Stored RBC Membrane and Vesicles from Heterozygous Beta Thalassemia Donors
Source: Int J Mol Sci. 2021 Mar 25;22(7):3369. doi: 10.3390/ijms22073369 (PMC8037027; doi:10.3390/ijms22073369)
Supplement: Supplementary file 1 [file ijms-22-03369-s001.zip › Supplementary Table S7.pdf]

**Supplementary Table S7.** RBC properties and RBC membrane components connected to EV characteristics.

| RBC parameter | Number of connections with EVs | |
|---|---|---|
| | βThal$^+$ | Control |
| Proteasome | 163 | 99 |
| Small GTPases | 90 | 139 |
| IgGs | 70 | 28 |
| Kinases | 36 | 49 |
| Molecular chaperones | 36 | 39 |
| Glutaredoxin SH3BGRL | 23 | 9 |
| SH3 domain-binding glutamic acid-rich-like protein | 23 | 9 |
| $Ca^{2+}$-regulated proteins | 21 | 20 |
| Phosphatases | 17 | 2 |
| Long chain fatty acid-CoA ligase 6 | 17 | 12 |
| MCH/MCHC | 17 | 6 |
| Glycophorin A | 15 | 5 |
| Spectrins | 15 | 3 |
| Synnexin | 15 | 5 |
| Total antioxidant capacity (supernatant) | 14 | 6 |
| Diamide-induced ROS | 3 | 16 |
| Oxidative hemolysis | 15 | - |
| Deoxyribose-phosphate aldolase | 13 | - |
| Spontaneous storage hemolysis | 12 | - |
