# Supplementary material for: Proteome of Stored RBC Membrane and Vesicles from Heterozygous Beta Thalassemia Donors
Source: Int J Mol Sci. 2021 Mar 25;22(7):3369. doi: 10.3390/ijms22073369 (PMC8037027; doi:10.3390/ijms22073369)
Supplement: Supplementary file 1 [file ijms-22-03369-s001.zip › Supplementary Table S8.pdf]

Supplementary Table S8. EV hubs prevailing in the biological networks of Figure 10 and Figure 11 and their connectivity to RBC-related parameters.

A. βThal^{+} EVs	Number of connections to RBC parameters
**Apolipoproteins**	**81**
**Hb**	**52**
**Small integral membrane protein 1 (SMIM1)**	**41**
**Carbonic anhydrase 1**	**36**
Flavin reductase	30
Thioredoxin	30
**IgGs**	**28**
GTPase activating protein (TBC1D24)	28
**Ubiquitin-related**	**28**
Fibrinogen	26
Sorcin	23
Phosphatidylethanolamine-binding protein (PEBP1)	22
α-hemoglobin-stabilizing protein (AHSP)	21
Peroxiredoxins	21
Phosphatidylinositol 5P 4-kinase type-2α (PIP4K2A)	20
Prothrombin	18
CD55	16
**Small GTPases**	**15**
**B. Control EVs**	**Number of connections to RBC parameters**
**Apolipoproteins**	**40**
Spectrins	38
**Ubiquitin-related**	**29**
S-formyl-glutathione hydrolase (ESD)	28
**Carbonic anhydrase 1**	**27**
Glycophorin A	26
**IgGs**	**26**
**Hb**	**22**
Heat shock proteins	22
**Small GTPases**	**20**
Calpastatin	19
Complement	18
Inter-α-trypsin inhibitor heavy chain (H4ITIH4)	18
**Small integral membrane protein 1 (SMIM1)**	**15**

Bold: high connectivity parameters present in both groups.
