# Supplementary material for: Proteome of Stored RBC Membrane and Vesicles from Heterozygous Beta Thalassemia Donors
Source: Int J Mol Sci. 2021 Mar 25;22(7):3369. doi: 10.3390/ijms22073369 (PMC8037027; doi:10.3390/ijms22073369)
Supplement: Supplementary file 1 [file ijms-22-03369-s001.zip › Table S1 - Proteomics analysis of RBC membranes.pdf]

#	Identified Proteins (1098)	Accession Number	Alternate ID	Molecular Weight	Total Spectrum Count																																													
					Control Day 7											Control Day 42												beta-Thalassemia Heterozygotes Day 7												beta-Thalassemia Heterozygotes Day 42											
1	Spectrin beta chain, erythrocytic OS=Homo sapiens OX=9606 GN=SPTB PE=1 SV=5	SPTB1_HUMAN	SPTB	246 kDa	4099	4086	4370	4009	3822	3966	4192	3919	4017	3840	4212	3490	3117	3132	3688	3646	3446	3171	3499	3414	3430	3552	3037	3144	3713	3223	3449	3549	3875	3653	3826	3975	4169	4098	4103	3904	3757	3816	3485	3781	3612	3866	3548	3532	3712	3257	4167
2	Spectrin alpha chain, erythrocytic 1 OS=Homo sapiens OX=9606 GN=SPTA1 PE=1 SV=5	SPTA1_HUMAN	SPTA1	280 kDa	3690	3643	4036	3754	3693	3788	3947	3556	3949	3797	3975	3541	3138	3257	3741	3460	3517	3284	3592	3487	3369	3503	2988	3377	3580	3398	3399	3467	3525	3318	3772	3698	3920	3675	3812	3676	3382	3576	3528	3643	3659	3895	3604	3583	3684	3403	3784
3	Ankyrin-1 OS=Homo sapiens OX=9606 GN=ANK1 PE=1 SV=3	ANK1_HUMAN	ANK1	206 kDa	2780	2662	2911	2675	2686	2760	2762	2576	2784	2693	2962	2528	2289	2293	2587	2589	2442	2299	2552	2435	2377	2504	2094	2270	2589	2453	2455	2523	2677	2516	2684	2742	2924	2759	2854	2639	2512	2603	2581	2641	2635	2801	2493	2606	2605	2357	2805
4	Band 3 anion transport protein OS=Homo sapiens OX=9606 GN=SLC4A1 PE=1 SV=3	B3AT_HUMAN	SLC4A1	102 kDa	1294	1265	1312	1220	1176	1212	1299	1222	1273	1263	1385	1179	1162	1118	1205	1190	1181	1078	1180	1173	1148	1208	1060	1064	1118	1055	1061	1090	1170	1124	1175	1214	1306	1371	1383	1241	1157	1150	1119	1164	1111	1206	1192	1146	1182	1127	1241
5	Glyceraldehyde-3-phosphate dehydrogenase OS=Homo sapiens OX=9606 GN=GAPDH PE=1 SV=3	G3P_HUMAN	GAPDH	36 kDa	1157	1234	1218	1122	1127	1154	1207	1110	1109	944	1038	1048	999	1006	1139	1145	1209	1021	1146	1026	1092	1144	958	1004	975	1038	986	1194	1137	1159	1298	1188	1264	1182	1171	1273	1016	1235	1108	1286	1188	1298	1114	1203	1073	1023	1350
6	Protein 4.1 OS=Homo sapiens OX=9606 GN=EPB41 PE=1 SV=4	41_HUMAN	EPB41	97 kDa	848	910	993	935	856	901	909	879	949	871	941	935	849	858	971	922	899	852	905	878	854	906	802	817	889	876	829	874	924	889	917	898	909	914	944	834	854	845	890	912	898	967	866	935	925	934	964
7	Erythrocyte membrane protein band 4.2 OS=Homo sapiens OX=9606 GN=EPB42 PE=1 SV=3	EPB42_HUMAN	EPB42	77 kDa	684	713	761	807	688	754	677	702	735	649	825	694	613	599	731	728	677	610	698	671	670	712	601	613	687	619	582	626	721	706	687	639	759	735	763	740	785	690	718	796	788	787	668	702	742	652	804
8	Actin, cytoplasmic 1 OS=Homo sapiens OX=9606 GN=ACTB PE=1 SV=1	ACTB_HUMAN	ACTB	42 kDa	677	677	742	646	665	756	694	673	692	613	722	617	576	591	621	609	628	625	619	623	575	632	589	572	600	582	594	618	577	597	676	647	719	714	719	658	554	633	602	623	620	635	629	613	614	595	644
9	Erythrocyte band 7 integral membrane protein OS=Homo sapiens OX=9606 GN=STOM PE=1 SV=3	STOM_HUMAN	STOM	32 kDa	470	442	476	391	390	452	423	417	456	431	431	351	342	340	374	359	364	352	333	322	372	384	377	374	393	345	391	411	376	413	421	370	433	433	462	374	379	397	369	393	401	423	376	373	373	346	371
10	Beta-adducin OS=Homo sapiens OX=9606 GN=ADD2 PE=1 SV=3	ADDB_HUMAN	ADD2	81 kDa	364	331	360	309	356	356	362	312	420	379	371	367	329	357	361	339	362	358	388	354	389	408	302	407	334	372	370	350	273	313	357	337	431	376	376	388	307	406	351	324	300	383	415	372	343	327	319
11	Flotillin-2 OS=Homo sapiens OX=9606 GN=FLOT2 PE=1 SV=2	FLOT2_HUMAN	FLOT2	47 kDa	338	369	363	335	311	347	361	364	366	360	395	336	298	291	335	330	330	300	289	290	307	337	291	319	343	313	332	342	335	356	347	344	378	387	386	366	338	347	348	353	339	361	311	343	347	319	356
12	Fructose-bisphosphate aldolase A OS=Homo sapiens OX=9606 GN=ALDOA PE=1 SV=2	ALDOA_HUMAN	ALDOA	39 kDa	194	216	175	233	208	259	334	328	273	213	216	255	245	253	280	244	247	247	215	232	243	259	220	227	227	263	226	259	258	242	309	225	209	224	161	297	240	268	309	301	314	275	270	343	301	275	389
13	55 kDa erythrocyte membrane protein OS=Homo sapiens OX=9606 GN=MPP1 PE=1 SV=2	EM55_HUMAN	MPP1	52 kDa	241	252	284	315	270	292	287	313	284	278	250	227	197	200	267	220	226	219	250	244	214	223	192	227	250	238	199	260	235	228	277	280	259	261	243	235	183	200	253	237	256	258	189	246	240	220	302
14	Flotillin-1 OS=Homo sapiens OX=9606 GN=FLOT1 PE=1 SV=3	FLOT1_HUMAN	FLOT1	47 kDa	278	255	307	202	194	267	278	250	245	238	236	266	216	221	277	229	216	213	204	214	210	243	195	253	248	273	219	258	236	249	257	213	247	286	241	240	188	215	257	262	248	271	215	270	286	282	257
15	Hemoglobin subunit alpha OS=Homo sapiens OX=9606 GN=HBA1 PE=1 SV=2	HBA_HUMAN	HBA1	15 kDa	258	182	116	164	236	221	208	234	223	192	173	208	145	192	123	113	137	239	256	170	242	188	191	207	127	316	268	240	122	161	163	159	131	106	147	106	97	204	217	147	162	145	186	211	223	287	164
16	Alpha-adducin OS=Homo sapiens OX=9606 GN=ADD1 PE=1 SV=2	ADDA_HUMAN	ADD1	81 kDa	217	207	221	166	181	203	218	176	207	205	210	178	169	176	175	165	169	181	179	143	179	191	165	177	182	186	199	199	158	171	201	181	215	187	197	172	155	212	171	148	165	191	209	177	181	145	171
17	Glycophorin-C OS=Homo sapiens OX=9606 GN=GYPC PE=1 SV=1	GLPC_HUMAN	GYPC	14 kDa	235	244	261	214	223	200	234	215	210	210	223	170	164	158	182	179	167	150	173	165	168	172	147	159	223	170	184	212	232	200	214	223	222	218	201	177	209	184	175	189	178	199	166	170	177	174	233
18	Protein argonaute-2 OS=Homo sapiens OX=9606 GN=AGO2 PE=1 SV=3	AGO2_HUMAN	AGO2	97 kDa	143	150	167	170	132	168	159	150	165	156	162	174	154	158	159	139	150	149	150	164	148	161	134	137	164	176	171	156	178	161	207	170	215	181	186	162	152	182	181	179	190	172	171	177	177	160	199
19	Myosin-9 OS=Homo sapiens OX=9606 GN=MYH9 PE=1 SV=4	MYH9_HUMAN	MYH9	227 kDa	85	113	66	74	62	89	79	49	79	91	55	75	110	105	113	127	176	151	135	124	123	143	124	84	119	257	215	89	140	149	419	198	172	115	248	129	371	241	627	218	253	125	216	327	198	73	355
20	Peroxiredoxin-2 OS=Homo sapiens OX=9606 GN=PRDX2 PE=1 SV=5	PRDX2_HUMAN	PRDX2	22 kDa	292	161	132	103	199	207	267	133	158	153	86	96	126	137	77	89	158	189	173	144	213	188	144	120	48	202	304	222	64	127	97	102	178	164	149	152	135	303	173	116	141	140	282	190	105	102	159
21	Clathrin heavy chain 1 OS=Homo sapiens OX=9606 GN=CLTC PE=1 SV=5	CLH1_HUMAN	CLTC	192 kDa	176	182	101	115	103	143	121	98	95	109	131	162	115	128	135	132	229	241	155	127	200	214	243	141	61	112	207	147	55	94	179	117	214	172	142	227	194	287	275	96	112	176	289	182	129	128	180
22	Dematin OS=Homo sapiens OX=9606 GN=DMTN PE=1 SV=3	DEMA_HUMAN	DMTN	46 kDa	102	125	137	128	110	130	106	88	165	148	159	149	143	157	141	139	136	146	153	151	145	143	117	149	131	146	126	132	120	121	146	132	143	142	142	130	110	137	130	143	128	151	146	146	144	157	116
23	Transitional endoplasmic reticulum ATPase OS=Homo sapiens OX=9606 GN=VCP PE=1 SV=4	TERA_HUMAN	VCP	89 kDa	180	137	125	71	114	152	172	115	122	145	84	80	90	98	81	100	153	187	135	83	189	161	185	110	51	161	216	162	67	115	101	101	163	162	105	153	109	241	150	85	101	146	246	167	79	72	156
24	Tropomodulin-1 OS=Homo sapiens OX=9606 GN=TMOD1 PE=1 SV=1	TMOD1_HUMAN	TMOD1	41 kDa	108	118	136	114	120	122	114	124	124	122	137	142	124	122	138	130	124	123	122	136	128	135	131	120	120	128	126	128	111	125	137	117	122	137	128	123	112	122	131	137	128	133	135	127	134	131	125
25	Aquaporin-1 OS=Homo sapiens OX=9606 GN=AQP1 PE=1 SV=3	AQP1_HUMAN	AQP1	29 kDa	146	143	153	153	129	117	152	137	155	148	166	117	128	114	134	106	137	102	129	138	112	134	92	111	96	84	105	127	128	97	140	118	147	111	109	124	124	128	117	106	118	146	123	131	113	109	133
26	E3 ubiquitin-protein ligase UBR4 OS=Homo sapiens OX=9606 GN=UBR4 PE=1 SV=1	UBR4_HUMAN	UBR4	574 kDa	125	151	101	52	86	84	89	62	83	106	50	89	110	126	111	105	190	161	160	74	212	181	183	81	4	25	91	169	17	58	70	39	113	149	40	235	104	171	137	78	95	201	157	114	65	59	98
27	ATP-citrate synthase OS=Homo sapiens OX=9606 GN=ACLY PE=1 SV=3	ACLY_HUMAN	ACLY	121 kDa	118	94	76	63	72	79	93	69	103	95	78	167	122	185	134	121	135	137	110	144	149	131	148	89	68	132	142	91	70	76	87	64	109	83	81	130	136	186	160	126	147	118	199	152	131	161	115
28	Plasma membrane calcium-transporting ATPase 4 OS=Homo sapiens OX=9606 GN=ATP2B4 PE=1 SV=2	AT2B4_HUMAN	ATP2B4	138 kDa	102	101	123	117	104	107	110	106	119	98	107	105	112	126	127	105	107	100	111	104	119	97	98	96	96	115	105	111	77	111	120	99	96	90	112	76	98	89	120	122	92	113	92	116	95	97	109
29	Solute carrier family 2, facilitated glucose transporter member 1 OS=Homo sapiens OX=9606 GN=SLC2A1 PE=1 SV=2	GTR1_HUMAN	SLC2A1	54 kDa	105	105	106	102	102	97	108	98	118	117	142	114	129	112	129	107	101	98	107	120	102	103	112	100	83	115	117	111	98	97	112	114	103	99	107	103	100	99	108	100	94	121	118	114	103	121	86
30	Hemoglobin subunit beta OS=Homo sapiens OX=9606 GN=HBB PE=1 SV=2	HBB_HUMAN	HBB	16 kDa	128	93	76	91	129	118	110	114	118	109	96	117	107	122	71	89	86	132	133	99	143	104	105	128	76	160	125	120	73	76	93	97	77	67	89	71	81	117	108	85	80	87	112	127	113	143	100
31	Kell blood group glycoprotein OS=Homo sapiens OX=9606 GN=KEL PE=1 SV=2	KELL_HUMAN	KEL	83 kDa	87	92	96	82	81	90	80	81	86	84	90	98	101	111	111	82	98	81	90	108	91	95	87	81	78	86	76	87	81	87	85	61	74	81	94	78	72	90	88	108	87	96	75	89	83	105	93
32	Ras-related protein Rap-1A OS=Homo sapiens OX=9606 GN=RAP1A PE=1 SV=1	RAP1A_HUMAN	RAP1A	21 kDa	114	54	114	74	71	114	99	64	102	94	107	91	63	64	70	77	87	68	55	62	80	74	76	78	123	80	72	90	67	89	97	98	112	108	94	87	73	88	80	68	59	97	81	63	112	83	92
33	Immunoglobulin gamma-1 heavy chain OS=Homo sapiens OX=9606 PE=1 SV=2	IGG1_HUMAN		49 kDa	77	66	37	71	68	81	94	108	97	79	110	101	73	77	140	130	102	77	78	64	72	93	84	70	84	85	108	111	66	112	95	53	74	72	86	56	73	69	107	137	85	109	95	95	143	99	35
34	Heat shock cognate 71 kDa protein OS=Homo sapiens OX=9606 GN=HSPA8 PE=1 SV=1	HSP7C_HUMAN	HSPA8	71 kDa	86	60	51	43	54	63	68	48	61	57	61	111	101	90	67	94	77	101	75	76	105	113	101	47	67	89	133	81	50	63	58	49	84	66	67	106	106	171	112	107	108	91	160	134	100	81	103
35	T-complex protein 1 subunit eta OS=Homo sapiens OX=9606 GN=CCT7 PE=1 SV=2	TCPH_HUMAN	CCT7	59 kDa	94	75	55	31	49	77	60	39	60	50	25	38	49	66	47	42	73	105	66	46	80	93	91	37	31	66	136	68	34	51	54	44	93	84	46	100	67	167	114	70	74	60	146	104	56	56	116
36	AP-2 complex subunit beta OS=Homo sapiens OX=9606 GN=AP2B1 PE=1 SV=1	AP2B1_HUMAN	AP2B1	105 kDa	64	64	50	48	46	52	64	40	36	51	53	75	57	50	56	59	81	69	60	50	69	65	81	58	50	76	88	66	61	79	87	64	92	76	65	71	89	102	89	80	75	70	95	84	80	65	78
37	Catalase OS=Homo sapiens OX=9606 GN=CAT PE=1 SV=3	CATA_HUMAN	CAT	60 kDa	172	66	41	26	98	96	92	49	65	72	21	26	45	51	22	23	49	105	99	79	121	97	60	43	17	103	157	116	8	48	34	34	62	67	74	38	44	129	99	41	56	72	127	75	38	45	55
38	ATP-binding cassette sub-family B member 6, mitochondrial OS=Homo sapiens OX=9606 GN=ABCB6 PE=1 SV=1	ABCB6_HUMAN	ABCB6	94 kDa	80	63	89	62	59	65	74	51	72	61	62	64	71	76	76	64	71	59	70	78	85	65	75	57	53	84	65	77	61	66	61	51	66	55	70	65	68	61	73	79	68	84	70	80	60	67	56
39	Arginase-1 OS=Homo sapiens OX=9606 GN=ARG1 PE=1 SV=2	ARGI1_HUMAN	ARG1	35 kDa	62	59	54	45	66	56	64	59	58	62	48	40	48	39	54	57	55	50	56	44	44	51	45	64	65	74	69	91	95	86	84	101	90	110	86	93	78	69	57	85	69	97	45	62	53	51	87
40	AP-2 complex subunit alpha-1 OS=Homo sapiens OX=9606 GN=AP2A1 PE=1 SV=3	AP2A1_HUMAN	AP2A1	108 kDa	55	44	48	32	32	39	71	33	34	44	46	60	50	41	46	55	88	54	55	41	69	61	76	54	38	70	79	58	53	66	80	57	75	61	71	66	90	101	91	73	77	70	100	91	79	62	77
41	Keratin, type I cytoskeletal 9 OS=Homo sapiens OX=9606 GN=KRT9 PE=1 SV=3	K1C9_HUMAN	KRT9	62 kDa	55	62	54	52	88	127	71	41	57	101	65	66	74	66	41	86	60	62	58	47	36	43	62	97	51	88	67	52	35	69	70	52	49	36	63	74	63	48	60	43	57	60	72	68	46	66	39
42	Keratin, type II cytoskeletal 1 OS=Homo sapiens OX=9606 GN=KRT1 PE=1 SV=6	K2C1_HUMAN	KRT1	66 kDa	63	62	53	47	72	95	68	59	53	89	82	77	112	94	57	108	81	77	75	78	54	59	74	83	47	87	73	59	33	63	69	49	49	44	57	72	64	56	79	43	68	57	69	68	51	76	41
43	Keratin, type I cytoskeletal 10 OS=Homo sapiens OX=9606 GN=KRT10 PE=1 SV=6	K1C10_HUMAN	KRT10	59 kDa	60	61	51	53	76	51	48	64	51	90	79	68	100	76	53	76	85	62	63	55	35	49	74	92	50	69	83	53	39	41	61	39	41	33	40	41	49	58	69	33	67	46	50	64	49	79	32
44	Tropomyosin alpha-3 chain OS=Homo sapiens OX=9606 GN=TPM3 PE=1 SV=2	TPM3_HUMAN	TPM3	33 kDa	55	48	59	57	55	59	52	50	64	65	64	56	46	54	44	56	50	57	56	54	61	47	55	54	58	57	70	60	57	67	60	59	61	63	55	54	51	76	63	50	57	64	69	61	58	51	58
45	Piezo-type mechanosensitive ion channel component 1 OS=Homo sapiens OX=9606 GN=PIEZO1 PE=1 SV=4	PIEZ1_HUMAN	PIEZO1	287 kDa	44	54	50	41	48	42	48	44	61	56	54	60	76	88	68	71	70	53	58	49	61	62	59	61	50	58	48	50	42	52	59	47	48	44	31	50	32	50	62	61	55	59	42	50	66	65	44
46	Erythroid membrane-associated protein OS=Homo sapiens OX=9606 GN=ERMAP PE=1 SV=1	ERMAP_HUMAN	ERMAP	53 kDa	46	44	47	51	54	60	53	57	54	52	59	54	55	42	50	64	53	49	54	43	60	54	46	60	66	54	61	58	66	56	67	65	64	56	62	63	54	52	68	77	84	75	60	67	58	53	63
47	Ras-related protein Rap-2b OS=Homo sapiens OX=9606 GN=RAP2B PE=1 SV=1	RAP2B_HUMAN	RAP2B	21 kDa	66	74	83	75	66	65	80	73	64	61	75	59	47	50	55	60	60	36	44	53	55	60	51	55	63	42	40	79	52	58	44	51	59	63	52	57	53	42	54	54	58	74	30	42	51	41	56
48	Guanine nucleotide-binding protein G(I)/G(S)/G(T) subunit beta-1 OS=Homo sapiens OX=9606 GN=GNB1 PE=1 SV=3	GBB1_HUMAN	GNB1	37 kDa	77	77	84	41	50	74	63	71	56	39	65	54	47	51	48	47	56	47	55	44	54	59	51	60	61	48	49	58	45	59	71	54	47	67	51	49	44	40	71	53	65	59	42	47	65	40	81
49	Heat shock protein HSP 90-alpha OS=Homo sapiens OX=9606 GN=HSP90AA1 PE=1 SV=5	HS90A_HUMAN	HSP90AA1	85 kDa	62	35	20	11	40	51	41	30	28	42	12	25	29	37	23	18	41	65	53	28	67	46	62	27	12	94	177	42	29	52	47	39	100	33	45	41	53	235	100	38	59	24	167	87	25	14	77
50	Myosin-10 OS=Homo sapiens OX=9606 GN=MYH10 PE=1 SV=3	MYH10_HUMAN	MYH10	229 kDa	16	26	11	22	10	24	19	11	25	20	15	18	34	29	31	43	54	44	36	41	39	40	37	21	66	154	100	27	73	73	273	122	79	31	149	36	224	115	388	154	150	39	109	204	130	23	176
51	Guanine nucleotide-binding protein G(k) subunit alpha OS=Homo sapiens OX=9606 GN=GNAI3 PE=1 SV=3	GNAI3_HUMAN	GNAI3	41 kDa	42	44	44	42	51	72	48	41	52	36	41	69	26	53	61	63	56	70	69	51	54	62	32	45	65	55	55	67	44	47	68	51	45	58	46	50	37	47	71	64	59	60	49	56	59	58	70
52	Proteasome subunit alpha type-6 OS=Homo sapiens OX=9606 GN=PSMA6 PE=1 SV=1	PSA6_HUMAN	PSMA6	27 kDa	83	76	35	13	31	29	56	21	28	44	18	17	27	31	18	25	46	74	48	30	89	43	67	26	11	61	132	60	13	34	26	18	83	48	36	43	60	146	61	23	43	38	133	82	17	14	95
53	T-complex protein 1 subunit alpha OS=Homo sapiens OX=9606 GN=TCP1 PE=1 SV=1	TCPA_HUMAN	TCP1	60 kDa	83	41	22	12	21	53	32	21	26	34	7	29	41	41	36	41	60	74	49	23	69	79	79	20	5	39	105	36	18	36	45	30	91	62	29	90	44	167	95	39	65	33	127	81	33	25	105
54	Acylamino-acid-releasing enzyme OS=Homo sapiens OX=9606 GN=APEH PE=1 SV=4	ACPH_HUMAN	APEH	81 kDa	96	51	29	15	54	60	70	21	33	60	8	18	32	34	16	16	43	86	73	32	89	58	66	48	13	74	153	75	12	29	18	23	61	46	37	32	35	131	47	25	31	37	141	58	14	16	43
55	Proteasome subunit beta type-4 OS=Homo sapiens OX=9606 GN=PSMB4 PE=1 SV=4	PSB4_HUMAN	PSMB4	29 kDa	93	78	57	21	61	71	77	36	40	82	19	9	26	31	14	19	65	68	56	23	80	74	64	40	6	70	90	78	14	66	34	37	85	85	50	81	83	100	81	37	77	60	105	82	27	11	94
56	26S proteasome non-ATPase regulatory subunit 2 OS=Homo sapiens OX=9606 GN=PSMD2 PE=1 SV=3	PSMD2_HUMAN	PSMD2	100 kDa	58	62	36	18	32	45	44	21	37	51	23	21	39	44	28	25	75	72	56	24	83	69	81	46	9	86	100	64	17	39	40	37	76	63	42	82	53	127	61	24	44	53	109	77	23	20	65
57	Long-chain-fatty-acid--CoA ligase 4 OS=Homo sapiens OX=9606 GN=ACSL4 PE=1 SV=2	ACSL4_HUMAN	ACSL4	79 kDa	50	47	40	40	39	48	41	29	54	51	49	47	47	57	50	44	52	52	52	48	44	48	57	42	30	46	47	55	44	49	53	42	54	52	49	54	50	49	61	67	44	59	44	53	40	52	37
58	T-complex protein 1 subunit epsilon OS=Homo sapiens OX=9606 GN=CCT5 PE=1 SV=1	TCPE_HUMAN	CCT5	60 kDa	65	39	30	9	31	61	34	23	35	38	10	16	29	36	16	22	41	65	31	12	62	57	60	24	7	47	110	53	20	28	48	35	81	67	30	70	37	153	81	22	47	27	138	65	13	11	95
59	Endonuclease domain-containing 1 protein OS=Homo sapiens OX=9606 GN=ENDOD1 PE=1 SV=2	ENDD1_HUMAN	ENDOD1	55 kDa	49	34	55	42	37	45	44	33	46	42	50	44	41	41	43	36	41	41	43	47	48	30	47	46	42	42	46	44	40	45	47	43	54	41	50	45	48	53	50	42	52	47	46	44	41	57	47
60	2',3'-cyclic-nucleotide 3'-phosphodiesterase OS=Homo sapiens OX=9606 GN=CNP PE=1 SV=2	CN37_HUMAN	CNP	48 kDa	49	47	57	32	38	32	45	33	44	35	43	54	54	51	39	51	47	41	44	53	51	44	40	47	42	50	42	34	31	39	38	27	43	34	40	32	29	35	35	38	39	57	40	43	46	51	34
61	T-complex protein 1 subunit zeta OS=Homo sapiens OX=9606 GN=CCT6A PE=1 SV=3	TCPZ_HUMAN	CCT6A	58 kDa	66	49	32	11	38	55	31	31	29	29	11	21	28	33	21	20	51	68	39	26	55	64	60	16	7	33	81	48	15	34	49	26	68	57	28	54	31	116	67	35	49	27	88	71	24	21	82
62	Multidrug resistance-associated protein 4 OS=Homo sapiens OX=9606 GN=ABCC4 PE=1 SV=3	MRP4_HUMAN	ABCC4	150 kDa	40	46	58	36	39	50	43	37	42	53	16	22	55	57	57	43	54	57	57	59	52	48	46	50	37	46	46	28	35	37	51	52	43	42	52	32	38	38	60	49	50	32	27	40	42	60	47
63	Proteasome subunit beta type-5 OS=Homo sapiens OX=9606 GN=PSMB5 PE=1 SV=3	PSB5_HUMAN	PSMB5	28 kDa	66	50	45	25	48	40	60	43	34	49	18	22	29	31	25	27	55	53	48	22	65	53	60	43	15	66	81	54	28	48	42	44	57	56	49	57	57	94	62	27	46	49	95	68	32	13	78
64	Basal cell adhesion molecule OS=Homo sapiens OX=9606 GN=BCAM PE=1 SV=2	BCAM_HUMAN	BCAM	67 kDa	23	41	45	32	44	23	44	43	63	26	37	42	39	67	60	49	56	31	59	54	47	46	32	48	28	48	70	86	47	32	48	36	65	47	55	32	50	62	56	24	51	87	55	47	31	54	44
65	Blood group Rh(CE) polypeptide OS=Homo sapiens OX=9606 GN=RHCE PE=1 SV=2	RHCE_HUMAN	RHCE	46 kDa	43	50	41	50	26	57	46	31	49	55	51	42	46	39	37	40	35	37	37	42	31	40	27	38	42	42	41	43	33	56	46	27	49	44	40	48	30	50	42	57	42	42	34	46	52	42	42
66	T-complex protein 1 subunit delta OS=Homo sapiens OX=9606 GN=CCT4 PE=1 SV=4	TCPD_HUMAN	CCT4	58 kDa	53	42	28	16	29	46	35	22	32	28	18	29	37	46	37	38	46	60	39	27	45	58	67	29	19	38	70	35	19	35	45	27	61	52	32	62	44	91	76	43	57	34	86	58	34	34	67
67	Golgi-associated plant pathogenesis-related protein 1 OS=Homo sapiens OX=9606 GN=GLIPR2 PE=1 SV=3	GAPR1_HUMAN	GLIPR2	17 kDa	55	50	63	38	41	40	43	37	52	40	45	47	44	54	44	38	41	31	42	43	45	40	34	39	40	34	40	53	41	40	41	37	44	42	44	40	44	36	43	42	31	45	38	38	39	41	40
68	Sorbitol dehydrogenase OS=Homo sapiens OX=9606 GN=SORD PE=1 SV=4	DHSO_HUMAN	SORD	38 kDa	50	65	57	41	57	40	62	59	34	28	35	23	42	34	54	38	53	29	45	34	33	50	40	33	48	47	24	42	51	51	51	42	40	51	45	42	34	31	36	51	54	49	18	44	46	35	47
69	Immunoglobulin kappa light chain OS=Homo sapiens OX=9606 PE=1 SV=1	IGK_HUMAN		23 kDa	50	43	20	39	46	36	97	88	47	39	72	61	28	41	97	65	46	43	34	35	19	32	34	30	41	35	44	68	21	77	33	24	29	26	48	18	40	22	52	92	34	57	35	59	96	59	18
70	Delta-aminolevulinic acid dehydratase OS=Homo sapiens OX=9606 GN=ALAD PE=1 SV=1	HEM2_HUMAN	ALAD	36 kDa	71	43	32	16	53	54	45	18	36	43	15	14	24	33	12	23	40	59	53	33	66	58	49	24	10	56	111	54	9	22	18	17	54	50	37	32	26	92	55	25	37	35	96	42	20	13	42
71	26S proteasome non-ATPase regulatory subunit 1 OS=Homo sapiens OX=9606 GN=PSMD1 PE=1 SV=2	PSMD1_HUMAN	PSMD1	106 kDa	48	49	39	24	31	35	43	25	26	44	11	12	29	23	16	19	67	47	37	15	59	51	63	22	5	44	72	43	22	37	34	37	66	55	33	68	50	107	51	26	44	46	86	49	23	7	57
72	Basigin OS=Homo sapiens OX=9606 GN=BSG PE=1 SV=2	BASI_HUMAN	BSG	42 kDa	37	34	39	37	43	35	32	41	51	40	34	40	41	49	55	39	41	41	45	42	37	36	44	35	44	53	44	38	52	47	50	58	50	43	43	44	48	43	45	65	69	43	43	51	35	39	54
73	Proteasome subunit alpha type-2 OS=Homo sapiens OX=9606 GN=PSMA2 PE=1 SV=2	PSA2_HUMAN	PSMA2	26 kDa	50	49	40	21	40	42	42	32	32	44	19	15	24	20	24	25	40	39	32	16	56	44	52	28	19	48	74	49	23	42	29	34	56	56	46	38	48	89	52	27	42	39	81	52	28	14	63
74	E2 ubiquitin-conjugating enzyme OS=Homo sapiens OX=9606 GN=UBE2O PE=1 SV=3	sp|Q9C0C9|UBE2O_HUMAN	UBE2O	141 kDa	56	38	18	15	26	33	30	21	31	31	10	26	30	33	29	32	63	55	48	25	55	55	63	18	3	41	77	57	11	30	37	25	41	54	42	72	55	104	67	35	50	60	83	58	18	11	59
75	Cullin-associated NEDD8-dissociated protein 1 OS=Homo sapiens OX=9606 GN=CAND1 PE=1 SV=2	CAND1_HUMAN	CAND1	136 kDa	62	42	14	10	45	42	48	19	31	37	17	28	33	42	29	24	44	50	48	29	73	59	49	18	15	40	76	52	11	27	30	20	49	33	22	34	42	97	53	36	36	36	78	55	31	38	53
76	Serum albumin OS=Homo sapiens OX=9606 GN=ALB PE=1 SV=2	ALBU_HUMAN	ALB	69 kDa	43	29	29	14	33	44	40	27	28	34	35	46	43	40	41	56	52	48	47	29	53	39	46	33	41	55	41	41	32	29	31	38	40	17	34	30	42	41	39	43	36	36	60	54	41	41	26
77	T-complex protein 1 subunit beta OS=Homo sapiens OX=9606 GN=CCT2 PE=1 SV=4	TCPB_HUMAN	CCT2	57 kDa	70	33	24	3	25	48	25	17	25	44	2	13	11	28	8	6	29	50	26	2	47	39	57	15		31	143	50	7	24	29	21	83	64	17	49	29	206	58	9	24	16	173	36	7	9	94
78	Proteasome activator complex subunit 2 OS=Homo sapiens OX=9606 GN=PSME2 PE=1 SV=4	PSME2_HUMAN	PSME2	27 kDa	68	46	36	29	39	40	48	29	39	43	20	18	32	40	23	25	38	55	50	34	62	44	50	33	18	41	81	46	20	26	24	33	56	36	26	49	51	98	50	27	35	44	95	53	30	15	67
79	Heat shock 70 kDa protein 1A OS=Homo sapiens OX=9606 GN=HSPA1A PE=1 SV=1	HS71A_HUMAN (+1)	HSPA1A	70 kDa	27	15	6	10	13	19	27	19	13	14	21	60	42	42	41	35	41	49	31	30	29	42	41	10	19	52	79	24	18	26	51	20	41	14	30	30	50	134	150	80	81	32	119	98	54	36	55
80	cAMP-dependent protein kinase type I-alpha regulatory subunit OS=Homo sapiens OX=9606 GN=PRKAR1A PE=1 SV=1	KAP0_HUMAN	PRKAR1A	43 kDa	35	25	26	24	20	30	25	26	42	35	23	31	55	49	68	40	48	52	45	50	45	33	50	65	35	55	43	49	37	46	45	40	38	33	35	33	33	41	51	45	44	47	44	51	51	51	33
81	Proteasome subunit beta type-3 OS=Homo sapiens OX=9606 GN=PSMB3 PE=1 SV=2	PSB3_HUMAN	PSMB3	23 kDa	58	47	32	21	40	31	55	26	27	42	14	11	26	19	18	25	44	43	37	17	54	48	48	27	5	46	68	51	17	34	32	29	56	50	37	54	56	89	50	21	52	45	75	54	23	15	63
82	Proteasome subunit alpha type-7 OS=Homo sapiens OX=9606 GN=PSMA7 PE=1 SV=1	PSA7_HUMAN	PSMA7	28 kDa	50	39	24	20	36	40	45	25	27	54	19	20	29	26	20	21	45	48	33	26	49	46	49	40	9	53	67	51	20	35	35	32	58	50	38	49	56	79	50	26	38	38	87	57	18	17	57
83	Protein disulfide-isomerase OS=Homo sapiens OX=9606 GN=P4HB PE=1 SV=3	PDIA1_HUMAN	P4HB	57 kDa	48	36	46	45	22	33	53	60	40	42	52	57	58	46	54	43	60	35	40	59	41	29	53	39	25	39	24	46	23	24	33	29	24	35	28	33	26	25	31	35	23	49	25	33	30	40	25
84	C-1-tetrahydrofolate synthase, cytoplasmic OS=Homo sapiens OX=9606 GN=MTHFD1 PE=1 SV=3	C1TC_HUMAN	MTHFD1	102 kDa	46	77	23	37	35	29	36	61	57	40	51	15	32	34	12	4	13	15	22	15	2	30	3	65	75	64	44	62	78	43	86	110	30	38	73	2	21	8	48	17	30	3	12	27	22	16	59
85	Glycophorin-A OS=Homo sapiens OX=9606 GN=GYPA PE=1 SV=2	GLPA_HUMAN	GYPA	16 kDa	29	24	27	23	20	21	27	23	32	30	27	31	39	30	32	26	27	28	29	31	21	26	28	33	22	27	25	21	24	28	27	25	38	31	34	24	32	24	19	18	24	27	28	28	34	27	21
86	Phosphatidylinositol 5-phosphate 4-kinase type-2 alpha OS=Homo sapiens OX=9606 GN=PIP4K2A PE=1 SV=2	PI42A_HUMAN	PIP4K2A	46 kDa	50	43	58	26	31	44	38	27	35	51	35	37	32	37	33	31	36	38	39	33	62	43	43	20	27	31	53	43	28	39	39	27	49	40	27	35	32	66	31	31	32	39	52	39	37	28	28
87	Ras-related protein Rab-10 OS=Homo sapiens OX=9606 GN=RAB10 PE=1 SV=1	RAB10_HUMAN	RAB10	23 kDa	36	39	44	24	24	39	41	33	26	24	22	27	22	20	27	25	26	25	25	21	31	37	32	31	73	26	34	29	36	31	44	48	44	50	29	59	32	33	44	47	45	33	29	32	77	32	49
88	T-complex protein 1 subunit gamma OS=Homo sapiens OX=9606 GN=CCT3 PE=1 SV=4	TCPG_HUMAN	CCT3	61 kDa	54	27	25	7	25	36	23	18	22	35	6	20	25	34	19	16	40	47	34	16	48	50	53	14	2	40	75	31	16	34	31	22	67	59	22	44	28	99	52	14	33	24	83	48	22	18	58
89	Guanine nucleotide-binding protein G(s) subunit alpha isoforms short OS=Homo sapiens OX=9606 GN=GNAS PE=1 SV=1	GNAS2_HUMAN	GNAS	46 kDa	39	30	37	37	33	48	34	42	54	44	44	42	29	35	37	38	35	45	31	37	36	35	40	32	43	46	51	48	42	41	46	49	44	41	41	42	35	44	42	45	48	46	46	42	48	36	46
90	GTPase KRas OS=Homo sapiens OX=9606 GN=KRAS PE=1 SV=1	RASK_HUMAN	KRAS	22 kDa	33	36	45	33	26	46	37	39	40	36	29	35	30	48	32	29	35	33	34	42	37	29	28	40	35	34	39	40	34	38	48	28	32	40	38	26	26	28	39	37	41	35	31	32	39	31	35
91	Proteasome subunit alpha type-5 OS=Homo sapiens OX=9606 GN=PSMA5 PE=1 SV=3	PSA5_HUMAN	PSMA5	26 kDa	49	43	21	13	27	28	28	22	20	25	16	14	33	27	13	19	30	35	35	21	44	30	39	24	12	34	94	32	13	25	19	24	43	38	23	29	34	138	34	25	27	21	103	39	21	8	82
92	Polypyrimidine tract-binding protein 1 OS=Homo sapiens OX=9606 GN=PTBP1 PE=1 SV=1	PTBP1_HUMAN	PTBP1	57 kDa	48	41	40	29	32	37	44	49	26	28	34	32	32	38	32	34	33	43	34	39	40	37	42	37	26	34	31	34	24	28	37	25	44	30	37	27	31	37	51	30	34	34	35	36	32	34	40
93	Acetylcholinesterase OS=Homo sapiens OX=9606 GN=ACHE PE=1 SV=1	ACES_HUMAN	ACHE	68 kDa	31	30	31	37	29	37	30	28	35	36	41	36	34	35	26	30	29	39	34	29	37	27	33	33	26	23	24	29	26	25	30	26	32	28	31	30	26	28	26	32	39	39	21	20	21	39	31
94	Proteasome activator complex subunit 1 OS=Homo sapiens OX=9606 GN=PSME1 PE=1 SV=1	PSME1_HUMAN	PSME1	29 kDa	39	30	28	14	26	29	35	32	31	36	12	11	22	18	13	11	26	34	31	12	39	31	36	20	9	33	53	41	17	19	20	15	36	26	18	31	22	56	29	15	24	26	53	36	15	15	38
95	Long-chain-fatty-acid--CoA ligase 6 OS=Homo sapiens OX=9606 GN=ACSL6 PE=2 SV=4	ACSL6_HUMAN	ACSL6	78 kDa	36	29	38	25	27	35	34	31	41	26	35	36	35	33	35	32	37	31	31	41	34	30	33	38	28	31	31	46	19	25	29	27	35	24	33	21	30	29	29	22	24	45	26	26	26	45	30
96	Proteasome subunit beta type-1 OS=Homo sapiens OX=9606 GN=PSMB1 PE=1 SV=2	PSB1_HUMAN	PSMB1	26 kDa	34	29	27	20	29	35	31	25	28	36	17	13	28	23	19	19	38	43	36	23	48	43	40	26	14	41	55	37	19	24	27	25	41	33	29	38	36	55	35	26	41	39	62	46	22	18	38
97	26S proteasome regulatory subunit 7 OS=Homo sapiens OX=9606 GN=PSMC2 PE=1 SV=3	PRS7_HUMAN	PSMC2	49 kDa	38	37	20	5	20	18	41	13	18	37	4	12	28	29	13	18	46	26	32	15	38	41	35	17	3	38	56	50	9	28	22	20	44	46	22	40	29	78	41	19	22	26	72	42	18	7	36
98	cAMP-dependent protein kinase catalytic subunit alpha OS=Homo sapiens OX=9606 GN=PRKACA PE=1 SV=2	KAPCA_HUMAN	PRKACA	41 kDa	37	13	27	26	16	38	23	35	36	36	10	13	22	28	37	23	25	37	14	34	27	23	24	33	38	45	31	38	41	44	38	49	33	31	24	27	27	26	39	43	39	46	27	46	32	30	50
99	Proteasome subunit alpha type-1 OS=Homo sapiens OX=9606 GN=PSMA1 PE=1 SV=1	PSA1_HUMAN	PSMA1	30 kDa	29	27	19	13	25	23	23	17	21	32	17	17	26	22	21	22	42	42	36	23	45	35	42	22	19	33	51	30	16	26	28	25	33	36	32	42	33	62	34	29	33	35	57	47	25	14	37
100	Aldehyde dehydrogenase family 16 member A1 OS=Homo sapiens OX=9606 GN=ALDH16A1 PE=1 SV=2	A16A1_HUMAN	ALDH16A1	85 kDa	32	23	20	17	19	24	29	15	27	30	16	17	29	31	17	26	31	29	28	26	38	35	39	16	13	46	44	30	22	29	27	21	39	39	32	35	41	45	47	43	38	42	47	63	22	23	32
101	Elongation factor 1-alpha 1 OS=Homo sapiens OX=9606 GN=EEF1A1 PE=1 SV=1	EF1A1_HUMAN	EEF1A1	50 kDa	28	17	25	24	22	18	16	20	44	31	26	35	35	41	33	27	34	21	35	17	34	33	30	37	33	27	37	17	35	22	40	42	42	34	16	28	16	47	45	27	40	19	40	32	35	26	51
102	E3 ubiquitin-protein ligase HUWE1 OS=Homo sapiens OX=9606 GN=HUWE1 PE=1 SV=3	HUWE1_HUMAN	HUWE1	482 kDa	41	45	24	2	24	25	21	22	8	19	4	7	10	11	9	14	46	47	36	7	55	52	65	8		7	67	27	2	11	16	9	48	35	7	57	20	110	40	5	14	33	94	28	4	2	32
103	Glutathione S-transferase LANCL1 OS=Homo sapiens OX=9606 GN=LANCL1 PE=1 SV=1	LANC1_HUMAN	LANCL1	45 kDa	11	13	14	11	9	19	13	16	26	17	23	39	29	41	37	44	36	39	31	37	44	35	37	31	35	44	37	33	19	22	32	25	21	22	21	40	43	46	48	49	41	45	43	43	48	40	49
104	NADH-cytochrome b5 reductase 3 OS=Homo sapiens OX=9606 GN=CYB5R3 PE=1 SV=3	NB5R3_HUMAN	CYB5R3	34 kDa	28	29	32	19	21	29	34	28	30	28	39	45	34	38	38	31	38	23	28	32	31	30	32	29	25	31	27	38	14	22	24	15	25	25	27	26	21	26	24	28	20	39	27	30	36	40	27
105	Deoxyribose-phosphate aldolase OS=Homo sapiens OX=9606 GN=DERA PE=1 SV=2	DEOC_HUMAN	DERA	35 kDa	26	17	18	9	19	18	24	13	28	29	16	15	20	35	13	26	26	29	27	20	33	24	39	17	13	40	37	32	18	29	29	18	31	29	31	32	35	46	44	49	40	38	48	60	16	20	31
106	Nucleosome assembly protein 1-like 4 OS=Homo sapiens OX=9606 GN=NAP1L4 PE=1 SV=1	NP1L4_HUMAN	NAP1L4	43 kDa	32	26	21	17	26	28	33	23	33	34	23	15	27	20	21	28	36	32	30	23	34	34	40	23	17	43	56	34	24	33	41	36	46	42	48	34	47	55	48	30	40	42	53	44	26	15	50
107	26S proteasome regulatory subunit 6A OS=Homo sapiens OX=9606 GN=PSMC3 PE=1 SV=3	PRS6A_HUMAN	PSMC3	49 kDa	34	43	28	11	17	29	31	23	17	31	11	11	19	16	8	13	34	27	22	9	43	28	37	22	7	38	53	39	14	29	33	25	46	43	28	48	32	57	39	10	33	31	48	41	15	6	43
108	Multidrug resistance-associated protein 1 OS=Homo sapiens OX=9606 GN=ABCC1 PE=1 SV=3	MRP1_HUMAN	ABCC1	172 kDa	18	30	21	16	31	27	20	18	34	23	34	38	24	36	25	32	33	31	39	23	18	28	25	32	41	31	21	15	22	24	32	26	20	24	23	29	21	19	35	34	30	26	21	31	38	34	30
109	UDP-glucose:glycoprotein glucosyltransferase 1 OS=Homo sapiens OX=9606 GN=UGGT1 PE=1 SV=3	UGGG1_HUMAN	UGGT1	177 kDa	29	22	40	27	18	22	39	38	34	30	39	63	43	48	55	27	52	21	18	38	43	21	46	38	16	24	13	32	6	10	13	12	18	17	16	24	10	12	13	11	9	39	11	20	10	50	17
110	T-complex protein 1 subunit theta OS=Homo sapiens OX=9606 GN=CCT8 PE=1 SV=4	TCPQ_HUMAN	CCT8	60 kDa	44	29	16	8	20	32	16	14	24	31	6	13	13	21	6	10	27	45	24	6	47	45	54	12	4	31	66	40	11	24	27	21	54	41	20	42	28	88	47	15	26	17	73	38	11	6	44
111	Calreticulin OS=Homo sapiens OX=9606 GN=CALR PE=1 SV=1	CALR_HUMAN	CALR	48 kDa	24	16	21	15	13	15	19	32	30	35	37	48	38	33	48	40	37	24	29	34	40	21	41	30	21	20	19	35	3	15	17	14	15	18	20	29	16	19	24	24	13	37	12	16	23	28	12
112	Dynamin-2 OS=Homo sapiens OX=9606 GN=DNM2 PE=1 SV=2	DYN2_HUMAN	DNM2	98 kDa	12	10	10	13	14	12	10	14	24	16	17	45	35	44	45	28	40	39	42	38	42	49	36	21	10	13	35	22	9	11	28	10	21	13	5	28	16	37	45	27	27	27	47	34	17	56	32
113	Probable ubiquitin carboxyl-terminal hydrolase FAF-X OS=Homo sapiens OX=9606 GN=USP9X PE=1 SV=3	USP9X_HUMAN	USP9X	292 kDa	38	22	15	4	20	19	18	11	12	23	3	3	6	9	8	9	29	32	30	4	46	36	42	8	0	28	63	41	6	24	22	15	49	34	19	32	28	96	41	7	22	21	90	39	11	1	50
114	Equilibrative nucleoside transporter 1 OS=Homo sapiens OX=9606 GN=SLC29A1 PE=1 SV=3	S29A1_HUMAN	SLC29A1	50 kDa	22	28	30	29	22	25	30	26	27	32	28	33	33	30	27	28	28	24	37	30	28	18	28	27	20	26	20	29	29	18	22	23	21	27	21	19	27	23	18	18	20	26	16	21	22	27	27
115	Proteasome subunit beta type-7 OS=Homo sapiens OX=9606 GN=PSMB7 PE=1 SV=1	PSB7_HUMAN	PSMB7	30 kDa	36	29	17	4	19	17	24	3	8	18	7	8	16	16	11	12	32	33	25	12	46	37	39	15	9	27	60	26	5	15	9	9	33	14	15	9	22	75	28	12	26	20	75	43	12	8	26
116	26S proteasome non-ATPase regulatory subunit 3 OS=Homo sapiens OX=9606 GN=PSMD3 PE=1 SV=2	PSMD3_HUMAN	PSMD3	61 kDa	38	33	32	13	25	33	35	24	23	30	17	13	19	18	8	14	40	30	25	9	39	31	36	14	8	36	39	32	14	24	23	20	39	43	23	37	25	59	32	13	22	26	42	35	16	8	36
117	Endoplasmic reticulum chaperone BiP OS=Homo sapiens OX=9606 GN=HSPA5 PE=1 SV=2	BIP_HUMAN	HSPA5	72 kDa	34	31	32	34	24	23	32	34	34	30	45	51	39	48	48	30	43	26	31	43	45	25	42	33	21	29	32	38	10	16	23	19	25	29	23	28	17	27	23	20	21	42	26	27	22	38	26
118	Ammonium transporter Rh type A OS=Homo sapiens OX=9606 GN=RHAG PE=1 SV=2	RHAG_HUMAN	RHAG	44 kDa	26	23	26	28	30	28	32	27	31	28	29	29	30	31	26	27	30	28	27	31	27	32	25	29	26	29	23	28	29	30	28	29	27	29	28	27	29	23	22	25	28	24	22	22	26	29	26
119	ATP-dependent 6-phosphofructokinase, liver type OS=Homo sapiens OX=9606 GN=PFKL PE=1 SV=6	PFKAL_HUMAN	PFKL	85 kDa	37	21	14	10	32	46	30	22	18	24	5	22	23	36	40	31	35	46	42	29	43	39	37	9	1	27	46	22	11	15	19	21	24	16	8	36	23	56	47	30	28	25	49	29	25	31	49
120	Semaphorin-7A OS=Homo sapiens OX=9606 GN=SEMA7A PE=1 SV=1	SEM7A_HUMAN	SEMA7A	75 kDa	19	21	27	26	21	28	21	20	30	17	22	44	24	40	39	36	36	35	33	33	31	28	27	39	22	19	26	31	16	11	29	16	14	16	29	16	15	17	33	11	29	34	22	13	20	34	19
121	GTPase-activating protein and VPS9 domain-containing protein 1 OS=Homo sapiens OX=9606 GN=GAPVD1 PE=1 SV=2	GAPD1_HUMAN	GAPVD1	165 kDa	36	25	22	8	22	15	17	12	15	25	4	9	14	19	18	15	32	28	25	8	48	27	42	10	2	17	46	25	6	18	20	10	40	25	16	35	37	70	43	19	23	27	68	39	11	1	32
122	Ribose-phosphate pyrophosphokinase 1 OS=Homo sapiens OX=9606 GN=PRPS1 PE=1 SV=2	PRPS1_HUMAN	PRPS1	35 kDa	21	16	6	3	20	25	25	15	22	25	3	22	19	31	33	27	31	47	38	35	39	36	36	17		24	50	16	1	7	12	2	21	13	2	30	19	67	40	20	26	17	66	39	21	49	41
123	Flavin reductase (NADPH) OS=Homo sapiens OX=9606 GN=BLVRB PE=1 SV=3	BLVRB_HUMAN	BLVRB	22 kDa	11	15	18	21	18	17	19	17	35	22	32	33	24	35	34	26	31	34	30	28	39	16	26	30	22	36	40	36	19	30	25	30	19	21	29	24	20	38	25	34	33	31	30	27	24	36	40
124	Calpain-5 OS=Homo sapiens OX=9606 GN=CAPN5 PE=1 SV=2	CAN5_HUMAN	CAPN5	73 kDa	24	25	31	32	39	29	27	30	27	27	23	27	24	26	36	26	27	30	33	36	21	21	19	23	28	15	18	27	19	24	20	13	16	16	24	16	25	15	12	26	17	26	10	12	17	25	14
125	Ezrin OS=Homo sapiens OX=9606 GN=EZR PE=1 SV=4	EZRI_HUMAN	EZR	69 kDa	19	21	20	10	17	17	22	18	24	24	21	24	20	14	16	28	22	21	24	13	33	22	23	21	19	29	33	30	16	24	21	18	25	21	17	14	24	31	24	20	20	20	28	27	27	22	24
126	Proteasome subunit alpha type-4 OS=Homo sapiens OX=9606 GN=PSMA4 PE=1 SV=1	PSA4_HUMAN	PSMA4	29 kDa	32	32	25	16	22	23	33	15	21	32	19	11	21	21	16	20	34	32	36	19	35	31	30	26	14	42	46	21	19	21	20	18	28	23	32	34	33	46	31	20	25	24	54	39	16	16	27
127	Immunoglobulin heavy constant gamma 2 OS=Homo sapiens OX=9606 GN=IGHG2 PE=1 SV=2	IGHG2_HUMAN	IGHG2	36 kDa	24	21	12	19	18	24	47	41	38	14	47	52	20	34	83	47	49	33	26	33	25	36	42	36	46	34	47	55	22	93	31	16	22	22	37	15	41	23	40	103	45	38	37	56	85	67	15
128	Neuropathy target esterase OS=Homo sapiens OX=9606 GN=PNPLA6 PE=1 SV=3	PLPL6_HUMAN	PNPLA6	151 kDa	27	18	46	21	16	18	34	31	29	27	39	38	31	33	36	20	36	31	25	24	31	14	32	29	21	21	18	45	13	12	2	10	23	31	11	42	13	22	1	15	15	52	20	17	19	29	9
129	Cell division control protein 42 homolog OS=Homo sapiens OX=9606 GN=CDC42 PE=1 SV=2	CDC42_HUMAN	CDC42	21 kDa	30	28	28	28	27	33	32	29	26	29	25	28	30	27	30	26	23	29	27	30	28	27	19	23	33	30	28	30	26	29	25	25	35	33	24	27	21	25	28	28	30	33	29	29	30	29	32
130	Atlastin-3 OS=Homo sapiens OX=9606 GN=ATL3 PE=1 SV=1	ATLA3_HUMAN	ATL3	61 kDa	16	22	23	20	16	17	24	21	19	24	23	29	33	30	27	28	25	25	22	31	26	24	26	34	29	19	16	27	11	22	21	15	18	15	26	29	17	20	16	22	14	32	16	22	24	35	22
131	Disheveled-associated activator of morphogenesis 1 OS=Homo sapiens OX=9606 GN=DAAM1 PE=1 SV=2	DAAM1_HUMAN	DAAM1	123 kDa	24	21	20	21	12	13	17	16	19	21	25	29	21	21	23	21	27	24	20	27	27	24	33	27	17	23	21	27	28	15	20	30	27	27	22	23	18	20	25	21	23	28	20	19	29	21	28
132	Importin-7 OS=Homo sapiens OX=9606 GN=IPO7 PE=1 SV=1	IPO7_HUMAN	IPO7	120 kDa	32	35	18	17	29	35	23	21	30	33	19	8	14	19	22	20	25	31	30	9	29	24	26	22	7	19	35	28	19	16	27	26	36	29	21	17	13	54	24	6	16	26	46	27	8	8	26
133	Neutral alpha-glucosidase AB OS=Homo sapiens OX=9606 GN=GANAB PE=1 SV=3	GANAB_HUMAN	GANAB	107 kDa	31	14	22	24	6	14	33	29	28	22	37	43	37	35	56	33	41	18	14	34	39	20	36	28	4	19	11	31	3	4	4	6	8	6	7	11	6	8	12	7	5	42	7	12	11	32	4
134	Hemoglobin subunit delta OS=Homo sapiens OX=9606 GN=HBD PE=1 SV=2	HBD_HUMAN	HBD	16 kDa	62	48	44	52	61	63	59	68	59	48	42	61	58	69	46	50	51	75	65	65	72	51	63	70	44	97	73	69	40	46	55	54	53	43	53	40	44	58	67	56	50	52	69	85	71	89	70
135	Proteasome subunit beta type-2 OS=Homo sapiens OX=9606 GN=PSMB2 PE=1 SV=1	PSB2_HUMAN	PSMB2	23 kDa	23	22	21	9	20	22	28	16	17	26	7	10	16	20	13	16	28	30	30	18	32	29	36	23	5	23	46	22	12	17	15	18	26	24	16	23	29	47	23	16	19	24	63	33	14	8	28
136	Actin, alpha skeletal muscle OS=Homo sapiens OX=9606 GN=ACTA1 PE=1 SV=1	ACTS_HUMAN	ACTA1	42 kDa	73	76	95	68	66	74	73	66	68	71	82	80	67	74	80	75	77	74	69	73	77	83	64	62	72	79	73	80	68	63	79	76	85	85	86	67	68	67	67	73	78	87	81	77	78	78	84
137	26S proteasome regulatory subunit 8 OS=Homo sapiens OX=9606 GN=PSMC5 PE=1 SV=1	PRS8_HUMAN	PSMC5	46 kDa	29	25	17	8	14	18	21	10	15	30	6	8	19	16	11	9	37	24	27	9	44	33	30	16	3	29	45	36	6	17	18	11	38	39	17	32	23	62	39	13	19	30	53	30	14	6	33
138	26S proteasome non-ATPase regulatory subunit 13 OS=Homo sapiens OX=9606 GN=PSMD13 PE=1 SV=2	PSD13_HUMAN	PSMD13	43 kDa	38	30	26	9	20	20	31	17	9	28	4	5	22	19	8	13	31	26	21	5	42	31	32	11	3	28	38	32	13	21	24	21	37	35	17	36	27	47	36	16	18	23	47	36	17	6	33
139	Monocarboxylate transporter 1 OS=Homo sapiens OX=9606 GN=SLC16A1 PE=1 SV=3	MOT1_HUMAN	SLC16A1	54 kDa	20	10	20	11	15	10	17	16	22	22	14	15	22	18	18	15	14	13	16	11	19	9	16	13	26	29	25	11	31	21	25	28	23	18	25	21	24	20	20	25	28	21	18	31	19	17	23
140	Protein disulfide-isomerase A3 OS=Homo sapiens OX=9606 GN=PDIA3 PE=1 SV=4	PDIA3_HUMAN	PDIA3	57 kDa	17	15	20	20	15	21	18	22	25	19	29	46	41	44	40	34	46	28	27	43	36	27	35	35	15	21	19	32	8	12	17	16	16	22	16	15	15	14	20	13	12	30	18	26	27	30	15
141	Ras-related protein Rab-35 OS=Homo sapiens OX=9606 GN=RAB35 PE=1 SV=1	RAB35_HUMAN	RAB35	23 kDa	48	21	50	57	41	39	40	33	42	28	24	29	23	27	29	22	25	27	24	39	30	30	26	31	30	26	35	29	22	26	30	26	37	33	23	30	17	29	29	30	32	36	28	24	32	34	28
142	ATP-dependent 6-phosphofructokinase, muscle type OS=Homo sapiens OX=9606 GN=PFKM PE=1 SV=2	PFKAM_HUMAN	PFKM	85 kDa	19	13	10	5	14	22	14	16	16	21	10	27	21	33	31	29	31	35	33	25	32	29	35	15	5	16	32	21	11	17	12	13	23	15	10	29	26	47	43	35	39	32	42	33	32	30	35
143	Proteasome subunit alpha type-3 OS=Homo sapiens OX=9606 GN=PSMA3 PE=1 SV=2	PSA3_HUMAN	PSMA3	28 kDa	38	30	23	15	22	28	33	16	18	27	15	9	22	16	9	12	23	32	23	12	29	25	29	24	8	30	39	24	16	22	17	14	32	28	26	31	30	51	32	17	22	26	46	26	14	8	33
144	AP-2 complex subunit mu OS=Homo sapiens OX=9606 GN=AP2M1 PE=1 SV=2	AP2M1_HUMAN	AP2M1	50 kDa	23	22	13	10	14	12	21	10	9	17	22	21	17	14	15	14	29	19	13	16	25	19	31	19	19	23	30	20	16	31	31	21	29	30	28	23	29	37	26	32	28	29	36	32	28	18	29
145	Sodium/potassium-transporting ATPase subunit alpha-1 OS=Homo sapiens OX=9606 GN=ATP1A1 PE=1 SV=1	AT1A1_HUMAN	ATP1A1	113 kDa	20	16	20	20	19	19	14	19	16	17	14	20	22	27	27	17	21	14	35	28	23	18	22	17	20	16	32	20	31	19	28	21	25	14	20	13	20	27	30	19	37	16	33	24	22	20	26
146	26S proteasome regulatory subunit 6B OS=Homo sapiens OX=9606 GN=PSMC4 PE=1 SV=2	PRS6B_HUMAN	PSMC4	47 kDa	26	29	22	8	10	15	19	8	8	15	7	5	19	13	9	8	35	28	20	8	34	28	34	17	2	21	35	22	5	16	22	14	30	24	18	36	17	56	26	7	19	23	54	23	12	4	29
147	Ras-related protein Rab-8A OS=Homo sapiens OX=9606 GN=RAB8A PE=1 SV=1	RAB8A_HUMAN	RAB8A	24 kDa	32	29	44	26	20	36	37	42	33	26	25	28	23	25	32	25	24	19	17	22	23	26	23	31	35	21	31	39	34	33	43	39	38	47	31	40	31	32	33	36	35	32	28	29	38	32	43
148	Tripeptidyl-peptidase 2 OS=Homo sapiens OX=9606 GN=TPP2 PE=1 SV=4	TPP2_HUMAN	TPP2	138 kDa	39	22	10	2	13	15	24	2	11	15	1	8	18	17	3	9	23	50	25	7	49	34	42	15	1	48	62	30	3	7	3	4	24	13	6	19	13	64	36	4	9	10	57	21	3	3	20
149	Dynactin subunit 1 OS=Homo sapiens OX=9606 GN=DCTN1 PE=1 SV=3	DCTN1_HUMAN	DCTN1	142 kDa	25	43	9	7	10	17	16	15	15	48	4	2	28	17	15	17	40	42	32	19	26	43	28	14		4	12	33	1	3	19	2	11	16	26	17	46	35	47	4	14	43	16	15	6	11	10
150	Calnexin OS=Homo sapiens OX=9606 GN=CANX PE=1 SV=2	CALX_HUMAN	CANX	68 kDa	25	19	26	29	16	17	28	17	21	23	38	37	33	25	26	23	30	20	20	30	24	22	18	25	13	11	10	30	5	6	13	14	12	11	9	17	4	15	12	8	6	30	9	9	10	28	13
151	Complement receptor type 1 OS=Homo sapiens OX=9606 GN=CR1 PE=1 SV=3	CR1_HUMAN	CR1	224 kDa	16	9	8		16	14	4	18	40	14	19	42	33	57	57	22	25	30	44	4	18	26	37	35	9		22	21	6		38	22	19	6	6	4	8	19	46	1	9	20	25		5	34	23
152	Annexin A4 OS=Homo sapiens OX=9606 GN=ANXA4 PE=1 SV=4	ANXA4_HUMAN	ANXA4	36 kDa	18	17	14	11	16	20	25	21	9	9	15	34	25	31	8	20	25	26	25	19	15	11	21	15	49	12	13	26	12	15	12	18	10	16	17	15	15	16	24	21	21	26	14	27	67	29	11
153	DnaJ homolog subfamily C member 13 OS=Homo sapiens OX=9606 GN=DNAJC13 PE=1 SV=5	DJC13_HUMAN	DNAJC13	254 kDa	6	15	3	6	5	8	5	8	15	10	9	23	29	32	30	15	25	13	15	13	17	19	17	15	8	17	27	11	6	12	20	14	20	16	4	33	27	61	56	21	34	20	41	37	20	37	54
154	Coiled-coil and C2 domain-containing protein 1A OS=Homo sapiens OX=9606 GN=CC2D1A PE=1 SV=1	C2D1A_HUMAN	CC2D1A	104 kDa	10	13	13	9	12	11	5	10	9	11	14	12	16	10	11	11	13	12	10	10	9	13	11	14	21	19	20	14	29	15	27	23	26	21	17	37	17	25	23	29	29	23	18	21	19	12	27

155	26S proteasome non-ATPase regulatory subunit 11 OS=Homo sapiens OX=9606 GN=PSMD11 PE=1 SV=3	PSD11_HUMAN	PSMD11	47 kDa	28	27	16	7	16	14	29	7	12	22	3	10	16	15	7	16	29	23	23	8	32	28	31	12	2	20	31	26	10	19	22	15	40	31	20	26	26	58	22	12	20	30	40	24	12	3	29
156	Plasma membrane calcium-transporting ATPase 1 OS=Homo sapiens OX=9606 GN=ATP2B1 PE=1 SV=4	AT2B1_HUMAN	ATP2B1	135 kDa	51	55	58	44	39	44	47	45	56	45	52	52	58	61	57	51	49	48	49	52	48	42	52	49	42	64	54	60	39	43	61	46	43	42	48	34	41	40	43	57	52	63	40	57	40	51	44
157	Copine-3 OS=Homo sapiens OX=9606 GN=CPNE3 PE=1 SV=1	CPNE3_HUMAN	CPNE3	60 kDa	19	17	21	22	17	19	22	20	15	9	18	30	16	31	18	21	22	31	31	25	27	14	32	24	12	17	13	20	21	20	19	22	14	8	15	8	20	15	30	20	19	17	23	25	17	35	27
158	Annexin A7 OS=Homo sapiens OX=9606 GN=ANXA7 PE=1 SV=3	ANXA7_HUMAN	ANXA7	53 kDa	38	20	10		23	26	38	37				17	8	12	13	11	16	29	29	23	29	18	21	0	17	3	1	50		20	8	19	8	23	41	36	18	32	31	44	21	28	16	22	28	23	12
159	F-actin-capping protein subunit beta OS=Homo sapiens OX=9606 GN=CAPZB PE=1 SV=4	CAPZB_HUMAN	CAPZB	31 kDa	18	22	13	12	11	14	20	20	20	16	14	27	22	23	36	27	29	32	28	30	28	34	27	17	13	19	21	25	6	14	23	12	16	16	22	20	18	25	32	34	28	27	24	29	27	24	23
160	26S proteasome non-ATPase regulatory subunit 6 OS=Homo sapiens OX=9606 GN=PSMD6 PE=1 SV=1	PSMD6_HUMAN	PSMD6	46 kDa	16	11	8	5	10	23	11	7	20	20	10	10	15	11	9	9	24	23	19	7	35	24	31	13	1	23	37	34	7	16	25	14	30	21	14	28	18	48	27	9	25	23	48	25	15	5	20
161	Complement decay-accelerating factor OS=Homo sapiens OX=9606 GN=CD55 PE=1 SV=4	DAF_HUMAN	CD55	41 kDa	22	25	24	20	20	22	19	20	28	22	20	21	20	26	21	23	23	19	22	23	19	23	22	17	20	22	21	25	22	21	26	24	28	22	22	20	21	26	29	22	26	24	21	19	15	25	25
162	26S proteasome regulatory subunit 4 OS=Homo sapiens OX=9606 GN=PSMC1 PE=1 SV=1	PRS4_HUMAN	PSMC1	49 kDa	18	19	17	8	11	16	17	11	14	20	8	9	19	22	11	13	26	25	18	14	30	25	28	19	4	24	35	28	10	20	23	14	37	28	12	40	19	50	33	15	21	22	51	26	17	8	30
163	Intercellular adhesion molecule 4 OS=Homo sapiens OX=9606 GN=ICAM4 PE=1 SV=1	ICAM4_HUMAN	ICAM4	29 kDa	18	23	17	9	21	11	17	18	20	12	22	29	14	25	28	32	25	17	28	15	28	26	28	23	24	27	14	18	19	17	18	14	11	16	18	24	23	13	25	17	23	28	10	23	28	29	12
164	Endoplasmin OS=Homo sapiens OX=9606 GN=HSP90B1 PE=1 SV=1	ENPL_HUMAN	HSP90B1	92 kDa	27	16	23	17	9	14	19	24	27	22	35	36	28	36	40	30	32	18	18	31	29	17	27	20	8	25	22	24	12	13	16	13	22	23	17	19	18	24	18	13	14	33	22	22	13	25	16
165	Proteasome adapter and scaffold protein ECM29 OS=Homo sapiens OX=9606 GN=ECPAS PE=1 SV=2	ECM29_HUMAN	ECPAS	204 kDa	11	24	12	4	16	12	10	11	15	23	1	6	10	17	13	9	29	26	26	6	31	28	20	8		18	33	21	2	10	21	11	29	22	17	36	26	61	33	8	16	19	41	33	6	3	35
166	26S proteasome regulatory subunit 10B OS=Homo sapiens OX=9606 GN=PSMC6 PE=1 SV=1	PRS10_HUMAN	PSMC6	44 kDa	20	25	19	12	9	10	20	14	18	21	8	9	16	17	11	9	30	22	19	7	28	21	27	19	6	24	32	31	11	14	20	17	26	25	17	35	22	42	27	15	18	18	28	19	15	7	33
167	Vacuolar protein sorting-associated protein 13A OS=Homo sapiens OX=9606 GN=VPS13A PE=1 SV=2	VP13A_HUMAN	VPS13A	360 kDa	12	10	9	8	7	11	8	12	15	15	16	26	19	24	17	9	24	11	23	22	12	13	17	18	7	18	8	22	5	9	11	5	8	18	5	16	7	15	10	12	18	24	13	19	16	17	16
168	Stress-induced-phosphoprotein 1 OS=Homo sapiens OX=9606 GN=STIP1 PE=1 SV=1	STIP1_HUMAN	STIP1	63 kDa	14	7	5	4	10	14	15	8	5	9	4	13	14	19	12	6	24	30	24	13	26	25	29	12	6	33	44	20	5	19	20	8	29	7	13	16	17	65	35	18	26	10	61	34	17	11	29
169	TBC1 domain family member 24 OS=Homo sapiens OX=9606 GN=TBC1D24 PE=1 SV=2	TBC24_HUMAN	TBC1D24	63 kDa	13	24	13	19	18	19	15	18	18	15	15	27	28	29	28	27	28	32	28	32	24	27	27	12	9	10	12	12	9	11	10	7	10	4	12	9	19	21	18	21	20	21	16	14	13	30	17
170	ATP-binding cassette sub-family G member 2 OS=Homo sapiens OX=9606 GN=ABCG2 PE=1 SV=3	ABCG2_HUMAN	ABCG2	72 kDa	18	16	19	20	14	19	15	11	12	16	21	29	25	20	19	27	25	25	23	26	22	19	16	20	25	26	12	26	16	16	20	9	10	11	14	15	16	14	23	25	26	27	17	23	24	16	10
171	Carbonic anhydrase 1 OS=Homo sapiens OX=9606 GN=CA1 PE=1 SV=2	CAH1_HUMAN	CA1	29 kDa	12	12	10	10	11	23	19	28	30	15	18	16	15	29	13	10	12	19	12	7	12	3	17	19	16	59	25	24	15	23	20	34	18	17	18	12	13	18	25	20	17	15	15	31	22	30	26
172	26S proteasome non-ATPase regulatory subunit 12 OS=Homo sapiens OX=9606 GN=PSMD12 PE=1 SV=3	PSD12_HUMAN	PSMD12	53 kDa	18	16	12	5	10	16	12	8	13	27	7	8	13	10	6	10	22	14	18	7	30	19	23	14	4	16	34	25	9	16	14	16	37	32	19	39	15	44	23	11	13	16	44	20	10	4	28
173	Calpastatin OS=Homo sapiens OX=9606 GN=CAST PE=1 SV=4	ICAL_HUMAN	CAST	77 kDa	35	18	9		18	28	21	3	8	19	0	6	12	11	0	8	18	39	30	11	42	30	39	15		31	60	31	1	9	7	4	25	25	7	18	12	51	33	8	9	15	60	20	3	3	16
174	Alpha-centractin OS=Homo sapiens OX=9606 GN=ACTR1A PE=1 SV=1	ACTZ_HUMAN	ACTR1A	43 kDa	19	27	16	13	14	19	19	15	18	26	9	12	25	19	18	20	29	18	20	15	24	29	20	18	9	16	18	20	6	12	20	4	16	15	21	21	25	23	31	13	15	26	22	17	15	17	14
175	Importin subunit beta-1 OS=Homo sapiens OX=9606 GN=KPNB1 PE=1 SV=2	IMB1_HUMAN	KPNB1	97 kDa	37	25	18	5	27	19	20	16	15	17	5	3	10	12	7	5	17	28	22	9	30	22	23	11	3	16	45	25	1	13	12	7	29	22	9	16	8	53	28	7	13	12	45	19	11	8	13
176	Immunoglobulin heavy constant gamma 3 OS=Homo sapiens OX=9606 GN=IGHG3 PE=1 SV=2	IGHG3_HUMAN	IGHG3	41 kDa	18	13	19	20	25	28	38	28	35	30	27	40	40	42	67	62	53	44	45	32	44	40	43	39	27	42	48	59	24	74	45	16	25	24	37	24	44	24	57	101	39	47	39	50	79	59	10
177	Tensin-1 OS=Homo sapiens OX=9606 GN=TNS1 PE=1 SV=2	TENS1_HUMAN	TNS1	186 kDa	1	7	5	7	5	5	5	7	6	10	10	17	18	12	30	16	31	24	19	25	11	23	16	13	20	17	15	16	9	17	21	11	12	10	11	13	9	11	26	31	22	23	12	20	13	18	11
178	Casein kinase I isoform alpha OS=Homo sapiens OX=9606 GN=CSNK1A1 PE=1 SV=2	KC1A_HUMAN	CSNK1A1	39 kDa	20	20	23	15	15	19	16	15	14	22	12	11	14	24	14	12	14	15	20	12	18	22	20	27	15	14	23	25	19	18	15	17	24	19	21	16	7	26	10	16	13	18	23	12	8	10	7
179	Galectin-3 OS=Homo sapiens OX=9606 GN=LGALS3 PE=1 SV=5	LEG3_HUMAN	LGALS3	26 kDa	17	22	21	12	18	11	19	17	17	16	21	17	18	14	20	18	19	15	17	15	17	18	17	15	17	14	16	13	18	15	16	19	13	12	16	14	16	16	16	15	17	12	15	14	19	18	16
180	26S proteasome non-ATPase regulatory subunit 5 OS=Homo sapiens OX=9606 GN=PSMD5 PE=1 SV=3	PSMD5_HUMAN	PSMD5	56 kDa	19	17	6	3	17	13	13	7	7	18	3	5	10	11	5	4	26	29	27	5	36	29	32	7		14	40	22	1	8	7	4	29	16	8	23	16	68	21	2	6	16	46	24	2	5	25
181	Keratin, type II cytoskeletal 2 epidermal OS=Homo sapiens OX=9606 GN=KRT2 PE=1 SV=2	K22E_HUMAN	KRT2	65 kDa	15	16	14	13	20	21	15	16	18	25	33	30	49	43	23	32	48	34	37	32	24	20	28	36	15	38	28	20	13	17	24	12	14	13	15	15	12	13	29	15	22	24	23	27	18	39	11
182	Gamma-adducin OS=Homo sapiens OX=9606 GN=ADD3 PE=1 SV=1	ADDG_HUMAN	ADD3	79 kDa	13	15	19	10	10	14	10	20	21	16	15	15	16	22	21	16	15	21	19	16	19	12	17	12	21	15	13	18	12	13	18	17	9	12	20	11	14	10	21	14	20	12	11	15	19	11	20
183	Ras-related protein Rab-21 OS=Homo sapiens OX=9606 GN=RAB21 PE=1 SV=3	RAB21_HUMAN	RAB21	24 kDa	18	23	19	12	13	17	14	11	20	16	19	14	17	17	13	9	16	12	12	15	13	16	13	14	22	9	13	15	19	12	25	15	20	17	15	23	16	16	17	14	21	14	14	13	18	11	22
184	Keratin, type II cytoskeletal 6A OS=Homo sapiens OX=9606 GN=KRT6A PE=1 SV=3	K2C6A_HUMAN	KRT6A	60 kDa	21	18	9	11	9	20	12	13	8	14	29	22	69	23	5	29	22	16	17	18	28	7	19	27	8	37	15	9	7	7	9	14	9	5	4	27	6	13	26	3	12	14	11	14	6	15	7
185	Radixin OS=Homo sapiens OX=9606 GN=RDX PE=1 SV=1	RADI_HUMAN	RDX	69 kDa	29	15	31	12	23	31	26	20	24	27	27	24	15	26	17	27	23	32	24	14	29	25	29	20	22	28	38	32	11	24	24	15	32	17	23	13	22	40	24	18	17	23	30	34	26	19	24
186	Fatty acid synthase OS=Homo sapiens OX=9606 GN=FASN PE=1 SV=3	FAS_HUMAN	FASN	273 kDa	27	10	6	2	16	12	13	3	6	11	1	1	3	6	1	1	14	17	15	1	21	14	19	3		12	65	24	3	14	6	6	40	24	14	18	19	80	25	2	14	7	69	16	3	1	14
187	14-3-3 protein epsilon OS=Homo sapiens OX=9606 GN=YWHAE PE=1 SV=1	1433E_HUMAN	YWHAE	29 kDa	26	14	9	5	18	19	20	13	10	15	15	15	15	11	7	10	11	16	14	10	14	10	13	22	5	21	27	20	4	8	6	6	8	12	5	19	10	25	15	9	10	14	22	13	12	12	17
188	Ras-related C3 botulinum toxin substrate 1 OS=Homo sapiens OX=9606 GN=RAC1 PE=1 SV=1	RAC1_HUMAN	RAC1	21 kDa	16	17	19	15	17	20	21	18	22	22	23	20	21	19	21	19	17	17	16	19	16	19	18	15	13	18	21	14	18	20	19	18	19	23	19	14	18	18	19	19	20	17	18	15	20	14	17
189	Syntaxin-7 OS=Homo sapiens OX=9606 GN=STX7 PE=1 SV=4	STX7_HUMAN	STX7	30 kDa	12	19	17	15	21	16	15	15	19	12	19	16	20	19	15	21	20	17	19	17	12	20	19	22	16	21	18	18	13	18	18	17	18	17	13	16	14	17	22	17	15	19	16	18	18	20	16
190	Neutral cholesterol ester hydrolase 1 OS=Homo sapiens OX=9606 GN=NCEH1 PE=1 SV=3	NCEH1_HUMAN	NCEH1	46 kDa	13	11	20	13	13	13	19	14	14	18	18	24	22	21	25	18	20	10	12	17	24	15	21	19	11	20	11	15	7	11	9	15	14	13	13	18	9	16	17	14	13	21	14	16	8	19	15
191	Immunoglobulin kappa constant OS=Homo sapiens OX=9606 GN=IGKC PE=1 SV=2	IGKC_HUMAN	IGKC	12 kDa	61	52	29	54	60	47	116	104	70	55	91	78	39	53	116	78	57	52	44	44	23	41	45	45	52	41	56	80	36	89	39	35	44	53	64	28	50	31	59	107	42	73	49	71	108	74	23
192	Transforming protein RhoA OS=Homo sapiens OX=9606 GN=RHOA PE=1 SV=1	RHOA_HUMAN	RHOA	22 kDa	16	12	19	18	14	21	12	14	16	16	22	15	12	10	14	10	15	9	18	12	11	15	12	16	9	11	12	12	10	13	14	16	17	16	13	16	12	15	12	14	14	15	17	11	13	13	17
193	CB1 cannabinoid receptor-interacting protein 1 OS=Homo sapiens OX=9606 GN=CNRIP1 PE=1 SV=1	CNRP1_HUMAN	CNRIP1	19 kDa	5	16	5	9	4	6	8	10	9	6	6	28	20	20	21	27	23	20	12	20	28	24	19	13	6	10	10	8	7	11	14	4	6	5	4	27	32	33	28	23	24	18	27	20	21	17	16
194	Phosphatidylinositide phosphatase SAC1 OS=Homo sapiens OX=9606 GN=SACM1L PE=1 SV=2	SAC1_HUMAN	SACM1L	67 kDa	18	20	20	13	10	17	16	21	16	15	18	23	17	21	16	14	25	14	17	15	20	15	14	20	12	15	15	17	10	10	10	13	17	15	13	17	10	17	13	11	8	18	7	13	14	21	17
195	Copper-transporting ATPase 1 OS=Homo sapiens OX=9606 GN=ATP7A PE=1 SV=4	ATP7A_HUMAN	ATP7A	163 kDa	8	11	12	14	9	12	4	7	13	11	18	21	23	25	18	17	19	22	14	27	15	11	15	25	10	10	11	17	7	14	7	9	9	11	6	15	8	8	11	17	12	13	8	14	4	21	10
196	COP9 signalosome complex subunit 4 OS=Homo sapiens OX=9606 GN=COPS4 PE=1 SV=1	CSN4_HUMAN	COPS4	46 kDa	35	25	19	5	15	21	25	9	12	15	1	4	9	11	3	4	20	23	21	8	25	20	19	7		15	38	18		10	6	9	23	14	10	14	10	42	16	1	7	8	29	9	3	1	15
197	Calpain-1 catalytic subunit OS=Homo sapiens OX=9606 GN=CAPN1 PE=1 SV=1	CAN1_HUMAN	CAPN1	82 kDa	24	6	7	6	12	6	6	6	5	7	3	8	12	9	5	8	8	15	22	11	31	17	11	6	9	15	28	11	5	11	4	8	11	12	15	10	14	52	12	15	9	13	36	20	13	8	10
198	Importin-5 OS=Homo sapiens OX=9606 GN=IPO5 PE=1 SV=4	IPO5_HUMAN	IPO5	124 kDa	17	8	7		9	8	9	4	4	9	2	4	3	8	2	1	6	13	16	3	15	7	13		1	18	56	15	5	7	18	12	22	6	13	5	13	77	41	6	26	9	67	31	0	1	32
199	AP-2 complex subunit alpha-2 OS=Homo sapiens OX=9606 GN=AP2A2 PE=1 SV=2	AP2A2_HUMAN	AP2A2	104 kDa	32	29	23	12	19	14	30	11	15	23	22	24	23	18	21	22	35	27	29	19	35	23	35	21	12	36	42	29	33	34	36	27	37	34	37	37	52	67	51	31	30	32	56	46	34	21	49
200	Intermediate conductance calcium-activated potassium channel protein 4 OS=Homo sapiens OX=9606 GN=KCNN4 PE=1 SV=1	KCNN4_HUMAN	KCNN4	48 kDa	18	13	17	18	17	16	16	16	15	18	15	18	16	14	17	14	13	18	19	21	16	11	16	14	16	16	15	9	11	11	19	18	17	12	11	16	15	12	22	14	15	9	13	16	12	15	12
201	SH3 domain-binding glutamic acid-rich-like protein 2 OS=Homo sapiens OX=9606 GN=SH3BGRL2 PE=1 SV=2	SH3L2_HUMAN	SH3BGRL2	12 kDa	10	7	5	8	8	11	11	8	10	17	21	24	14	11	12	15	16	18	11	10	14	30	14	9	12	11	18	9	11	13	18	10	18	17	8	14	10	17	14	13	15	7	14	10	14	13	8
202	Guanine nucleotide-binding protein G(I)/G(S)/G(T) subunit beta-2 OS=Homo sapiens OX=9606 GN=GNB2 PE=1 SV=3	GBB2_HUMAN	GNB2	37 kDa	48	48	55	23	29	41	39	40	40	30	43	40	33	36	38	34	34	37	35	30	37	40	32	42	43	38	39	51	27	44	52	43	38	53	28	38	25	30	57	43	48	47	32	39	57	30	62
203	AP-1 complex subunit beta-1 OS=Homo sapiens OX=9606 GN=AP1B1 PE=1 SV=2	AP1B1_HUMAN	AP1B1	105 kDa	34	29	23	14	15	19	30	13	14	22	24	37	29	22	25	21	44	29	31	20	36	30	44	31	26	55	50	30	31	36	46	26	51	32	33	37	41	60	43	38	41	31	58	40	39	29	38
204	Long-chain-fatty-acid--CoA ligase 3 OS=Homo sapiens OX=9606 GN=ACSL3 PE=1 SV=3	ACSL3_HUMAN	ACSL3	80 kDa	18	14	15	14	10	7	16	10	16	14	17	20	24	19	25	16	26	11	17	17	15	17	14	27	12	11	8	26	10	11	14	13	16	16	14	18	16	15	19	19	11	27	6	16	8	21	11
205	Immunoglobulin heavy constant mu OS=Homo sapiens OX=9606 GN=IGHM PE=1 SV=4	IGHM_HUMAN	IGHM	49 kDa	8	10	2	0	13	25	17	4	8	7	13	25	15	18	26	31	18	41	30	1	12	13	19	14	6	14	7	16	4	18	10	5	1	8	8	14	10	2	24	34	8	13	5	15	19	31	4
206	Beta-2-glycoprotein 1 OS=Homo sapiens OX=9606 GN=APOH PE=1 SV=3	APOH_HUMAN	APOH	38 kDa	18	6	8	18	4	13	14	6	11	15	10	4	9	11	8	6	11	11		10	19	10	19	13	3	7	16	12	12	31	11	6	9	17	19	17	15	9	10	28	9	7	10	9	6	20	2
207	Myosin regulatory light chain 12B OS=Homo sapiens OX=9606 GN=MYL12B PE=1 SV=2	ML12B_HUMAN	MYL12B	20 kDa	5	9	5	7	3	4	5	4	4	5	3	4	10	9	11	10	14	14	10	13	8	9	12	6	4	28	14	7	7	9	62	9	9	6	15	5	36	19	100	21	28	10	23	47	17	5	46
208	Ubiquitin carboxyl-terminal hydrolase 15 OS=Homo sapiens OX=9606 GN=USP15 PE=1 SV=3	UBP15_HUMAN	USP15	112 kDa	34	15	8	3	16	14	19	9	12	14	4	2	6	7	4	2	14	25	21	5	30	23	20	8	1	20	36	13	1	11	4	5	18	13	10	16	10	45	19	3	16	14	44	22	3	1	14
209	Transmembrane protein 222 OS=Homo sapiens OX=9606 GN=TMEM222 PE=1 SV=2	TM222_HUMAN	TMEM222	23 kDa	20	20	22	13	12	15	16	11	16	17	17	19	10	14	18	14	13	12	16	17	18	15	13	14	10	12	14	18	9	14	11	13	13	11	11	11	14	16	17	15	21	16	11	14	11	14	17
210	Protein XRP2 OS=Homo sapiens OX=9606 GN=RP2 PE=1 SV=4	XRP2_HUMAN	RP2	40 kDa	14	16	12	11	13	13	13	13	15	11	12	12	19	19	20	16	16	15	16	12	20	12	16	15	16	12	12	12	7	9	8	10	8	6	9	7	11	7	13	13	6	17	11	12	9	21	14
211	Polyubiquitin-B OS=Homo sapiens OX=9606 GN=UBB PE=1 SV=1	UBB_HUMAN (+1)	UBB	26 kDa	13	9	12	6	7	14	10	9	14	7	12	27	21	20	22	16	17	22	24	23	18	9	13	10	7	17	13	14	11	9	17	12	14	13	16	6	12	14	14	11	17	16	16	13	12	21	15
212	Peptidyl-prolyl cis-trans isomerase FKBP3 OS=Homo sapiens OX=9606 GN=FKBP3 PE=1 SV=1	FKBP3_HUMAN	FKBP3	25 kDa	8	8	18	8	5	11	9	8	7	6	14	11	10	6	8	13	15	11	8	11	16	8	16	11	13	27	15	16	17	21	17	16	12	12	17	13	20	12	24	19	17	18	17	20	17	16	20
213	26S proteasome non-ATPase regulatory subunit 14 OS=Homo sapiens OX=9606 GN=PSMD14 PE=1 SV=1	PSDE_HUMAN	PSMD14	35 kDa	13	15	12	7	11	12	12	7	8	14	5	4	13	10	6	6	16	15	11	8	17	14	16	8	2	14	18	20	6	15	14	12	23	19	16	21	12	34	18	16	15	19	26	16	10	4	20
214	Phosphoribosyl pyrophosphate synthase-associated protein 2 OS=Homo sapiens OX=9606 GN=PRPSAP2 PE=1 SV=1	KPRB_HUMAN	PRPSAP2	41 kDa	22	12	9	4	12	18	19	8	5	9	3	9	9	7	9	13	14	17	15	9	19	17	15	5	2	13	31	15	4	9	13	5	14	11	2	20	9	40	23	14	19	13	27	18	12	11	20
215	Vesicular integral-membrane protein VIP36 OS=Homo sapiens OX=9606 GN=LMAN2 PE=1 SV=1	LMAN2_HUMAN	LMAN2	40 kDa	7	6	4	4	3	6	7	3	13	6	8	16	10	16	8	8	12	8	14	14	14	9	9	15	9	18	17	8	10	5	15	13	17	19	9	13	3	17	15	15	14	10	15	14	16	21	13
216	SH3 domain-binding glutamic acid-rich-like protein OS=Homo sapiens OX=9606 GN=SH3BGRL PE=1 SV=1	SH3L1_HUMAN	SH3BGRL	13 kDa	19	19	12	15	12	13	6	17	19	13	17	11	16	22	10	8	4	5	6	6	20	13	6	14	15	18	15	12	10	12	13	14	11	15	14	13	16	23	11	10	12	12	17	15	10	9	22
217	Solute carrier family 40 member 1 OS=Homo sapiens OX=9606 GN=SLC40A1 PE=1 SV=1	S40A1_HUMAN	SLC40A1	63 kDa	14	11	13	14	9	12	13	12	17	20	15	11	14	17	14	13	12	16	14	17	12	13	13	10	16	14	12	12	12	12	16	13	8	12	15	14	12	13	13	15	14	13	11	16	9	15	10
218	Guanine nucleotide-binding protein subunit alpha-13 OS=Homo sapiens OX=9606 GN=GNA13 PE=1 SV=2	GNA13_HUMAN	GNA13	44 kDa	14	19	14	9	15	14	14	13	19	17	19	19	14	19	21	16	21	17	16	12	14	22	17	14	31	21	21	14	17	16	24	20	19	24	16	18	12	23	31	16	24	13	20	17	26	18	23
219	G protein-coupled receptor kinase 6 OS=Homo sapiens OX=9606 GN=GRK6 PE=1 SV=2	GRK6_HUMAN	GRK6	66 kDa	8	9	15	7	9	13	9	7	11	13	8	12	13	10	13	14	10	14	10	15	16	10	7	12	10	14	13	12	10	13	10	12	11	12	14	10	7	6	14	13	14	15	14	17	17	7	8
220	Phosphatidylinositol 4-kinase type 2-alpha OS=Homo sapiens OX=9606 GN=PI4K2A PE=1 SV=1	P4K2A_HUMAN	PI4K2A	54 kDa	13	10	14	14	10	16	9	10	19	15	16	13	17	15	13	6	11	17	12	17	13	12	19	16	6	11	17	4	9	5	9	12	19	10	10	10	8	14	10	6	9	12	15	11	6	14	15
221	Small membrane A-kinase anchor protein OS=Homo sapiens OX=9606 GN=C2orf88 PE=1 SV=2	SMAKA_HUMAN	C2orf88	11 kDa	10	13	10	12	9	18	11	19	13	16	6	7	15	11	16	8	9	9	8	9	11	10	15	14	10	15	11	19	15	20	16	13	14	15	16	8	13	7	12	21	18	14	10	14	14	15	21
222	Peptidyl-prolyl cis-trans isomerase B OS=Homo sapiens OX=9606 GN=PPIB PE=1 SV=2	PPIB_HUMAN	PPIB	24 kDa	10	6	11	10	2	6	12	9	13	12	17	16	15	14	16	13	12	16	9	13	20	10	16	10	12	16	13	20	7	11	11	14	13	13	12	10	12	11	16	14	9	20	13	18	12	14	10
223	Heat shock 70 kDa protein 4 OS=Homo sapiens OX=9606 GN=HSPA4 PE=1 SV=4	HSP74_HUMAN	HSPA4	94 kDa	23	16	7	1	15	19	21	4	4	9		0	1	3	1		8	25	17	0	27	23	17	3		16	53	16	1	4	5	1	19	14	7	9	5	40	25	6	4	9	47	8	1	1	6
224	Retinal dehydrogenase 1 OS=Homo sapiens OX=9606 GN=ALDH1A1 PE=1 SV=2	AL1A1_HUMAN	ALDH1A1	55 kDa	49	12	2	2	18	26	26	5	13	20	2	2	4	8	0		10	26	12	7	19	15	15	8		21	54	28		7		2	9	10	11	7	1	47	19	5	5	9	29	15	1	4	6
225	Endoplasmic reticulum resident protein 44 OS=Homo sapiens OX=9606 GN=ERP44 PE=1 SV=1	ERP44_HUMAN	ERP44	47 kDa	9	11	12	8	6	6	10	10	12	11	18	9	16	11	11	11	10	5	12	12	10	11	10	9	16	15	11	10	8	10	9	16	11	12	15	10	11	12	10	10	9	17	11	17	14	13	11
226	Myosin light chain 4 OS=Homo sapiens OX=9606 GN=MYL4 PE=1 SV=3	MYL4_HUMAN	MYL4	22 kDa	5	9	3	3	2	5	2	2	5	6	9	4	18	9	11	7	11	15	12	13	9	9	6	9	10	28	23	4	5	8	31	14	9	6	13	12	23	21	40	18	16	12	29	31	27	8	19
227	CD44 antigen OS=Homo sapiens OX=9606 GN=CD44 PE=1 SV=3	CD44_HUMAN	CD44	82 kDa	9	12	14	17	10	15	12	12	13	19	13	18	23	24	14	12	13	17	23	25	8	13	12	14	9	12	10	10	9	15	12	9	13	8	10	6	9	11	8	14	9	9	7	14	8	19	10
228	Ras-related protein Rab-5C OS=Homo sapiens OX=9606 GN=RAB5C PE=1 SV=2	RAB5C_HUMAN	RAB5C	23 kDa	6	13	11	13	10	9	7	11	8	10	13	16	18	17	15	13	12	12	20	16	14	17	17	12	12	11	11	14	9	8	16	9	11	12	12	11	10	9	15	12	16	11	16	11	20	16	11
229	Syntaxin-binding protein 3 OS=Homo sapiens OX=9606 GN=STXBP3 PE=1 SV=2	STXB3_HUMAN	STXBP3	68 kDa	12	17	14	10	11	17	14	17	12	13	15	17	11	11	15	13	13	12	15	14	18	19	15	14	11	7	5	20	3	9	11	1	5	8	6	5	6	8	16	14	9	18	5	9	13	20	5
230	Cullin-1 OS=Homo sapiens OX=9606 GN=CUL1 PE=1 SV=2	CUL1_HUMAN	CUL1	90 kDa	24	8	7	0	5	6	7	8	9	8	2	7	6	11	11	13	13	23	11	10	19	18	17	9		13	26	18	1	4	2	6	10	8	5	12	11	32	16	8	9	8	31	16	7	10	21
231	DnaJ homolog subfamily B member 1 OS=Homo sapiens OX=9606 GN=DNAJB1 PE=1 SV=4	DNJB1_HUMAN	DNAJB1	38 kDa	10	10	10	11	9	7	7	8	14	5	9	10	7	10	8	9	12	13	10	15	12	10	12	12	11	9	13	13	6	10	9	4	16	11	10	10	11	16	9	13	12	12	12	8	8	16	9
232	Zinc transporter 1 OS=Homo sapiens OX=9606 GN=SLC30A1 PE=1 SV=3	ZNT1_HUMAN	SLC30A1	55 kDa	12	10	10	9	8	13	12	11	14	17	6	9	11	14	17	8	12	15	12	14	15	6	14	13	5	10	9	10	4	3	17	8	7	5	6	10	5	8	20	8	8	9	13	14	9	11	9
233	Protein arginine N-methyltransferase 5 OS=Homo sapiens OX=9606 GN=PRMT5 PE=1 SV=4	ANM5_HUMAN	PRMT5	73 kDa	28	15	8	1	6	10	15	3	11	11	2	1	5	9		1	11	20	16	6	17	12	21	6	1	19	27	7	1	8	3	4	20	17	6	7	11	34	8	3	8	6	34	13	1		16
234	Guanine nucleotide-binding protein G(i) subunit alpha-2 OS=Homo sapiens OX=9606 GN=GNAI2 PE=1 SV=3	GNAI2_HUMAN	GNAI2	40 kDa	39	34	36	41	43	63	44	40	42	33	33	59	24	48	58	57	54	67	63	48	49	53	33	45	60	50	51	60	42	37	53	39	36	46	42	43	40	43	51	55	54	50	48	54	51	51	53
235	Very-long-chain 3-oxoacyl-CoA reductase OS=Homo sapiens OX=9606 GN=HSD17B12 PE=1 SV=2	DHB12_HUMAN	HSD17B12	34 kDa	6	13	10	9	5	8	12	10	12	8	11	24	16	13	17	16	16	6	10	16	18	13	12	14	13	13	10	11	10	8	7	12	10	10	8	9	7	10	10	10	4	7	10	11	12	17	13
236	Vesicle-trafficking protein SEC22b OS=Homo sapiens OX=9606 GN=SEC22B PE=1 SV=4	SC22B_HUMAN	SEC22B	25 kDa	14	16	17	8	10	13	11	10	16	13	18	12	12	11	11	10	10	12	13	11	11	11	10	13	8	13	15	17	7	9	12	12	18	11	16	11	10	18	12	12	10	16	12	12	12	12	14
237	Junctional adhesion molecule A OS=Homo sapiens OX=9606 GN=F11R PE=1 SV=1	JAM1_HUMAN	F11R	33 kDa	9	10	12	8	12	11	10	11	12	12	10	12	13	13	19	12	9	10	15	16	18	17	15	11	9	13	6	14	9	8	14	14	8	9	11	7	10	8	13	9	14	13	12	15	9	10	12
238	BMP-2-inducible protein kinase OS=Homo sapiens OX=9606 GN=BMP2K PE=1 SV=2	BMP2K_HUMAN	BMP2K	129 kDa	12	12	13	6	7	12	11	7	8	11	13	6	6	7	10	8	14	8	7	6	15	11	12	5	7	11	17	14	4	12	11	6	16	13	6	10	13	21	18	7	13	15	18	10	11	7	13
239	Phospholipid transfer protein C2CD2L OS=Homo sapiens OX=9606 GN=C2CD2L PE=1 SV=3	C2C2L_HUMAN	C2CD2L	76 kDa	11	8	14	11	4	6	8	9	15	8	8	14	9	12	12	7	9	10	10	15	14	11	11	11	11	18	11	12	5	8	9	7	10	12	9	9	8	14	13	12	9	18	8	17	14	9	13
240	Moesin OS=Homo sapiens OX=9606 GN=MSN PE=1 SV=3	MOES_HUMAN	MSN	68 kDa	23	15	25	7	13	18	22	17	15	19	22	18	12	14	13	20	15	19	17	8	18	16	18	15	18	19	27	21	14	19	18	14	23	13	13	8	22	22	17	13	11	16	15	20	20	12	17
241	Guanine nucleotide-binding protein G(q) subunit alpha OS=Homo sapiens OX=9606 GN=GNAQ PE=1 SV=4	GNAQ_HUMAN	GNAQ	42 kDa	10	8	11	6	6	11	9	10	10	13	12	12	11	10	9	11	10	10	12	11	14	13	12	16	13	11	11	13	10	9	18	13	10	10	11	10	6	7	13	11	13	13	10	16	17	16	11
242	Unconventional myosin-XVIIIa OS=Homo sapiens OX=9606 GN=MYO18A PE=1 SV=3	MY18A_HUMAN	MYO18A	233 kDa	4	8	2	7	1	5	3	3	1	7	0	6	7	6	10	13	18	25	12	20	10	9	14	1	4	4	8	7	2	5	19	3	5	4	7	6	14	18	45	18	18	21	14	8	19	7	10
243	Beta-actin-like protein 2 OS=Homo sapiens OX=9606 GN=ACTBL2 PE=1 SV=2	ACTBL_HUMAN	ACTBL2	42 kDa	50	51	57	38	41	48	38	44	40	41	54	38	33	34	37	37	37	33	37	32	37	42	30	33	36	32	39	41	45	42	43	44	54	52	52	36	40	39	30	40	39	43	41	34	41	39	50
244	F-actin-capping protein subunit alpha-1 OS=Homo sapiens OX=9606 GN=CAPZA1 PE=1 SV=3	CAZA1_HUMAN	CAPZA1	33 kDa	11	13	4	2	5	1	9	11	3	3	3	13	13	16	15	12	19	14	17	16	8	16	16	10	3	9	8	8	1	5	3	0	4	4	4	3	12	8	22	20	13	14	6	13	12	16	7
245	Protein disulfide-isomerase A6 OS=Homo sapiens OX=9606 GN=PDIA6 PE=1 SV=1	PDIA6_HUMAN	PDIA6	48 kDa	9	9	9	7	6	11	13	13	12	13	16	16	13	13	15	10	17	10	10	12	16	9	13	9	9	11	8	15	5	9	6	8	9	14	11	12	7	7	7	7	6	12	9	10	11	12	6
246	Ras-related protein Rab-8B OS=Homo sapiens OX=9606 GN=RAB8B PE=1 SV=2	RAB8B_HUMAN	RAB8B	24 kDa	14	16	17	15	10	13	11	14	16	11	13	17	12	17	19	16	15	14	12	18	14	14	14	19	19	13	19	17	14	18	20	17	19	19	17	19	13	15	15	18	22	21	13	15	18	18	19
247	Vesicle-associated membrane protein-associated protein A OS=Homo sapiens OX=9606 GN=VAPA PE=1 SV=3	VAPA_HUMAN	VAPA	28 kDa	13	7	12	15	11	12	11	6	11	13	12	13	14	19	15	18	18	11	16	17	15	15	17	8	8	17	11	14	10	8	11	10	10	16	14	10	11	11	10	10	10	16	14	10	13	17	11
248	Ras-related protein Ral-A OS=Homo sapiens OX=9606 GN=RALA PE=1 SV=1	RALA_HUMAN	RALA	24 kDa	8	8	6	11	4	5	6	8	6	4	4	11	6	9	10	8	6	10	10	11	6	4	8	14	6	6	7	5	6	6	7	5	7	10	8	7	8	7	5	7	6	4	6	9	7	12	9
249	COP9 signalosome complex subunit 2 OS=Homo sapiens OX=9606 GN=COPS2 PE=1 SV=1	CSN2_HUMAN	COPS2	52 kDa	18	10	6	1	8	15	9	1	4	10	2	1	1	1	1	4	13	21	10	1	19	13	17	6		11	32	31		6	2	2	21	5	3	11	4	36	14	1	1	12	35	10	1		8
250	Peroxiredoxin-1 OS=Homo sapiens OX=9606 GN=PRDX1 PE=1 SV=1	PRDX1_HUMAN	PRDX1	22 kDa	22	9	3	8	16	21	10	9	15	16	7	7	12	12	6	7	20	19	19	14	25	21	16	8	5	28	34	25	7	14	8	8	24	16	16	12	13	30	19	15	15	17	32	24	14	8	12
251	COP9 signalosome complex subunit 3 OS=Homo sapiens OX=9606 GN=COPS3 PE=1 SV=3	CSN3_HUMAN	COPS3	48 kDa	20	12	13	4	14	15	14	7	9	12	4	3	8	7	2	5	13	15	8	8	21	14	18	8	4	14	29	16	1	7	5	6	21	19	9	12	7	31	13	5	5	12	26	8	3	3	14
252	26S proteasome non-ATPase regulatory subunit 7 OS=Homo sapiens OX=9606 GN=PSMD7 PE=1 SV=2	PSMD7_HUMAN	PSMD7	37 kDa	13	13	10	2	8	4	9	4	6	12	1	5	14	6	1	1	18	15	10	3	20	13	20	6	2	13	19	13	3	5	6	6	21	21	7	19	11	35	15	2	7	8	29	12	3	1	12
253	WD repeat-containing protein 81 OS=Homo sapiens OX=9606 GN=WDR81 PE=1 SV=2	WDR81_HUMAN	WDR81	212 kDa	5	5	3		6	5	4		0	6	0	0	3	1	1	2	17	12	12	0	21	11	16	2		2	16	9	2	4	7	2	21	11	1	25	11	41	19	3	7	10	35	15		0	12
254	Small integral membrane protein 1 OS=Homo sapiens OX=9606 GN=SMIM1 PE=1 SV=1	SMIM1_HUMAN	SMIM1	9 kDa	11	14	13	8	12	8	10	8	8	8	11	15	12	11	10	13	11	7	18	14	13	10	13	11	14	9	5	21	9	6	7	9	12	8	12	7	12	6	8	9	11	15	6	7	10	14	10
255	Calmodulin-1 OS=Homo sapiens OX=9606 GN=CALM1 PE=1 SV=1	CALM1_HUMAN (+2)	CALM1	17 kDa	9	7	6	5	6	6	9	6	10	13	7	15	14	9	10	12	8	11	11	10	15	10	12	10	11	13	14	9	6	8	8	10	10	11	9	6	10	13	14	9	13	11	12	12	14	9	11
256	Heat shock protein HSP 90-beta OS=Homo sapiens OX=9606 GN=HSP90AB1 PE=1 SV=4	HS90B_HUMAN	HSP90AB1	83 kDa	23	16	11	9	22	24	16	13	15	25	13	11	15	22	15	12	25	28	27	14	35	25	29	13	8	39	82	19	14	24	19	18	34	19	24	22	23	124	50	18	28	13	73	34	14	8	33
257	Urea transporter 1 OS=Homo sapiens OX=9606 GN=SLC14A1 PE=1 SV=2	UT1_HUMAN	SLC14A1	43 kDa	6	6	6	11	6	10	7	6	7	7	13	15	13	15	9	9	13	10	13	11	9	10	10	6	7	5	9	7	10	9	5	8	10	12	11	6	8	8	12	5	7	11	10	8	10	9	9
258	LanC-like protein 2 OS=Homo sapiens OX=9606 GN=LANCL2 PE=1 SV=1	LANC2_HUMAN	LANCL2	51 kDa	6	7	6	4	5	5	6	3	6	6	4	15	17	16	14	20	15	16	16	17	21	16	18	7	4	15	12	9	6	3	2	4	4	4	4	6	8	12	17	14	13	16	17	22	13	17	9
259	Ras-related protein Rap-1b OS=Homo sapiens OX=9606 GN=RAP1B PE=1 SV=1	RAP1B_HUMAN	RAP1B	21 kDa	108	43	103	68	66	100	94	64	94	83	79	64	46	47	52	58	69	54	44	50	56	53	56	69	104	65	60	77	62	74	85	85	92	81	73	68	62	69	63	50	43	80	64	47	79	69	76
260	Ubiquitin carboxyl-terminal hydrolase 14 OS=Homo sapiens OX=9606 GN=USP14 PE=1 SV=3	UBP14_HUMAN	USP14	56 kDa	22	10	4	2	11	11	12	6	8	9	1	3	5	5	2	3	5	10	9	2	16	16	13	5		16	28	9	2	4	3	7	16	13	5	12	9	45	19	1	9	5	35	15	2	3	18
261	Vesicle-fusing ATPase OS=Homo sapiens OX=9606 GN=NSF PE=1 SV=3	NSF_HUMAN	NSF	83 kDa	16	13	6	5	12	11	12	9	7	9	11	11	8	12	8	6	14	7	10	6	14	10	11	5	5	7	11	10	5	5	6	8	11	8	5	8	5	23	11	3	5	8	13	9	5	13	32
262	Ras-related protein Rab-7a OS=Homo sapiens OX=9606 GN=RAB7A PE=1 SV=1	RAB7A_HUMAN	RAB7A	23 kDa	5	6	5	4	3	5	8	3	6	3	8	13	11	10	11	6	13	14	11	11	16	17	9	15	12	9	15	5	3	6	12	10	11	8	3	10	5	11	23	8	14	7	20	11	19	14	12
263	Tropomyosin alpha-1 chain OS=Homo sapiens OX=9606 GN=TPM1 PE=1 SV=2	TPM1_HUMAN	TPM1	33 kDa	31	24	33	21	31	31	31	23	27	30	24	21	20	24	16	17	27	28	26	25	25	24	23	26	23	26	36	27	23	33	25	25	20	32	28	19	22	31	24	23	25	32	30	29	25	17	27
264	Methyltransferase-like protein 7A OS=Homo sapiens OX=9606 GN=METTL7A PE=1 SV=1	MET7A_HUMAN	METTL7A	28 kDa	17	6	16	16	8	19	8	9	6	8	11	14	6	6	19	10	9	18	9	11	13	6	9	9	9	11	8	8	4	9	6	5	6	5	6	6	9	7	7	12	7	14	8	7	11	11	5
265	Ras-related protein Rab-2B OS=Homo sapiens OX=9606 GN=RAB2B PE=1 SV=1	RAB2B_HUMAN	RAB2B	24 kDa	6	8	7	9	8	9	7	7	8	9	10	11	14	11	16	9	9	11	11	14	14	15	11	10	12	9	11	8	9	6	11	11	10	7	6	9	7	10	16	10	17	10	17	8	17	19	13
266	Calpain small subunit 1 OS=Homo sapiens OX=9606 GN=CAPNS1 PE=1 SV=1	CPNS1_HUMAN	CAPNS1	28 kDa	21	9	11	4	12	9	8	7	5	8	6	2	5	3	4	5	4	11	10	10	15	10	8	4	7	10	18	13	6	6	5	3	13	9	8	9	9	30	8	14	9	13	26	14	5	4	14
267	Keratin, type I cytoskeletal 16 OS=Homo sapiens OX=9606 GN=KRT16 PE=1 SV=4	K1C16_HUMAN	KRT16	51 kDa	6	14	2	4	9	7	2	9	7	4	31	11	46	15	0	27	11	7	6	8	9	3	3	20	3	26	6	2	2	4	2	7	2	2	2	29	5	11	29	1	5	8	3	6	0	2	3
268	GMP reductase 1 OS=Homo sapiens OX=9606 GN=GMPR PE=1 SV=1	GMPR1_HUMAN	GMPR	37 kDa	23	10	7	5	13	10	13	5	7	9	2	5	9	5	3	6	11	17	13	9	17	13	8	7		11	34	10	3	8	5	6	11	6	6	4	8	29	14	8	7	10	27	10		7	8
269	RuvB-like 2 OS=Homo sapiens OX=9606 GN=RUVBL2 PE=1 SV=3	RUVB2_HUMAN	RUVBL2	51 kDa	17	8		0	6	7	10	0	1	8		5	7	4	1	2	13	10	15	1	4	11	16	4	1	17	35	17	0	6	6	4	22	6	10	5	10	39	26	9	10	10	26	22	4	4	25
270	Epoxide hydrolase 1 OS=Homo sapiens OX=9606 GN=EPHX1 PE=1 SV=1	HYEP_HUMAN	EPHX1	53 kDa	3	8	8	8	7	5	12	5	9	8	7	10	9	12	8	9	9	11	13	9	7	9	6	14	4	5	5	15	7	7	10	7	7	8	5	5	5	8	10	5	11	16	7	4	13	10	8
271	Phosphatidylinositol-binding clathrin assembly protein OS=Homo sapiens OX=9606 GN=PICALM PE=1 SV=2	PICAL_HUMAN	PICALM	71 kDa	12	15	11	14	8	13	12	12	12	8	11	11	11	6	7	10	12	11	11	10	10	16	12	14	16	5	5	15	8	11	9	8	9	11	6	12	6	5	8	7	6	9	6	8	8	12	9
272	Hsc70-interacting protein OS=Homo sapiens OX=9606 GN=ST13 PE=1 SV=2	F10A1_HUMAN	ST13	41 kDa	11	7	6	6	10	11	9	5	7	10	6	4	10	11	7	7	12	14	12	8	18	13	13	7	3	19	16	8	6	5	8	6	10	8	9	8	9	28	12	6	4	4	19	15	8	9	12
273	Protein diaphanous homolog 1 OS=Homo sapiens OX=9606 GN=DIAPH1 PE=1 SV=2	DIAP1_HUMAN	DIAPH1	141 kDa	35	12	10	4	11	12	20	9	2	13	4	3	8	8	3	2	15	19	12	3	27	16	23	2		7	17	13	2	3	3	3	11	13	5	10	4	20	7	3	4	5	18	9	2	3	5
274	WD repeat-containing protein 91 OS=Homo sapiens OX=9606 GN=WDR91 PE=1 SV=2	WDR91_HUMAN	WDR91	83 kDa	12	9	6	1	6	5	10	5	2	7	1	1	1	3	2	3	14	12	10	0	19	11	18	2		6	18	7	3	2	10	3	20	13	6	17	20	28	20	5	11	14	23	14	2	1	20
275	CD59 glycoprotein OS=Homo sapiens OX=9606 GN=CD59 PE=1 SV=1	CD59_HUMAN	CD59	14 kDa	10	10	10	5	9	7	9	8	8	5	6	13	12	11	13	11	13	14	14	10	17	9	12	13	8	13	10	7	6	7	7	9	6	6	8	6	8	4	12	11	8	9	8	8	12	14	6
276	Phospholipid hydroperoxide glutathione peroxidase OS=Homo sapiens OX=9606 GN=GPX4 PE=1 SV=3	GPX4_HUMAN	GPX4	22 kDa	4	4	4	5	4	6	7	4	8	7	5	8	9	11	10	13	20	13	17	15	11	8	11	5	7	5	4	7	4	8	8	3	6	1	4	4	5	6	13	14	13	19	4	10	10	9	7
277	L-lactate dehydrogenase B chain OS=Homo sapiens OX=9606 GN=LDHB PE=1 SV=2	LDHB_HUMAN	LDHB	37 kDa	17	10	3	3	11	16	12	7	14	11	2	1	9	10	2	1	9	19	14	7	10	12	7	3	2	11	27	13	2	6	4	5	13	9	7	5	6	19	11	4	9	7	23	10	4	2	9
278	Lymphocyte function-associated antigen 3 OS=Homo sapiens OX=9606 GN=CD58 PE=1 SV=1	LFA3_HUMAN	CD58	28 kDa	9	8	11	9	8	9	8	11	7	7	8	14	13	13	15	13	11	14	13	11	10	11	13	10	7	11	10	10	8	8	10	10	11	8	10	8	7	10	13	11	10	13	9	6	9	13	9
279	Fructosamine-3-kinase OS=Homo sapiens OX=9606 GN=FN3K PE=1 SV=1	FN3K_HUMAN	FN3K	35 kDa	4	6	3	10	7	8	7	7	14	9	9	11	9	17	11	14	12	15	18	13	14	8	10	11	7	15	10	7	9	8	6	15	7	7	4	5	4	13	17	10	12	8	10	20	12	19	16
280	26S proteasome non-ATPase regulatory subunit 8 OS=Homo sapiens OX=9606 GN=PSMD8 PE=1 SV=2	PSMD8_HUMAN	PSMD8	40 kDa	7	8	6	5	8	8	8	6	7	11	6	5	9	4	6	6	12	11	9	4	19	14	11	6	2	5	14	11	7	7	6	5	15	12	6	14	9	23	8	4	9	9	20	11	7	1	13
281	Ras-related protein Rab-18 OS=Homo sapiens OX=9606 GN=RAB18 PE=1 SV=1	RAB18_HUMAN	RAB18	23 kDa	9	9	16	10	12	14	4	11	11	14	15	6	5	8	7	5	6	7	6	8	7	7	10	9	10	5	9	8	7	10	14	15	14	15	15	13	7	12	10	7	8	9	4	7	9	6	14
282	Reticulon-4 OS=Homo sapiens OX=9606 GN=RTN4 PE=1 SV=2	RTN4_HUMAN	RTN4	130 kDa	4	2	2	2	3	3	4	2	4	3	4	8	7	9	6	7	8	9	9	5	6	6	6	7	8	9	15	4	9	10	21	18	20	6	12	6	8	19	19	11	13	8	13	8	11	8	25
283	Dynactin subunit 2 OS=Homo sapiens OX=9606 GN=DCTN2 PE=1 SV=4	DCTN2_HUMAN	DCTN2	44 kDa	10	19	5	7	11	9	14	8	12	22	5	1	12	8	2	3	13	13	9	3	10	15	11	7	1	5	7	9	3	2	9	2	6	4	9	6	15	11	14	1	3	10	12	10	4	4	5
284	ATP-binding cassette sub-family E member 1 OS=Homo sapiens OX=9606 GN=ABCE1 PE=1 SV=1	ABCE1_HUMAN	ABCE1	67 kDa	9	5	7	1	7	5	5	4	11	8	7	8	4	9	11	1	4	14	9	4	11	6	7	4	7	5	15	4	4	0	12	6	10	5	2	13	3	19	12	6	9	3	13	10	7	11	11
285	Alpha-soluble NSF attachment protein OS=Homo sapiens OX=9606 GN=NAPA PE=1 SV=3	SNAA_HUMAN	NAPA	33 kDa	4	3	3	2	2	1	0	5	3	1	1	15	11	11	10	6	12	8	15	8	12	12	9	9	6	11	4	6	4	2	8	7	1	2	1	13	9	20	22	12	13	15	18	18	14	22	17
286	Membrane transport protein XK OS=Homo sapiens OX=9606 GN=XK PE=1 SV=5	XK_HUMAN	XK	51 kDa	5	7	6	6	7	6	4	5	7	8	7	11	12	11	13	11	11	9	9	14	10	13	11	11	9	12	10	9	7	6	8	8	6	7	6	6	6	6	12	8	8	8	8	11	11	13	5
287	Exportin-7 OS=Homo sapiens OX=9606 GN=XPO7 PE=1 SV=3	XPO7_HUMAN	XPO7	124 kDa	24	9	4	1	8	17	10	2	3	8	1	2	3	7			5	16	12	4	21	11	13	2		10	35	14		3	2	1	15	4	4	2	4	47	17		4	2	33	12		1	19
288	Immunoglobulin lambda-like polypeptide 5 OS=Homo sapiens OX=9606 GN=IGLL5 PE=2 SV=2	IGLL5_HUMAN	IGLL5	23 kDa	12	8	3	10	9	3	11	19	10	11	10	9	14	10	18	8	6	7	5	6	3	6	3	7	6	5	10	7	7	12	7	5	5	5	9	7	11	6	6	19	5	8	8	7	14	7	2
289	Nucleosome assembly protein 1-like 1 OS=Homo sapiens OX=9606 GN=NAP1L1 PE=1 SV=1	NP1L1_HUMAN	NAP1L1	45 kDa	12	9	8	5	8	7	11	5	11	12	9	4	8	10	7	8	18	16	13	4	12	11	19	8	9	18	20	11	8	12	13	13	14	10	14	13	14	24	19	13	13	15	26	21	7	6	16
290	Multiple inositol polyphosphate phosphatase 1 OS=Homo sapiens OX=9606 GN=MINPP1 PE=1 SV=1	MINP1_HUMAN	MINPP1	55 kDa	13	5	12	10	9	7	10	11	6	10	10	17	14	13	17	10	13	8	10	13	14	7	13	10	7	10	4	16	3	1	5	7	4	5	3	5	5	7	5	2	4	15	5	7	3	13	9
291	Phospholipid-transporting ATPase IG OS=Homo sapiens OX=9606 GN=ATP11C PE=1 SV=3	AT11C_HUMAN	ATP11C	129 kDa	6	7	8	3	5	8	4	8	11	5	8	7	6	7	5	4	7	7	5	11	8	7	6	7	9	7	3	7	5	7	6	4	6	7	8	5	6	6	6	9	11	6	8	11	8	8	7
292	Serine/threonine-protein phosphatase 6 regulatory subunit 1 OS=Homo sapiens OX=9606 GN=PPP6R1 PE=1 SV=5	PP6R1_HUMAN	PPP6R1	97 kDa	9	8	5	1	6	3	4	3	3	6		2	6	6	3	3	11	15	9	3	14	9	13	5		7	19	6	2	3	9	4	10	6	4	17	8	25	18	4	13	5	26	13	1	1	13
293	Membrane-associated progesterone receptor component 2 OS=Homo sapiens OX=9606 GN=PGRMC2 PE=1 SV=1	PGRC2_HUMAN	PGRMC2	24 kDa	9	8	13	8	4	8	11	8	11	11	15	11	9	9	11	9	13	8	9	11	10	6	9	8	6	9	7	10	8	4	4	5	8	9	6	9	10	9	2	6	5	9	5	6	5	9	8
294	COP9 signalosome complex subunit 6 OS=Homo sapiens OX=9606 GN=COPS6 PE=1 SV=1	CSN6_HUMAN	COPS6	36 kDa	20	9	3	2	5	6	7	2	3	11	1	0	5	8	0	3	10	16	15	7	20	11	13	3		7	21	6	1	3	3	2	7	5	4	5	4	23	10	3	2	5	18	6	1	1	5
295	Protein DDI1 homolog 2 OS=Homo sapiens OX=9606 GN=DDI2 PE=1 SV=1	DDI2_HUMAN	DDI2	45 kDa	26	15	4	2	11	14	15	4	5	8	0	3	4	3		4	13	16	9	6	12	10	14	7	1	16	27	16	2	5	6	1	7	9	5	10	5	27	9	6	9	8	18	10	1	2	9
296	Gamma-aminobutyric acid receptor-associated protein-like 2 OS=Homo sapiens OX=9606 GN=GABARAPL2 PE=1 SV=1	GBRL2_HUMAN	GABARAPL2	14 kDa	8	7	7	2	6	5	6	6	8	9	9	9	14	11	10	14	9	9	8	8	9	10	9	11	7	14	9	5	7	7	7	7	7	9	7	9	5	10	11	13	10	6	8	13	11	14	11
297	Vesicle-associated membrane protein 3 OS=Homo sapiens OX=9606 GN=VAMP3 PE=1 SV=3	VAMP3_HUMAN	VAMP3	11 kDa	9	9	10	9	11	9	8	9	12	12	8	8	9	9	8	7	8	7	12	10	8	8	7	10	12	6	9	9	9	9	12	9	10	12	9	13	8	11	9	13	7	5	8	9	8	14	11
298	Very-long-chain (3R)-3-hydroxyacyl-CoA dehydratase 3 OS=Homo sapiens OX=9606 GN=HACD3 PE=1 SV=2	HACD3_HUMAN	HACD3	43 kDa	6	7	8	4	4	2	5	5	6	4	8	9	8	7	8	6	10	7	6	10	9	7	8	9	9	10	9	13	6	3	7	5	8	5	5	6	4	8	7	8	8	14	8	10	8	9	6
299	Keratin, type I cytoskeletal 14 OS=Homo sapiens OX=9606 GN=KRT14 PE=1 SV=4	K1C14_HUMAN	KRT14	52 kDa	12	14	5	10	10	18	7	17	10	8	23	15	29	14	1	17	14	9	10	11	11	9	9	15	4	27	9	3	5	6	10	7	5	2	4	18	10	12	13	2	10	15	7	11	0	9	4
300	ATP-dependent RNA helicase A OS=Homo sapiens OX=9606 GN=DHX9 PE=1 SV=4	DHX9_HUMAN	DHX9	141 kDa	6	4	2		2	7	1	1		5	3	8	3	6	7	3	9	13	10	8	14	7	9	3		9	29	2		2	4	1	15	4	6	4	4	32	26	7	7	1	28	11	6	10	18
301	Serine/threonine-protein phosphatase 6 catalytic subunit OS=Homo sapiens OX=9606 GN=PPP6C PE=1 SV=1	PPP6_HUMAN	PPP6C	35 kDa	8	7	7	5	7	11	8	5	8	7	3	4	8	8	6	5	9	14	9	5	14	15	9	5	1	11	18	11	5	3	7	5	14	12	5	13	7	14	13	4	10	11	19	14	3	2	14
302	RNA 3'-terminal phosphate cyclase OS=Homo sapiens OX=9606 GN=RTCA PE=1 SV=1	RTCA_HUMAN	RTCA	39 kDa	5	3	3	1	5	8	3	4	13	6	4	9	10	11	8	10	7	11	6	16	11	9	9	10	5	9	11	11	2	7	5	5	5	7	4	10	7	14	13	11	11	8	8	5	5	13	7
303	Phospholipid scramblase 1 OS=Homo sapiens OX=9606 GN=PLSCR1 PE=1 SV=1	PLS1_HUMAN	PLSCR1	35 kDa	14	5	9	11	9	11	7	9	10	8	8	11	7	7	9	8	9	11	6	8	11	5	11	8	10	7	7	4	6	7	8	4	8	9	7	11	6	5	3	9	9	9	5	4	9	8	9
304	RuvB-like 1 OS=Homo sapiens OX=9606 GN=RUVBL1 PE=1 SV=1	RUVB1_HUMAN	RUVBL1	50 kDa	11	8	3	1	8	6	9	2	2	5	1	7	7	4	3	3	8	7	10	4	6	6	8	5	1	9	16	9	3	5	5	5	11	5	5	4	6	32	16	7	4	4	22	14	5	4	17
305	Fructose-bisphosphate aldolase C OS=Homo sapiens OX=9606 GN=ALDOC PE=1 SV=2	ALDOC_HUMAN	ALDOC	39 kDa	22	28	19	24	18	29	25	29	27	27	27	29	20	31	27	25	34	25	24	25	30	26	27	23	23	32	30	33	25	29	37	22	27	26	25	25	23	32	33	34	33	34	30	42	37	31	31
306	Pyrroline-5-carboxylate reductase 3 OS=Homo sapiens OX=9606 GN=PYCR3 PE=1 SV=3	P5CR3_HUMAN	PYCR3	29 kDa	6	2	6	6	1		3	2	4	2	2	11	13	13	12	15	15	8	2	13	23	10	14	10	1		12	11	3		7	2	5	6	1	11	9	14	18	9	13	15	16		9	16	10
307	DNA damage-binding protein 1 OS=Homo sapiens OX=9606 GN=DDB1 PE=1 SV=1	DDB1_HUMAN	DDB1	127 kDa	19	3	3		8	9	10	2	1	7		1	0	0		1	3	20	8		21	14	16	4		9	23	11			1	1	6	6	1	4		24	10	1	0	7	24	5			5
308	Transcriptional activator protein Pur-alpha OS=Homo sapiens OX=9606 GN=PURA PE=1 SV=2	PURA_HUMAN	PURA	35 kDa	9	9	6		7	9	10	8	5	7	5	5	6	5	6	5	9	8	8	3	13	9	8	7	3	12	16	8	6	6	9	7	11	8	7	7	14	16	17	6	10	7	18	10	5	3	8
309	Carnitine O-palmitoyltransferase 1, liver isoform OS=Homo sapiens OX=9606 GN=CPT1A PE=1 SV=2	CPT1A_HUMAN	CPT1A	88 kDa	4	6	5	9	5	6	5	8	5	5	6	13	10	7	14	9	14	10	10	15	10	9	10	13	14	6	6	11	1	4	8	3	4	3	9	4	6	5	5	5	8	12	7	5	5	13	4
310	Proteasome subunit beta type-6 OS=Homo sapiens OX=9606 GN=PSMB6 PE=1 SV=4	PSB6_HUMAN	PSMB6	25 kDa	7	12	5	4	3	3	5	4	4	6	4	1	11	2	3	1	7	8	7	3	8	5	9	4	2	9	7	7	2	5	4	5	9	8	6	4	10	16	9	4	5	6	11	6	4	1	14

311	MAGUK p55 subfamily member 6 OS=Homo sapiens OX=9606 GN=MPP6 PE=1 SV=2	MPP6_HUMAN	MPP6	61 kDa	6	11	13	2	9	14	7	10	11	3	6	2	4	6	3	2	3	6	10		8	6	5	10	5	8	7	8	4	11	7	9	3	5	6	2	2	5	3	4	4	5	2	7	1	9	6
312	Leucine-rich repeat-containing protein 57 OS=Homo sapiens OX=9606 GN=LRRC57 PE=1 SV=1	LRC57_HUMAN	LRRC57	27 kDa	3	6	5	5	5	2	4	6	5	2	4	6	8	7	7	13	9	10	9	10	6	8	8	10	9	6	7	8	8	3	10	7	3	3	5	3	8	5	7	10	9	7	9	7	7	9	5
313	Syntaxin-4 OS=Homo sapiens OX=9606 GN=STX4 PE=1 SV=2	STX4_HUMAN	STX4	34 kDa	6	9	7	1	2	4	4	8	6	6	7	7	12	6	14	10	12	10	12	12	10	11	14	11	6	8	3	7	0	5	4	3	1	1	5	3	1	4	8	7	6	9	4	4	7	9	1
314	Annexin A11 OS=Homo sapiens OX=9606 GN=ANXA11 PE=1 SV=1	ANX11_HUMAN	ANXA11	54 kDa	9	12	11	8	8	7	11	11	8	6	4	5	8	9	5	4	5	7	7	8	10	6	10	4	5	6	5	8	5	6	4	10	5	7	8	9	8	6	4	10	6	6	4	7	3	6	9
315	Hydroxyacylglutathione hydrolase, mitochondrial OS=Homo sapiens OX=9606 GN=HAGH PE=1 SV=2	GLO2_HUMAN	HAGH	34 kDa	2	2	1	3	4	4	4	7	6	6	5	9	9	8	8	8	7	13	9	14	4	5	2	11	9	15	6	7	5	4	4	3	3	4	3	2	2	5	11	8	10	4	8	9	10	20	7
316	Glucosidase 2 subunit beta OS=Homo sapiens OX=9606 GN=PRKCSH PE=1 SV=2	GLU2B_HUMAN	PRKCSH	59 kDa	6	5	7	5	3	5	7	6	8	9	15	12	11	14	15	8	10	6	6	8	7	6	7	11	2	5	3	9	2	0	3	2	4	7	4	5	1	2	4	3	4	10	2	2	1	7	4
317	V-type proton ATPase subunit B, brain isoform OS=Homo sapiens OX=9606 GN=ATP6V1B2 PE=1 SV=3	VATB2_HUMAN	ATP6V1B2	57 kDa	24	8	5	1	11	11	10	3	6	5	1			1			5	10	6	1	11	6	11			9	25	13	0	3	3	3	11	9	3	5	3	25	10		4	3	20	7			10
318	Multidrug resistance-associated protein 5 OS=Homo sapiens OX=9606 GN=ABCC5 PE=1 SV=2	MRP5_HUMAN	ABCC5	161 kDa	8	7	10	7	10	9	6	9	11	10	4	1		9	8	2	4	5	10	8	2	2	8	6	5	11	12	4	7	3	6	11	11	11	7	12	5	9	9	5	7	11	6	18	6	8	12
319	14-3-3 protein zeta/delta OS=Homo sapiens OX=9606 GN=YWHAZ PE=1 SV=1	1433Z_HUMAN	YWHAZ	28 kDa	19	9	5	4	6	7	11	7	8	9	5	9	8	10	5	5	13	14	14	7	14	15	12	11	4	21	18	17	6	7	7	4	10	6	5	11	11	19	16	7	12	13	12	9	13	9	12
320	Transmembrane emp24 domain-containing protein 7 OS=Homo sapiens OX=9606 GN=TMED7 PE=1 SV=2	TMED7_HUMAN	TMED7	25 kDa	8	5	8	4	6	5	7	4	9	4	7	7	6	9	7	5	8	6	7	5	8	4	7	5	6	10	8	5	7	7	5	7	13	11	10	10	10	13	9	11	10	13	7	9	10	8	14
321	Leukocyte surface antigen CD47 OS=Homo sapiens OX=9606 GN=CD47 PE=1 SV=1	CD47_HUMAN	CD47	35 kDa	8	6	11	5	5	5	9	8	8	8	7	9	11	9	9	9	9	9	8	11	8	8	8	10	6	9	6	10	5	6	8	8	7	7	6	7	5	9	8	8	7	9	6	9	7	8	6
322	CDGSH iron-sulfur domain-containing protein 2 OS=Homo sapiens OX=9606 GN=CISD2 PE=1 SV=1	CISD2_HUMAN	CISD2	15 kDa	6	4	4	4	3	3	4	5	6	6	5	9	8	10	8	4	11	5	12	7	8	7	7	7	7	9	8	10	3	6	6	6	5	7	6	2	4	5	3	6	4	8	8	8	6	10	3
323	Reticulon-3 OS=Homo sapiens OX=9606 GN=RTN3 PE=1 SV=2	RTN3_HUMAN	RTN3	113 kDa	11	6	8	7	9	6	11	5	7	8	11	7	8	4	4	7	9	4	9	7	8	6	9	8	8	9	5	5	10	8	4	14	8	10	9	7	9	6	4	5	9	7	3	8	10	7	9
324	Eukaryotic translation initiation factor 2 subunit 3 OS=Homo sapiens OX=9606 GN=EIF2S3 PE=1 SV=3	IF2G_HUMAN	EIF2S3	51 kDa	7	9	6	2	7	9	7	6	6	6	4	2	5	6	2	2	10	13	11	4	13	13	9	2	2	5	14	7	4	4	7	6	7	7	6	8	5	14	12	1	6	3	14	9	6	4	12
325	Thioredoxin domain-containing protein 5 OS=Homo sapiens OX=9606 GN=TXNDC5 PE=1 SV=2	TXND5_HUMAN	TXNDC5	48 kDa	7	7	4	5	3	3	2	7	5	6	11	8	12	9	9	10	7	6	7	4	10	9	8	8	4	6	4	7	3	6	5	3	6	6	4	4	6	5	5	6	9	7	5	7	6	8	4
326	EH domain-binding protein 1-like protein 1 OS=Homo sapiens OX=9606 GN=EHBP1L1 PE=1 SV=2	EH1L1_HUMAN	EHBP1L1	162 kDa	9	7	5	2	5	6	6	3	3	5	6	3	4	3	4	4	7	9	4	2	9	6	8	3	8	9	10	10	6	2	6	4	9	8	1	11	5	13	16	5	6	10	12	14	9	5	6
327	Hemoglobin subunit gamma-2 OS=Homo sapiens OX=9606 GN=HBG2 PE=1 SV=2	HBG2_HUMAN	HBG2	16 kDa	13			9		42		11	15	12	8	10	11	15	11	6		39	8	10	10	9	12	25		24	13	15	15	17	12	17	13	12	25	14	17	18	18	17	13	9	13	15	11	28	15
328	Extended synaptotagmin-1 OS=Homo sapiens OX=9606 GN=ESYT1 PE=1 SV=1	ESYT1_HUMAN	ESYT1	123 kDa	5	13	7	6	5	8	8	18	8	9	11	6	12	7	12	5	10	4	4	4	13	10	6	13	6	3	5	10	1	5	2	5	3	3	7	6	8	6	4	4	5	9	3	1	3	12	6
329	Endoplasmic reticulum resident protein 29 OS=Homo sapiens OX=9606 GN=ERP29 PE=1 SV=4	ERP29_HUMAN	ERP29	29 kDa	10	9	11	6	5	5	10	13	7	8	8	11	8	8	10	7	11	5	8	8	10	4	7	8	1	2	2	6	1	2	2	3	4	4	1	4	3	3	3	2	1	7	1	5	4	7	2
330	Staphylococcal nuclease domain-containing protein 1 OS=Homo sapiens OX=9606 GN=SND1 PE=1 SV=1	SND1_HUMAN	SND1	102 kDa	1	1	2						0	1	1					0	1	0			1			3	7	24	10	1	11	5	18	19	12	2	11		12	11	24	10	19	1	19	23	9	3	21
331	Ecto-ADP-ribosyltransferase 4 OS=Homo sapiens OX=9606 GN=ART4 PE=2 SV=2	NAR4_HUMAN	ART4	36 kDa	4	7	7	4	3	5	5	7	5	6	5	4	3	4	4	4	3	4	2	4	2	3	4	6	6	6	5	3	3	3	5	4	6	3	4	4	4	5	4	5	6	5	4	5	7	6	6
332	Calcium and integrin-binding protein 1 OS=Homo sapiens OX=9606 GN=CIB1 PE=1 SV=4	CIB1_HUMAN	CIB1	22 kDa	10	4	4	4	3	1	5	4	6	7	4	10	9	8	5	7	9	5	7	9	7	8	8	9	4	12	4	6	4	5	4	7	5	5	5	3	3	4	5	7	7	6	6	8	7	9	4
333	Phenylalanine--tRNA ligase beta subunit OS=Homo sapiens OX=9606 GN=FARSB PE=1 SV=3	SYFB_HUMAN	FARSB	66 kDa		5	1		8		5	5	5	12	0	3	9	15	2		8		8	1	3	5		1	10	22	9		13	2	26	10	11		8		10	7	25	3	20		6	17	10	2	11
334	Solute carrier family 2, facilitated glucose transporter member 4 OS=Homo sapiens OX=9606 GN=SLC2A4 PE=1 SV=1	GLUT4_HUMAN	SLC2A4	55 kDa	8	15	7	11	11	5	11	7	5	6	6	10	8	5	9	5	13	8	10	11	6	13	5	9	10	7	4	9	2	5	3	1	1	7	2	6	2	2	4	8	2	9	5	5	8	7	1
335	Malignant T-cell-amplified sequence 1 OS=Homo sapiens OX=9606 GN=MCTS1 PE=1 SV=1	MCTS1_HUMAN	MCTS1	21 kDa	1	1	3	2		6	3		0			5	6	1		4	7	6	2	4	10	7	6	0	4	5	13	9	5	9	9	4	7	5	4	1	6	12	10	7	14	6	10	7	8	4	10
336	Vesicle-associated membrane protein-associated protein B/C OS=Homo sapiens OX=9606 GN=VAPB PE=1 SV=3	VAPB_HUMAN	VAPB	27 kDa	5	4	4	4	2	4	2	3	3	4	5	5	5	7	8	10	9	8	7	7	12	6	8	7	4	7	4	7	3	5	6	4	2	3	6	6	3	6	7	4	5	7	8	9	6	10	3
337	COP9 signalosome complex subunit 1 OS=Homo sapiens OX=9606 GN=GPS1 PE=1 SV=4	CSN1_HUMAN	GPS1	56 kDa	11	7	4	0	5	9	6	2	2	6		0	2	6	1	1	7	9	5	2	17	9	9	1		7	19	9		3	1	3	14	2	4	4	1	28	6		1	6	20	5			7
338	Protein ERGIC-53 OS=Homo sapiens OX=9606 GN=LMAN1 PE=1 SV=2	LMAN1_HUMAN	LMAN1	58 kDa	6	6	11	5	3	2	9	5	4	4	4	9	3	3	9	5	6	4	5	9	9	3	7	3	5	11	5	4	2	4	7	3	7	7	4	3	2	6	8	6	6	4	8	12	11	7	7
339	Rabankyrin-5 OS=Homo sapiens OX=9606 GN=ANKFY1 PE=1 SV=2	ANFY1_HUMAN	ANKFY1	128 kDa	12	7	3	1	6	7	3	2	3	4	0	2	4	4	1	4	7	11	10	1	13	12	10	3		10	21	11	1	5	5	1	9	9	7	5	6	24	10	0	2	4	20	11		0	3
340	Serine/threonine-protein phosphatase 2A 65 kDa regulatory subunit A alpha isoform OS=Homo sapiens OX=9606 GN=PPP2R1A PE=1 SV=4	2AAA_HUMAN	PPP2R1A	65 kDa	32	7			8	11	7	2	4	10	0			1	2		1	18	13	4	9	9	8	3		7	36	7		2		3	8	5	4	4		38	13		1		28	5		2	7
341	AP-2 complex subunit sigma OS=Homo sapiens OX=9606 GN=AP2S1 PE=1 SV=2	AP2S1_HUMAN	AP2S1	17 kDa	2	3	3	2	1	4	3	1	1	3	2	8	6	7	3	6	10	10	5	6	6	7	9	3	1	13	11	3	3	5	7	3	6	8	9	3	7	21	13	7	7	7	10	11	7	8	5
342	Phosphoglycerate kinase 1 OS=Homo sapiens OX=9606 GN=PGK1 PE=1 SV=3	PGK1_HUMAN	PGK1	45 kDa	1			1		2	5	5	3	0	1	15	6	19	9	13	11	12	5	12	16	9	9	4		7	2	2		4	4	2				5	4	12	9	8	6	4	7	10	11	25	9
343	Diacylglycerol kinase alpha OS=Homo sapiens OX=9606 GN=DGKA PE=1 SV=3	DGKA_HUMAN	DGKA	83 kDa	8	3	2		0	2	2		2	7	1	8	8	16	14	10	11	9	8	8	18	14	15	0		9	3	1		1	1		0	1	0	13	0	16	3	3	4	6	7	9	8	5	0
344	Ubiquitin carboxyl-terminal hydrolase 5 OS=Homo sapiens OX=9606 GN=USP5 PE=1 SV=2	UBP5_HUMAN	USP5	96 kDa	13	4	2	2	9	11	5	3	2	10			2	3			3	14	7	1	14	8	8	2	1	9	26	14		4	2	1	8	6	3	1	2	33	8	3	5	2	19	6	1		5
345	Transmembrane emp24 domain-containing protein 2 OS=Homo sapiens OX=9606 GN=TMED2 PE=1 SV=1	TMED2_HUMAN	TMED2	23 kDa	9	7	11	3	6	4	11	2	6	5	5	5	4	5	6	2	4	1	6	4	4	4	2	10	3	8	9	5	5	7	9	6	7	9	9	9	6	10	8	9	10	10	5	9	10	10	6
346	DnaJ homolog subfamily B member 4 OS=Homo sapiens OX=9606 GN=DNAJB4 PE=1 SV=1	DNJB4_HUMAN	DNAJB4	38 kDa	2	4	4	5	4	9	7	2	2	1	7	10	5	2	9	9	8	9	5	4	8	6	6	4	7	1	4	7	2	7	4	2	3	7	3	5	3	8	8	10	10	8	4	1	4	9	8
347	F-box only protein 7 OS=Homo sapiens OX=9606 GN=FBXO7 PE=1 SV=1	FBX7_HUMAN	FBXO7	59 kDa	7	5	3	1	6	8	6	4	4	5	2	6	2	7	1	3	5	11	7	3	9	8	9	8	3	10	13	11		4	3	2	4	7	5	5	2	15	10	1	4	3	12	6	2	4	3
348	Syntaxin-binding protein 2 OS=Homo sapiens OX=9606 GN=STXBP2 PE=1 SV=2	STXB2_HUMAN	STXBP2	66 kDa	6	6	7	7	6	2	7	8	4	5	4	9	6	7	7	7	14	6	6	5	7	8	6	9	4	6	4	6	2	5	7	2	4	2	1	4	2	3	5	3	3	5	2	4	5	10	6
349	V-type proton ATPase 116 kDa subunit a isoform 1 OS=Homo sapiens OX=9606 GN=ATP6V0A1 PE=1 SV=3	VPP1_HUMAN	ATP6V0A1	96 kDa	5	3	3	6	2	8	3	5	1	3	9	11	4	9	9	1	13	11	6	17	7	10	10	11	7		1	2		0	1		4	7		10	3	5	2	1		3	5	3	6	9	
350	MAP/microtubule affinity-regulating kinase 3 OS=Homo sapiens OX=9606 GN=MARK3 PE=1 SV=5	MARK3_HUMAN	MARK3	84 kDa	3	8	5	3	2	6	2	1	3	4	6	5	7	1	8	6	13	5	9	9	3	10	5	7	7	10	8	5	1	5	10	5	9	6	3	2	1	5	10	6	9	7	8	5	3	10	4
351	Calcium-binding protein 39 OS=Homo sapiens OX=9606 GN=CAB39 PE=1 SV=1	CAB39_HUMAN	CAB39	40 kDa	3	5	4	7	3	7	2	6	5	5	6	4	1	3	6	3	9	8	4	6	4	5	6	9	6	3	4	7	4	3	8	3	4	3	2	4	2	3	10	9	6	7	4	5	3	2	4
352	Ras-related protein Rab-1B OS=Homo sapiens OX=9606 GN=RAB1B PE=1 SV=1	RAB1B_HUMAN	RAB1B	22 kDa	10	11	10	10	9	12	8	10	9	8	9	16	13	15	11	13	11	12	11	11	17	19	15	13	10	12	19	13	8	9	16	11	14	13	14	12	8	15	14	15	15	13	14	14	22	15	13
353	UV excision repair protein RAD23 homolog A OS=Homo sapiens OX=9606 GN=RAD23A PE=1 SV=1	RD23A_HUMAN	RAD23A	40 kDa	24	3	1		12	13	12	3	3	5	1	1	1	4		1	2	21	5	1	9	5	6	1	1	5	30	15	2	2	1	4	4	4	4	4	6	22	6	1	4	4	15	3	2	1	3
354	Heat shock protein beta-1 OS=Homo sapiens OX=9606 GN=HSPB1 PE=1 SV=2	HSPB1_HUMAN	HSPB1	23 kDa	2	1	1		6	5	4							1	1	1	3	3	8		2	3	2			10	30	2	5		7	1	25	5	1	5		40	18	1	10		36	8	6		6
355	Transmembrane protein 56 OS=Homo sapiens OX=9606 GN=TMEM56 PE=1 SV=1	TMM56_HUMAN	TMEM56	30 kDa	3	4	2	4	2	5	3	3	4	6	3	7	6	5	7	6	5	5	6	6	7	6	5	4	5	5	3	5	3	3	5	4	4	5	3	4	3	5	5	4	5	6	5	5	5	5	5
356	Adipocyte plasma membrane-associated protein OS=Homo sapiens OX=9606 GN=APMAP PE=1 SV=2	APMAP_HUMAN	APMAP	46 kDa	5	2	2	4	2	3	3	1	5	4	8	7	4	7	8	1	7	0	5	6	2	5	7	9	4	11	5	7	2	3	6	4	8	7	3	3	4	6	8	8	8	7	6	5	5	7	1
357	Serine hydroxymethyltransferase, cytosolic OS=Homo sapiens OX=9606 GN=SHMT1 PE=1 SV=1	GLYC_HUMAN	SHMT1	53 kDa	5	12	3	8	2	16	5	5	7	3	2	1	8	9	2	5	2	10	3	8	4	7	8	9	4	13	9	0	4	8	13	19	7	10	8	5	4	6	7	3	3	6	5	5	3	1	17
358	Thioredoxin-like protein 1 OS=Homo sapiens OX=9606 GN=TXNL1 PE=1 SV=3	TXNL1_HUMAN	TXNL1	32 kDa	7	7	3	1	3	4	8	3	3	8	0	0	3	1			7	9	5	0	7	8	7	4		5	18	10	1	2	8	2	6	5	1	10	4	16	9	3	8	5	14	4		0	7
359	Adenylosuccinate lyase OS=Homo sapiens OX=9606 GN=ADSL PE=1 SV=2	PUR8_HUMAN	ADSL	55 kDa	9	2			3	2	0	1	3	6		6	5	15			3	18	13	4	18	13	1	2		6	19	8								3	1	20	8	1	0	0	15	10	8	3	9
360	Protein lifeguard 3 OS=Homo sapiens OX=9606 GN=TMBIM1 PE=1 SV=2	LFG3_HUMAN	TMBIM1	35 kDa	5	6	5	7	7	6	4	6	7	7	8	7	8	6	5	4	7	7	5	7	5	5	7	7	4	5	5	9	4	8	6	4	7	7	7	4	6	6	6	3	5	8	5	6	5	7	6
361	Perilipin-3 OS=Homo sapiens OX=9606 GN=PLIN3 PE=1 SV=3	PLIN3_HUMAN	PLIN3	47 kDa	3	7	4	1	1	0	3		1	3	3	8	8	9	8	15	9	12	13	3	11	10	10	4	4	7	3	5	3	0	1	1	1	2	5	7	5	7	2	5	9	12	3	9	12	7	3
362	Atypical chemokine receptor 1 OS=Homo sapiens OX=9606 GN=ACKR1 PE=1 SV=3	ACKR1_HUMAN	ACKR1	36 kDa	8	5	10	6	7	6	6	6	6	8	8	3	4	4	5	5	6	4	2	4	4	3	5	5	3	4	3	10	6	9	8	6	5	6	7	5	5	4	6	6	4	6	3	4	4	2	6
363	Nicastrin OS=Homo sapiens OX=9606 GN=NCSTN PE=1 SV=2	NICA_HUMAN	NCSTN	78 kDa	5	6	4	5	2	6	3	6	6	4	5	10	5	6	6	4	6	6	5	6	7	6	7	7	6	9	9	7	7	6	8	5	6	5	6	3	6	4	5	6	6	8	6	7	5	7	7
364	Serine/threonine-protein phosphatase 6 regulatory ankyrin repeat subunit A OS=Homo sapiens OX=9606 GN=ANKRD28 PE=1 SV=5	ANR28_HUMAN	ANKRD28	113 kDa	4	4	3	2	2	4	3	1	2	3	1	1	1	3	3	2	9	9	11	0	11	9	13	2	2	6	6	6	2	4	5	2	12	7	4	13	7	21	7	4	4	8	19	11	3		5
365	Transmembrane emp24 domain-containing protein 10 OS=Homo sapiens OX=9606 GN=TMED10 PE=1 SV=2	TMEDA_HUMAN	TMED10	25 kDa	5	3	3	7	6	3	3	4	7	4	3	8	9	11	7	4	11	7	7	10	9	4	6	11	8	7	6	5	4	5	8	4	6	7	3	5	6	5	8	7	5	6	4	6	5	10	4
366	Serine/threonine-protein kinase OSR1 OS=Homo sapiens OX=9606 GN=OXSR1 PE=1 SV=1	OXSR1_HUMAN	OXSR1	58 kDa	4	3	1		2	5	1	1	6	2	2	5	4	6	4	1	9	9	7	4	7	16	6	2	1	3	9	7	2	2	2	2	4	7		12	3	20	11	2	5	8	11	6	3	5	9
367	Phenylalanine--tRNA ligase alpha subunit OS=Homo sapiens OX=9606 GN=FARSA PE=1 SV=3	SYFA_HUMAN	FARSA	58 kDa	1	5	1		1		0	1	6	5	3	3	11	9	4	1	4		8	1	4	7		1	7	13	9	1	7	4	15	6	5		8		5	6	22	6	20		9	19	11	2	9
368	Interferon-induced protein with tetratricopeptide repeats 5 OS=Homo sapiens OX=9606 GN=IFIT5 PE=1 SV=1	IFIT5_HUMAN	IFIT5	56 kDa	2	3	3	5	5	2	6	5	4	7	6	10	6	7	10	12	13	6	5	9	4	7	4	9	7	7	3	5	4	5	5	5	4	1	1	5		4	9	7	3	5	2	8	7	8	1
369	Solute carrier family 43 member 3 OS=Homo sapiens OX=9606 GN=SLC43A3 PE=1 SV=2	S43A3_HUMAN	SLC43A3	55 kDa	3	4	4	5	2	3	3	5	3	3	6	2	3	5	6	4	3	3	3	6	5	3	5	7	4	5	4	3	2	5	3	2	4	4	2	4	2	2	3	2	3	2	2	2	1	5	3
370	PRA1 family protein 3 OS=Homo sapiens OX=9606 GN=ARL6IP5 PE=1 SV=1	PRAF3_HUMAN	ARL6IP5	22 kDa	5	6	8	2	6	5	4	7	2	3	5	5	4	6	7	7	7	5	7	4	8	5	8	7	6	7	5	8	4	5	4	6	7	5	5	8	3	6	7	6	7	9	5	6	3	7	9
371	Saccharopine dehydrogenase-like oxidoreductase OS=Homo sapiens OX=9606 GN=SCCPDH PE=1 SV=1	SCPDL_HUMAN	SCCPDH	47 kDa	4	4	7	5	2	3	6	3	3	1	11	9	6	6	9	11	7	9	8	11	10	5	9	10	5	4	3	8	1	2	4	4	2	0	1	2	2	2	5	4	4	7	1	7	5	9	5
372	Guanine nucleotide-binding protein G(I)/G(S)/G(O) subunit gamma-5 OS=Homo sapiens OX=9606 GN=GNG5 PE=1 SV=3	GBG5_HUMAN	GNG5	7 kDa	6	5	6	7	7	7	5	4	7	4	7	4	2	4	4	2	5	8	2	2	3	6	4	8	6	6	7	5	5	7	6	5	6	5	4	7	4	7	7	6	6	5	4	4	4	3	7
373	SUMO-conjugating enzyme UBC9 OS=Homo sapiens OX=9606 GN=UBE2I PE=1 SV=1	UBC9_HUMAN	UBE2I	18 kDa	1	1	1	2	3	4	3	1	7	1	6	6	4	4	6	8	6	4	3	3	4	4	3	4	2	3	5	4	2	4	9	6	3	4	2	2	6	7	8	13	3	9	7	9	3	3	3
374	Peptidyl-prolyl cis-trans isomerase NIMA-interacting 4 OS=Homo sapiens OX=9606 GN=PIN4 PE=1 SV=1	PIN4_HUMAN	PIN4	14 kDa	5	2	6	3	4	5	7	5	4	3	5	5	4	5	5	5	5	6	5	2	6	3	5	6	7	7	4	4	4	3	7	7	5	4	7	5	4	9	8	3	7	3	4	6	7	4	11
375	Band 4.1-like protein 2 OS=Homo sapiens OX=9606 GN=EPB41L2 PE=1 SV=1	E41L2_HUMAN	EPB41L2	113 kDa	1	4	6	5	6	1	11	3	4	10	13	12	6	0	1	1	13	1	4	2	0	1	0	3	7	0	2	2	7	11	1	1	8	12	3	7		6		7	5	1	5	1	2	2	
376	26S proteasome non-ATPase regulatory subunit 4 OS=Homo sapiens OX=9606 GN=PSMD4 PE=1 SV=1	PSMD4_HUMAN	PSMD4	41 kDa	6	5	1		3	5	8	4	6	9	1	5	6	3	5	4	12	8	4	3	13	9	8	6	4	11	12	11	1	6	8	4	7	7	5	12	9	15	9	7	6	9	14	6	4	3	5
377	Large neutral amino acids transporter small subunit 3 OS=Homo sapiens OX=9606 GN=SLC43A1 PE=1 SV=1	LAT3_HUMAN	SLC43A1	61 kDa	5	9	5	6	7	5	3	5	4	8	6	6	11	7	9	3	10	7	8	11	4	7	8	6	4	9	5	1	2	5	7	3	6	3	5	2	5	3	4	3	1	1	5	2	4	8	8
378	Galectin-9 OS=Homo sapiens OX=9606 GN=LGALS9 PE=1 SV=2	LEG9_HUMAN	LGALS9	40 kDa	8	8	7	6	8	8	7	10	11	6	9	5	3	6	7	7	6	6	6	4	4	6	10	8	4	5	11	8	3		5	7	9	5	3	8	3	13	6	5	5	9	6	6		7	3
379	Metallo-beta-lactamase domain-containing protein 2 OS=Homo sapiens OX=9606 GN=MBLAC2 PE=1 SV=3	MBLC2_HUMAN	MBLAC2	31 kDa	3	6	4	3	3	4	3	2	5	5	5	8	6	7	4	7	8	6	5	4	6	5	7	8	7	6	7	8	4	5	6	5	5	4	4	3	4	2	5	9	3	5	4	5	6	4	6
380	Serine/threonine-protein kinase WNK1 OS=Homo sapiens OX=9606 GN=WNK1 PE=1 SV=2	WNK1_HUMAN	WNK1	251 kDa	4	11	1		3	3	2	1	1	3	0	2	3	5	3	3	7	9	9	1	7	11	10	3		1	11	4			3	1	3	2		5	2	13	6	2	4	9	13	6			5
381	Ras-related protein Rap-2a OS=Homo sapiens OX=9606 GN=RAP2A PE=1 SV=1	RAP2A_HUMAN	RAP2A	21 kDa	44	56	53	36	40	44	41	44	35	41	52	27	28	26	27	25	32	22	25	26	30	32	29	33	40	22	26	44	33	37	35	33	39	37	35	33	29	28	28	30	34	39	17	22	25	23	34
382	Surfeit locus protein 4 OS=Homo sapiens OX=9606 GN=SURF4 PE=1 SV=3	SURF4_HUMAN	SURF4	30 kDa	3	5	5	4	4	5	1	7	4	5	3	7	4	6	3	2	9	4	3	5	3	5	8	7	8	7	5	3	4	6	5	4	3	5	5	5	4	4	5	4	3	6	4	3	3	4	6
383	Purine nucleoside phosphorylase OS=Homo sapiens OX=9606 GN=PNP PE=1 SV=2	PNPH_HUMAN	PNP	32 kDa	14				8	12	9	0	1	2		2		0			4	14	7	3	9	5	2	2		7	20	12		0				0	0	0		17	9	1	2	2	11	4		5	1
384	Vesicle transport protein SEC20 OS=Homo sapiens OX=9606 GN=BNIP1 PE=1 SV=3	SEC20_HUMAN	BNIP1	26 kDa	3		4		1	1	2	3	3	2	5	5	6	7	5	5	4	3	2	4	4	3	9	7	4	8	7	7	2	4	9	4	6	5	4	5	3	6	9	5	6	8	5	8	4	6	2
385	14-3-3 protein gamma OS=Homo sapiens OX=9606 GN=YWHAG PE=1 SV=2	1433G_HUMAN	YWHAG	28 kDa	14	7	5	5	9	8	7	7	9	8	10	10	10	8	6		8	6	8	5	9	6	10	10	5	14	15	10	7	7	8	7	8	6	3	11	9	16	11	8	11	11	9	11	8	4	12
386	Synaptosomal-associated protein 23 OS=Homo sapiens OX=9606 GN=SNAP23 PE=1 SV=1	SNP23_HUMAN	SNAP23	23 kDa	5	6	3	2	4	5	4	4	5	2	3	5	5	3	8	5	9	6	5	13	7	5	4	3	5	6	3	2	6	4	4	4	3	5	6	2	6	6	5	3	5	6	6	5	5	9	3
387	E3 ubiquitin-protein ligase RNF123 OS=Homo sapiens OX=9606 GN=RNF123 PE=1 SV=1	RN123_HUMAN	RNF123	149 kDa	17	1	1	1	4	7	4		1	4	0	1	2	4			3	17	9	4	11	10	11			4	19	11					5	2		2		23	4	0			22	6	0		2
388	Transmembrane 9 superfamily member 3 OS=Homo sapiens OX=9606 GN=TM9SF3 PE=1 SV=2	TM9S3_HUMAN	TM9SF3	68 kDa	2		3	3	1	1	1		4	3	3	3	5	5	2	2	1	0	1	0	5	4	2	7	7	10	5	5	4	5	7	8	9	6	5	3	5	9	7	14	4	10	9	15	8	4	4
389	Phosphoribosyl pyrophosphate synthase-associated protein 1 OS=Homo sapiens OX=9606 GN=PRPSAP1 PE=1 SV=2	KPRA_HUMAN	PRPSAP1	39 kDa	12	7	7	5	8	10	8	9	5	9		5	6	8	6	7	8	13	8	5	10	10	11	4		7	21	10	1	1	5	4	9	8	2	14	7	25	13		9	11	17	9	5	2	10
390	Solute carrier family 12 member 7 OS=Homo sapiens OX=9606 GN=SLC12A7 PE=1 SV=3	S12A7_HUMAN	SLC12A7	119 kDa	3	5	3	5	6	4	3	2	5	3	4	5	5	8	6	1	1	6	7	6	4			5	1	9	6	5		4	13	11	2	1	6	3	2	3	11	7	2	5	3	7	1	6	8
391	NSFL1 cofactor p47 OS=Homo sapiens OX=9606 GN=NSFL1C PE=1 SV=2	NSF1C_HUMAN	NSFL1C	41 kDa	10	5	1	1	6	2	7	4	1	6				3	2	1	4	12	5	1	10	6	9	1		5	20	11		4	1		5	2		7	4	20	5	1	5	2	11	9	2	1	12
392	Obg-like ATPase 1 OS=Homo sapiens OX=9606 GN=OLA1 PE=1 SV=2	OLA1_HUMAN	OLA1	45 kDa	2	2	2	5	3	6	5	5	4	5	2	1	2	1	1	1	2	4	1	1	2	3	2	3	4	8	10	4	9	8	9	5	9	5	8	2	4	10	13	6	6	1	7	6	7	5	10
393	Cytoskeleton-associated protein 5 OS=Homo sapiens OX=9606 GN=CKAP5 PE=1 SV=3	CKAP5_HUMAN	CKAP5	226 kDa	3	5	3	2		4	3	3		1	3	1	1		1	2	2	3	1	2	2	1		2	2	4	10	3	5	7	10	6	12	4	6	3	7	9	14	6	4	3	10	4	5	4	7
394	Transmembrane emp24 domain-containing protein 9 OS=Homo sapiens OX=9606 GN=TMED9 PE=1 SV=2	TMED9_HUMAN	TMED9	27 kDa	3	1	2		2	1	1	1	1	2	1	5	4	4	3	5	5	4	4	5	7	4	4	3	5	6	8	6	3	3	5	3	3	4	5	6	3	5	6	4	5	6	4	4	4	7	6
395	Transmembrane and coiled-coil domains protein 2 OS=Homo sapiens OX=9606 GN=TMCC2 PE=1 SV=3	TMCC2_HUMAN	TMCC2	77 kDa	6	4	6	4	1	4	7	4	5	5	4	4	3	2	5	4	9	2	0	4	4	4	4	9	7	5	3	8	0	2	16	6	4	2		2	0	3	19	4	4	4	3	4	8	5	6
396	Arf-GAP with SH3 domain, ANK repeat and PH domain-containing protein 1 OS=Homo sapiens OX=9606 GN=ASAP1 PE=1 SV=4	ASAP1_HUMAN	ASAP1	126 kDa	1		2		0					2		1	0	3	1	1	7	4	5	2	13	9	5	1		2	12	1		1	3	1	12	3		6	0	26	17	1	4	2	21	12	2		9
397	Programmed cell death protein 6 OS=Homo sapiens OX=9606 GN=PDCD6 PE=1 SV=1	PDCD6_HUMAN	PDCD6	22 kDa	2	3	4		3	0	6	5	1	2	1	5	4	8	5	5	9	7	9	7	6	3	9	4	1	1	1	13	1	3	3	3	2	3	3	0	4	2	6	8	7	10	4	5	5	11	3
398	Protein-glutamine gamma-glutamyltransferase 2 OS=Homo sapiens OX=9606 GN=TGM2 PE=1 SV=2	TGM2_HUMAN	TGM2	77 kDa	20	3	2		7	9	4		6	2			1	7			2	6	6	0	4	6	5			3	23	12				1	4	1	2	1	4	23	9	0	5	0	10	2	1		3
399	F-actin-capping protein subunit alpha-2 OS=Homo sapiens OX=9606 GN=CAPZA2 PE=1 SV=3	CAZA2_HUMAN	CAPZA2	33 kDa	4	6	2	2	1	4	4	9	3	2	1	5	4	2	5	6	5	7	3	8	4	4	6	4	1	3	4	3	1	1	6	3	3	3	1	4	4	3	8	13	6	3	3	4	3	5	6
400	Carbonic anhydrase 2 OS=Homo sapiens OX=9606 GN=CA2 PE=1 SV=2	CAH2_HUMAN	CA2	29 kDa	4		0	3	4	4	1	5	7	2	3	4	2	8	2	2	1	5	4	3	3	3	3	4	4	10	11	6	4	2	6	6	4	2	4	0	4	4	7	3	5	1	4	5	2	4	5
401	Protein VAC14 homolog OS=Homo sapiens OX=9606 GN=VAC14 PE=1 SV=1	VAC14_HUMAN	VAC14	88 kDa	5	8	1		1	1	5	1		2		3	5	6	2	2	8	5	5	1	15	11	8	1			10	5					5	3		7	5	15	7		3	3	11	2	3	2	6
402	Multifunctional protein ADE2 OS=Homo sapiens OX=9606 GN=PAICS PE=1 SV=3	PUR6_HUMAN	PAICS	47 kDa	18	2	1		6	14	5	3	4	5		2	0	5	0		3	15	4	3	14	7	11	0			12	6					2	3		1		18	3		0		13	0		0	1
403	RELT-like protein 1 OS=Homo sapiens OX=9606 GN=RELL1 PE=1 SV=1	RELL1_HUMAN	RELL1	29 kDa	6	4	9	4	3	5	3	8	8	7	7	4	5	5	4	5	3	4	2	4	4	3	5	8	6	2	5	6	4	4	6	6	7	6	5	6	5	6	3	6	3	4	2	2	2	3	4
404	COP9 signalosome complex subunit 8 OS=Homo sapiens OX=9606 GN=COPS8 PE=1 SV=1	CSN8_HUMAN	COPS8	23 kDa	7	6	1		4	6	5	1	2	6			1	3			3	9	3	2	9	9	7	3		3	8	6		4			8	8	4	2	2	12	7		0	4	11	3			3
405	Phosphatidylinositol 4-phosphate 5-kinase type-1 alpha OS=Homo sapiens OX=9606 GN=PIP5K1A PE=1 SV=1	PI51A_HUMAN	PIP5K1A	63 kDa	1	2	5	0	1	1	1	1	3	3	5	6	2	9	4	6	3	5	4	4	5	2	5	8	2	3	4	2	0	3	2	2	4	0	3	2	3	5	7	5	0	3	5	2	4	2	1
406	Cytochrome b5 type B OS=Homo sapiens OX=9606 GN=CYB5B PE=1 SV=3	CYB5B_HUMAN	CYB5B	17 kDa	2	2	4	2	3	3	2	2	5	6	7	6	3	5	6	7	4	5	4	6	4	6	5	6	6	8	5	8	1	6	4	3	5	4	3	4	1	4	4	3	4	8	5	3	6	9	4
407	Guanine nucleotide-binding protein subunit alpha-11 OS=Homo sapiens OX=9606 GN=GNA11 PE=1 SV=2	GNA11_HUMAN	GNA11	42 kDa	12	6	9	4	3	4	5	6	9	6	7	6	4	6	6	6	4	6	6	5	5	7	6	16	7	5	6	8	6	5	10	5	3	3	4	2	1	3	8	4	4	11	6	9	8	11	6
408	Nuclear receptor-binding protein OS=Homo sapiens OX=9606 GN=NRBP1 PE=1 SV=1	NRBP_HUMAN	NRBP1	60 kDa	8	5	3	2	7	6	2	4	2	4		1					1	4	2		4	1	3	2		3	8	7	0	2	3	1	5	5	2	6	4	13	6		3	3	7	6			5
409	Hypoxia up-regulated protein 1 OS=Homo sapiens OX=9606 GN=HYOU1 PE=1 SV=1	HYOU1_HUMAN	HYOU1	111 kDa	4		3	3	1	2	1	3	4	2	5	9	11	8	11	6	8	6	4	7	9	3	12	11	2	2	1	6		1	1			3	1	4		1	1		1	4	2			1	6
410	Protein THEM6 OS=Homo sapiens OX=9606 GN=THEM6 PE=1 SV=2	THEM6_HUMAN	THEM6	24 kDa	1	0	1	1	0	2	0	0	3	2	1	3	6	6	7	9	7	3	3	6	7	3	5	8	3	3	4	4		0	3	1	0	3	2	2	1	2	4	4	1	6	4	3	3	6	1
411	Proliferation-associated protein 2G4 OS=Homo sapiens OX=9606 GN=PA2G4 PE=1 SV=3	PA2G4_HUMAN	PA2G4	44 kDa	1	1	2		3	3	4	2	2	2	1	3	2	1	2	2	6		4		5	5	0		3	5	5	3	2	3	8	4	4	4	2	3	3	14	17	2	6	4	6	7	4	3	10
412	COP9 signalosome complex subunit 7b OS=Homo sapiens OX=9606 GN=COPS7B PE=1 SV=1	CSN7B_HUMAN	COPS7B	30 kDa	9	4	4	1	6	4	6	4	1	5	1		2	3		3	5	10	7	2	11	8	10	2		5	10	4		3	1	2	7	3	5	4	1	7	3			1	3	13	3		3
413	Thioredoxin-related transmembrane protein 1 OS=Homo sapiens OX=9606 GN=TMX1 PE=1 SV=1	TMX1_HUMAN	TMX1	32 kDa	7	3	5	2	1	3	4	2	4	4	7	4	3	10	4	4	6	5	3	7	5	3	5	6	5	3	4	6	0	1	2	2	2	2	3	4	4	4		1	3	6	4	2	3	4	2
414	V-type proton ATPase catalytic subunit A OS=Homo sapiens OX=9606 GN=ATP6V1A PE=1 SV=2	VATA_HUMAN	ATP6V1A	68 kDa	14	5	3		7	4	4	2		3				1			2	6	1	1	7	3	6	2		5	16	12		2	3	1	5	3	4	4	3	17	7		2	4	11	5	1		7
415	Nectin-1 OS=Homo sapiens OX=9606 GN=NECTIN1 PE=1 SV=3	NECT1_HUMAN	NECTIN1	57 kDa	7	7	7	4	2	4	4	8	10	4	4	6	5	11	7	4	4		3	7	6	4	1	5		1	2	4	2	2	4	2	0	3	2	1	1	2	4	1		5	1	1		5	2
416	Eukaryotic translation initiation factor 2 subunit 1 OS=Homo sapiens OX=9606 GN=EIF2S1 PE=1 SV=3	IF2A_HUMAN	EIF2S1	36 kDa	5	2	4		4	3	6	1	2	2		6	3	1	4	1	9	8	7	2	11	7	6	3		3	6	6		2	6	2	6	2		3	2	9	7	3	4	3	12	7	5	3	7
417	COP9 signalosome complex subunit 7a OS=Homo sapiens OX=9606 GN=COPS7A PE=1 SV=1	CSN7A_HUMAN	COPS7A	30 kDa	10	3	5	1	8	9	4	1	5	7		1	2	3	0	1	7	10	4	1	10	6	8	2		7	10	7		1	2	2	9	6	2	6	2	14	6	1		5	16	5	1		4
418	Complement C4-B OS=Homo sapiens OX=9606 GN=C4B PE=1 SV=2	CO4B_HUMAN	C4B	193 kDa	3	3	4	1	4	2	3	3	4	2		1	1	6	7	5	7	7	6	3	7	5	6	5	9	3	4	4	1	7	1	3	3	3	2	3	3	3	6	6	5	4	4	3	9	4	1
419	Myeloid-associated differentiation marker OS=Homo sapiens OX=9606 GN=MYADM PE=1 SV=2	MYADM_HUMAN	MYADM	35 kDa	7	7	9	1	4	6	9	3	4	4	5	4	3	5	4	6	5	7	7	5	3	3	5	3	5	2	2	6		2	4	2	3	4	5	2	1	4	4	3	3	6	1	3	5	5	2
420	Phospholipid scramblase 4 OS=Homo sapiens OX=9606 GN=PLSCR4 PE=1 SV=2	PLS4_HUMAN	PLSCR4	37 kDa	8	5	4	6	5	5	3	3	5	4	6	6	3	4	8	7	4	6	6	6	7	4	6	5	6	3	2	4	6	3	3	4	3	5	4	5	3	3	4	3	7	5	3	5	5	7	5
421	Microtubule-associated protein RP/EB family member 1 OS=Homo sapiens OX=9606 GN=MAPRE1 PE=1 SV=3	MARE1_HUMAN	MAPRE1	30 kDa	2	1	2	1	2	3	6	1	1	1	4	2	3	1	3	4	4	2	3	1	4	3	3	3	5	4	6	3	6	2	5	4	5	4	4	2	4	6	5	5	4	4	4	5	4	2	3
422	Retinoic acid receptor responder protein 2 OS=Homo sapiens OX=9606 GN=RARRES2 PE=1 SV=1	RARR2_HUMAN	RARRES2	19 kDa	3	3	4	3	5	3	5	4	3	4	1	2	3	5	5	5	4	4	7	5	5	5	6	5	3	4	2	3	3	6	1	5			3	3	2	5	5	4	3	5	3	4	2	8	
423	Choline transporter-like protein 2 OS=Homo sapiens OX=9606 GN=SLC44A2 PE=1 SV=3	CTL2_HUMAN	SLC44A2	80 kDa	8	2	4	2	2	3	7	1	5		1	6	4	7	3	5	7	1	3	2	4	3	5	3	1	5	3	4	2	3	1	5	1	0	3	0	3	1	2	4	4	6	2	7	3	6	2
424	Phosphatidate phosphatase LPIN2 OS=Homo sapiens OX=9606 GN=LPIN2 PE=1 SV=1	LPIN2_HUMAN	LPIN2	99 kDa	12	6	2		1	1	6	1	5	4		2	5	5	1	1	12	6	5	1	9	10	14			1	8	4					8	2		6		11	2		2	1	13	2			3
425	E3 ubiquitin-protein ligase KCMF1 OS=Homo sapiens OX=9606 GN=KCMF1 PE=1 SV=2	KCMF1_HUMAN	KCMF1	42 kDa	2	2	2	3	2	2	3	2	7	5	1	4	6	7	3	3	9	8	5	4	8	8	6	4	1	1	3	5	1	1	2	2	1	3	1	7	2	6	3	3	2	10	5	4	2	4	2
426	Clusterin OS=Homo sapiens OX=9606 GN=CLU PE=1 SV=1	CLUS_HUMAN	CLU	52 kDa	3	3	4	1	4	4	4	3	3	3	3	5	3	5	3	6	4	5	5	3	5	3	3	4	2	3	2	4	4	3	1	2	3	3	2	3	2	3	2	1	4	5	4	6	3	3	3
427	COP9 signalosome complex subunit 5 OS=Homo sapiens OX=9606 GN=COPS5 PE=1 SV=4	CSN5_HUMAN	COPS5	38 kDa	4	3	2		4	3	4	1	2	3			1	1			4	4	3		7	9	5			3	14	1		1	1	1	6	1	1	2	1	24	4		1	1	10	1			2
428	Methanethiol oxidase OS=Homo sapiens OX=9606 GN=SELENBP1 PE=1 SV=2	SBP1_HUMAN	SELENBP1	52 kDa	2	2	2	1	1	3	2	3	6	2	2	3	0	9	3	4	1	2	5	3	5	1				6	8	7	3	4	4	5	2	1	8	2	8	5	7	4	4	4	4	7	4	1	5
429	Immunoglobulin heavy constant alpha 1 OS=Homo sapiens OX=9606 GN=IGHA1 PE=1 SV=2	IGHA1_HUMAN	IGHA1	38 kDa	1				4	3	5	2	5	2		2	3	7	1	9	3	3	4	3	2	1	3	3	4	3	9	7	3	12	2	1	6	1	2	2	3	4	2	10	2	8	9	1	5	7	
430	Ras-related protein Rab-5B OS=Homo sapiens OX=9606 GN=RAB5B PE=1 SV=1	RAB5B_HUMAN	RAB5B	24 kDa	7	7	8	9	7	9	8		7	8	12	8	10	11	8	7	6	11	10	7	9	13	9	12	10	6	10	11	7	6	14	7	10	12	12	13	8	10	11	7	12	9	11	8	17	11	7
431	Neuroblastoma-amplified sequence OS=Homo sapiens OX=9606 GN=NBAS PE=1 SV=2	NBAS_HUMAN	NBAS	269 kDa	5	2	4	2		1	5	5	3	2	3	7	1	1	5	2	4	1		1		1	3	0	5	2	5	4	2	0	2	5	3	5	3	4	3	4	4	4	4	7	4	3	7	5	7
432	ADP-ribosylation factor 6 OS=Homo sapiens OX=9606 GN=ARF6 PE=1 SV=2	ARF6_HUMAN	ARF6	20 kDa	2	2	1			4	3		3	4	2	3	0	4	2	3	5	5	4		7	10	4	5	16	5	8	1	1	3	5	1	3	3	2	2	2	3	3	4	2	2	5	3	9	7	0
433	Ankyrin-3 OS=Homo sapiens OX=9606 GN=ANK3 PE=1 SV=3	ANK3_HUMAN	ANK3	480 kDa	20	18	19	21	21	17	16	21	21	19	26	27	21	33	31	25	25	19	31	31	19	23	25	17	26	23	22	25	19	20	25	25	25	22	27	20	23	22	30	26	27	29	23	25	27	23	25
434	CSC1-like protein 2 OS=Homo sapiens OX=9606 GN=TMEM63B PE=1 SV=1	CSCL2_HUMAN	TMEM63B	95 kDa	4	4	4	3	2	5	2	3	2	4	3	3	3	5	4		4	2	3	3	2	2	2	5	1		0	0		0			2	1	1	2	1	1				2				3	0
435	ADP-ribosylation factor 3 OS=Homo sapiens OX=9606 GN=ARF3 PE=1 SV=2	ARF3_HUMAN	ARF3	21 kDa	4	1	2			2	1	1	3	2	3	6	6	5	6	4	6	3	6	4	8	8	5	5	2	6	5	3	5	4	4	3	5	4	4	4	3	4	4	3	3	2	5	4	5	5	0
436	Aspartyl aminopeptidase OS=Homo sapiens OX=9606 GN=DNPEP PE=1 SV=1	DNPEP_HUMAN	DNPEP	52 kDa	13	5	4		5	2	4	1	0	5						0	3	8	7	1	8	7	3			3	12	6		1			7	5	4	3	3	14	5	0	4	4	10	3	1		8
437	Neuroplastin OS=Homo sapiens OX=9606 GN=NPTN PE=1 SV=2	NPTN_HUMAN	NPTN	44 kDa	5	3	3	2	5	4	2	3	3	3	3	4	4	5	3	1	2	3	5	4	3	2	2	5	2	3	3	2	2	4	0	3	5	2	3	3	2	4	4	5	3	4	4	4	4	2	4
438	Lysophospholipid acyltransferase 2 OS=Homo sapiens OX=9606 GN=MBOAT2 PE=2 SV=2	MBOA2_HUMAN	MBOAT2	60 kDa	1	3	1	0	2	1	2	2	1	2	4	4	3	2	2	1	2	4	1	5	2	1	3	3	3	2	1	4	1	2	4	1	1	2	2	0	2	2	1	1	0	2	3	2	2	3	1
439	Vesicle transport protein GOT1B OS=Homo sapiens OX=9606 GN=GOLT1B PE=1 SV=1	GOT1B_HUMAN	GOLT1B	15 kDa	3	4	3	3	2	4	3	4	4	4	4	5	4	5	3	3	3	5	3	3	4	3	6	4	3	3	4	4	3	5	6	5	5	5	6	4	4	3	4	4	4	4	2	4	3	5	4
440	E3 ubiquitin/ISG15 ligase TRIM25 OS=Homo sapiens OX=9606 GN=TRIM25 PE=1 SV=2	TRI25_HUMAN	TRIM25	71 kDa	1		1			2	3			0	1						4	1			4		1	5	3	5	12	6	2	7	8	4	5	5	4	7	6	10	8	8	6	3	9	4	3	1	8
441	Large proline-rich protein BAG6 OS=Homo sapiens OX=9606 GN=BAG6 PE=1 SV=2	BAG6_HUMAN	BAG6	119 kDa	2					1	1							1			0		1		2	1	1			2	7	2			7	2	11	3	1	5	5	12	15	0	3		18	13	1		9
442	GTPase NRas OS=Homo sapiens OX=9606 GN=NRAS PE=1 SV=1	RASN_HUMAN	NRAS	21 kDa	26	27	37	28	24	36	30	33	33	37	26	27	22	32	27		29	28	29	30	31	26	24	32	33	27	36	40	31	33	45	27	28	35	35	22	20	22	35	35	36	37	25	28	36	27	31
443	Solute carrier family 2, facilitated glucose transporter member 14 OS=Homo sapiens OX=9606 GN=SLC2A14 PE=2 SV=1	GTR14_HUMAN	SLC2A14	56 kDa	3	3	3	2	4	5	2	5	2	3	1	4	3	5	2	0	1	1	3	4		6	2	3	0	0	3	1	1	1	3	1	2	3	3	2	1	1	3	1	2	4	2	3	2	6	1
444	Pyridoxal phosphate homeostasis protein OS=Homo sapiens OX=9606 GN=PLPBP PE=1 SV=1	PLPHP_HUMAN	PLPBP	30 kDa							1	2				7	5	5	9	8	6	10	6	9	6	10	9			1		0								4	4	7	8	9	4	5	4	7	5	7	6
445	UTP--glucose-1-phosphate uridylyltransferase OS=Homo sapiens OX=9606 GN=UGP2 PE=1 SV=5	UGPA_HUMAN	UGP2	57 kDa	6	4	2		5	9	4		2	2			2	1			4	6	8	2	7	7	7	1		6	16	10		1	0		5	1	3	1	1	15	5		1	1	8	4	1		3
446	V-type proton ATPase subunit d 1 OS=Homo sapiens OX=9606 GN=ATP6V0D1 PE=1 SV=1	VA0D1_HUMAN	ATP6V0D1	40 kDa	2	2	2	1	1	3	2	1	2	2	2	6	3	5	9	0	10	9	3	8	7	7	8	8	5	2	3	2	2	1	2	2	2	4	3	4	1	4	4	3	1	4	1	0	6	5	2
447	Cullin-3 OS=Homo sapiens OX=9606 GN=CUL3 PE=1 SV=2	CUL3_HUMAN	CUL3	89 kDa	5	2	2	2	7	4	3	3	2	3	1						1	4	1		7	6	3		0		15	4	2	3	1	3	7	3	2	3	3	16	5	1	1		11	2	1	1	5
448	Receptor expression-enhancing protein 5 OS=Homo sapiens OX=9606 GN=REEP5 PE=1 SV=3	REEP5_HUMAN	REEP5	21 kDa	5	5	5	3	3	4	4	3	3		4	3	3	4	3	3	3	1	4	2	1	4	1	5	2	2	0	5	3	2	4	2	2	2	1	4	2	1	3	2	0	3	2	4	2	7	4
449	Atlastin-2 OS=Homo sapiens OX=9606 GN=ATL2 PE=1 SV=2	ATLA2_HUMAN	ATL2	66 kDa	1	4	5	1	1	0	1	3	5	3		2	4	4	2		2	2	3	4	3	2	1	2	3	8	8	6	3	2	7	5	6	5	6	4	2	5	6	5	4	3	6	8	3	2	4
450	Histone-arginine methyltransferase CARM1 OS=Homo sapiens OX=9606 GN=CARM1 PE=1 SV=3	CARM1_HUMAN	CARM1	66 kDa	11	3	1	0	6	6	5	0	2	4	0	2	1	0	1	2	4	11	4	2	6	5	6	2		4	13	6		2	2	2	4	2	2	3	1	15	4	2	1	2	16	3		1	1
451	Methylosome protein 50 OS=Homo sapiens OX=9606 GN=WDR77 PE=1 SV=1	MEP50_HUMAN	WDR77	37 kDa	5	4			1	2	3			0			1				6	6	2	1	6	3	6	3		5	8	3		2	1		6	3	2	0	1	10	3	1	3	2	12	4		0	6
452	GTP-binding nuclear protein Ran OS=Homo sapiens OX=9606 GN=RAN PE=1 SV=3	RAN_HUMAN	RAN	24 kDa	2	1	1		3	2	2	2	1	2	0	1	1	3	0		1	4	2	1	6	5	6		1	9	9	4		2	2	2	4	2	2	3	4	13	4	2	4	3	9	6	2	5	5
453	Dual specificity mitogen-activated protein kinase kinase 3 OS=Homo sapiens OX=9606 GN=MAP2K3 PE=1 SV=2	MP2K3_HUMAN	MAP2K3	39 kDa	1	1	2		2	3	1	3	2		1	6		4	2	4	8	5	3	2	2	5	0	1	2		5	2	0	0	1	1	2		0	3	1	11	15	6	8	4	13	8	4	4	12
454	Zinc finger ZZ-type and EF-hand domain-containing protein 1 OS=Homo sapiens OX=9606 GN=ZZEF1 PE=1 SV=6	ZZEF1_HUMAN	ZZEF1	331 kDa	0		0		1							0	1	3			2	1	2	1	9	5	2			2	8	5		3	0		5	2	0	10	1	20	4	0	1	4	17	4	2	2	3
455	Ubiquitin-like modifier-activating enzyme 1 OS=Homo sapiens OX=9606 GN=UBA1 PE=1 SV=3	UBA1_HUMAN	UBA1	118 kDa	12	5			8	5	9	2		2			0				1	5	1		4	6	1			6	19	8					2	0				22	8		1	0	10	3			1
456	Mannose-P-dolichol utilization defect 1 protein OS=Homo sapiens OX=9606 GN=MPDU1 PE=1 SV=2	MPU1_HUMAN	MPDU1	27 kDa	3	2	5	3	2	3	4	3	3	3	4	3	4	4	6	4	3	3	4	5	3	7	1	1	3	4	4	4	2	2	2	4	2	1	2	1	3	2	1	2	3	4	4	2	4	4	2
457	Cysteine-rich and transmembrane domain-containing protein 1 OS=Homo sapiens OX=9606 GN=CYSTM1 PE=1 SV=1	CYTM1_HUMAN	CYSTM1	11 kDa	4	4	3	4	5	3	5	1	3	4	4				2		2	0	1	2		1	0	0	4	0	6	7	2	5	7	2	5	5	3	3	3	4	1	5	4	9	1			0	3
458	Centromere/kinetochore protein zw10 homolog OS=Homo sapiens OX=9606 GN=ZW10 PE=1 SV=3	ZW10_HUMAN	ZW10	89 kDa	3	3	2				1	1			2	5	1	1	3	1	4		1	2	3	1	2	2	1	3	4	6	1	1	8	3	1	2	2	3	1	4	7		5	2	3	4	4	3	5
459	Protein disulfide-isomerase A4 OS=Homo sapiens OX=9606 GN=PDIA4 PE=1 SV=2	PDIA4_HUMAN	PDIA4	73 kDa	5	4	5	3	1	4	6	3	6	5	3	3	5	3	4	5	5		1	4	2	2	5	3	2	2	0	3	3	2	1	2	2	2	2	5		2	2	1	2	2	2	1	1	2	1
460	CD99 antigen OS=Homo sapiens OX=9606 GN=CD99 PE=1 SV=1	CD99_HUMAN	CD99	19 kDa	1	3	1	3	1	1	4	5	5	4	6	2	3	7	3	2	2	2	2	3	1	1	1	6	3	4	1	6	2	5	6	5	0	1	4	1	5	2	2	2	2	5	1	3	2	4	4
461	Synaptophysin-like protein 1 OS=Homo sapiens OX=9606 GN=SYPL1 PE=1 SV=1	SYPL1_HUMAN	SYPL1	29 kDa	2	2	2	1	2	2	2	2	5	6	5	4	4	3	5	2	4	3	6	1	5	2	4	2	2	5	5	2	4	4	3	2	3	3	4	4	3	6	6	4	2	3	2	1	3	2	3
462	Extended synaptotagmin-2 OS=Homo sapiens OX=9606 GN=ESYT2 PE=1 SV=1	ESYT2_HUMAN	ESYT2	102 kDa	3	5	6	1	2	1	7	6	3	3	4	4	6	5	8	4	4	1	4	4	4	6	3	3	2		0	3	0	1		2	3		3	3	0	3	2	1	0	4	1		2	6	1
463	Importin subunit alpha-7 OS=Homo sapiens OX=9606 GN=KPNA6 PE=1 SV=1	IMA7_HUMAN	KPNA6	60 kDa	9	6	1	1	4	6	3	4	2	4	0	2	1	5	1	1	3	8	7	1	7	6	8	1		1	9	5		1	2		3	3		1	1	10	3		2	3	6	1			3
464	Vasodilator-stimulated phosphoprotein OS=Homo sapiens OX=9606 GN=VASP PE=1 SV=3	VASP_HUMAN	VASP	40 kDa	7	2	4	1	1	3	6	2	3	2	4	3	2	2	1	0	3	1	2	3	1	3	3	5	1	2	2	4	4	2	1	2	1	6	4	2	2	2	1	1	2	5	2		0	4	2
465	Actin-related protein 10 OS=Homo sapiens OX=9606 GN=ACTR10 PE=1 SV=1	ARP10_HUMAN	ACTR10	46 kDa	3	4	2		3	2	4	2	2	4	2		0	3	4	2	5	4	4	2	5	3	4	4	0	1	2	1	0	2	3	1	1	2	2	3	5	5	5	2	2	8	2	2	2	0	2
466	Transcription elongation factor A protein 1 OS=Homo sapiens OX=9606 GN=TCEA1 PE=1 SV=2	TCEA1_HUMAN	TCEA1	34 kDa		1				0			1		1	3	2	6	6	7	7	6	4	5	7	4	6	2	1	3	2	2		3	4				1	5	3	7	7	10	4	6	3	5	2	7	4

467	Cathepsin E OS=Homo sapiens OX=9606 GN=CTSE PE=1 SV=3	CATE_HUMAN	CTSE	43 kDa	2	3	[illegible]
468	Thioredoxin-related transmembrane protein 4 OS=Homo sapiens OX=9606 GN=TMX4 PE=1 SV=1	TMX4_HUMAN	TMX4	39 kDa			[illegible]
469	Disco-interacting protein 2 homolog B OS=Homo sapiens OX=9606 GN=DIP2B PE=1 SV=3	DIP2B_HUMAN	DIP2B	171 kDa	2	1	[illegible]
470	Ferritin heavy chain OS=Homo sapiens OX=9606 GN=FTH1 PE=1 SV=2	FRIH_HUMAN	FTH1	21 kDa	1		[illegible]
471	Dolichyl-diphosphooligosaccharide--protein glycosyltransferase subunit 1 OS=Homo sapiens OX=9606 GN=RPN1 PE=1 SV=1	RPN1_HUMAN	RPN1	69 kDa	2	3	[illegible]
472	Serine/threonine-protein kinase STK11 OS=Homo sapiens OX=9606 GN=STK11 PE=1 SV=1	STK11_HUMAN	STK11	49 kDa	4	5	[illegible]
473	MAGUK p55 subfamily member 7 OS=Homo sapiens OX=9606 GN=MPP7 PE=1 SV=1	MPP7_HUMAN	MPP7	66 kDa	2	2	[illegible]
474	STE20-related kinase adapter protein beta OS=Homo sapiens OX=9606 GN=STRADB PE=1 SV=1	STRAB_HUMAN	STRADB	47 kDa	3	1	[illegible]
475	Large neutral amino acids transporter small subunit 4 OS=Homo sapiens OX=9606 GN=SLC43A2 PE=1 SV=1	LAT4_HUMAN	SLC43A2	63 kDa	0	2	[illegible]
476	Rab GDP dissociation inhibitor beta OS=Homo sapiens OX=9606 GN=GDI2 PE=1 SV=2	GDIB_HUMAN	GDI2	51 kDa	5	0	[illegible]
477	Ubiquitin-associated domain-containing protein 1 OS=Homo sapiens OX=9606 GN=UBAC1 PE=1 SV=1	UBAC1_HUMAN	UBAC1	45 kDa	10	2	[illegible]
478	ATP-binding cassette sub-family A member 7 OS=Homo sapiens OX=9606 GN=ABCA7 PE=1 SV=3	ABCA7_HUMAN	ABCA7	234 kDa	2	4	[illegible]
479	Porphobilinogen deaminase OS=Homo sapiens OX=9606 GN=HMBS PE=1 SV=2	HEM3_HUMAN	HMBS	39 kDa	2	1	[illegible]
480	Epidermal growth factor receptor substrate 15 OS=Homo sapiens OX=9606 GN=EPS15 PE=1 SV=2	EPS15_HUMAN	EPS15	99 kDa	2	4	[illegible]
481	Endoplasmic reticulum-Golgi intermediate compartment protein 1 OS=Homo sapiens OX=9606 GN=ERGIC1 PE=1 SV=1	ERGI1_HUMAN	ERGIC1	33 kDa	1	1	[illegible]
482	Ribonuclease inhibitor OS=Homo sapiens OX=9606 GN=RNH1 PE=1 SV=2	RINI_HUMAN	RNH1	50 kDa	11		[illegible]
483	Signal transducing adapter molecule 1 OS=Homo sapiens OX=9606 GN=STAM PE=1 SV=3	STAM1_HUMAN	STAM	59 kDa	11	2	[illegible]
484	Ubiquitin carboxyl-terminal hydrolase isozyme L5 OS=Homo sapiens OX=9606 GN=UCHL5 PE=1 SV=3	UCHL5_HUMAN	UCHL5	38 kDa	1	2	[illegible]
485	Guanine nucleotide-binding protein subunit beta-4 OS=Homo sapiens OX=9606 GN=GNB4 PE=1 SV=3	GBB4_HUMAN	GNB4	38 kDa	11	16	[illegible]
486	Small VCP/p97-interacting protein OS=Homo sapiens OX=9606 GN=SVIP PE=1 SV=1	SVIP_HUMAN	SVIP	8 kDa	1	2	[illegible]
487	Septin-2 OS=Homo sapiens OX=9606 GN=SEPT2 PE=1 SV=1	SEPT2_HUMAN	2-Sep	41 kDa	1		[illegible]
488	Dynactin subunit 4 OS=Homo sapiens OX=9606 GN=DCTN4 PE=1 SV=1	DCTN4_HUMAN	DCTN4	52 kDa	2	4	[illegible]
489	Cullin-4A OS=Homo sapiens OX=9606 GN=CUL4A PE=1 SV=3	CUL4A_HUMAN	CUL4A	88 kDa	11	3	[illegible]
490	Adenosylhomocysteinase OS=Homo sapiens OX=9606 GN=AHCY PE=1 SV=4	SAHH_HUMAN	AHCY	48 kDa	9	2	[illegible]
491	Mitochondrial fission 1 protein OS=Homo sapiens OX=9606 GN=FIS1 PE=1 SV=2	FIS1_HUMAN	FIS1	17 kDa	4	3	[illegible]
492	Cell cycle control protein 50A OS=Homo sapiens OX=9606 GN=TMEM30A PE=1 SV=1	CC50A_HUMAN	TMEM30A	41 kDa			[illegible]
493	Endophilin-B2 OS=Homo sapiens OX=9606 GN=SH3GLB2 PE=1 SV=1	SHLB2_HUMAN	SH3GLB2	44 kDa	4	1	[illegible]
494	E3 ubiquitin-protein ligase HECTD3 OS=Homo sapiens OX=9606 GN=HECTD3 PE=1 SV=1	HECD3_HUMAN	HECTD3	97 kDa	3	4	[illegible]
495	Immunoglobulin kappa variable 3-20 OS=Homo sapiens OX=9606 GN=IGKV3-20 PE=1 SV=2	KV320_HUMAN	IGKV3-20	13 kDa			[illegible]
496	DnaJ homolog subfamily C member 5 OS=Homo sapiens OX=9606 GN=DNAJC5 PE=1 SV=1	DNJC5_HUMAN	DNAJC5	22 kDa			[illegible]
497	Charged multivesicular body protein 6 OS=Homo sapiens OX=9606 GN=CHMP6 PE=1 SV=3	CHMP6_HUMAN	CHMP6	23 kDa	5	5	[illegible]
498	Endoplasmic reticulum-Golgi intermediate compartment protein 2 OS=Homo sapiens OX=9606 GN=ERGIC2 PE=1 SV=2	ERGI2_HUMAN	ERGIC2	43 kDa	2	1	[illegible]
499	Selenocysteine-specific elongation factor OS=Homo sapiens OX=9606 GN=EEFSEC PE=1 SV=4	SELB_HUMAN	EEFSEC	65 kDa	2	1	[illegible]
500	1-acyl-sn-glycerol-3-phosphate acyltransferase alpha OS=Homo sapiens OX=9606 GN=AGPAT1 PE=1 SV=2	PLCA_HUMAN	AGPAT1	32 kDa	3	0	[illegible]
501	Elongation factor 2 OS=Homo sapiens OX=9606 GN=EEF2 PE=1 SV=4	EF2_HUMAN	EEF2	95 kDa			[illegible]
502	Tubulin alpha-1B chain OS=Homo sapiens OX=9606 GN=TUBA1B PE=1 SV=1	TBA1B_HUMAN	TUBA1B	50 kDa			[illegible]
503	Protein FAM234A OS=Homo sapiens OX=9606 GN=FAM234A PE=1 SV=1	F234A_HUMAN	FAM234A	60 kDa	7	5	[illegible]
504	Catechol O-methyltransferase OS=Homo sapiens OX=9606 GN=COMT PE=1 SV=2	COMT_HUMAN	COMT	30 kDa		4	[illegible]
505	Tyrosine--tRNA ligase, cytoplasmic OS=Homo sapiens OX=9606 GN=YARS PE=1 SV=4	SYYC_HUMAN	YARS	59 kDa	0		[illegible]
506	Transmembrane 9 superfamily member 2 OS=Homo sapiens OX=9606 GN=TM9SF2 PE=1 SV=1	TM9S2_HUMAN	TM9SF2	76 kDa	2	2	[illegible]
507	BAG family molecular chaperone regulator 2 OS=Homo sapiens OX=9606 GN=BAG2 PE=1 SV=1	BAG2_HUMAN	BAG2	24 kDa	3	4	[illegible]
508	Sarcoplasmic/endoplasmic reticulum calcium ATPase 2 OS=Homo sapiens OX=9606 GN=ATP2A2 PE=1 SV=1	AT2A2_HUMAN	ATP2A2	115 kDa	2	3	[illegible]
509	Cytochrome b reductase 1 OS=Homo sapiens OX=9606 GN=CYBRD1 PE=1 SV=1	CYBR1_HUMAN	CYBRD1	32 kDa	3	2	[illegible]
510	Glutathione peroxidase 1 OS=Homo sapiens OX=9606 GN=GPX1 PE=1 SV=4	GPX1_HUMAN	GPX1	22 kDa	2	1	[illegible]
511	Adaptin ear-binding coat-associated protein 2 OS=Homo sapiens OX=9606 GN=NECAP2 PE=1 SV=1	NECP2_HUMAN	NECAP2	28 kDa	2	2	[illegible]
512	Guanine nucleotide-binding protein G(I)/G(S)/G(O) subunit gamma-2 OS=Homo sapiens OX=9606 GN=GNG2 PE=1 SV=2	GBG2_HUMAN	GNG2	8 kDa	2	2	[illegible]
513	14-3-3 protein beta/alpha OS=Homo sapiens OX=9606 GN=YWHAB PE=1 SV=3	1433B_HUMAN	YWHAB	28 kDa	10	4	[illegible]
514	Casein kinase II subunit alpha OS=Homo sapiens OX=9606 GN=CSNK2A1 PE=1 SV=1	CSK21_HUMAN	CSNK2A1	45 kDa	2	1	[illegible]
515	Ran GTPase-activating protein 1 OS=Homo sapiens OX=9606 GN=RANGAP1 PE=1 SV=1	RAGP1_HUMAN	RANGAP1	64 kDa	10	5	[illegible]
516	Sec1 family domain-containing protein 2 OS=Homo sapiens OX=9606 GN=SCFD2 PE=1 SV=2	SCFD2_HUMAN	SCFD2	75 kDa	1	2	[illegible]
517	UBX domain-containing protein 1 OS=Homo sapiens OX=9606 GN=UBXN1 PE=1 SV=2	UBXN1_HUMAN	UBXN1	33 kDa	4	1	[illegible]
518	Cytokine receptor-like factor 3 OS=Homo sapiens OX=9606 GN=CRLF3 PE=1 SV=2	CRLF3_HUMAN	CRLF3	50 kDa	9	1	[illegible]
519	Tubulin beta-4B chain OS=Homo sapiens OX=9606 GN=TUBB4B PE=1 SV=1	TBB4B_HUMAN	TUBB4B	50 kDa	0		[illegible]
520	Clathrin light chain A OS=Homo sapiens OX=9606 GN=CLTA PE=1 SV=1	CLCA_HUMAN	CLTA	27 kDa	0	0	[illegible]
521	Serotransferrin OS=Homo sapiens OX=9606 GN=TF PE=1 SV=3	TRFE_HUMAN	TF	77 kDa	2		[illegible]
522	Importin subunit alpha-3 OS=Homo sapiens OX=9606 GN=KPNA4 PE=1 SV=1	IMA3_HUMAN	KPNA4	58 kDa	4	6	[illegible]
523	Serine/threonine-protein phosphatase 2A catalytic subunit alpha isoform OS=Homo sapiens OX=9606 GN=PPP2CA PE=1 SV=1	PP2AA_HUMAN	PPP2CA	36 kDa	9		[illegible]
524	Peroxiredoxin-4 OS=Homo sapiens OX=9606 GN=PRDX4 PE=1 SV=1	PRDX4_HUMAN	PRDX4	31 kDa	4	2	[illegible]
525	Tyrosine-protein kinase SYK OS=Homo sapiens OX=9606 GN=SYK PE=1 SV=1	KSYK_HUMAN	SYK	72 kDa	4	4	[illegible]
526	GTP-binding protein 1 OS=Homo sapiens OX=9606 GN=GTPBP1 PE=1 SV=3	GTPB1_HUMAN	GTPBP1	72 kDa	0	1	[illegible]
527	Protein GUCD1 OS=Homo sapiens OX=9606 GN=GUCD1 PE=1 SV=2	GUCD1_HUMAN	GUCD1	27 kDa	4	4	[illegible]
528	60S acidic ribosomal protein P2 OS=Homo sapiens OX=9606 GN=RPLP2 PE=1 SV=1	RLA2_HUMAN	RPLP2	12 kDa	2	2	[illegible]
529	Transmembrane channel-like protein 8 OS=Homo sapiens OX=9606 GN=TMC8 PE=1 SV=1	TMC8_HUMAN	TMC8	82 kDa	1		[illegible]
530	Myosin light polypeptide 6 OS=Homo sapiens OX=9606 GN=MYL6 PE=1 SV=2	MYL6_HUMAN	MYL6	17 kDa	1	2	[illegible]
531	Rab3 GTPase-activating protein catalytic subunit OS=Homo sapiens OX=9606 GN=RAB3GAP1 PE=1 SV=3	RB3GP_HUMAN	RAB3GAP1	111 kDa	2	1	[illegible]
532	Serine/threonine-protein kinase TAO3 OS=Homo sapiens OX=9606 GN=TAOK3 PE=1 SV=2	TAOK3_HUMAN	TAOK3	105 kDa	1		[illegible]
533	Ras-related protein Rab-14 OS=Homo sapiens OX=9606 GN=RAB14 PE=1 SV=4	RAB14_HUMAN	RAB14	24 kDa	3	3	[illegible]
534	Fermitin family homolog 3 OS=Homo sapiens OX=9606 GN=FERMT3 PE=1 SV=1	URP2_HUMAN	FERMT3	76 kDa	1		[illegible]
535	Ras-related protein Rab-5A OS=Homo sapiens OX=9606 GN=RAB5A PE=1 SV=2	RAB5A_HUMAN	RAB5A	24 kDa	5	7	[illegible]
536	Sec1 family domain-containing protein 1 OS=Homo sapiens OX=9606 GN=SCFD1 PE=1 SV=4	SCFD1_HUMAN	SCFD1	72 kDa	2	1	[illegible]
537	Adenylyl cyclase-associated protein 1 OS=Homo sapiens OX=9606 GN=CAP1 PE=1 SV=5	CAP1_HUMAN	CAP1	52 kDa	6	3	[illegible]
538	Nuclease-sensitive element-binding protein 1 OS=Homo sapiens OX=9606 GN=YBX1 PE=1 SV=3	YBOX1_HUMAN	YBX1	36 kDa			[illegible]
539	Zinc finger CCCH-type antiviral protein 1 OS=Homo sapiens OX=9606 GN=ZC3HAV1 PE=1 SV=3	ZCCHV_HUMAN	ZC3HAV1	101 kDa	2	0	[illegible]
540	Ribonucleoside-diphosphate reductase large subunit OS=Homo sapiens OX=9606 GN=RRM1 PE=1 SV=1	RIR1_HUMAN	RRM1	90 kDa	3		[illegible]
541	Dolichyldiphosphatase 1 OS=Homo sapiens OX=9606 GN=DOLPP1 PE=2 SV=1	DOPP1_HUMAN	DOLPP1	27 kDa	3	4	[illegible]
542	Casein kinase I isoform gamma-2 OS=Homo sapiens OX=9606 GN=CSNK1G2 PE=1 SV=1	KC1G2_HUMAN	CSNK1G2	47 kDa	3	6	[illegible]
543	Actin-related protein 3 OS=Homo sapiens OX=9606 GN=ACTR3 PE=1 SV=3	ARP3_HUMAN	ACTR3	47 kDa		1	[illegible]
544	N-acyl-phosphatidylethanolamine-hydrolyzing phospholipase D OS=Homo sapiens OX=9606 GN=NAPEPLD PE=1 SV=2	NAPEP_HUMAN	NAPEPLD	46 kDa	3	2	[illegible]
545	Tumor necrosis factor receptor superfamily member 6 OS=Homo sapiens OX=9606 GN=FAS PE=1 SV=1	TNR6_HUMAN	FAS	38 kDa	1		[illegible]
546	Proteasome inhibitor PI31 subunit OS=Homo sapiens OX=9606 GN=PSMF1 PE=1 SV=2	PSMF1_HUMAN	PSMF1	30 kDa	4	2	[illegible]
547	Importin-9 OS=Homo sapiens OX=9606 GN=IPO9 PE=1 SV=3	IPO9_HUMAN	IPO9	116 kDa	11	4	[illegible]
548	Very long-chain acyl-CoA synthetase OS=Homo sapiens OX=9606 GN=SLC27A2 PE=1 SV=2	S27A2_HUMAN	SLC27A2	70 kDa	1	2	[illegible]
549	Keratin, type II cytoskeletal 5 OS=Homo sapiens OX=9606 GN=KRT5 PE=1 SV=3	K2C5_HUMAN	KRT5	62 kDa	10	11	[illegible]
550	Golgin subfamily A member 7 OS=Homo sapiens OX=9606 GN=GOLGA7 PE=1 SV=2	GOGA7_HUMAN	GOLGA7	16 kDa	2	1	[illegible]
551	26S proteasome non-ATPase regulatory subunit 10 OS=Homo sapiens OX=9606 GN=PSMD10 PE=1 SV=1	PSD10_HUMAN	PSMD10	24 kDa	1	2	[illegible]
552	Serpin H1 OS=Homo sapiens OX=9606 GN=SERPINH1 PE=1 SV=2	SERPH_HUMAN	SERPINH1	46 kDa	1	2	[illegible]
553	Casein kinase I isoform gamma-3 OS=Homo sapiens OX=9606 GN=CSNK1G3 PE=1 SV=2	KC1G3_HUMAN	CSNK1G3	51 kDa	0	2	[illegible]
554	Syntaxin-2 OS=Homo sapiens OX=9606 GN=STX2 PE=1 SV=3	STX2_HUMAN	STX2	33 kDa	2	3	[illegible]
555	Dehydrogenase/reductase SDR family member 7B OS=Homo sapiens OX=9606 GN=DHRS7B PE=1 SV=2	DRS7B_HUMAN	DHRS7B	35 kDa	1	3	[illegible]
556	Septin-7 OS=Homo sapiens OX=9606 GN=SEPT7 PE=1 SV=2	SEPT7_HUMAN	7-Sep	51 kDa	1		[illegible]
557	S-phase kinase-associated protein 1 OS=Homo sapiens OX=9606 GN=SKP1 PE=1 SV=2	SKP1_HUMAN	SKP1	19 kDa	5	3	[illegible]
558	Pyruvate kinase PKLR OS=Homo sapiens OX=9606 GN=PKLR PE=1 SV=2	KPYR_HUMAN	PKLR	62 kDa	9		[illegible]
559	Rho-related GTP-binding protein RhoG OS=Homo sapiens OX=9606 GN=RHOG PE=1 SV=1	RHOG_HUMAN	RHOG	21 kDa	2	2	[illegible]
560	Nicotinamide/nicotinic acid mononucleotide adenylyltransferase 3 OS=Homo sapiens OX=9606 GN=NMNAT3 PE=1 SV=2	NMNA3_HUMAN	NMNAT3	28 kDa			[illegible]
561	POTE ankyrin domain family member E OS=Homo sapiens OX=9606 GN=POTEE PE=2 SV=3	POTEE_HUMAN	POTEE	121 kDa	222		[illegible]
562	14-3-3 protein theta OS=Homo sapiens OX=9606 GN=YWHAQ PE=1 SV=1	1433T_HUMAN	YWHAQ	28 kDa	9	2	[illegible]
563	Disks large homolog 1 OS=Homo sapiens OX=9606 GN=DLG1 PE=1 SV=2	DLG1_HUMAN	DLG1	100 kDa	2	4	[illegible]
564	Ras-related protein Rab-2A OS=Homo sapiens OX=9606 GN=RAB2A PE=1 SV=1	RAB2A_HUMAN	RAB2A	24 kDa	2	3	[illegible]
565	V-type proton ATPase subunit H OS=Homo sapiens OX=9606 GN=ATP6V1H PE=1 SV=1	VATH_HUMAN	ATP6V1H	56 kDa	1	1	[illegible]
566	DNA-dependent protein kinase catalytic subunit OS=Homo sapiens OX=9606 GN=PRKDC PE=1 SV=3	PRKDC_HUMAN	PRKDC	469 kDa			[illegible]
567	Mannosyl-oligosaccharide glucosidase OS=Homo sapiens OX=9606 GN=MOGS PE=1 SV=5	MOGS_HUMAN	MOGS	92 kDa	2	2	[illegible]
568	Receptor expression-enhancing protein 6 OS=Homo sapiens OX=9606 GN=REEP6 PE=1 SV=2	REEP6_HUMAN	REEP6	23 kDa	3	3	[illegible]
569	Tumor protein D54 OS=Homo sapiens OX=9606 GN=TPD52L2 PE=1 SV=2	TPD54_HUMAN	TPD52L2	22 kDa	1	3	[illegible]
570	Gamma-aminobutyric acid receptor-associated protein-like 1 OS=Homo sapiens OX=9606 GN=GABARAPL1 PE=1 SV=1	GBRL1_HUMAN	GABARAPL1	14 kDa			[illegible]
571	ATPase ASNA1 OS=Homo sapiens OX=9606 GN=ASNA1 PE=1 SV=2	ASNA_HUMAN	ASNA1	39 kDa	2	1	[illegible]
572	Glycerophosphodiester phosphodiesterase 1 OS=Homo sapiens OX=9606 GN=GDE1 PE=1 SV=1	GDE1_HUMAN	GDE1	38 kDa	1	0	[illegible]
573	High affinity copper uptake protein 1 OS=Homo sapiens OX=9606 GN=SLC31A1 PE=1 SV=1	COPT1_HUMAN	SLC31A1	21 kDa	0	1	[illegible]
574	Coiled-coil domain-containing protein 124 OS=Homo sapiens OX=9606 GN=CCDC124 PE=1 SV=1	CC124_HUMAN	CCDC124	26 kDa		2	[illegible]
575	Nucleobindin-2 OS=Homo sapiens OX=9606 GN=NUCB2 PE=1 SV=3	NUCB2_HUMAN	NUCB2	50 kDa	2	1	[illegible]
576	Dolichyl-diphosphooligosaccharide--protein glycosyltransferase subunit 2 OS=Homo sapiens OX=9606 GN=RPN2 PE=1 SV=3	RPN2_HUMAN	RPN2	69 kDa	4	1	[illegible]
577	E3 ubiquitin-protein ligase TRIM58 OS=Homo sapiens OX=9606 GN=TRIM58 PE=2 SV=2	TRI58_HUMAN	TRIM58	55 kDa	1		[illegible]
578	Serine/threonine-protein phosphatase 6 regulatory subunit 3 OS=Homo sapiens OX=9606 GN=PPP6R3 PE=1 SV=2	PP6R3_HUMAN	PPP6R3	98 kDa	3	1	[illegible]
579	Ubiquitin-fold modifier 1 OS=Homo sapiens OX=9606 GN=UFM1 PE=1 SV=1	UFM1_HUMAN	UFM1	9 kDa	3	3	[illegible]
580	Cullin-2 OS=Homo sapiens OX=9606 GN=CUL2 PE=1 SV=2	CUL2_HUMAN	CUL2	87 kDa	8	2	[illegible]
581	Hepatocyte growth factor-regulated tyrosine kinase substrate OS=Homo sapiens OX=9606 GN=HGS PE=1 SV=1	HGS_HUMAN	HGS	86 kDa	2	2	[illegible]
582	Gephyrin OS=Homo sapiens OX=9606 GN=GPHN PE=1 SV=1	GEPH_HUMAN	GPHN	80 kDa	1	3	[illegible]
583	Cyclin-dependent kinase 2 OS=Homo sapiens OX=9606 GN=CDK2 PE=1 SV=2	CDK2_HUMAN	CDK2	34 kDa			[illegible]
584	Hemoglobin subunit mu OS=Homo sapiens OX=9606 GN=HBM PE=1 SV=1	HBM_HUMAN	HBM	16 kDa			[illegible]
585	MICAL-like protein 2 OS=Homo sapiens OX=9606 GN=MICALL2 PE=1 SV=1	MILK2_HUMAN	MICALL2	98 kDa			[illegible]
586	TSC22 domain family protein 4 OS=Homo sapiens OX=9606 GN=TSC22D4 PE=1 SV=2	T22D4_HUMAN	TSC22D4	41 kDa	2		[illegible]
587	Small glutamine-rich tetratricopeptide repeat-containing protein alpha OS=Homo sapiens OX=9606 GN=SGTA PE=1 SV=1	SGTA_HUMAN	SGTA	34 kDa	1	1	[illegible]
588	Deoxyribonuclease-1-like 1 OS=Homo sapiens OX=9606 GN=DNASE1L1 PE=1 SV=1	DNSL1_HUMAN	DNASE1L1	34 kDa			[illegible]
589	Oxysterol-binding protein 2 OS=Homo sapiens OX=9606 GN=OSBP2 PE=1 SV=2	OSBP2_HUMAN	OSBP2	101 kDa	5	2	[illegible]
590	Bleomycin hydrolase OS=Homo sapiens OX=9606 GN=BLMH PE=1 SV=1	BLMH_HUMAN	BLMH	53 kDa	2	2	[illegible]
591	Immunoglobulin heavy constant gamma 4 OS=Homo sapiens OX=9606 GN=IGHG4 PE=1 SV=1	IGHG4_HUMAN	IGHG4	36 kDa	21	16	[illegible]
592	Endoplasmic reticulum junction formation protein lunapark OS=Homo sapiens OX=9606 GN=LNPK PE=1 SV=2	LNP_HUMAN	LNPK	48 kDa	2	1	[illegible]
593	Peroxiredoxin-6 OS=Homo sapiens OX=9606 GN=PRDX6 PE=1 SV=3	PRDX6_HUMAN	PRDX6	25 kDa	1	2	[illegible]
594	Lysophospholipid acyltransferase 5 OS=Homo sapiens OX=9606 GN=LPCAT3 PE=1 SV=1	MBOA5_HUMAN	LPCAT3	56 kDa	1	0	[illegible]
595	NIF3-like protein 1 OS=Homo sapiens OX=9606 GN=NIF3L1 PE=1 SV=2	NIF3L_HUMAN	NIF3L1	42 kDa	4		[illegible]
596	Chitinase domain-containing protein 1 OS=Homo sapiens OX=9606 GN=CHID1 PE=1 SV=1	CHID1_HUMAN	CHID1	45 kDa	2		[illegible]
597	O-phosphoseryl-tRNA(Sec) selenium transferase OS=Homo sapiens OX=9606 GN=SEPSECS PE=1 SV=2	SPCS_HUMAN	SEPSECS	56 kDa			[illegible]
598	EH domain-containing protein 1 OS=Homo sapiens OX=9606 GN=EHD1 PE=1 SV=2	EHD1_HUMAN	EHD1	61 kDa			[illegible]
599	Protein-L-isoaspartate(D-aspartate) O-methyltransferase OS=Homo sapiens OX=9606 GN=PCMT1 PE=1 SV=4	PIMT_HUMAN	PCMT1	25 kDa	2	1	[illegible]
600	ADP-ribosylation factor-like protein 15 OS=Homo sapiens OX=9606 GN=ARL15 PE=1 SV=1	ARL15_HUMAN	ARL15	23 kDa	4	2	[illegible]
601	Eukaryotic translation initiation factor 5A-1 OS=Homo sapiens OX=9606 GN=EIF5A PE=1 SV=2	IF5A1_HUMAN	EIF5A	17 kDa			[illegible]
602	Unconventional myosin-Ic OS=Homo sapiens OX=9606 GN=MYO1C PE=1 SV=4	MYO1C_HUMAN	MYO1C	122 kDa	3		[illegible]
603	Secretory carrier-associated membrane protein 4 OS=Homo sapiens OX=9606 GN=SCAMP4 PE=1 SV=1	SCAM4_HUMAN	SCAMP4	26 kDa			[illegible]
604	DnaJ homolog subfamily B member 2 OS=Homo sapiens OX=9606 GN=DNAJB2 PE=1 SV=3	DNJB2_HUMAN	DNAJB2	36 kDa			[illegible]
605	60S ribosomal protein L13 OS=Homo sapiens OX=9606 GN=RPL13 PE=1 SV=4	RL13_HUMAN	RPL13	24 kDa	2	3	[illegible]
606	Vacuolar protein sorting-associated protein 45 OS=Homo sapiens OX=9606 GN=VPS45 PE=1 SV=1	VPS45_HUMAN	VPS45	65 kDa	1	2	[illegible]
607	AMP deaminase 3 OS=Homo sapiens OX=9606 GN=AMPD3 PE=1 SV=1	AMPD3_HUMAN	AMPD3	89 kDa	1		[illegible]
608	Y-box-binding protein 3 OS=Homo sapiens OX=9606 GN=YBX3 PE=1 SV=4	YBOX3_HUMAN	YBX3	40 kDa	1	1	[illegible]
609	Immediate early response 3-interacting protein 1 OS=Homo sapiens OX=9606 GN=IER3IP1 PE=1 SV=1	IR3IP_HUMAN	IER3IP1	9 kDa	4	2	[illegible]
610	Synaptogyrin-2 OS=Homo sapiens OX=9606 GN=SYNGR2 PE=1 SV=1	SNG2_HUMAN	SYNGR2	25 kDa	1	1	[illegible]
611	Pleckstrin-2 OS=Homo sapiens OX=9606 GN=PLEK2 PE=1 SV=1	PLEK2_HUMAN	PLEK2	40 kDa	2	2	[illegible]
612	Peptidyl-prolyl cis-trans isomerase A OS=Homo sapiens OX=9606 GN=PPIA PE=1 SV=2	PPIA_HUMAN	PPIA	18 kDa	1	0	[illegible]
613	CAD protein OS=Homo sapiens OX=9606 GN=CAD PE=1 SV=3	PYR1_HUMAN	CAD	243 kDa	2		[illegible]
614	Glyceraldehyde-3-phosphate dehydrogenase, testis-specific OS=Homo sapiens OX=9606 GN=GAPDHS PE=1 SV=2	G3PT_HUMAN	GAPDHS	45 kDa		0	[illegible]
615	IST1 homolog OS=Homo sapiens OX=9606 GN=IST1 PE=1 SV=1	IST1_HUMAN	IST1	40 kDa	0		[illegible]
616	Ran-binding protein 10 OS=Homo sapiens OX=9606 GN=RANBP10 PE=1 SV=1	RBP10_HUMAN	RANBP10	67 kDa			[illegible]
617	Eukaryotic translation initiation factor 4 gamma 1 OS=Homo sapiens OX=9606 GN=EIF4G1 PE=1 SV=4	IF4G1_HUMAN	EIF4G1	175 kDa	2	1	[illegible]
618	Keratin, type II cuticular Hb6 OS=Homo sapiens OX=9606 GN=KRT86 PE=1 SV=1	KRT86_HUMAN	KRT86	53 kDa			[illegible]
619	Retinol dehydrogenase 11 OS=Homo sapiens OX=9606 GN=RDH11 PE=1 SV=2	RDH11_HUMAN	RDH11	35 kDa		2	[illegible]
620	Heterogeneous nuclear ribonucleoprotein D0 OS=Homo sapiens OX=9606 GN=HNRNPD PE=1 SV=1	HNRPD_HUMAN	HNRNPD	38 kDa		1	[illegible]
621	Palmitoyltransferase ZDHHC3 OS=Homo sapiens OX=9606 GN=ZDHHC3 PE=1 SV=2	ZDHC3_HUMAN	ZDHHC3	34 kDa	2	0	[illegible]
622	60S ribosomal protein L12 OS=Homo sapiens OX=9606 GN=RPL12 PE=1 SV=1	RL12_HUMAN	RPL12	18 kDa	1	3	[illegible]

623	Protein kish-A OS=Homo sapiens OX=9606 GN=TMEM167A PE=1 SV=1	KISHA_HUMAN	TMEM167A	8 kDa		1	[illegible]
624	Phosphatidylinositol 3,4,5-trisphosphate 5-phosphatase 1 OS=Homo sapiens OX=9606 GN=INPP5D PE=1 SV=2	SHIP1_HUMAN	INPP5D	133 kDa	5	2	[illegible]
625	Serine/threonine-protein phosphatase 5 OS=Homo sapiens OX=9606 GN=PPP5C PE=1 SV=1	PPP5_HUMAN	PPP5C	57 kDa	2	1	[illegible]
626	Rab3 GTPase-activating protein non-catalytic subunit OS=Homo sapiens OX=9606 GN=RAB3GAP2 PE=1 SV=1	RBGPR_HUMAN	RAB3GAP2	156 kDa	2		[illegible]
627	Proteasome assembly chaperone 1 OS=Homo sapiens OX=9606 GN=PSMG1 PE=1 SV=1	PSMG1_HUMAN	PSMG1	33 kDa	1		[illegible]
628	Sodium/potassium-transporting ATPase subunit beta-3 OS=Homo sapiens OX=9606 GN=ATP1B3 PE=1 SV=1	AT1B3_HUMAN	ATP1B3	32 kDa			[illegible]
629	Heterogeneous nuclear ribonucleoprotein Q OS=Homo sapiens OX=9606 GN=SYNCRIP PE=1 SV=2	HNRPQ_HUMAN	SYNCRIP	70 kDa	3	4	[illegible]
630	Syntaxin-6 OS=Homo sapiens OX=9606 GN=STX6 PE=1 SV=1	STX6_HUMAN	STX6	29 kDa	1		[illegible]
631	Uncharacterized protein C1orf198 OS=Homo sapiens OX=9606 GN=C1orf198 PE=1 SV=1	CA198_HUMAN	C1orf198	36 kDa	2	3	[illegible]
632	Dynactin subunit 3 OS=Homo sapiens OX=9606 GN=DCTN3 PE=1 SV=1	DCTN3_HUMAN	DCTN3	21 kDa	1	3	[illegible]
633	Sorting nexin-2 OS=Homo sapiens OX=9606 GN=SNX2 PE=1 SV=2	SNX2_HUMAN	SNX2	58 kDa			[illegible]
634	Oxysterol-binding protein-related protein 8 OS=Homo sapiens OX=9606 GN=OSBPL8 PE=1 SV=3	OSBL8_HUMAN	OSBPL8	101 kDa	1		[illegible]
635	DnaJ homolog subfamily A member 4 OS=Homo sapiens OX=9606 GN=DNAJA4 PE=1 SV=1	DNJA4_HUMAN	DNAJA4	45 kDa	1		[illegible]
636	Acidic leucine-rich nuclear phosphoprotein 32 family member A OS=Homo sapiens OX=9606 GN=ANP32A PE=1 SV=1	AN32A_HUMAN	ANP32A	29 kDa	3	2	[illegible]
637	Choline transporter-like protein 1 OS=Homo sapiens OX=9606 GN=SLC44A1 PE=1 SV=1	CTL1_HUMAN	SLC44A1	73 kDa	1		[illegible]
638	Transmembrane emp24 domain-containing protein 1 OS=Homo sapiens OX=9606 GN=TMED1 PE=1 SV=1	TMED1_HUMAN	TMED1	25 kDa			[illegible]
639	Beta-arrestin-2 OS=Homo sapiens OX=9606 GN=ARRB2 PE=1 SV=2	ARRB2_HUMAN	ARRB2	46 kDa	1	0	[illegible]
640	Peptidyl-prolyl cis-trans isomerase FKBP2 OS=Homo sapiens OX=9606 GN=FKBP2 PE=1 SV=2	FKBP2_HUMAN	FKBP2	16 kDa	3	3	[illegible]
641	Endoplasmic reticulum aminopeptidase 1 OS=Homo sapiens OX=9606 GN=ERAP1 PE=1 SV=3	ERAP1_HUMAN	ERAP1	107 kDa	4		[illegible]
642	Protein PRRC1 OS=Homo sapiens OX=9606 GN=PRRC1 PE=1 SV=1	PRRC1_HUMAN	PRRC1	47 kDa	1		[illegible]
643	Tubulin--tyrosine ligase-like protein 12 OS=Homo sapiens OX=9606 GN=TTLL12 PE=1 SV=2	TTL12_HUMAN	TTLL12	74 kDa	2		[illegible]
644	Spectrin beta chain, non-erythrocytic 2 OS=Homo sapiens OX=9606 GN=SPTBN2 PE=1 SV=3	SPTN2_HUMAN	SPTBN2	271 kDa	12		[illegible]
645	RING finger and SPRY domain-containing protein 1 OS=Homo sapiens OX=9606 GN=RSPRY1 PE=1 SV=1	RSPRY_HUMAN	RSPRY1	64 kDa	2	3	[illegible]
646	PITH domain-containing protein 1 OS=Homo sapiens OX=9606 GN=PITHD1 PE=1 SV=1	PITH1_HUMAN	PITHD1	24 kDa	1	1	[illegible]
647	V-type proton ATPase subunit E 1 OS=Homo sapiens OX=9606 GN=ATP6V1E1 PE=1 SV=1	VATE1_HUMAN	ATP6V1E1	26 kDa	7		[illegible]
648	Ubiquitin carboxyl-terminal hydrolase 7 OS=Homo sapiens OX=9606 GN=USP7 PE=1 SV=2	UBP7_HUMAN	USP7	128 kDa	6		[illegible]
649	Ras-related protein Ral-B OS=Homo sapiens OX=9606 GN=RALB PE=1 SV=1	RALB_HUMAN	RALB	23 kDa	5	4	[illegible]
650	Translocon-associated protein subunit delta OS=Homo sapiens OX=9606 GN=SSR4 PE=1 SV=1	SSRD_HUMAN	SSR4	19 kDa	1	3	[illegible]
651	Phosphatidylserine lipase ABHD16A OS=Homo sapiens OX=9606 GN=ABHD16A PE=1 SV=3	ABHGA_HUMAN	ABHD16A	63 kDa	2		[illegible]
652	Serine--tRNA ligase, cytoplasmic OS=Homo sapiens OX=9606 GN=SARS PE=1 SV=3	SYSC_HUMAN	SARS	59 kDa			[illegible]
653	Uroporphyrinogen decarboxylase OS=Homo sapiens OX=9606 GN=UROD PE=1 SV=2	DCUP_HUMAN	UROD	41 kDa	2		[illegible]
654	Desmoplakin OS=Homo sapiens OX=9606 GN=DSP PE=1 SV=3	DESP_HUMAN	DSP	332 kDa		2	[illegible]
655	Long-chain-fatty-acid--CoA ligase 5 OS=Homo sapiens OX=9606 GN=ACSL5 PE=1 SV=1	ACSL5_HUMAN	ACSL5	76 kDa	5	2	[illegible]
656	Erbin OS=Homo sapiens OX=9606 GN=ERBIN PE=1 SV=2	ERBIN_HUMAN	ERBIN	158 kDa	4	5	[illegible]
657	Protein RER1 OS=Homo sapiens OX=9606 GN=RER1 PE=1 SV=1	RER1_HUMAN	RER1	23 kDa	2		[illegible]
658	Bisphosphoglycerate mutase OS=Homo sapiens OX=9606 GN=BPGM PE=1 SV=2	PMGE_HUMAN	BPGM	30 kDa	4	2	[illegible]
659	Epidermal growth factor receptor substrate 15-like 1 OS=Homo sapiens OX=9606 GN=EPS15L1 PE=1 SV=1	EP15R_HUMAN	EPS15L1	94 kDa			[illegible]
660	Zinc transporter 7 OS=Homo sapiens OX=9606 GN=SLC30A7 PE=2 SV=1	ZNT7_HUMAN	SLC30A7	42 kDa	2	2	[illegible]
661	WD repeat-containing protein 26 OS=Homo sapiens OX=9606 GN=WDR26 PE=1 SV=3	WDR26_HUMAN	WDR26	72 kDa	1	2	[illegible]
662	Phospholipase D1 OS=Homo sapiens OX=9606 GN=PLD1 PE=1 SV=1	PLD1_HUMAN	PLD1	124 kDa			[illegible]
663	Transmembrane emp24 domain-containing protein 5 OS=Homo sapiens OX=9606 GN=TMED5 PE=1 SV=1	TMED5_HUMAN	TMED5	26 kDa	1	1	[illegible]
664	Spastin OS=Homo sapiens OX=9606 GN=SPAST PE=1 SV=1	SPAST_HUMAN	SPAST	67 kDa	0		[illegible]
665	GDP-L-fucose synthase OS=Homo sapiens OX=9606 GN=TSTA3 PE=1 SV=1	FCL_HUMAN	TSTA3	36 kDa	3	2	[illegible]
666	Vacuolar protein sorting-associated protein 13C OS=Homo sapiens OX=9606 GN=VPS13C PE=1 SV=1	VP13C_HUMAN	VPS13C	422 kDa	4	1	[illegible]
667	Ubiquitin-like protein 4A OS=Homo sapiens OX=9606 GN=UBL4A PE=1 SV=1	UBL4A_HUMAN	UBL4A	18 kDa		1	[illegible]
668	Ephrin-B1 OS=Homo sapiens OX=9606 GN=EFNB1 PE=1 SV=1	EFNB1_HUMAN	EFNB1	38 kDa	3	2	[illegible]
669	Transferrin receptor protein 1 OS=Homo sapiens OX=9606 GN=TFRC PE=1 SV=2	TFR1_HUMAN	TFRC	85 kDa		1	[illegible]
670	CD2-associated protein OS=Homo sapiens OX=9606 GN=CD2AP PE=1 SV=1	CD2AP_HUMAN	CD2AP	71 kDa	2		[illegible]
671	Fibrinogen gamma chain OS=Homo sapiens OX=9606 GN=FGG PE=1 SV=3	FIBG_HUMAN	FGG	52 kDa	4		[illegible]
672	Presenilin-1 OS=Homo sapiens OX=9606 GN=PSEN1 PE=1 SV=1	PSN1_HUMAN	PSEN1	53 kDa	1	2	[illegible]
673	Translocon-associated protein subunit alpha OS=Homo sapiens OX=9606 GN=SSR1 PE=1 SV=3	SSRA_HUMAN	SSR1	32 kDa	1		[illegible]
674	Synaptic vesicle membrane protein VAT-1 homolog OS=Homo sapiens OX=9606 GN=VAT1 PE=1 SV=2	VAT1_HUMAN	VAT1	42 kDa	0		[illegible]
675	Ribosome maturation protein SBDS OS=Homo sapiens OX=9606 GN=SBDS PE=1 SV=4	SBDS_HUMAN	SBDS	29 kDa			[illegible]
676	Complement C3 OS=Homo sapiens OX=9606 GN=C3 PE=1 SV=2	CO3_HUMAN	C3	187 kDa			[illegible]
677	Immunoglobulin heavy variable 3-74 OS=Homo sapiens OX=9606 GN=IGHV3-74 PE=3 SV=1	HV374_HUMAN	IGHV3-74	13 kDa	1		[illegible]
678	Rho-related GTP-binding protein RhoB OS=Homo sapiens OX=9606 GN=RHOB PE=1 SV=1	RHOB_HUMAN	RHOB	22 kDa			[illegible]
679	Sn1-specific diacylglycerol lipase beta OS=Homo sapiens OX=9606 GN=DAGLB PE=1 SV=2	DGLB_HUMAN	DAGLB	74 kDa	1	3	[illegible]
680	Immunoglobulin kappa variable 3-15 OS=Homo sapiens OX=9606 GN=IGKV3-15 PE=1 SV=2	KV315_HUMAN	IGKV3-15	12 kDa	1	2	[illegible]
681	cAMP-dependent protein kinase catalytic subunit beta OS=Homo sapiens OX=9606 GN=PRKACB PE=1 SV=2	KAPCB_HUMAN	PRKACB	41 kDa	8		[illegible]
682	60S acidic ribosomal protein P0 OS=Homo sapiens OX=9606 GN=RPLP0 PE=1 SV=1	RLA0_HUMAN	RPLP0	34 kDa	2		[illegible]
683	High affinity cationic amino acid transporter 1 OS=Homo sapiens OX=9606 GN=SLC7A1 PE=1 SV=1	CTR1_HUMAN	SLC7A1	68 kDa	1	0	[illegible]
684	Transmembrane channel-like protein 6 OS=Homo sapiens OX=9606 GN=TMC6 PE=1 SV=2	TMC6_HUMAN	TMC6	90 kDa			[illegible]
685	Spectrin alpha chain, non-erythrocytic 1 OS=Homo sapiens OX=9606 GN=SPTAN1 PE=1 SV=3	SPTN1_HUMAN	SPTAN1	285 kDa	0	2	[illegible]
686	Sodium/potassium-transporting ATPase subunit beta-2 OS=Homo sapiens OX=9606 GN=ATP1B2 PE=1 SV=3	AT1B2_HUMAN	ATP1B2	33 kDa			[illegible]
687	Lysosome-associated membrane glycoprotein 1 OS=Homo sapiens OX=9606 GN=LAMP1 PE=1 SV=3	LAMP1_HUMAN	LAMP1	45 kDa	1	1	[illegible]
688	Fibrinogen beta chain OS=Homo sapiens OX=9606 GN=FGB PE=1 SV=2	FIBB_HUMAN	FGB	56 kDa			[illegible]
689	60S ribosomal protein L8 OS=Homo sapiens OX=9606 GN=RPL8 PE=1 SV=2	RL8_HUMAN	RPL8	28 kDa	3	2	[illegible]
690	Polyadenylate-binding protein 1 OS=Homo sapiens OX=9606 GN=PABPC1 PE=1 SV=2	PABP1_HUMAN	PABPC1	71 kDa	2	0	[illegible]
691	Vacuolar protein-sorting-associated protein 25 OS=Homo sapiens OX=9606 GN=VPS25 PE=1 SV=1	VPS25_HUMAN	VPS25	21 kDa			[illegible]
692	Adenylate kinase isoenzyme 1 OS=Homo sapiens OX=9606 GN=AK1 PE=1 SV=3	KAD1_HUMAN	AK1	22 kDa	1		[illegible]
693	DnaJ homolog subfamily B member 11 OS=Homo sapiens OX=9606 GN=DNAJB11 PE=1 SV=1	DJB11_HUMAN	DNAJB11	41 kDa	1	1	[illegible]
694	DCN1-like protein 1 OS=Homo sapiens OX=9606 GN=DCUN1D1 PE=1 SV=1	DCNL1_HUMAN	DCUN1D1	30 kDa	2	1	[illegible]
695	Keratin, type I cuticular Ha1 OS=Homo sapiens OX=9606 GN=KRT31 PE=1 SV=3	K1H1_HUMAN	KRT31	47 kDa		1	[illegible]
696	Methylosome subunit pICln OS=Homo sapiens OX=9606 GN=CLNS1A PE=1 SV=1	ICLN_HUMAN	CLNS1A	26 kDa	2		[illegible]
697	Protein canopy homolog 2 OS=Homo sapiens OX=9606 GN=CNPY2 PE=1 SV=1	CNPY2_HUMAN	CNPY2	21 kDa		0	[illegible]
698	Transmembrane protein 14C OS=Homo sapiens OX=9606 GN=TMEM14C PE=1 SV=1	TM14C_HUMAN	TMEM14C	12 kDa	1	3	[illegible]
699	Proteasomal ubiquitin receptor ADRM1 OS=Homo sapiens OX=9606 GN=ADRM1 PE=1 SV=2	ADRM1_HUMAN	ADRM1	42 kDa	1	1	[illegible]
700	Reticulophagy regulator 3 OS=Homo sapiens OX=9606 GN=RETREG3 PE=1 SV=1	RETR3_HUMAN	RETREG3	51 kDa			[illegible]
701	Probable phospholipid-transporting ATPase IF OS=Homo sapiens OX=9606 GN=ATP11B PE=1 SV=2	AT11B_HUMAN	ATP11B	134 kDa	1	2	[illegible]
702	14-3-3 protein eta OS=Homo sapiens OX=9606 GN=YWHAH PE=1 SV=4	1433F_HUMAN	YWHAH	28 kDa	8	3	[illegible]
703	Autophagy-related protein 9A OS=Homo sapiens OX=9606 GN=ATG9A PE=1 SV=3	ATG9A_HUMAN	ATG9A	94 kDa		1	[illegible]
704	Vesicle transport protein USE1 OS=Homo sapiens OX=9606 GN=USE1 PE=1 SV=2	USE1_HUMAN	USE1	29 kDa	2		[illegible]
705	Nicotinate phosphoribosyltransferase OS=Homo sapiens OX=9606 GN=NAPRT PE=1 SV=2	PNCB_HUMAN	NAPRT	58 kDa	6		[illegible]
706	Erlin-2 OS=Homo sapiens OX=9606 GN=ERLIN2 PE=1 SV=1	ERLN2_HUMAN	ERLIN2	38 kDa	3	1	[illegible]
707	VIP36-like protein OS=Homo sapiens OX=9606 GN=LMAN2L PE=1 SV=1	LMA2L_HUMAN	LMAN2L	40 kDa	0	1	[illegible]
708	Syntaxin-16 OS=Homo sapiens OX=9606 GN=STX16 PE=1 SV=3	STX16_HUMAN	STX16	37 kDa			[illegible]
709	Actin-related protein 2 OS=Homo sapiens OX=9606 GN=ACTR2 PE=1 SV=1	ARP2_HUMAN	ACTR2	45 kDa			[illegible]
710	Glucose-induced degradation protein 8 homolog OS=Homo sapiens OX=9606 GN=GID8 PE=1 SV=1	GID8_HUMAN	GID8	27 kDa	1	3	[illegible]
711	Glutathione reductase, mitochondrial OS=Homo sapiens OX=9606 GN=GSR PE=1 SV=2	GSHR_HUMAN	GSR	56 kDa	1		[illegible]
712	Ferritin light chain OS=Homo sapiens OX=9606 GN=FTL PE=1 SV=2	FRIL_HUMAN	FTL	20 kDa			[illegible]
713	Elongation factor 1-gamma OS=Homo sapiens OX=9606 GN=EEF1G PE=1 SV=3	EF1G_HUMAN	EEF1G	50 kDa			[illegible]
714	Optineurin OS=Homo sapiens OX=9606 GN=OPTN PE=1 SV=3	OPTN_HUMAN	OPTN	66 kDa	4	1	[illegible]
715	L-xylulose reductase OS=Homo sapiens OX=9606 GN=DCXR PE=1 SV=2	DCXR_HUMAN	DCXR	26 kDa	1		[illegible]
716	Bifunctional purine biosynthesis protein PURH OS=Homo sapiens OX=9606 GN=ATIC PE=1 SV=3	PUR9_HUMAN	ATIC	65 kDa	4		[illegible]
717	60S ribosomal protein L18 OS=Homo sapiens OX=9606 GN=RPL18 PE=1 SV=2	RL18_HUMAN	RPL18	22 kDa		1	[illegible]
718	WD repeat and FYVE domain-containing protein 1 OS=Homo sapiens OX=9606 GN=WDFY1 PE=1 SV=1	WDFY1_HUMAN	WDFY1	46 kDa	0	1	[illegible]
719	ER lumen protein-retaining receptor 1 OS=Homo sapiens OX=9606 GN=KDELR1 PE=1 SV=1	ERD21_HUMAN	KDELR1	25 kDa			[illegible]
720	ADP-ribosylation factor-like protein 6-interacting protein 1 OS=Homo sapiens OX=9606 GN=ARL6IP1 PE=1 SV=2	AR6P1_HUMAN	ARL6IP1	23 kDa			[illegible]
721	Merlin OS=Homo sapiens OX=9606 GN=NF2 PE=1 SV=1	MERL_HUMAN	NF2	70 kDa	1		[illegible]
722	Calcium-binding and coiled-coil domain-containing protein 1 OS=Homo sapiens OX=9606 GN=CALCOCO1 PE=1 SV=2	CACO1_HUMAN	CALCOCO1	77 kDa		1	[illegible]
723	Translin-associated protein X OS=Homo sapiens OX=9606 GN=TSNAX PE=1 SV=1	TSNAX_HUMAN	TSNAX	33 kDa	2		[illegible]
724	Vacuolar protein sorting-associated protein 28 homolog OS=Homo sapiens OX=9606 GN=VPS28 PE=1 SV=1	VPS28_HUMAN	VPS28	25 kDa	1		[illegible]
725	Sorting nexin-9 OS=Homo sapiens OX=9606 GN=SNX9 PE=1 SV=1	SNX9_HUMAN	SNX9	67 kDa	1		[illegible]
726	Armadillo repeat-containing protein 8 OS=Homo sapiens OX=9606 GN=ARMC8 PE=1 SV=2	ARMC8_HUMAN	ARMC8	76 kDa		1	[illegible]
727	Immunoglobulin heavy variable 4-34 OS=Homo sapiens OX=9606 GN=IGHV4-34 PE=1 SV=2	HV434_HUMAN	IGHV4-34	14 kDa	1	1	[illegible]
728	Alpha-synuclein OS=Homo sapiens OX=9606 GN=SNCA PE=1 SV=1	SYUA_HUMAN	SNCA	14 kDa	1	1	[illegible]
729	Rho-associated protein kinase 2 OS=Homo sapiens OX=9606 GN=ROCK2 PE=1 SV=4	ROCK2_HUMAN	ROCK2	161 kDa	0		[illegible]
730	Protein argonaute-1 OS=Homo sapiens OX=9606 GN=AGO1 PE=1 SV=3	AGO1_HUMAN	AGO1	97 kDa		25	[illegible]
731	V-type proton ATPase subunit G 1 OS=Homo sapiens OX=9606 GN=ATP6V1G1 PE=1 SV=3	VATG1_HUMAN	ATP6V1G1	14 kDa	2	1	[illegible]
732	PRA1 family protein 2 OS=Homo sapiens OX=9606 GN=PRAF2 PE=1 SV=1	PRAF2_HUMAN	PRAF2	19 kDa	1	2	[illegible]
733	Regulator of nonsense transcripts 3B OS=Homo sapiens OX=9606 GN=UPF3B PE=1 SV=1	REN3B_HUMAN	UPF3B	58 kDa			[illegible]
734	FAS-associated factor 2 OS=Homo sapiens OX=9606 GN=FAF2 PE=1 SV=2	FAF2_HUMAN	FAF2	53 kDa			[illegible]
735	L-lactate dehydrogenase A chain OS=Homo sapiens OX=9606 GN=LDHA PE=1 SV=2	LDHA_HUMAN	LDHA	37 kDa	3	1	[illegible]
736	Rab GTPase-binding effector protein 1 OS=Homo sapiens OX=9606 GN=RABEP1 PE=1 SV=2	RABE1_HUMAN	RABEP1	99 kDa	2	1	[illegible]
737	Titin OS=Homo sapiens OX=9606 GN=TTN PE=1 SV=4	TITIN_HUMAN	TTN	3816 kDa			[illegible]
738	General vesicular transport factor p115 OS=Homo sapiens OX=9606 GN=USO1 PE=1 SV=2	USO1_HUMAN	USO1	108 kDa	1	1	[illegible]
739	Complement C1q subcomponent subunit C OS=Homo sapiens OX=9606 GN=C1QC PE=1 SV=3	C1QC_HUMAN	C1QC	26 kDa	1		[illegible]
740	Ethylmalonyl-CoA decarboxylase OS=Homo sapiens OX=9606 GN=ECHDC1 PE=1 SV=2	ECHD1_HUMAN	ECHDC1	34 kDa		0	[illegible]
741	Clathrin light chain B OS=Homo sapiens OX=9606 GN=CLTB PE=1 SV=1	CLCB_HUMAN	CLTB	25 kDa			[illegible]
742	RNA-binding motif protein, X chromosome OS=Homo sapiens OX=9606 GN=RBMX PE=1 SV=3	RBMX_HUMAN	RBMX	42 kDa	1	1	[illegible]
743	E3 ubiquitin-protein ligase CHIP OS=Homo sapiens OX=9606 GN=STUB1 PE=1 SV=2	CHIP_HUMAN	STUB1	35 kDa			[illegible]
744	Leucyl-cystinyl aminopeptidase OS=Homo sapiens OX=9606 GN=LNPEP PE=1 SV=3	LCAP_HUMAN	LNPEP	117 kDa		2	[illegible]
745	Ras-related protein Rab-1A OS=Homo sapiens OX=9606 GN=RAB1A PE=1 SV=3	RAB1A_HUMAN	RAB1A	23 kDa	8		[illegible]
746	Protein lin-7 homolog C OS=Homo sapiens OX=9606 GN=LIN7C PE=1 SV=1	LIN7C_HUMAN	LIN7C	22 kDa	1		[illegible]
747	Quinone oxidoreductase OS=Homo sapiens OX=9606 GN=CRYZ PE=1 SV=1	QOR_HUMAN	CRYZ	35 kDa	0		[illegible]
748	Nucleoside diphosphate kinase A OS=Homo sapiens OX=9606 GN=NME1 PE=1 SV=1	NDKA_HUMAN	NME1	17 kDa	2		[illegible]
749	Ras-related protein Rap-2c OS=Homo sapiens OX=9606 GN=RAP2C PE=1 SV=1	RAP2C_HUMAN	RAP2C	21 kDa	39	50	[illegible]
750	Lck-interacting transmembrane adapter 1 OS=Homo sapiens OX=9606 GN=LIME1 PE=1 SV=1	LIME1_HUMAN	LIME1	31 kDa	1	1	[illegible]
751	Prosaposin OS=Homo sapiens OX=9606 GN=PSAP PE=1 SV=2	SAP_HUMAN	PSAP	58 kDa	1		[illegible]
752	Platelet basic protein OS=Homo sapiens OX=9606 GN=PPBP PE=1 SV=3	CXCL7_HUMAN	PPBP	14 kDa			[illegible]
753	Alpha-actinin-4 OS=Homo sapiens OX=9606 GN=ACTN4 PE=1 SV=2	ACTN4_HUMAN	ACTN4	105 kDa	1	1	[illegible]
754	Heme-binding protein 1 OS=Homo sapiens OX=9606 GN=HEBP1 PE=1 SV=1	HEBP1_HUMAN	HEBP1	21 kDa	2		[illegible]
755	F-BAR domain only protein 2 OS=Homo sapiens OX=9606 GN=FCHO2 PE=1 SV=2	FCHO2_HUMAN	FCHO2	89 kDa	1	2	[illegible]
756	Nascent polypeptide-associated complex subunit alpha, muscle-specific form OS=Homo sapiens OX=9606 GN=NACA PE=1 SV=1	NACAM_HUMAN	NACA	205 kDa	2		[illegible]
757	HBS1-like protein OS=Homo sapiens OX=9606 GN=HBS1L PE=1 SV=1	HBS1L_HUMAN	HBS1L	75 kDa	1		[illegible]
758	Protein S100-A4 OS=Homo sapiens OX=9606 GN=S100A4 PE=1 SV=1	S10A4_HUMAN	S100A4	12 kDa	0	0	[illegible]
759	Selenoprotein F OS=Homo sapiens OX=9606 GN=SELENOF PE=1 SV=4	SEP15_HUMAN	SELENOF	18 kDa	3	3	[illegible]
760	Myeloid-derived growth factor OS=Homo sapiens OX=9606 GN=MYDGF PE=1 SV=1	MYDGF_HUMAN	MYDGF	19 kDa		2	[illegible]
761	26S proteasome non-ATPase regulatory subunit 9 OS=Homo sapiens OX=9606 GN=PSMD9 PE=1 SV=3	PSMD9_HUMAN	PSMD9	25 kDa			[illegible]
762	Glucose-6-phosphate exchanger SLC37A4 OS=Homo sapiens OX=9606 GN=SLC37A4 PE=1 SV=1	G6PT1_HUMAN	SLC37A4	46 kDa			[illegible]
763	Alpha-mannosidase 2C1 OS=Homo sapiens OX=9606 GN=MAN2C1 PE=1 SV=1	MA2C1_HUMAN	MAN2C1	116 kDa	1		[illegible]
764	CTP synthase 2 OS=Homo sapiens OX=9606 GN=CTPS2 PE=1 SV=1	PYRG2_HUMAN	CTPS2	66 kDa	1		[illegible]
765	Proteasome assembly chaperone 2 OS=Homo sapiens OX=9606 GN=PSMG2 PE=1 SV=1	PSMG2_HUMAN	PSMG2	29 kDa			[illegible]
766	Proteolipid protein 2 OS=Homo sapiens OX=9606 GN=PLP2 PE=1 SV=1	PLP2_HUMAN	PLP2	17 kDa			[illegible]
767	Structural maintenance of chromosomes protein 2 OS=Homo sapiens OX=9606 GN=SMC2 PE=1 SV=2	SMC2_HUMAN	SMC2	136 kDa	1	1	[illegible]
768	Annexin A1 OS=Homo sapiens OX=9606 GN=ANXA1 PE=1 SV=2	ANXA1_HUMAN	ANXA1	39 kDa			[illegible]
769	Syntenin-1 OS=Homo sapiens OX=9606 GN=SDCBP PE=1 SV=1	SDCB1_HUMAN	SDCBP	32 kDa			[illegible]
770	Proteasome subunit beta type-8 OS=Homo sapiens OX=9606 GN=PSMB8 PE=1 SV=3	PSB8_HUMAN	PSMB8	30 kDa	2	0	[illegible]
771	1-acyl-sn-glycerol-3-phosphate acyltransferase gamma OS=Homo sapiens OX=9606 GN=AGPAT3 PE=1 SV=1	PLCC_HUMAN	AGPAT3	43 kDa	1	1	[illegible]
772	40S ribosomal protein S5 OS=Homo sapiens OX=9606 GN=RPS5 PE=1 SV=4	RS5_HUMAN	RPS5	23 kDa	2	2	[illegible]
773	Immunoglobulin kappa variable 3-11 OS=Homo sapiens OX=9606 GN=IGKV3-11 PE=1 SV=1	KV311_HUMAN (+1)	IGKV3-11	13 kDa	0		[illegible]
774	Tether containing UBX domain for GLUT4 OS=Homo sapiens OX=9606 GN=ASPSCR1 PE=1 SV=1	ASPC1_HUMAN	ASPSCR1	60 kDa			[illegible]
775	CD82 antigen OS=Homo sapiens OX=9606 GN=CD82 PE=1 SV=1	CD82_HUMAN	CD82	30 kDa			[illegible]
776	Actin-related protein 2/3 complex subunit 1A OS=Homo sapiens OX=9606 GN=ARPC1A PE=2 SV=2	ARC1A_HUMAN	ARPC1A	42 kDa			[illegible]
777	Malate dehydrogenase, mitochondrial OS=Homo sapiens OX=9606 GN=MDH2 PE=1 SV=3	MDHM_HUMAN	MDH2	36 kDa	1		[illegible]
778	Unconventional myosin-Id OS=Homo sapiens OX=9606 GN=MYO1D PE=1 SV=2	MYO1D_HUMAN	MYO1D	116 kDa	1	2	[illegible]

779	Immunoglobulin heavy variable 3-7 OS=Homo sapiens OX=9606 GN=IGHV3-7 PE=1 SV=2	HV307_HUMAN	IGHV3-7	13 kDa		0			0		1	1		0		0	1	1	3	2	0	0	0					0		0	1	0		1									0	2	0	0		4	2	2	
780	H(+)/Cl(-) exchange transporter 3 OS=Homo sapiens OX=9606 GN=CLCN3 PE=1 SV=2	CLCN3_HUMAN	CLCN3	91 kDa	0										1	0	0	1			3	1	0	5	2	1	0	2				1										1	0			0	0		0	2	
781	ATP synthase subunit beta, mitochondrial OS=Homo sapiens OX=9606 GN=ATP5F1B PE=1 SV=3	ATPB_HUMAN	ATP5F1B	57 kDa	8		2			0			1			1	1										8			1	1	1					3					2				1					
782	V-type proton ATPase subunit S1 OS=Homo sapiens OX=9606 GN=ATP6AP1 PE=1 SV=2	VAS1_HUMAN	ATP6AP1	52 kDa		1	2				1					1	1	1	1		1	0	1	1	1		1				0						1					0			0	1	0			1	
783	Proteasomal ATPase-associated factor 1 OS=Homo sapiens OX=9606 GN=PAAF1 PE=1 SV=2	PAAF1_HUMAN	PAAF1	42 kDa					1												0	0			1	1				0	4	1					1	2	1	2	1	5	1		2	1	4				2
784	Apolipoprotein A-I OS=Homo sapiens OX=9606 GN=APOA1 PE=1 SV=1	APOA1_HUMAN	APOA1	31 kDa							0						2	1		0	2	1	0			2	0	0		3	1	0										1	0				4	2			
785	Thioredoxin OS=Homo sapiens OX=9606 GN=TXN PE=1 SV=3	THIO_HUMAN	TXN	12 kDa				0								1	1	1	1	1	1	1	1	1	1	2		2	1	1				1	1	1			0				1	1	1	0	1	1	1	1	2
786	Translation initiation factor eIF-2B subunit delta OS=Homo sapiens OX=9606 GN=EIF2B4 PE=1 SV=2	EI2BD_HUMAN	EIF2B4	58 kDa	2	0	2		2	1	2	1									2	2	1		1		1				1					0	1	1		2	1	1	1		1		2	1	1		1
787	60S acidic ribosomal protein P1 OS=Homo sapiens OX=9606 GN=RPLP1 PE=1 SV=1	RLA1_HUMAN	RPLP1	12 kDa	1	2			1		1			1	2	1	1	1	1		1	1	1			1					2				0		1			1		1	1	0	1	1	1				
788	60S ribosomal protein L3 OS=Homo sapiens OX=9606 GN=RPL3 PE=1 SV=2	RL3_HUMAN	RPL3	46 kDa		1	0		2		1	2	1					1															2		1	2	1					2	1				0		1		4
789	Receptor of activated protein C kinase 1 OS=Homo sapiens OX=9606 GN=RACK1 PE=1 SV=3	RACK1_HUMAN	RACK1	35 kDa														1	2		3				1					0	1					2						3	3	1	2		3		1		4
790	Immunoglobulin-binding protein 1 OS=Homo sapiens OX=9606 GN=IGBP1 PE=1 SV=1	IGBP1_HUMAN	IGBP1	39 kDa																		4	1		2		2				1											9	0				3				1
791	CTP synthase 1 OS=Homo sapiens OX=9606 GN=CTPS1 PE=1 SV=2	PYRG1_HUMAN	CTPS1	67 kDa																																						5	3				6	2			2
792	Voltage-gated potassium channel subunit beta-2 OS=Homo sapiens OX=9606 GN=KCNAB2 PE=1 SV=2	KCAB2_HUMAN	KCNAB2	41 kDa	1	2	1		0	1	0			0		1						1	2	0		0	2		1		1		1	0	0		1			1		1			2		0		1		0
793	C-C motif chemokine 14 OS=Homo sapiens OX=9606 GN=CCL14 PE=1 SV=1	CCL14_HUMAN	CCL14	11 kDa	1	1				1	2	1					1			2	1	3		2	1									0	1				0			0	2	2	2	1				2	1
794	Vacuolar protein-sorting-associated protein 36 OS=Homo sapiens OX=9606 GN=VPS36 PE=1 SV=1	VPS36_HUMAN	VPS36	44 kDa												1	0	1	1		1		1		2	1	0													2	1	2	1		1	3	1			2	1
795	Casein kinase I isoform gamma-1 OS=Homo sapiens OX=9606 GN=CSNK1G1 PE=1 SV=1	KC1G1_HUMAN	CSNK1G1	49 kDa	1	4	4				3			1	4												1	2	0				2	1	1		1			3	1		4	1				0		0	3
796	CDP-diacylglycerol--inositol 3-phosphatidyltransferase OS=Homo sapiens OX=9606 GN=CDIPT PE=1 SV=1	CDIPT_HUMAN	CDIPT	24 kDa		1	2	2		1			1	3	2	1					2						0	1				1			1		2	1		0	0	1				2					0
797	Immunoglobulin heavy variable 1-69 OS=Homo sapiens OX=9606 GN=IGHV1-69 PE=1 SV=2	HV169_HUMAN	IGHV1-69	13 kDa	0	1					3	2			2	0		0	1	1	1	0									0			1							1	1	1	2	1	2			3	2	
798	Galectin-8 OS=Homo sapiens OX=9606 GN=LGALS8 PE=1 SV=4	LEG8_HUMAN	LGALS8	36 kDa	2	2	1	1	3	0	1	2	0		3	0		0							0		1	1	1	1							0	0			1		0	0							0
799	GMP reductase 2 OS=Homo sapiens OX=9606 GN=GMPR2 PE=1 SV=1	GMPR2_HUMAN	GMPR2	38 kDa	0												1				2	2	2		2	1	2	0		1	3										1		0				7				
800	Solute carrier family 12 member 6 OS=Homo sapiens OX=9606 GN=SLC12A6 PE=1 SV=2	S12A6_HUMAN	SLC12A6	128 kDa	0		1									1					1			3	1			0		0													3				1	1	1	3	
801	Oxidoreductase HTATIP2 OS=Homo sapiens OX=9606 GN=HTATIP2 PE=1 SV=2	HTAI2_HUMAN	HTATIP2	27 kDa										0			0		1	0			0							1		0			0		0		1				1			1	1	2	0	1	
802	Immunoglobulin lambda variable 1-47 OS=Homo sapiens OX=9606 GN=IGLV1-47 PE=1 SV=2	LV147_HUMAN	IGLV1-47	12 kDa	2	2		1	1		2	2	1	1	2				6	1		2		1							1	1		1							2		2	1					2		
803	Apolipoprotein A-IV OS=Homo sapiens OX=9606 GN=APOA4 PE=1 SV=3	APOA4_HUMAN	APOA4	45 kDa	1	1	1	2	1	1	1	1	1	0		0			0		0	0			1			0	1				2			1	0		0	0					0				0		
804	60S ribosomal protein L4 OS=Homo sapiens OX=9606 GN=RPL4 PE=1 SV=5	RL4_HUMAN	RPL4	48 kDa								1						1					1			0										1	0					1	5		1				1		5
805	Epithelial membrane protein 3 OS=Homo sapiens OX=9606 GN=EMP3 PE=1 SV=1	EMP3_HUMAN	EMP3	18 kDa				1	2	1		0	0	0		1	3	0	0			0		1		0				0		0	0				0									0	0	0		0	
806	Keratin, type II cuticular Hb5 OS=Homo sapiens OX=9606 GN=KRT85 PE=1 SV=1	KRT85_HUMAN	KRT85	56 kDa				9						6			5									1							22																		
807	Immunoglobulin heavy variable 5-51 OS=Homo sapiens OX=9606 GN=IGHV5-51 PE=3 SV=1	HV551_HUMAN	IGHV5-51	13 kDa					1		1	2			1	2	0		3	2	3	1	1									1		2									2	2			0		4	1	
808	Protein argonaute-3 OS=Homo sapiens OX=9606 GN=AGO3 PE=1 SV=2	AGO3_HUMAN	AGO3	97 kDa			44											38	37				37	41		41		34		42	48	38			58		61	48		43	38				53	45		54		36	
809	Putative PIP5K1A and PSMD4-like protein OS=Homo sapiens OX=9606 GN=PIPSL PE=5 SV=1	PIPSL_HUMAN	PIPSL	95 kDa										2							3	6			4	2	3			2	4	3		3	1				1		2	3	6			2	3	2			
810	Ubiquitin-like modifier-activating enzyme ATG7 OS=Homo sapiens OX=9606 GN=ATG7 PE=1 SV=1	ATG7_HUMAN	ATG7	78 kDa	2				1	1	1		1								1	3				1				1	4	3					2			1		3	2				2				1
811	60S ribosomal protein L7 OS=Homo sapiens OX=9606 GN=RPL7 PE=1 SV=1	RL7_HUMAN	RPL7	29 kDa		1			1		3	2	1					1	1		1		1			1		0			2				1			1					1						2		4
812	Leucine-rich repeat-containing protein 59 OS=Homo sapiens OX=9606 GN=LRRC59 PE=1 SV=1	LRC59_HUMAN	LRRC59	35 kDa						1				0	1	2	1	1	1				0	0	0			1			0	0		1	0			1				0	1	1	0	0	1		1	2	
813	Protein EFR3 homolog A OS=Homo sapiens OX=9606 GN=EFR3A PE=1 SV=2	EFR3A_HUMAN	EFR3A	93 kDa			1		2				1					0	1	2												1			0	2		0	1		1			3	1			2			1
814	Mannose-1-phosphate guanyltransferase alpha OS=Homo sapiens OX=9606 GN=GMPPA PE=1 SV=1	GMPPA_HUMAN	GMPPA	46 kDa			1		1				1									0	0		1					1	4									1		7	2				3	0	0	0	2
815	Lys-63-specific deubiquitinase BRCC36 OS=Homo sapiens OX=9606 GN=BRCC3 PE=1 SV=2	BRCC3_HUMAN	BRCC3	36 kDa	1	2								1								1			1					0	3	0					1			0		5					7	1			1
816	Phosphoribosylformylglycinamidine synthase OS=Homo sapiens OX=9606 GN=PFAS PE=1 SV=4	PUR4_HUMAN	PFAS	145 kDa	3	1			1	2	0			1																1	7							1				3					2				0
817	D-aminoacyl-tRNA deacylase 1 OS=Homo sapiens OX=9606 GN=DTD1 PE=1 SV=2	DTD1_HUMAN	DTD1	23 kDa	1	1			1			1				1	1	1	1	1	1	2	1	1	1	2	1	1		1	1	0	1				1					1	1			0	0		1	2	
818	60S ribosomal protein L30 OS=Homo sapiens OX=9606 GN=RPL30 PE=1 SV=2	RL30_HUMAN	RPL30	13 kDa	0	1	1				1		1				0	1	1				1		1	1					2		1		2	2	1					1	1	1	2		1		1		2
819	ATP-binding cassette sub-family F member 1 OS=Homo sapiens OX=9606 GN=ABCF1 PE=1 SV=2	ABCF1_HUMAN	ABCF1	96 kDa	2	1	2		2	1	1	2	2	1																	1				1		2		1			2	3		1		3				2
820	40S ribosomal protein S7 OS=Homo sapiens OX=9606 GN=RPS7 PE=1 SV=1	RS7_HUMAN	RPS7	22 kDa		1			0		1	1									1					2					3				1		2	1			0	1	2	1	2		1		1		3
821	Transmembrane emp24 domain-containing protein 4 OS=Homo sapiens OX=9606 GN=TMED4 PE=1 SV=1	TMED4_HUMAN	TMED4	26 kDa			1				1				0				2								2	2	1	3					3	1	2	1	3	0			4	3		0	2	1		3	
822	BRISC and BRCA1-A complex member 2 OS=Homo sapiens OX=9606 GN=BABAM2 PE=1 SV=2	BABA2_HUMAN	BABAM2	44 kDa		1												1				1			1	1				0	4	1					0					4	1		1		3	0			0
823	Translin OS=Homo sapiens OX=9606 GN=TSN PE=1 SV=1	TSN_HUMAN	TSN	26 kDa	1						0											0									7	0					1					3	3				2				
824	Emerin OS=Homo sapiens OX=9606 GN=EMD PE=1 SV=1	EMD_HUMAN	EMD	29 kDa			0			1				0	1	1	1		1		1	0	0	0	1		2	2				1	1				1			1	1		0			1	1	1		1	
825	Ubiquitin-conjugating enzyme E2 L3 OS=Homo sapiens OX=9606 GN=UBE2L3 PE=1 SV=1	UB2L3_HUMAN	UBE2L3	18 kDa				1			1	0			0		1	1			0	1			1					2	2	2	0	2	1	1	1						1				0	2	1	2	
826	Condensin complex subunit 3 OS=Homo sapiens OX=9606 GN=NCAPG PE=1 SV=1	CND3_HUMAN	NCAPG	114 kDa	1						1														1	1	1				4	1			1						1	5	4				6	3			
827	Protein kinase C and casein kinase substrate in neurons protein 2 OS=Homo sapiens OX=9606 GN=PACSIN2 PE=1 SV=2	PACN2_HUMAN	PACSIN2	56 kDa	0			1	1		0		0	0	1										2		1					1		1			1			0		1			1		1		1		
828	Complement C1q subcomponent subunit B OS=Homo sapiens OX=9606 GN=C1QB PE=1 SV=3	C1QB_HUMAN		27 kDa												3	4	4	4	2	2	1	2	2			0																						2	2	
829	40S ribosomal protein S17 OS=Homo sapiens OX=9606 GN=RPS17 PE=1 SV=2	RS17_HUMAN	RPS17	16 kDa	1					0		1													2		1				3		0			0	2					3	2				2				3
830	60S ribosomal protein L9 OS=Homo sapiens OX=9606 GN=RPL9 PE=1 SV=1	RL9_HUMAN	RPL9	22 kDa												2	1	1	1		1	0		0	1	1		0		0													3	0			0	0	1	0	
831	Immunoglobulin delta heavy chain OS=Homo sapiens OX=9606 PE=1 SV=1	IGD_HUMAN		56 kDa																							0					1		7	1								3	10							
832	Poly(rC)-binding protein 1 OS=Homo sapiens OX=9606 GN=PCBP1 PE=1 SV=2	PCBP1_HUMAN	PCBP1	37 kDa												2	2	1	1	1	1	1	1	1	2	1		1								2	2					1					0		0	1	2
833	F-box/LRR-repeat protein 20 OS=Homo sapiens OX=9606 GN=FBXL20 PE=1 SV=2	FXL20_HUMAN	FBXL20	48 kDa						1		1	1	1	3	1	1		1		1	1				1				1		0	2				2						1	0	1						
834	Glutamate--cysteine ligase regulatory subunit OS=Homo sapiens OX=9606 GN=GCLM PE=1 SV=1	GSH0_HUMAN	GCLM	31 kDa	2	1			2		1							1			1	1	1	1	2					1											1	4	0							3	3
835	Syntaxin-18 OS=Homo sapiens OX=9606 GN=STX18 PE=1 SV=1	STX18_HUMAN	STX18	39 kDa	0						0	1				1			1									0		2	1	1	1			0	1	1					1	0	1	1		1			2
836	Protein S100-A6 OS=Homo sapiens OX=9606 GN=S100A6 PE=1 SV=1	S10A6_HUMAN	S100A6	10 kDa		1									2	2	2	1	0	0	1	1	0		0	0	0	0		0													0	0			0	0	0	1	
837	Protein canopy homolog 4 OS=Homo sapiens OX=9606 GN=CNPY4 PE=2 SV=1	CNPY4_HUMAN	CNPY4	28 kDa	2	2	4		0	0	1	1	2		2	0		0													1	1		1					1	1		1									
838	Casein kinase II subunit beta OS=Homo sapiens OX=9606 GN=CSNK2B PE=1 SV=1	CSK2B_HUMAN	CSNK2B	25 kDa		1															1	2			2	1					1				1		1			1		3	2				2				1
839	Ubiquitin carboxyl-terminal hydrolase 47 OS=Homo sapiens OX=9606 GN=USP47 PE=1 SV=3	UBP47_HUMAN	USP47	157 kDa																			0		2						1	1								0	0	10					2				
840	ADP-ribose glycohydrolase OARD1 OS=Homo sapiens OX=9606 GN=OARD1 PE=1 SV=2	OARD1_HUMAN	OARD1	17 kDa					1				0			1	1	1	1	0	0	1	1	1	1	1	1	1		1											1		1				1	1			
841	Elongin-C OS=Homo sapiens OX=9606 GN=ELOC PE=1 SV=1	ELOC_HUMAN	ELOC	12 kDa	2	1					1																			0	3							1		1		3	2			1	2	1			2
842	Serine/threonine-protein kinase MARK2 OS=Homo sapiens OX=9606 GN=MARK2 PE=1 SV=2	MARK2_HUMAN	MARK2	88 kDa																		2		1				2		1				1		2	3			2			4	1	2		3	2	1	2	
843	Keratin, type I cytoskeletal 17 OS=Homo sapiens OX=9606 GN=KRT17 PE=1 SV=2	K1C17_HUMAN	KRT17	48 kDa	3				2	3		4			6		13																							3			4								
844	Major facilitator superfamily domain-containing protein 2B OS=Homo sapiens OX=9606 GN=MFSD2B PE=1 SV=4	MFS2B_HUMAN	MFSD2B	54 kDa	1	2		1	1	2			2		2		0	2						1	0	1		1	2					1			1	1		2		1								1	
845	Rab-interacting lysosomal protein OS=Homo sapiens OX=9606 GN=RILP PE=1 SV=1	RILP_HUMAN	RILP	44 kDa		1																			1						6						2				1	6	1		1		3				2
846	Beta-centractin OS=Homo sapiens OX=9606 GN=ACTR1B PE=1 SV=1	ACTY_HUMAN	ACTR1B	42 kDa		7								5			12					8				12	9					3									8					5					
847	Protein SLFN14 OS=Homo sapiens OX=9606 GN=SLFN14 PE=1 SV=2	SLN14_HUMAN	SLFN14	104 kDa					1																						0									1		3	4	0	2		1				6
848	60S ribosomal protein L22 OS=Homo sapiens OX=9606 GN=RPL22 PE=1 SV=2	RL22_HUMAN	RPL22	15 kDa		1	1				1						1	1	1				1			1		1			2					2	1					1	3				1	1	1	0	1
849	Heterogeneous nuclear ribonucleoprotein A3 OS=Homo sapiens OX=9606 GN=HNRNPA3 PE=1 SV=2	ROA3_HUMAN	HNRNPA3	40 kDa	0				1		1	1			0				0			1	0								0									1		0	1	1				1			1
850	Hemoglobin subunit theta-1 OS=Homo sapiens OX=9606 GN=HBQ1 PE=1 SV=2	HBAT_HUMAN	HBQ1	16 kDa									2					5	1						3					1	2						1					2				2		2			
851	Deubiquitinating protein VCIP135 OS=Homo sapiens OX=9606 GN=VCPIP1 PE=1 SV=2	VCIP1_HUMAN	VCPIP1	134 kDa																					1						2	2			1					2		5			1	1	5				
852	Spectrin beta chain, non-erythrocytic 4 OS=Homo sapiens OX=9606 GN=SPTBN4 PE=1 SV=2	SPTN4_HUMAN	SPTBN4	289 kDa			14			11			10	7								4		5				5																8		10					5
853	Myosin light chain 3 OS=Homo sapiens OX=9606 GN=MYL3 PE=1 SV=3	MYL3_HUMAN	MYL3	22 kDa			0															3	3					2	2		2							2				1	4				2		1		2
854	Insulin-degrading enzyme OS=Homo sapiens OX=9606 GN=IDE PE=1 SV=4	IDE_HUMAN	IDE	118 kDa	2						0			0											0						5	0										6					2				
855	Secretory carrier-associated membrane protein 2 OS=Homo sapiens OX=9606 GN=SCAMP2 PE=1 SV=2	SCAM2_HUMAN	SCAMP2	37 kDa												2		2	2		2	1			0	1	1	1			1	2			1					1					1	1	1	1		0	
856	Programmed cell death 6-interacting protein OS=Homo sapiens OX=9606 GN=PDCD6IP PE=1 SV=1	PDC6I_HUMAN	PDCD6IP	96 kDa	1											1	1	0	1		1	2	1	1	1															1		1	1		1		2	1		0	2
857	Interferon-stimulated gene 20 kDa protein OS=Homo sapiens OX=9606 GN=ISG20 PE=1 SV=2	ISG20_HUMAN	ISG20	20 kDa												1			1	1		1	1	1	1	1	2	0	0	1					1						0			1			0			1	0
858	TBC1 domain family member 10B OS=Homo sapiens OX=9606 GN=TBC1D10B PE=1 SV=3	TB10B_HUMAN	TBC1D10B	87 kDa													1			1	1	0	1	1	0	1		1	1	1													2				1	2	2		
859	V-type proton ATPase subunit D OS=Homo sapiens OX=9606 GN=ATP6V1D PE=1 SV=1	VATD_HUMAN	ATP6V1D	28 kDa	1					1											0	0			2	1	2			0	4						1	1				1					3				1
860	Signal peptidase complex subunit 3 OS=Homo sapiens OX=9606 GN=SPCS3 PE=1 SV=1	SPCS3_HUMAN	SPCS3	20 kDa												2		2	1	0	3	0		2	1		3	2																		1				2	
861	Eukaryotic peptide chain release factor subunit 1 OS=Homo sapiens OX=9606 GN=ETF1 PE=1 SV=3	ERF1_HUMAN	ETF1	49 kDa	1																				1	2					1				1					0		2	3		1		3	1			1
862	BRISC and BRCA1-A complex member 1 OS=Homo sapiens OX=9606 GN=BABAM1 PE=1 SV=1	BABA1_HUMAN	BABAM1	37 kDa		0																									4	1				1	1	1		1	2	4				0	4				2
863	Keratin, type II cuticular Hb4 OS=Homo sapiens OX=9606 GN=KRT84 PE=2 SV=2	KRT84_HUMAN	KRT84	65 kDa																										22																					
864	E3 ubiquitin-protein ligase rififylin OS=Homo sapiens OX=9606 GN=RFFL PE=1 SV=1	RFFL_HUMAN	RFFL	41 kDa	1	1	2	1	1	1	2	2		1	1			1			1		1	3			1	1								1														1	
865	Vitamin K epoxide reductase complex subunit 1-like protein 1 OS=Homo sapiens OX=9606 GN=VKORC1L1 PE=1 SV=2	VKORL_HUMAN	VKORC1L1	20 kDa	1		1	0			2	1	1	1	2	1		1	1	1	1					1											1									1	1			2	
866	PDZK1-interacting protein 1 OS=Homo sapiens OX=9606 GN=PDZK1IP1 PE=1 SV=1	PDZ1I_HUMAN	PDZK1IP1	12 kDa	1				2	1		1	1	1	2								0			0			1				0	1		1	1	1		2	1	0		2							
867	Ras-related protein Rab-11B OS=Homo sapiens OX=9606 GN=RAB11B PE=1 SV=4	RB11B_HUMAN	RAB11B	24 kDa	0												1	1	0				1	1			0	1	0		1	1	1		1	1	0		1	1		1							0		
868	Coxsackievirus and adenovirus receptor OS=Homo sapiens OX=9606 GN=CXADR PE=1 SV=1	CXAR_HUMAN	CXADR	40 kDa		1	2		0	1	1	1	1	0	1	0		1										0										0		0											
869	Transmembrane protein 237 OS=Homo sapiens OX=9606 GN=TMEM237 PE=1 SV=2	TM237_HUMAN	TMEM237	46 kDa	0	2	2	0			1	0		0											0		0	0		0			1										1	1	1	1	0				1
870	Transmembrane 9 superfamily member 4 OS=Homo sapiens OX=9606 GN=TM9SF4 PE=1 SV=2	TM9S4_HUMAN	TM9SF4	75 kDa																								0	2	0	0				0				0	0	0	0	1	2	2	1	0	0		2	
871	Calcium load-activated calcium channel OS=Homo sapiens OX=9606 GN=TMCO1 PE=1 SV=2	TMCO1_HUMAN	TMCO1	27 kDa	1	1	1				2					1	2	1	1	1	1			0	1	1	1	1		1																				2	
872	Transmembrane protein with metallophosphoesterase domain OS=Homo sapiens OX=9606 GN=TMPPE PE=2 SV=2	TMPPE_HUMAN	TMPPE	49 kDa	1		1		0		1	0			1		1	0				1		1			0	1								1	2	2			0	1								1	2
873	Signal peptidase complex catalytic subunit SEC11A OS=Homo sapiens OX=9606 GN=SEC11A PE=1 SV=1	SC11A_HUMAN	SEC11A	21 kDa	2		1				1			1	1	1			1		1				1		2					1				0				1						1				1	
874	E3 ubiquitin-protein ligase TRIM21 OS=Homo sapiens OX=9606 GN=TRIM21 PE=1 SV=1	RO52_HUMAN	TRIM21	54 kDa												1	1	2				0	1		1			1			1											1	2	1		1	0	1	1	2	0
875	Reticulophagy regulator 2 OS=Homo sapiens OX=9606 GN=RETREG2 PE=1 SV=3	RETR2_HUMAN	RETREG2	58 kDa																									1		2					1		2		1		1	1	1	0	1			1		1
876	ADP-ribosyl cyclase/cyclic ADP-ribose hydrolase 1 OS=Homo sapiens OX=9606 GN=CD38 PE=1 SV=2	CD38_HUMAN	CD38	34 kDa							1		0	0	1		0	1		1	1				0	0				1		0		0	1						0	1	2							0	
877	Microsomal glutathione S-transferase 3 OS=Homo sapiens OX=9606 GN=MGST3 PE=1 SV=1	MGST3_HUMAN	MGST3	17 kDa	3					1						0		3			0	0		1	1		4			1	0	1							1							0		0			
878	Ras GTPase-activating protein-binding protein 1 OS=Homo sapiens OX=9606 GN=G3BP1 PE=1 SV=1	G3BP1_HUMAN	G3BP1	52 kDa												1					1				1	1					1											3	4		1		3				1
879	FAS-associated factor 1 OS=Homo sapiens OX=9606 GN=FAF1 PE=1 SV=2	FAF1_HUMAN	FAF1	74 kDa		0					0											1			1	0					2	1					1	1				4					1				
880	Peptidyl-prolyl cis-trans isomerase FKBP4 OS=Homo sapiens OX=9606 GN=FKBP4 PE=1 SV=3	FKBP4_HUMAN	FKBP4	52 kDa																		1									6											9					4				
881	Polypeptide N-acetylgalactosaminyltransferase 6 OS=Homo sapiens OX=9606 GN=GALNT6 PE=2 SV=2	GALT6_HUMAN	GALNT6	71 kDa	0		2			1			1						0						1						1	1			1	1	1	1		1		1				1		1			2
882	Keratin, type II cytoskeletal 6C OS=Homo sapiens OX=9606 GN=KRT6C PE=1 SV=3	K2C6C_HUMAN	KRT6C	60 kDa											24		70			29		16			27			26		33										25			26								
883	Ubiquitin carboxyl-terminal hydrolase 24 OS=Homo sapiens OX=9606 GN=USP24 PE=1 SV=3	UBP24_HUMAN	USP24	294 kDa	3													0				1									3						1					4					2				
884	Peflin OS=Homo sapiens OX=9606 GN=PEF1 PE=1 SV=1	PEF1_HUMAN	PEF1	30 kDa								1				1	1	1							1							3								1		1	1	1		1	1	2	1	1	1
885	Calcium-regulated heat-stable protein 1 OS=Homo sapiens OX=9606 GN=CARHSP1 PE=1 SV=2	CHSP1_HUMAN	CARHSP1	16 kDa																		1		1	1	1				1	1						0				0	0	2		0	0	0	1		1	
886	Eukaryotic initiation factor 4A-I OS=Homo sapiens OX=9606 GN=EIF4A1 PE=1 SV=1	IF4A1_HUMAN	EIF4A1	46 kDa													2													2	1				1				1			3	2				0	1		1	0
887	Protein DPCD OS=Homo sapiens OX=9606 GN=DPCD PE=1 SV=2	DPCD_HUMAN	DPCD	23 kDa	1						1											0				0					2	1				0	0				1	4	1				1				2
888	Guanine nucleotide exchange factor for Rab-3A OS=Homo sapiens OX=9606 GN=RAB3IL1 PE=1 SV=1	R3GEF_HUMAN	RAB3IL1	43 kDa	1																		0								1						1			1		3	2		1		4				3
889	Coiled-coil domain-containing protein 6 OS=Homo sapiens OX=9606 GN=CCDC6 PE=1 SV=2	CCDC6_HUMAN	CCDC6	53 kDa																										1	1				1		1					2	0		1		1	0			2
890	Endoplasmic reticulum-Golgi intermediate compartment protein 3 OS=Homo sapiens OX=9606 GN=ERGIC3 PE=1 SV=1	ERGI3_HUMAN	ERGIC3	43 kDa																						1		1		1	4											0		1		1	3	2	2		
891	MARVEL domain-containing protein 2 OS=Homo sapiens OX=9606 GN=MARVELD2 PE=1 SV=2	MALD2_HUMAN	MARVELD2	64 kDa													1			0		0	1					1				0			2					1		1	3						1		
892	Hypoxanthine-guanine phosphoribosyltransferase OS=Homo sapiens OX=9606 GN=HPRT1 PE=1 SV=2	HPRT_HUMAN	HPRT1	25 kDa	1																				1					2	4											4	2				3				0
893	Dolichyl-diphosphooligosaccharide--protein glycosyltransferase 48 kDa subunit OS=Homo sapiens OX=9606 GN=DDOST PE=1 SV=4	OST48_HUMAN	DDOST	51 kDa				1					1	2	1	1		1	0					1		1		1												0						1		1		0	
894	Haptoglobin OS=Homo sapiens OX=9606 GN=HP PE=1 SV=1	HPT_HUMAN	HP	45 kDa							2			1			1														1	1		0								2			1	2	3	2			
895	B-cell receptor-associated protein 29 OS=Homo sapiens OX=9606 GN=BCAP29 PE=1 SV=2	BAP29_HUMAN	BCAP29	28 kDa												1	1	1		0		1	0	1			1	0				0									1					1				1	
896	NEDD8 OS=Homo sapiens OX=9606 GN=NEDD8 PE=1 SV=1	NEDD8_HUMAN	NEDD8	9 kDa	3	1	1				3	0													1					1	1	1			1								0				1				
897	RAD50-interacting protein 1 OS=Homo sapiens OX=9606 GN=RINT1 PE=1 SV=1	RINT1_HUMAN	RINT1	91 kDa												1			3		1			0		0				1	0				1								2		0			2	0	1	
898	Clathrin interactor 1 OS=Homo sapiens OX=9606 GN=CLINT1 PE=1 SV=1	EPN4_HUMAN	CLINT1	68 kDa												1		1	1	2	3		1	0	1	1	0																0					0		2	
899	Eukaryotic translation initiation factor 4E OS=Homo sapiens OX=9606 GN=EIF4E PE=1 SV=2	IF4E_HUMAN	EIF4E	25 kDa	0		1		0		2																					1					1				1	5			0						3
900	Coronin-1C OS=Homo sapiens OX=9606 GN=CORO1C PE=1 SV=1	COR1C_HUMAN	CORO1C	53 kDa																											1				2	0						1	2	3	1		1				5
901	Dynamin-3 OS=Homo sapiens OX=9606 GN=DNM3 PE=1 SV=4	DYN3_HUMAN	DNM3	98 kDa													10	9					12		9		10															8	7		6		9			16	
902	Charged multivesicular body protein 4b OS=Homo sapiens OX=9606 GN=CHMP4B PE=1 SV=1	CHM4B_HUMAN	CHMP4B	25 kDa												0	2	3			0	0		1		1														1			1							0	0
903	DDB1- and CUL4-associated factor 11 OS=Homo sapiens OX=9606 GN=DCAF11 PE=1 SV=1	DCA11_HUMAN	DCAF11	62 kDa	4																				0		1				1	0										3					2	0			0
904	Vacuolar protein sorting-associated protein 4A OS=Homo sapiens OX=9606 GN=VPS4A PE=1 SV=1	VPS4A_HUMAN	VPS4A	49 kDa			0									1		1					3							0												1					0			3	1
905	Vacuolar protein sorting-associated protein VTA1 homolog OS=Homo sapiens OX=9606 GN=VTA1 PE=1 SV=1	VTA1_HUMAN	VTA1	34 kDa																							0				2		0					0			0	3			0		2				
906	Serine/threonine-protein kinase 10 OS=Homo sapiens OX=9606 GN=STK10 PE=1 SV=1	STK10_HUMAN	STK10	112 kDa												1				1	1	1				1	1			1	1				2								1	2		2	2	2	1		
907	E3 ubiquitin-protein ligase MARCH8 OS=Homo sapiens OX=9606 GN=MARCH8 PE=1 SV=1	MARH8_HUMAN	8-Mar	33 kDa									1				2	1	1			1	1			0		1				0				1			0		1				1	1					
908	Regulatory-associated protein of mTOR OS=Homo sapiens OX=9606 GN=RPTOR PE=1 SV=1	RPTOR_HUMAN	RPTOR	149 kDa	2																										2						1	1		1		3	0				3				1
909	C-C chemokine receptor-like 2 OS=Homo sapiens OX=9606 GN=CCRL2 PE=1 SV=2	CCRL2_HUMAN	CCRL2	40 kDa	0	0			1		0		0		1		2			0						1	0	1		0																				0	
910	Casein kinase II subunit alpha' OS=Homo sapiens OX=9606 GN=CSNK2A2 PE=1 SV=1	CSK22_HUMAN	CSNK2A2	41 kDa	0						1			0																	2						1					2					4	1			
911	DNA repair protein RAD50 OS=Homo sapiens OX=9606 GN=RAD50 PE=1 SV=1	RAD50_HUMAN	RAD50	154 kDa																											2						1					4			1		6	0			1
912	Fibrinogen alpha chain OS=Homo sapiens OX=9606 GN=FGA PE=1 SV=2	FIBA_HUMAN	FGA	95 kDa												0	8	2													1																1				
913	ADP-dependent glucokinase OS=Homo sapiens OX=9606 GN=ADPGK PE=1 SV=1	ADPGK_HUMAN	ADPGK	54 kDa	2			1		2		1	0	0		1			1		1											1														1				1	
914	Heterogeneous nuclear ribonucleoprotein K OS=Homo sapiens OX=9606 GN=HNRNPK PE=1 SV=1	HNRPK_HUMAN	HNRNPK	51 kDa																	1				1	0				1	1											4	2		0		2	2			
915	DDB1- and CUL4-associated factor 6 OS=Homo sapiens OX=9606 GN=DCAF6 PE=1 SV=1	DCAF6_HUMAN	DCAF6	96 kDa	1				0		1														1	1	0				1									1		2					2				
916	60 kDa heat shock protein, mitochondrial OS=Homo sapiens OX=9606 GN=HSPD1 PE=1 SV=2	CH60_HUMAN	HSPD1	61 kDa	5		0						1	1		1		0									3			0											0										
917	Exportin-1 OS=Homo sapiens OX=9606 GN=XPO1 PE=1 SV=1	XPO1_HUMAN	XPO1	123 kDa	3																										2											1					1			1	
918	Nardilysin OS=Homo sapiens OX=9606 GN=NRDC PE=1 SV=3	NRDC_HUMAN	NRDC	132 kDa																			0								1						2					5	1				1				
919	Claudin domain-containing protein 1 OS=Homo sapiens OX=9606 GN=CLDND1 PE=1 SV=1	CLDN1_HUMAN	CLDND1	29 kDa		0													2	2	1				1	1	1	1		0								1								2	1			1	
920	Glycogen [starch] synthase, muscle OS=Homo sapiens OX=9606 GN=GYS1 PE=1 SV=2	GYS1_HUMAN	GYS1	84 kDa	1	1					1			0	1													1		0	1		0				0					3					2				1
921	60S ribosomal protein L10 OS=Homo sapiens OX=9606 GN=RPL10 PE=1 SV=4	RL10_HUMAN	RPL10	25 kDa															0				0			1									1		1					1	3		1		2		1		3
922	28 kDa heat- and acid-stable phosphoprotein OS=Homo sapiens OX=9606 GN=PDAP1 PE=1 SV=1	HAP28_HUMAN	PDAP1	21 kDa																			2				1				2						1					1	2				1		0	1	3
923	Golgi to ER traffic protein 4 homolog OS=Homo sapiens OX=9606 GN=GET4 PE=1 SV=1	GET4_HUMAN	GET4	37 kDa																		1				0					2	0					1	1			1	1	1				1	0	1		0
924	Protein S100-A9 OS=Homo sapiens OX=9606 GN=S100A9 PE=1 SV=1	S10A9_HUMAN	S100A9	13 kDa	2	1		1							3		1	0										1															2		2						
925	Septin-11 OS=Homo sapiens OX=9606 GN=SEPT11 PE=1 SV=3	SEP11_HUMAN	11-Sep	49 kDa														1			2		2								1				5							1	4				2				
926	Kinesin-1 heavy chain OS=Homo sapiens OX=9606 GN=KIF5B PE=1 SV=1	KINH_HUMAN	KIF5B	110 kDa					1		0																				2								0			4					1				
927	N-terminal kinase-like protein OS=Homo sapiens OX=9606 GN=SCYL1 PE=1 SV=1	SCYL1_HUMAN	SCYL1	90 kDa																					0						2											5	1				2				
928	Ras-related protein Rab-22A OS=Homo sapiens OX=9606 GN=RAB22A PE=1 SV=2	RB22A_HUMAN	RAB22A	22 kDa																									5											1									7		
929	Dynactin subunit 5 OS=Homo sapiens OX=9606 GN=DCTN5 PE=1 SV=1	DCTN5_HUMAN	DCTN5	20 kDa										1			1	0			1	1	1		1	1		2				1									1		1			0					
930	Elongin-B OS=Homo sapiens OX=9606 GN=ELOB PE=1 SV=1	ELOB_HUMAN	ELOB	13 kDa																					1		1			1	1	1					1	1		1		3				0	2				
931	Triosephosphate isomerase OS=Homo sapiens OX=9606 GN=TPI1 PE=1 SV=3	TPIS_HUMAN	TPI1	31 kDa		1			1	0		1					0	1				1	1	0	1			1			1	0																		1	0
932	Thioredoxin reductase-like selenoprotein T OS=Homo sapiens OX=9606 GN=SELENOT PE=1 SV=2	SELT_HUMAN	SELENOT	22 kDa										1	1	1	1		0					2			1					1			1			0				0				2				1	0
933	Guanine nucleotide-binding protein G(i) subunit alpha-1 OS=Homo sapiens OX=9606 GN=GNAI1 PE=1 SV=2	GNAI1_HUMAN	GNAI1	40 kDa							34			24			15				40						18							31	43	33					30			44							
934	Actin-related protein 2/3 complex subunit 4 OS=Homo sapiens OX=9606 GN=ARPC4 PE=1 SV=3	ARPC4_HUMAN	ARPC4	20 kDa								1				1			0											1											0	1	1		0		1	2		1	1

935	Vitronectin OS=Homo sapiens OX=9606 GN=VTN PE=1 SV=1	VTNC_HUMAN	VTN	54 kDa											0	1		1		0	2				0					2																	1		0	1	2	
936	Biliverdin reductase A OS=Homo sapiens OX=9606 GN=BLVRA PE=1 SV=2	BIEA_HUMAN	BLVRA	33 kDa							0		2				1	1					2							2		0																1			0	1
937	Eukaryotic translation initiation factor 2D OS=Homo sapiens OX=9606 GN=EIF2D PE=1 SV=3	EIF2D_HUMAN	EIF2D	65 kDa	0		2	1			1																				1		1			0		1				2		0			0				1
938	Fumarate hydratase, mitochondrial OS=Homo sapiens OX=9606 GN=FH PE=1 SV=3	FUMH_HUMAN	FH	55 kDa	2						1																				4	1										1					0				
939	Immunoglobulin alpha-2 heavy chain OS=Homo sapiens OX=9606 PE=1 SV=2	IGA2_HUMAN		49 kDa															2	6												5							1					8					3	6	
940	Keratin, type I cuticular Ha3-II OS=Homo sapiens OX=9606 GN=KRT33B PE=1 SV=3	KT33B_HUMAN	KRT33B	46 kDa				12						6																			11																		
941	Lupus La protein OS=Homo sapiens OX=9606 GN=SSB PE=1 SV=2	LA_HUMAN	SSB	47 kDa	1		0															2			2	1	1			1	2											2	1				1				
942	Coatomer subunit gamma-1 OS=Homo sapiens OX=9606 GN=COPG1 PE=1 SV=1	COPG1_HUMAN	COPG1	98 kDa	1				1																						1						1					2	2		1		2	0			2
943	40S ribosomal protein S4, X isoform OS=Homo sapiens OX=9606 GN=RPS4X PE=1 SV=2	RS4X_HUMAN	RPS4X	30 kDa																					2	2					1											1	3		1		2		1		1
944	Vacuolar-sorting protein SNF8 OS=Homo sapiens OX=9606 GN=SNF8 PE=1 SV=1	SNF8_HUMAN	SNF8	29 kDa													0				0	2			1	1	2													1		1	1				1	1			
945	Oxysterol-binding protein 1 OS=Homo sapiens OX=9606 GN=OSBP PE=1 SV=1	OSBP1_HUMAN	OSBP	89 kDa					2									1	1				1	0				0			0	1														1	0			1	
946	Rab-like protein 6 OS=Homo sapiens OX=9606 GN=RABL6 PE=1 SV=2	RABL6_HUMAN	RABL6	80 kDa	1																										1						1	1		2		2	1				3				
947	Elongation factor 1-delta OS=Homo sapiens OX=9606 GN=EEF1D PE=1 SV=5	EF1D_HUMAN	EEF1D	31 kDa																											2						1			4		3	3				2				
948	Eukaryotic translation initiation factor 3 subunit J OS=Homo sapiens OX=9606 GN=EIF3J PE=1 SV=2	EIF3J_HUMAN	EIF3J	29 kDa	2						1																				2	0								0		5					1				
949	Synembryn-A OS=Homo sapiens OX=9606 GN=RIC8A PE=1 SV=3	RIC8A_HUMAN	RIC8A	60 kDa																	0		1			1																1	2				0	0		1	
950	40S ribosomal protein SA OS=Homo sapiens OX=9606 GN=RPSA PE=1 SV=4	RSSA_HUMAN	RPSA	33 kDa												1		1			1				1	2					1					0							1				1	1	2		1
951	Acetolactate synthase-like protein OS=Homo sapiens OX=9606 GN=ILVBL PE=1 SV=2	ILVBL_HUMAN	ILVBL	68 kDa							1	1							1												2						1	1		1		2					1		1		
952	Synaptogyrin-1 OS=Homo sapiens OX=9606 GN=SYNGR1 PE=1 SV=2	SNG1_HUMAN	SYNGR1	25 kDa																											1	0			1	1							0	1	1	1	2	2			0
953	Importin subunit alpha-4 OS=Homo sapiens OX=9606 GN=KPNA3 PE=1 SV=2	IMA4_HUMAN	KPNA3	58 kDa																						1					1	2								1		3	2		1		2				
954	60S ribosomal protein L11 OS=Homo sapiens OX=9606 GN=RPL11 PE=1 SV=2	RL11_HUMAN	RPL11	20 kDa																					1						1				2							3	0		1		0			1	2
955	Gelsolin OS=Homo sapiens OX=9606 GN=GSN PE=1 SV=1	GELS_HUMAN	GSN	86 kDa															1		1		1		1	1																		1						4	
956	Aldehyde dehydrogenase family 3 member B1 OS=Homo sapiens OX=9606 GN=ALDH3B1 PE=1 SV=1	AL3B1_HUMAN	ALDH3B1	52 kDa					0			1		0					1		0		1									1													1	2		0			
957	Folate transporter 1 OS=Homo sapiens OX=9606 GN=SLC19A1 PE=1 SV=3	S19A1_HUMAN	SLC19A1	65 kDa		1			1				0		1	0	0	0					0			2		2									0														
958	Structural maintenance of chromosomes protein 4 OS=Homo sapiens OX=9606 GN=SMC4 PE=1 SV=2	SMC4_HUMAN	SMC4	147 kDa																	0					1					1											2	3				1				
959	Eukaryotic translation initiation factor 2 subunit 2 OS=Homo sapiens OX=9606 GN=EIF2S2 PE=1 SV=2	IF2B_HUMAN	EIF2S2	38 kDa	1	0																									1	1					1					3					2				
960	Protein FAM114A2 OS=Homo sapiens OX=9606 GN=FAM114A2 PE=1 SV=4	F1142_HUMAN	FAM114A2	55 kDa						0												1			1						2											3	0				3				
961	Protein SETSIP OS=Homo sapiens OX=9606 GN=SETSIP PE=1 SV=1	SETLP_HUMAN	SETSIP	35 kDa	0																									1	0									0			1				1	1			0
962	Serine/threonine-protein kinase mTOR OS=Homo sapiens OX=9606 GN=MTOR PE=1 SV=1	MTOR_HUMAN	MTOR	289 kDa																						1				0												2	0				2				1
963	Xaa-Pro dipeptidase OS=Homo sapiens OX=9606 GN=PEPD PE=1 SV=3	PEPD_HUMAN	PEPD	55 kDa	0				0																						3	1										1					0				
964	Heat shock protein 105 kDa OS=Homo sapiens OX=9606 GN=HSPH1 PE=1 SV=1	HS105_HUMAN	HSPH1	97 kDa																											3											2	0				4				
965	Pleckstrin homology domain-containing family F member 2 OS=Homo sapiens OX=9606 GN=PLEKHF2 PE=1 SV=1	PKHF2_HUMAN	PLEKHF2	28 kDa			1				1									1		1					1	1		0	1				1							1		1			1	1			
966	Complement C1r subcomponent OS=Homo sapiens OX=9606 GN=C1R PE=1 SV=2	C1R_HUMAN	C1R	80 kDa								1			2	0			2	1												1																		1	
967	Transcriptional repressor NF-X1 OS=Homo sapiens OX=9606 GN=NFX1 PE=1 SV=2	NFX1_HUMAN	NFX1	124 kDa																		1					1	1	0													1		1	1						
968	Phosphorylase b kinase regulatory subunit alpha, liver isoform OS=Homo sapiens OX=9606 GN=PHKA2 PE=1 SV=1	KPB2_HUMAN	PHKA2	138 kDa																											0				1							3	1				4				
969	PQ-loop repeat-containing protein 1 OS=Homo sapiens OX=9606 GN=PQLC1 PE=1 SV=1	PQLC1_HUMAN	PQLC1	30 kDa												1					0				0	0		0			0	0										0	0							0	
970	Protein YIF1A OS=Homo sapiens OX=9606 GN=YIF1A PE=1 SV=2	YIF1A_HUMAN	YIF1A	32 kDa																								2			1	1			1					2	1	1			1						
971	Solute carrier family 22 member 23 OS=Homo sapiens OX=9606 GN=SLC22A23 PE=1 SV=2	S22AN_HUMAN	SLC22A23	74 kDa						0							1	1			0		1	2					0										1							0					
972	Putative heat shock protein HSP 90-beta 4 OS=Homo sapiens OX=9606 GN=HSP90AB4P PE=5 SV=1	H90B4_HUMAN	HSP90AB4P	58 kDa										4							3				5						12											18					12				
973	60S ribosomal protein L6 OS=Homo sapiens OX=9606 GN=RPL6 PE=1 SV=3	RL6_HUMAN	RPL6	33 kDa														1	0												0				2		0					1	3		0						1
974	Plexin-A1 OS=Homo sapiens OX=9606 GN=PLXNA1 PE=1 SV=3	PLXA1_HUMAN	PLXNA1	211 kDa			0		2		1				0			0			1		1		1																										
975	Sorting nexin-22 OS=Homo sapiens OX=9606 GN=SNX22 PE=1 SV=1	SNX22_HUMAN	SNX22	22 kDa																	1	1			0	0	0			0												1					0	0			
976	Cyclin-D1-binding protein 1 OS=Homo sapiens OX=9606 GN=CCNDBP1 PE=1 SV=2	CCDB1_HUMAN	CCNDBP1	40 kDa																											2											4	1				1				
977	Cysteine and histidine-rich domain-containing protein 1 OS=Homo sapiens OX=9606 GN=CHORDC1 PE=1 SV=2	CHRD1_HUMAN	CHORDC1	37 kDa																											2											3	2								1
978	Microtubule-associated serine/threonine-protein kinase 1 OS=Homo sapiens OX=9606 GN=MAST1 PE=1 SV=2	MAST1_HUMAN	MAST1	171 kDa									3			0		3						0			1																								
979	Leucine-rich repeat-containing protein 75A OS=Homo sapiens OX=9606 GN=LRRC75A PE=2 SV=2	LR75A_HUMAN	LRRC75A	38 kDa			1			1	1				1					1								0		1									1		1							1			
980	Beta-2-microglobulin OS=Homo sapiens OX=9606 GN=B2M PE=1 SV=1	B2MG_HUMAN	B2M	14 kDa				1				1	1				1		1	1	0		1	1						0																					
981	Zinc finger CCCH-type antiviral protein 1-like OS=Homo sapiens OX=9606 GN=ZC3HAV1L PE=1 SV=2	ZCCHL_HUMAN	ZC3HAV1L	33 kDa																								1			2				1									3	1	2	1				1
982	Annexin A6 OS=Homo sapiens OX=9606 GN=ANXA6 PE=1 SV=3	ANXA6_HUMAN	ANXA6	76 kDa																1							2														1		1	1			1		1		
983	Cullin-5 OS=Homo sapiens OX=9606 GN=CUL5 PE=1 SV=4	CUL5_HUMAN	CUL5	91 kDa	1																	0			0					0	2	2										3					1				
984	Coatomer subunit alpha OS=Homo sapiens OX=9606 GN=COPA PE=1 SV=2	COPA_HUMAN	COPA	138 kDa																					0						1											1	1				1				2
985	Transportin-3 OS=Homo sapiens OX=9606 GN=TNPO3 PE=1 SV=3	TNPO3_HUMAN	TNPO3	104 kDa	2						1																				2											2					2				
986	Serine/threonine-protein phosphatase 2A 65 kDa regulatory subunit A beta isoform OS=Homo sapiens OX=9606 GN=PPP2R1B PE=1 SV=3	2AAB_HUMAN	PPP2R1B	66 kDa	12																	6									11											15					12				
987	Translation initiation factor eIF-2B subunit epsilon OS=Homo sapiens OX=9606 GN=EIF2B5 PE=1 SV=3	EI2BE_HUMAN	EIF2B5	80 kDa	1						2																				3											0					4				
988	Glucosamine-6-phosphate isomerase 1 OS=Homo sapiens OX=9606 GN=GNPDA1 PE=1 SV=1	GNPI1_HUMAN	GNPDA1	33 kDa	1																										4											1					4				
989	Rap guanine nucleotide exchange factor 2 OS=Homo sapiens OX=9606 GN=RAPGEF2 PE=1 SV=1	RPGF2_HUMAN	RAPGEF2	167 kDa																											1											4	0		0		1				
990	Ran-specific GTPase-activating protein OS=Homo sapiens OX=9606 GN=RANBP1 PE=1 SV=1	RANG_HUMAN	RANBP1	23 kDa																					0					1												4	1				2				
991	Mannose-1-phosphate guanyltransferase beta OS=Homo sapiens OX=9606 GN=GMPPB PE=1 SV=2	GMPPB_HUMAN	GMPPB	40 kDa																											1											3					3				
992	Dipeptidyl peptidase 3 OS=Homo sapiens OX=9606 GN=DPP3 PE=1 SV=2	DPP3_HUMAN	DPP3	83 kDa																											3											0	1				3				
993	Tyrosine-protein kinase Yes OS=Homo sapiens OX=9606 GN=YES1 PE=1 SV=3	YES_HUMAN	YES1	61 kDa			1							1							1					0		0								1						1		1				0			1
994	Ubiquitin carboxyl-terminal hydrolase 19 OS=Homo sapiens OX=9606 GN=USP19 PE=1 SV=2	UBP19_HUMAN	USP19	146 kDa																											1			1						1	1	2					0				
995	FH1/FH2 domain-containing protein 1 OS=Homo sapiens OX=9606 GN=FHOD1 PE=1 SV=3	FHOD1_HUMAN	FHOD1	127 kDa																											3											3	2		1		1				
996	Voltage-dependent anion-selective channel protein 3 OS=Homo sapiens OX=9606 GN=VDAC3 PE=1 SV=1	VDAC3_HUMAN	VDAC3	31 kDa	2								1												1		4			0																					
997	3-ketoacyl-CoA thiolase, mitochondrial OS=Homo sapiens OX=9606 GN=ACAA2 PE=1 SV=2	THIM_HUMAN	ACAA2	42 kDa																											2								0										1		1
998	Alpha-2-macroglobulin OS=Homo sapiens OX=9606 GN=A2M PE=1 SV=3	A2MG_HUMAN	A2M	163 kDa																						2								1													2				
999	Golgi SNAP receptor complex member 2 OS=Homo sapiens OX=9606 GN=GOSR2 PE=1 SV=2	GOSR2_HUMAN	GOSR2	25 kDa																	1								1		0						1					0			2		1				1
1000	Desmoglein-1 OS=Homo sapiens OX=9606 GN=DSG1 PE=1 SV=2	DSG1_HUMAN	DSG1	114 kDa													1				1	1	0				0			1	1																		1	2	
1001	3-mercaptopyruvate sulfurtransferase OS=Homo sapiens OX=9606 GN=MPST PE=1 SV=3	THTM_HUMAN	MPST	33 kDa			1						1		0			2					1							0	1																				1
1002	Serine incorporator 3 OS=Homo sapiens OX=9606 GN=SERINC3 PE=2 SV=2	SERC3_HUMAN	SERINC3	53 kDa			1									1	1	1	1				0		1																										
1003	Apolipoprotein L3 OS=Homo sapiens OX=9606 GN=APOL3 PE=1 SV=3	APOL3_HUMAN	APOL3	44 kDa					1	1		0		1									1									1			0									0							
1004	Ras-related protein Rab-23 OS=Homo sapiens OX=9606 GN=RAB23 PE=1 SV=1	RAB23_HUMAN	RAB23	27 kDa	1	0	1																									1			1			0					0								
1005	HLA class I histocompatibility antigen, A-33 alpha chain OS=Homo sapiens OX=9606 GN=HLA-A PE=1 SV=3	1A33_HUMAN		41 kDa							1								0		0	1	1																												
1006	Mitogen-activated protein kinase 1 OS=Homo sapiens OX=9606 GN=MAPK1 PE=1 SV=3	MK01_HUMAN	MAPK1	41 kDa																																						0	2				0			0	2
1007	Transportin-1 OS=Homo sapiens OX=9606 GN=TNPO1 PE=1 SV=2	TNPO1_HUMAN	TNPO1	102 kDa	2																										0											1					0				
1008	Estradiol 17-beta-dehydrogenase 11 OS=Homo sapiens OX=9606 GN=HSD17B11 PE=1 SV=3	DHB11_HUMAN	HSD17B11	33 kDa		2			2							1							1			1		0																							
1009	Actin-related protein 2/3 complex subunit 2 OS=Homo sapiens OX=9606 GN=ARPC2 PE=1 SV=1	ARPC2_HUMAN	ARPC2	34 kDa								1															0			1													1						1	1	1
1010	Thioredoxin-dependent peroxide reductase, mitochondrial OS=Homo sapiens OX=9606 GN=PRDX3 PE=1 SV=3	PRDX3_HUMAN	PRDX3	28 kDa	2		1			1			1														0				1						0														
1011	Heterogeneous nuclear ribonucleoprotein A1 OS=Homo sapiens OX=9606 GN=HNRNPA1 PE=1 SV=5	ROA1_HUMAN	HNRNPA1	39 kDa																		1					1										1					2					0				
1012	Condensin complex subunit 1 OS=Homo sapiens OX=9606 GN=NCAPD2 PE=1 SV=3	CND1_HUMAN	NCAPD2	157 kDa																																						1			0		1		2		
1013	Protein phosphatase 1 regulatory subunit 7 OS=Homo sapiens OX=9606 GN=PPP1R7 PE=1 SV=1	PP1R7_HUMAN	PPP1R7	42 kDa																											3											3					1				
1014	HLA class I histocompatibility antigen, B-35 alpha chain OS=Homo sapiens OX=9606 GN=HLA-B PE=1 SV=1	1B35_HUMAN (+2)	HLA-B	40 kDa						3		1										3																													
1015	Ubiquitin thioesterase OTU1 OS=Homo sapiens OX=9606 GN=YOD1 PE=1 SV=1	OTU1_HUMAN	YOD1	38 kDa									0				0	3												1																					
1016	SEC14-like protein 4 OS=Homo sapiens OX=9606 GN=SEC14L4 PE=1 SV=1	S14L4_HUMAN	SEC14L4	47 kDa																								0			0											1					2				
1017	Platelet glycoprotein 4 OS=Homo sapiens OX=9606 GN=CD36 PE=1 SV=2	CD36_HUMAN	CD36	53 kDa			1			1				1																			1		2	1		0													
1018	Filaggrin-2 OS=Homo sapiens OX=9606 GN=FLG2 PE=1 SV=1	FILA2_HUMAN	FLG2	248 kDa													1			1								1							0											1			0	1	
1019	Coatomer subunit zeta-1 OS=Homo sapiens OX=9606 GN=COPZ1 PE=1 SV=1	COPZ1_HUMAN	COPZ1	20 kDa																											1					0						1	1				1				2
1020	Tetratricopeptide repeat protein 37 OS=Homo sapiens OX=9606 GN=TTC37 PE=1 SV=1	TTC37_HUMAN	TTC37	175 kDa																											1										0	2	1				1				1
1021	Double-strand break repair protein MRE11 OS=Homo sapiens OX=9606 GN=MRE11 PE=1 SV=3	MRE11_HUMAN	MRE11	81 kDa																											1						1					1	1				3				
1022	Dermcidin OS=Homo sapiens OX=9606 GN=DCD PE=1 SV=2	DCD_HUMAN	DCD	11 kDa		0							0		3		1	1																																	
1023	40S ribosomal protein S9 OS=Homo sapiens OX=9606 GN=RPS9 PE=1 SV=3	RS9_HUMAN	RPS9	23 kDa																						0																1	1		0						2
1024	Ubiquitin-like modifier-activating enzyme 5 OS=Homo sapiens OX=9606 GN=UBA5 PE=1 SV=1	UBA5_HUMAN	UBA5	45 kDa	1																									0	3	0										2									
1025	Thioredoxin reductase 1, cytoplasmic OS=Homo sapiens OX=9606 GN=TXNRD1 PE=1 SV=3	TRXR1_HUMAN	TXNRD1	71 kDa																					1						3											1					0				
1026	Nucleophosmin OS=Homo sapiens OX=9606 GN=NPM1 PE=1 SV=2	NPM_HUMAN	NPM1	33 kDa																											2				1								4								
1027	60S ribosomal protein L5 OS=Homo sapiens OX=9606 GN=RPL5 PE=1 SV=3	RL5_HUMAN	RPL5	34 kDa																																							2		3				1		
1028	Charged multivesicular body protein 2a OS=Homo sapiens OX=9606 GN=CHMP2A PE=1 SV=1	CHM2A_HUMAN	CHMP2A	25 kDa					0													0										0			0				0											1	
1029	Talin-2 OS=Homo sapiens OX=9606 GN=TLN2 PE=1 SV=4	TLN2_HUMAN	TLN2	272 kDa																																	0					4									2
1030	Filamin-A OS=Homo sapiens OX=9606 GN=FLNA PE=1 SV=4	FLNA_HUMAN	FLNA	281 kDa								0																			1																2				
1031	Type 1 phosphatidylinositol 4,5-bisphosphate 4-phosphatase OS=Homo sapiens OX=9606 GN=PIP4P1 PE=1 SV=1	PP4P1_HUMAN	PIP4P1	29 kDa												1	1	0			1		1																							2					
1032	60S ribosomal protein L36 OS=Homo sapiens OX=9606 GN=RPL36 PE=1 SV=3	RL36_HUMAN	RPL36	12 kDa																											1											1	1		1				1		
1033	Importin subunit alpha-5 OS=Homo sapiens OX=9606 GN=KPNA1 PE=1 SV=3	IMA5_HUMAN	KPNA1	60 kDa																		6					5				6											4					4				
1034	Calcyclin-binding protein OS=Homo sapiens OX=9606 GN=CACYBP PE=1 SV=2	CYBP_HUMAN	CACYBP	26 kDa																															1	0						2	2								1
1035	BRISC complex subunit Abraxas 2 OS=Homo sapiens OX=9606 GN=ABRAXAS2 PE=1 SV=2	ABRX2_HUMAN	ABRAXAS2	47 kDa																		1								1	0											1									
1036	Transaldolase OS=Homo sapiens OX=9606 GN=TALDO1 PE=1 SV=2	TALDO_HUMAN	TALDO1	38 kDa	1																										2	1											1								
1037	Plastin-2 OS=Homo sapiens OX=9606 GN=LCP1 PE=1 SV=6	PLSL_HUMAN	LCP1	70 kDa								1																				1		1									1								
1038	60S ribosomal protein L21 OS=Homo sapiens OX=9606 GN=RPL21 PE=1 SV=2	RL21_HUMAN	RPL21	19 kDa			1				1												0																												1
1039	Ras GTPase-activating-like protein IQGAP2 OS=Homo sapiens OX=9606 GN=IQGAP2 PE=1 SV=4	IQGA2_HUMAN	IQGAP2	181 kDa																	0				0																	2					1				
1040	40S ribosomal protein S16 OS=Homo sapiens OX=9606 GN=RPS16 PE=1 SV=2	RS16_HUMAN	RPS16	16 kDa																						1					0												1								2
1041	Prefoldin subunit 5 OS=Homo sapiens OX=9606 GN=PFDN5 PE=1 SV=2	PFD5_HUMAN	PFDN5	17 kDa																											1											2					1				
1042	Glutamate--cysteine ligase catalytic subunit OS=Homo sapiens OX=9606 GN=GCLC PE=1 SV=2	GSH1_HUMAN	GCLC	73 kDa																											1	1										1									
1043	Coatomer subunit beta' OS=Homo sapiens OX=9606 GN=COPB2 PE=1 SV=2	COPB2_HUMAN	COPB2	102 kDa																											1											0					1				1
1044	Protein TFG OS=Homo sapiens OX=9606 GN=TFG PE=1 SV=2	TFG_HUMAN	TFG	43 kDa	0																				1																	2					0				
1045	Importin-11 OS=Homo sapiens OX=9606 GN=IPO11 PE=1 SV=1	IPO11_HUMAN	IPO11	113 kDa																											0											1					1				
1046	Keratin, type I cuticular Ha6 OS=Homo sapiens OX=9606 GN=KRT36 PE=2 SV=1	KRT36_HUMAN	KRT36	52 kDa										1																5																					
1047	RUN and FYVE domain-containing protein 1 OS=Homo sapiens OX=9606 GN=RUFY1 PE=1 SV=2	RUFY1_HUMAN	RUFY1	80 kDa					0																																	2									0
1048	60S ribosomal protein L7a OS=Homo sapiens OX=9606 GN=RPL7A PE=1 SV=2	RL7A_HUMAN	RPL7A	30 kDa								1																															3		1		1				
1049	Tyrosine-protein kinase Lyn OS=Homo sapiens OX=9606 GN=LYN PE=1 SV=3	LYN_HUMAN	LYN	59 kDa	0	1		1								1																											2								
1050	Probable ATP-dependent RNA helicase DDX17 OS=Homo sapiens OX=9606 GN=DDX17 PE=1 SV=2	DDX17_HUMAN	DDX17	80 kDa	0																										1											1					2				
1051	C4b-binding protein alpha chain OS=Homo sapiens OX=9606 GN=C4BPA PE=1 SV=2	C4BPA_HUMAN	C4BPA	67 kDa												1		1																															2		
1052	Cofilin-1 OS=Homo sapiens OX=9606 GN=CFL1 PE=1 SV=3	COF1_HUMAN	CFL1	19 kDa																																						1			1						3
1053	40S ribosomal protein S3 OS=Homo sapiens OX=9606 GN=RPS3 PE=1 SV=2	RS3_HUMAN	RPS3	27 kDa																									1													0	1								1
1054	Keratin, type II cytoskeletal 6B OS=Homo sapiens OX=9606 GN=KRT6B PE=1 SV=5	K2C6B_HUMAN	KRT6B	60 kDa													74			36								34		41																					
1055	Tubulin gamma-2 chain OS=Homo sapiens OX=9606 GN=TUBG2 PE=2 SV=1	TBG2_HUMAN	TUBG2	51 kDa																											0									1		1					0				
1056	ATP synthase subunit alpha, mitochondrial OS=Homo sapiens OX=9606 GN=ATP5F1A PE=1 SV=1	ATPA_HUMAN	ATP5F1A	60 kDa	2																						1																								
1057	Serine/threonine-protein phosphatase PP1-alpha catalytic subunit OS=Homo sapiens OX=9606 GN=PPP1CA PE=1 SV=1	PP1A_HUMAN		38 kDa	1																																					1									
1058	STE20-like serine/threonine-protein kinase OS=Homo sapiens OX=9606 GN=SLK PE=1 SV=1	SLK_HUMAN	SLK	143 kDa																											0												0				2				
1059	Vesicle transport through interaction with t-SNAREs homolog 1A OS=Homo sapiens OX=9606 GN=VTI1A PE=1 SV=2	VTI1A_HUMAN	VTI1A	25 kDa										1								1									1																	1			
1060	Quinone oxidoreductase PIG3 OS=Homo sapiens OX=9606 GN=TP53I3 PE=1 SV=2	QORX_HUMAN	TP53I3	36 kDa	2	1																																									0				1
1061	Phosphorylase b kinase gamma catalytic chain, liver/testis isoform OS=Homo sapiens OX=9606 GN=PHKG2 PE=1 SV=1	PHKG2_HUMAN	PHKG2	46 kDa																											1											2					2				
1062	Condensin complex subunit 2 OS=Homo sapiens OX=9606 GN=NCAPH PE=1 SV=3	CND2_HUMAN	NCAPH	83 kDa																											1						1					2									
1063	Phospholipase A-2-activating protein OS=Homo sapiens OX=9606 GN=PLAA PE=1 SV=2	PLAP_HUMAN	PLAA	87 kDa																		1		0		1																					1				
1064	Prohibitin-2 OS=Homo sapiens OX=9606 GN=PHB2 PE=1 SV=2	PHB2_HUMAN	PHB2	33 kDa	1																	0					1			0																					
1065	Plasminogen OS=Homo sapiens OX=9606 GN=PLG PE=1 SV=2	PLMN_HUMAN	PLG	91 kDa											0																0	2														1					
1066	AP-1 complex subunit mu-1 OS=Homo sapiens OX=9606 GN=AP1M1 PE=1 SV=3	AP1M1_HUMAN	AP1M1	49 kDa																											2											1	0								
1067	Junction plakoglobin OS=Homo sapiens OX=9606 GN=JUP PE=1 SV=3	PLAK_HUMAN	JUP	82 kDa						2							0											1																							
1068	L-fucose kinase OS=Homo sapiens OX=9606 GN=FCSK PE=1 SV=2	FCSK_HUMAN	FCSK	118 kDa																											2											3									
1069	Keratin, type II cytoskeletal 4 OS=Homo sapiens OX=9606 GN=KRT4 PE=1 SV=4	K2C4_HUMAN	KRT4	57 kDa																				5																										4	
1070	Pyruvate kinase PKM OS=Homo sapiens OX=9606 GN=PKM PE=1 SV=4	KPYM_HUMAN	PKM	58 kDa																											3																				
1071	Phosphorylase b kinase regulatory subunit beta OS=Homo sapiens OX=9606 GN=PHKB PE=1 SV=3	KPBB_HUMAN	PHKB	125 kDa																																											3				
1072	Aspartate aminotransferase, mitochondrial OS=Homo sapiens OX=9606 GN=GOT2 PE=1 SV=3	AATM_HUMAN	GOT2	48 kDa																													1	2																	1
1073	Synaptosomal-associated protein 29 OS=Homo sapiens OX=9606 GN=SNAP29 PE=1 SV=1	SNP29_HUMAN	SNAP29	29 kDa																	1																					1				2					
1074	Programmed cell death protein 5 OS=Homo sapiens OX=9606 GN=PDCD5 PE=1 SV=3	PDCD5_HUMAN	PDCD5	14 kDa																						1					2											1									
1075	40S ribosomal protein S6 OS=Homo sapiens OX=9606 GN=RPS6 PE=1 SV=1	RS6_HUMAN	RPS6	29 kDa																																							2		1				1		
1076	Protein canopy homolog 3 OS=Homo sapiens OX=9606 GN=CNPY3 PE=1 SV=1	CNPY3_HUMAN		31 kDa													2	1						0																											
1077	Prefoldin subunit 6 OS=Homo sapiens OX=9606 GN=PFDN6 PE=1 SV=1	PFD6_HUMAN	PFDN6	15 kDa																											0											1					1				
1078	Ubiquitin-like protein ISG15 OS=Homo sapiens OX=9606 GN=ISG15 PE=1 SV=5	ISG15_HUMAN		18 kDa																	2	1																													
1079	ATP-dependent RNA helicase DHX29 OS=Homo sapiens OX=9606 GN=DHX29 PE=1 SV=2	DHX29_HUMAN	DHX29	155 kDa																																							1								1
1080	UDP-N-acetylglucosamine--peptide N-acetylglucosaminyltransferase 110 kDa subunit OS=Homo sapiens OX=9606 GN=OGT PE=1 SV=3	OGT1_HUMAN	OGT	117 kDa																																						1									1
1081	Prefoldin subunit 1 OS=Homo sapiens OX=9606 GN=PFDN1 PE=1 SV=2	PFD1_HUMAN		14 kDa																											1											2									
1082	Enhancer of mRNA-decapping protein 4 OS=Homo sapiens OX=9606 GN=EDC4 PE=1 SV=1	EDC4_HUMAN		152 kDa																																				1		1									
1083	Microtubule-associated protein 1S OS=Homo sapiens OX=9606 GN=MAP1S PE=1 SV=2	MAP1S_HUMAN	MAP1S	112 kDa																																	0					0					1				
1084	E3 ubiquitin-protein ligase ARIH2 OS=Homo sapiens OX=9606 GN=ARIH2 PE=1 SV=1	ARI2_HUMAN	ARIH2	58 kDa	1																										0																0				
1085	Host cell factor 1 OS=Homo sapiens OX=9606 GN=HCFC1 PE=1 SV=2	HCFC1_HUMAN	HCFC1	209 kDa		0																															0						1								
1086	6-phosphogluconate dehydrogenase, decarboxylating OS=Homo sapiens OX=9606 GN=PGD PE=1 SV=3	6PGD_HUMAN	PGD	53 kDa																					0																		2								
1087	Intersectin-1 OS=Homo sapiens OX=9606 GN=ITSN1 PE=1 SV=3	ITSN1_HUMAN	ITSN1	195 kDa																																						1									
1088	FK506-binding protein 15 OS=Homo sapiens OX=9606 GN=FKBP15 PE=1 SV=2	FKB15_HUMAN	FKBP15	134 kDa																																				1								1			
1089	Phosducin-like protein 3 OS=Homo sapiens OX=9606 GN=PDCL3 PE=1 SV=1	PDCL3_HUMAN		28 kDa																											1											1									
1090	60S ribosomal protein L23a OS=Homo sapiens OX=9606 GN=RPL23A PE=1 SV=1	RL23A_HUMAN		18 kDa														2							1																										

1091	Pterin-4-alpha-carbinolamine dehydratase 2 OS=Homo sapiens OX=9606 GN=PCBD2 PE=1 SV=4	PHS2_HUMAN		14 kDa		1		1			
1092	Arf-GAP with coiled-coil, ANK repeat and PH domain-containing protein 1 OS=Homo sapiens OX=9606 GN=ACAP1 PE=1 SV=1	ACAP1_HUMAN	ACAP1	82 kDa	0						1
1093	Nuclear cap-binding protein subunit 1 OS=Homo sapiens OX=9606 GN=NCBP1 PE=1 SV=1	NCBP1_HUMAN		92 kDa						2	
1094	Hsp90 co-chaperone Cdc37 OS=Homo sapiens OX=9606 GN=CDC37 PE=1 SV=1	CDC37_HUMAN	CDC37	44 kDa						2	
1095	Methionine aminopeptidase 2 OS=Homo sapiens OX=9606 GN=METAP2 PE=1 SV=1	MAP2_HUMAN	METAP2	53 kDa						2	
1096	TBC1 domain family member 17 OS=Homo sapiens OX=9606 GN=TBC1D17 PE=1 SV=2	TBC17_HUMAN		73 kDa						1	
1097	Prefoldin subunit 3 OS=Homo sapiens OX=9606 GN=VBP1 PE=1 SV=4	PFD3_HUMAN		23 kDa						1	
1098	Properdin OS=Homo sapiens OX=9606 GN=CFP PE=1 SV=2	PROP_HUMAN		51 kDa			1				
