# Supplementary material for: Proteome of Stored RBC Membrane and Vesicles from Heterozygous Beta Thalassemia Donors
Source: Int J Mol Sci. 2021 Mar 25;22(7):3369. doi: 10.3390/ijms22073369 (PMC8037027; doi:10.3390/ijms22073369)
Supplement: Supplementary file 1 [file ijms-22-03369-s001.zip › Table S2 - Proteomics analysis of pooled RBC membranes.pdf]

#	Identified Proteins (833)	Accession Number	Alternate ID	Molecular Weight	Total Spectrum Count											
					beta-Thalassemia Heterozygotes						Control					
					Day 7	Day 14	Day 21	Day 28	Day 35	Day 42	Day 7	Day 14	Day 21	Day 28	Day 35	Day 42
1	Spectrin alpha chain, erythrocytic 1 OS=Homo sapiens OX=9606 GN=SPTA1 PE=1 SV=5	SPTA1_HUMAN	SPTA1	280 kDa	3785	3377	3620	3731	3625	3245	3102	3293	3387	3424	3157	3446
2	Spectrin beta chain, erythrocytic OS=Homo sapiens OX=9606 GN=SPTB PE=1 SV=5	SPTB1_HUMAN	SPTB	246 kDa	3600	3165	3312	3457	3307	3147	2780	2932	3104	3138	2957	3240
3	Ankyrin-1 OS=Homo sapiens OX=9606 GN=ANK1 PE=1 SV=3	ANK1_HUMAN	ANK1	206 kDa	2719	2527	2587	2682	2613	2416	2270	2351	2465	2481	2348	2544
4	Band 3 anion transport protein OS=Homo sapiens OX=9606 GN=SLC4A1 PE=1 SV=3	B3AT_HUMAN	SLC4A1	102 kDa	1167	1047	1112	1158	1119	1072	1013	1016	1075	1071	1034	1076
5	Glyceraldehyde-3-phosphate dehydrogenase OS=Homo sapiens OX=9606 GN=GAPDH PE=1 SV=3	G3P_HUMAN	GAPDH	36 kDa	1139	1104	1120	1169	1124	1048	945	996	992	1022	973	1050
6	Protein 4.1 OS=Homo sapiens OX=9606 GN=EPB41 PE=1 SV=4	41_HUMAN	EPB41	97 kDa	971	904	967	953	955	912	845	897	961	938	859	920
7	Erythrocyte membrane protein band 4.2 OS=Homo sapiens OX=9606 GN=EPB42 PE=1 SV=3	EPB42_HUMAN	EPB42	77 kDa	899	751	779	851	774	822	612	683	726	752	666	786
8	Actin, cytoplasmic 1 OS=Homo sapiens OX=9606 GN=ACTB PE=1 SV=1	ACTB_HUMAN	ACTB	42 kDa	638	657	642	670	656	611	615	618	646	654	666	650
9	Erythrocyte band 7 integral membrane protein OS=Homo sapiens OX=9606 GN=STOM PE=1 SV=3	STOM_HUMAN	STOM	32 kDa	411	386	427	418	395	389	408	414	400	418	403	400
10	Beta-adducin OS=Homo sapiens OX=9606 GN=ADD2 PE=1 SV=3	ADDB_HUMAN	ADD2	81 kDa	413	407	414	402	425	348	368	395	392	377	383	378
11	Blood group Rh(CE) polypeptide OS=Homo sapiens OX=9606 GN=RHCE PE=1 SV=2	RHCE_HUMAN	RHCE	46 kDa	323	327	323	350	324	308	294	308	318	318	318	318
12	Fructose-bisphosphate aldolase A OS=Homo sapiens OX=9606 GN=ALDOA PE=1 SV=2	ALDOA_HUMAN	ALDOA	39 kDa	292	275	330	345	315	337	223	242	275	277	250	283
13	Flotillin-2 OS=Homo sapiens OX=9606 GN=FLOT2 PE=1 SV=2	FLOT2_HUMAN	FLOT2	47 kDa	336	311	319	322	335	300	278	291	310	300	282	323
14	Flotillin-1 OS=Homo sapiens OX=9606 GN=FLOT1 PE=1 SV=3	FLOT1_HUMAN	FLOT1	47 kDa	264	251	243	249	262	243	236	237	249	241	230	256
15	Alpha-adducin OS=Homo sapiens OX=9606 GN=ADD1 PE=1 SV=2	ADDA_HUMAN	ADD1	81 kDa	255	224	252	252	239	201	214	231	217	231	214	212
16	Hemoglobin subunit alpha OS=Homo sapiens OX=9606 GN=HBA1 PE=1 SV=2	HBA_HUMAN	HBA1	15 kDa	223	234	198	158	177	201	266	273	273	239	171	219
17	Clathrin heavy chain 1 OS=Homo sapiens OX=9606 GN=CLTC PE=1 SV=5	CLH1_HUMAN	CLTC	192 kDa	185	230	242	220	255	251	161	218	203	224	226	237
18	Myosin-9 OS=Homo sapiens OX=9606 GN=MYH9 PE=1 SV=4	MYH9_HUMAN	MYH9	227 kDa	199	267	239	277	297	230	114	139	135	142	136	144
19	55 kDa erythrocyte membrane protein OS=Homo sapiens OX=9606 GN=MPP1 PE=1 SV=2	EM55_HUMAN	MPP1	52 kDa	220	182	205	209	197	192	187	189	201	203	193	198
20	Peroxiredoxin-2 OS=Homo sapiens OX=9606 GN=PRDX2 PE=1 SV=5	PRDX2_HUMAN	PRDX2	22 kDa	214	219	196	184	264	216	197	220	178	169	179	193
21	Protein argonaute-2 OS=Homo sapiens OX=9606 GN=AGO2 PE=1 SV=3	AGO2_HUMAN	AGO2	97 kDa	209	186	195	182	200	174	178	177	173	184	162	180
22	E3 ubiquitin-protein ligase UBR4 OS=Homo sapiens OX=9606 GN=UBR4 PE=1 SV=1	UBR4_HUMAN	UBR4	574 kDa	101	159	147	147	172	177	135	207	140	180	197	191
23	Dematin OS=Homo sapiens OX=9606 GN=DMTN PE=1 SV=3	DEMA_HUMAN	DMTN	46 kDa	156	139	166	159	158	136	168	164	154	161	172	158
24	ATP-citrate synthase OS=Homo sapiens OX=9606 GN=ACLY PE=1 SV=3	ACLY_HUMAN	ACLY	121 kDa	96	124	166	166	160	159	91	117	117	130	133	150
25	Solute carrier family 2, facilitated glucose transporter member 1 OS=Homo sapiens OX=9606 GN=SLC2A1 PE=1 SV=2	GTR1_HUMAN	SLC2A1	54 kDa	160	126	153	160	152	151	135	153	138	154	125	158
26	Transitional endoplasmic reticulum ATPase OS=Homo sapiens OX=9606 GN=VCP PE=1 SV=4	TERA_HUMAN	VCP	89 kDa	103	138	117	122	124	111	141	159	127	119	145	114
27	Plasma membrane calcium-transporting ATPase 4 OS=Homo sapiens OX=9606 GN=ATP2B4 PE=1 SV=2	AT2B4_HUMAN	ATP2B4	138 kDa	115	114	110	116	117	114	123	131	123	130	125	121
28	Glycophorin-C OS=Homo sapiens OX=9606 GN=GYPC PE=1 SV=1	GLPC_HUMAN	GYPC	14 kDa	184	146	152	148	149	131	117	133	140	149	122	142
29	Kell blood group glycoprotein OS=Homo sapiens OX=9606 GN=KEL PE=1 SV=2	KELL_HUMAN	KEL	83 kDa	107	102	92	94	92	109	117	111	102	115	115	121
30	Tropomodulin-1 OS=Homo sapiens OX=9606 GN=TMOD1 PE=1 SV=1	TMOD1_HUMAN	TMOD1	41 kDa	116	110	125	126	118	117	101	102	123	119	115	117
31	Hemoglobin subunit beta OS=Homo sapiens OX=9606 GN=HBB PE=1 SV=2	HBB_HUMAN	HBB	16 kDa	97	100	105	89	92	103	123	116	139	107	98	98
32	AP-2 complex subunit beta OS=Homo sapiens OX=9606 GN=AP2B1 PE=1 SV=1	AP2B1_HUMAN	AP2B1	105 kDa	90	109	111	103	105	108	71	84	86	89	87	79
33	Immunoglobulin gamma-1 heavy chain OS=Homo sapiens OX=9606 PE=1 SV=2	IGG1_HUMAN		49 kDa	93	95	72	97	95	86	90	89	88	88	97	87
34	Heat shock cognate 71 kDa protein OS=Homo sapiens OX=9606 GN=HSPA8 PE=1 SV=1	HSP7C_HUMAN	HSPA8	71 kDa	67	96	102	89	120	99	71	88	83	83	91	84
35	AP-2 complex subunit alpha-1 OS=Homo sapiens OX=9606 GN=AP2A1 PE=1 SV=3	AP2A1_HUMAN	AP2A1	108 kDa	76	95	93	90	87	86	63	64	69	74	73	80
36	Catalase OS=Homo sapiens OX=9606 GN=CAT PE=1 SV=3	CATA_HUMAN	CAT	60 kDa	76	77	67	66	94	77	94	84	74	58	78	66
37	ATP-binding cassette sub-family B member 6, mitochondrial OS=Homo sapiens OX=9606 GN=ABCB6 PE=1 SV=1	ABCB6_HUMAN	ABCB6	94 kDa	80	86	73	88	78	73	93	95	86	92	90	95
38	Myosin-10 OS=Homo sapiens OX=9606 GN=MYH10 PE=1 SV=3	MYH10_HUMAN	MYH10	229 kDa	139	178	166	195	198	163	34	36	39	50	44	47
39	Erythroid membrane-associated protein OS=Homo sapiens OX=9606 GN=ERMAP PE=1 SV=1	ERMAP_HUMAN	ERMAP	53 kDa	81	68	80	90	88	78	59	64	71	59	65	73
40	26S proteasome non-ATPase regulatory subunit 2 OS=Homo sapiens OX=9606 GN=PSMD2 PE=1 SV=3	PSMD2_HUMAN	PSMD2	100 kDa	85	92	88	93	97	91	73	82	59	71	80	71
41	Heat shock protein HSP 90-alpha OS=Homo sapiens OX=9606 GN=HSP90AA1 PE=1 SV=5	HS90A_HUMAN	HSP90AA1	85 kDa	76	97	87	72	115	97	54	64	52	50	61	45
42	Piezo-type mechanosensitive ion channel component 1 OS=Homo sapiens OX=9606 GN=PIEZO1 PE=1 SV=4	PIEZ1_HUMAN	PIEZO1	287 kDa	60	58	70	66	61	68	81	78	75	72	75	81
43	Ras-related protein Rap-1A OS=Homo sapiens OX=9606 GN=RAP1A PE=1 SV=1	RAP1A_HUMAN	RAP1A	21 kDa	118	78	79	86	68	58	62	59	65	54	56	51
44	Aquaporin-1 OS=Homo sapiens OX=9606 GN=AQP1 PE=1 SV=3	AQP1_HUMAN	AQP1	29 kDa	87	67	67	80	88	71	77	79	72	93	75	94
45	Multidrug resistance-associated protein 4 OS=Homo sapiens OX=9606 GN=ABCC4 PE=1 SV=3	MRP4_HUMAN	ABCC4	150 kDa	60	61	66	66	57	60	74	73	67	78	66	73
46	T-complex protein 1 subunit eta OS=Homo sapiens OX=9606 GN=CCT7 PE=1 SV=2	TCPH_HUMAN	CCT7	59 kDa	60	74	71	81	87	81	52	67	54	56	53	60
47	Arginase-1 OS=Homo sapiens OX=9606 GN=ARG1 PE=1 SV=2	ARGI1_HUMAN	ARG1	35 kDa	79	81	70	80	67	70	50	53	59	55	50	51
48	Acylamino-acid-releasing enzyme OS=Homo sapiens OX=9606 GN=APEH PE=1 SV=4	ACPH_HUMAN	APEH	81 kDa	49	53	63	50	80	53	73	77	54	46	63	51
49	T-complex protein 1 subunit zeta OS=Homo sapiens OX=9606 GN=CCT6A PE=1 SV=3	TCPZ_HUMAN	CCT6A	58 kDa	52	53	66	71	79	70	41	53	41	49	51	62
50	Cullin-associated NEDD8-dissociated protein 1 OS=Homo sapiens OX=9606 GN=CAND1 PE=1 SV=2	CAND1_HUMAN	CAND1	136 kDa	48	54	62	48	70	62	46	54	50	42	57	62
51	T-complex protein 1 subunit alpha OS=Homo sapiens OX=9606 GN=TCP1 PE=1 SV=1	TCPA_HUMAN	TCP1	60 kDa	58	64	57	65	81	84	39	54	42	41	50	58
52	Serum albumin OS=Homo sapiens OX=9606 GN=ALB PE=1 SV=2	ALBU_HUMAN	ALB	69 kDa	55	59	67	51	61	52	71	58	48	52	55	43
53	Keratin, type I cytoskeletal 10 OS=Homo sapiens OX=9606 GN=KRT10 PE=1 SV=6	K1C10_HUMAN	KRT10	59 kDa	44	80	64	55	45	54	76	76	59	58	49	46
54	E2 ubiquitin-conjugating enzyme OS=Homo sapiens OX=9606 GN=UBE2O PE=1 SV=3	sp|Q9C0C9|UBE2O_HUMAN	UBE2O	141 kDa	46	65	52	69	75	67	43	59	50	53	61	60
55	Delta-aminolevulinic acid dehydratase OS=Homo sapiens OX=9606 GN=ALAD PE=1 SV=1	HEM2_HUMAN	ALAD	36 kDa	48	55	54	48	64	57	53	58	43	46	61	52
56	Long-chain-fatty-acid--CoA ligase 4 OS=Homo sapiens OX=9606 GN=ACSL4 PE=1 SV=2	ACSL4_HUMAN	ACSL4	79 kDa	50	53	53	60	61	54	63	52	57	50	57	54
57	Basal cell adhesion molecule OS=Homo sapiens OX=9606 GN=BCAM PE=1 SV=2	BCAM_HUMAN	BCAM	67 kDa	62	61	62	54	69	55	58	53	55	62	59	63
58	Endonuclease domain-containing 1 protein OS=Homo sapiens OX=9606 GN=ENDOD1 PE=1 SV=2	ENDD1_HUMAN	ENDOD1	55 kDa	48	50	58	50	56	54	54	51	49	46	47	53
59	Proteasome subunit beta type-4 OS=Homo sapiens OX=9606 GN=PSMB4 PE=1 SV=4	PSB4_HUMAN	PSMB4	29 kDa	66	71	71	75	79	62	59	62	52	63	66	59
60	Guanine nucleotide-binding protein G(I)/G(S)/G(T) subunit beta-1 OS=Homo sapiens OX=9606 GN=GNB1 PE=1 SV=3	GBB1_HUMAN	GNB1	37 kDa	62	54	50	54	51	39	49	56	58	51	61	71
61	T-complex protein 1 subunit epsilon OS=Homo sapiens OX=9606 GN=CCT5 PE=1 SV=1	TCPE_HUMAN	CCT5	60 kDa	55	65	51	50	65	61	38	57	36	45	49	42
62	26S proteasome non-ATPase regulatory subunit 1 OS=Homo sapiens OX=9606 GN=PSMD1 PE=1 SV=2	PSMD1_HUMAN	PSMD1	106 kDa	50	51	39	51	49	67	38	46	39	36	43	45
63	E3 ubiquitin-protein ligase HUWE1 OS=Homo sapiens OX=9606 GN=HUWE1 PE=1 SV=3	HUWE1_HUMAN	HUWE1	482 kDa	32	54	41	49	51	54	33	60	35	48	64	62
64	Guanine nucleotide-binding protein G(i) subunit alpha-2 OS=Homo sapiens OX=9606 GN=GNAI2 PE=1 SV=3	GNAI2_HUMAN	GNAI2	40 kDa	49	56	58	59	56	57	64	61	58	54	51	58
65	2',3'-cyclic-nucleotide 3'-phosphodiesterase OS=Homo sapiens OX=9606 GN=CNP PE=1 SV=2	CN37_HUMAN	CNP	48 kDa	51	48	47	44	45	40	58	51	57	56	51	45
66	Aldehyde dehydrogenase family 16 member A1 OS=Homo sapiens OX=9606 GN=ALDH16A1 PE=1 SV=2	A16A1_HUMAN	ALDH16A1	85 kDa	49	61	61	59	69	56	39	41	40	43	47	52
67	Heat shock 70 kDa protein 1A OS=Homo sapiens OX=9606 GN=HSPA1A PE=1 SV=1	HS71A_HUMAN (+1)	HSPA1A	70 kDa	46	71	78	72	86	79	30	33	42	43	53	48
68	Ammonium transporter Rh type A OS=Homo sapiens OX=9606 GN=RHAG PE=1 SV=2	RHAG_HUMAN	RHAG	44 kDa	47	54	54	55	53	59	49	55	57	55	52	52
69	Sorbitol dehydrogenase OS=Homo sapiens OX=9606 GN=SORD PE=1 SV=4	DHSO_HUMAN	SORD	38 kDa	62	47	46	51	51	44	59	54	48	48	56	39
70	Ras-related protein Rap-2b OS=Homo sapiens OX=9606 GN=RAP2B PE=1 SV=1	RAP2B_HUMAN	RAP2B	21 kDa	56	47	50	47	51	44	54	60	52	55	49	53
71	Golgi-associated plant pathogenesis-related protein 1 OS=Homo sapiens OX=9606 GN=GLIPR2 PE=1 SV=3	GAPR1_HUMAN	GLIPR2	17 kDa	50	52	48	46	50	44	58	55	53	53	58	57
72	26S proteasome regulatory subunit 7 OS=Homo sapiens OX=9606 GN=PSMC2 PE=1 SV=3	PRS7_HUMAN	PSMC2	49 kDa	43	60	41	52	56	49	40	47	38	41	39	44
73	Glutathione S-transferase LANCL1 OS=Homo sapiens OX=9606 GN=LANCL1 PE=1 SV=1	LANC1_HUMAN	LANCL1	45 kDa	36	49	52	61	58	56	39	40	46	54	51	50
74	Probable ubiquitin carboxyl-terminal hydrolase FAF-X OS=Homo sapiens OX=9606 GN=USP9X PE=1 SV=3	USP9X_HUMAN	USP9X	292 kDa	38	41	51	43	56	54	25	43	21	27	40	35
75	Phosphatidylinositol 5-phosphate 4-kinase type-2 alpha OS=Homo sapiens OX=9606 GN=PIP4K2A PE=1 SV=2	PI42A_HUMAN	PIP4K2A	46 kDa	46	38	45	46	48	42	44	55	53	58	56	48
76	Proteasome subunit alpha type-5 OS=Homo sapiens OX=9606 GN=PSMA5 PE=1 SV=3	PSA5_HUMAN	PSMA5	26 kDa	33	60	56	58	87	36	41	45	30	36	43	32
77	NADH-cytochrome b5 reductase 3 OS=Homo sapiens OX=9606 GN=CYB5R3 PE=1 SV=3	NB5R3_HUMAN	CYB5R3	34 kDa	29	28	44	31	34	27	50	43	48	53	55	60
78	GTPase-activating protein and VPS9 domain-containing protein 1 OS=Homo sapiens OX=9606 GN=GAPVD1 PE=1 SV=2	GAPD1_HUMAN	GAPVD1	165 kDa	27	43	40	45	50	43	23	34	23	32	40	35
79	T-complex protein 1 subunit gamma OS=Homo sapiens OX=9606 GN=CCT3 PE=1 SV=4	TCPG_HUMAN	CCT3	61 kDa	38	51	42	49	49	46	45	50	44	43	44	35
80	T-complex protein 1 subunit delta OS=Homo sapiens OX=9606 GN=CCT4 PE=1 SV=4	TCPD_HUMAN	CCT4	58 kDa	42	50	47	50	62	52	31	44	47	44	52	49
81	Protein disulfide-isomerase OS=Homo sapiens OX=9606 GN=P4HB PE=1 SV=3	PDIA1_HUMAN	P4HB	57 kDa	39	41	39	38	43	41	54	50	48	48	48	48
82	Keratin, type II cytoskeletal 1 OS=Homo sapiens OX=9606 GN=KRT1 PE=1 SV=6	K2C1_HUMAN	KRT1	66 kDa	45	64	52	42	39	48	57	80	51	51	42	40
83	C-1-tetrahydrofolate synthase, cytoplasmic OS=Homo sapiens OX=9606 GN=MTHFD1 PE=1 SV=3	C1TC_HUMAN	MTHFD1	102 kDa	76	42	41	33	30	36	66	48	35	28	19	22
84	Neutral alpha-glucosidase AB OS=Homo sapiens OX=9606 GN=GANAB PE=1 SV=3	GANAB_HUMAN	GANAB	107 kDa	20	20	24	17	28	24	56	47	52	53	50	55
85	cAMP-dependent protein kinase type I-alpha regulatory subunit OS=Homo sapiens OX=9606 GN=PRKAR1A PE=1 SV=1	KAP0_HUMAN	PRKAR1A	43 kDa	48	39	42	39	43	44	43	40	43	36	38	41
86	Tripeptidyl-peptidase 2 OS=Homo sapiens OX=9606 GN=TPP2 PE=1 SV=4	TPP2_HUMAN	TPP2	138 kDa	21	50	34	34	54	35	47	68	40	37	47	36
87	Tropomyosin alpha-3 chain OS=Homo sapiens OX=9606 GN=TPM3 PE=1 SV=2	TPM3_HUMAN	TPM3	33 kDa	46	45	42	43	40	40	45	43	46	43	40	42
88	Disheveled-associated activator of morphogenesis 1 OS=Homo sapiens OX=9606 GN=DAAM1 PE=1 SV=2	DAAM1_HUMAN	DAAM1	123 kDa	44	34	38	37	36	31	37	34	27	35	37	36

89	T-complex protein 1 subunit beta OS=Homo sapiens OX=9606 GN=CCT2 PE=1 SV=4	TCPB_HUMAN	CCT2	57 kDa	48	44	44	46	52	59	39	51	28	25	30	29
90	UDP-glucose:glycoprotein glucosyltransferase 1 OS=Homo sapiens OX=9606 GN=UGGT1 PE=1 SV=3	UGGG1_HUMAN	UGGT1	177 kDa	29	30	29	26	25	31	51	54	54	49	57	61
91	Keratin, type I cytoskeletal 9 OS=Homo sapiens OX=9606 GN=KRT9 PE=1 SV=3	K1C9_HUMAN	KRT9	62 kDa	30	55	36	24	41	33	60	62	35	50	33	29
92	T-complex protein 1 subunit theta OS=Homo sapiens OX=9606 GN=CCT8 PE=1 SV=4	TCPQ_HUMAN	CCT8	60 kDa	39	45	42	39	44	45	30	46	33	36	37	35
93	Acetylcholinesterase OS=Homo sapiens OX=9606 GN=ACHE PE=1 SV=1	ACES_HUMAN	ACHE	68 kDa	32	28	35	36	29	34	34	27	37	38	34	34
94	Proteasome subunit alpha type-7 OS=Homo sapiens OX=9606 GN=PSMA7 PE=1 SV=1	PSA7_HUMAN	PSMA7	28 kDa	32	47	35	41	56	37	38	53	31	40	41	33
95	Proteasome subunit beta type-1 OS=Homo sapiens OX=9606 GN=PSMB1 PE=1 SV=2	PSB1_HUMAN	PSMB1	26 kDa	32	42	39	41	43	38	36	43	37	38	39	38
96	Guanine nucleotide-binding protein G(s) subunit alpha isoforms short OS=Homo sapiens OX=9606 GN=GNAS PE=1 SV=1	GNAS2_HUMAN	GNAS	46 kDa	53	43	50	48	45	43	43	40	41	45	39	43
97	Proteasome activator complex subunit 2 OS=Homo sapiens OX=9606 GN=PSME2 PE=1 SV=4	PSME2_HUMAN	PSME2	27 kDa	38	43	39	41	50	49	38	41	42	43	37	43
98	Polypyrimidine tract-binding protein 1 OS=Homo sapiens OX=9606 GN=PTBP1 PE=1 SV=1	PTBP1_HUMAN	PTBP1	57 kDa	37	35	39	38	36	43	42	35	39	40	43	32
99	Long-chain-fatty-acid--CoA ligase 6 OS=Homo sapiens OX=9606 GN=ACSL6 PE=2 SV=4	ACSL6_HUMAN	ACSL6	78 kDa	29	36	33	33	37	36	39	39	39	35	43	46
100	Proteasome subunit beta type-5 OS=Homo sapiens OX=9606 GN=PSMB5 PE=1 SV=3	PSB5_HUMAN	PSMB5	28 kDa	36	47	45	46	48	39	46	49	32	37	43	43
101	Stress-induced-phosphoprotein 1 OS=Homo sapiens OX=9606 GN=STIP1 PE=1 SV=1	STIP1_HUMAN	STIP1	63 kDa	27	31	34	37	49	48	25	36	27	24	35	22
102	Neuropathy target esterase OS=Homo sapiens OX=9606 GN=PNPLA6 PE=1 SV=3	PLPL6_HUMAN	PNPLA6	151 kDa	40	29	32	27	35	36	46	34	37	45	36	47
103	Multidrug resistance-associated protein 1 OS=Homo sapiens OX=9606 GN=ABCC1 PE=1 SV=3	MRP1_HUMAN	ABCC1	172 kDa	28	30	38	36	30	31	38	29	39	32	36	33
104	26S proteasome regulatory subunit 6A OS=Homo sapiens OX=9606 GN=PSMC3 PE=1 SV=3	PRS6A_HUMAN	PSMC3	49 kDa	29	40	35	48	39	39	25	39	24	36	35	27
105	Proteasome subunit beta type-6 OS=Homo sapiens OX=9606 GN=PSMB6 PE=1 SV=4	PSB6_HUMAN	PSMB6	25 kDa	22	38	36	37	39	45	29	38	22	19	37	19
106	Proteasome subunit beta type-3 OS=Homo sapiens OX=9606 GN=PSMB3 PE=1 SV=2	PSB3_HUMAN	PSMB3	23 kDa	26	38	31	38	44	37	27	32	20	32	32	25
107	26S proteasome non-ATPase regulatory subunit 13 OS=Homo sapiens OX=9606 GN=PSMD13 PE=1 SV=2	PSD13_HUMAN	PSMD13	43 kDa	38	42	36	37	42	44	29	40	31	35	34	37
108	Semaphorin-7A OS=Homo sapiens OX=9606 GN=SEMA7A PE=1 SV=1	SEM7A_HUMAN	SEMA7A	75 kDa	31	33	33	37	32	37	51	49	46	47	43	42
109	Dynamin-2 OS=Homo sapiens OX=9606 GN=DNM2 PE=1 SV=2	DYN2_HUMAN	DNM2	98 kDa	16	28	36	35	43	30	26	34	37	32	35	38
110	26S proteasome non-ATPase regulatory subunit 3 OS=Homo sapiens OX=9606 GN=PSMD3 PE=1 SV=2	PSMD3_HUMAN	PSMD3	61 kDa	32	44	38	40	38	27	34	39	27	33	33	29
111	Ezrin OS=Homo sapiens OX=9606 GN=EZR PE=1 SV=4	EZRI_HUMAN	EZR	69 kDa	36	30	35	32	39	36	37	30	32	27	33	28
112	AP-2 complex subunit mu OS=Homo sapiens OX=9606 GN=AP2M1 PE=1 SV=2	AP2M1_HUMAN	AP2M1	50 kDa	37	30	44	50	43	44	23	34	28	26	26	30
113	Immunoglobulin kappa light chain OS=Homo sapiens OX=9606 PE=1 SV=1	IGK_HUMAN		23 kDa	44	36	30	36	50	50	46	40	33	38	50	48
114	Nucleosome assembly protein 1-like 4 OS=Homo sapiens OX=9606 GN=NAP1L4 PE=1 SV=1	NP1L4_HUMAN	NAP1L4	43 kDa	38	48	41	47	52	43	36	43	29	34	36	36
115	Vacuolar protein sorting-associated protein 13A OS=Homo sapiens OX=9606 GN=VPS13A PE=1 SV=2	VP13A_HUMAN	VPS13A	360 kDa	21	21	30	20	23	22	35	31	34	36	29	33
116	Proteasome activator complex subunit 1 OS=Homo sapiens OX=9606 GN=PSME1 PE=1 SV=1	PSME1_HUMAN	PSME1	29 kDa	27	39	33	41	40	38	28	37	34	36	32	35
117	cAMP-dependent protein kinase catalytic subunit alpha OS=Homo sapiens OX=9606 GN=PRKACA PE=1 SV=2	KAPCA_HUMAN	PRKACA	41 kDa	43	41	37	40	39	33	25	32	29	34	32	25
118	Calreticulin OS=Homo sapiens OX=9606 GN=CALR PE=1 SV=1	CALR_HUMAN	CALR	48 kDa	32	28	29	26	27	24	42	38	44	39	38	48
119	Calpain-5 OS=Homo sapiens OX=9606 GN=CAPN5 PE=1 SV=2	CAN5_HUMAN	CAPN5	73 kDa	31	26	24	25	30	23	43	42	38	41	38	35
120	DnaJ homolog subfamily C member 13 OS=Homo sapiens OX=9606 GN=DNAJC13 PE=1 SV=5	DJC13_HUMAN	DNAJC13	254 kDa	16	33	38	50	40	52	14	19	24	23	35	33
121	Sodium/potassium-transporting ATPase subunit alpha-1 OS=Homo sapiens OX=9606 GN=ATP1A1 PE=1 SV=1	AT1A1_HUMAN	ATP1A1	113 kDa	26	35	26	37	37	31	34	39	32	31	36	31
122	26S proteasome regulatory subunit 6B OS=Homo sapiens OX=9606 GN=PSMC4 PE=1 SV=2	PRS6B_HUMAN	PSMC4	47 kDa	26	40	35	26	38	36	26	32	28	32	31	31
123	Importin-7 OS=Homo sapiens OX=9606 GN=IPO7 PE=1 SV=1	IPO7_HUMAN	IPO7	120 kDa	40	27	37	29	38	33	34	37	24	26	30	37
124	Proteasome subunit alpha type-1 OS=Homo sapiens OX=9606 GN=PSMA1 PE=1 SV=1	PSA1_HUMAN	PSMA1	30 kDa	30	37	36	36	39	30	26	29	27	26	34	25
125	Elongation factor 1-alpha 1 OS=Homo sapiens OX=9606 GN=EEF1A1 PE=1 SV=1	EF1A1_HUMAN	EEF1A1	50 kDa	36	38	35	35	34	34	29	22	23	22	31	29
126	Keratin, type II cytoskeletal 2 epidermal OS=Homo sapiens OX=9606 GN=KRT2 PE=1 SV=2	K22E_HUMAN	KRT2	65 kDa	18	42	31	45	22	26	41	40	36	24	28	18
127	Ras-related protein Rab-35 OS=Homo sapiens OX=9606 GN=RAB35 PE=1 SV=1	RAB35_HUMAN	RAB35	23 kDa	48	39	39	56	39	40	33	44	40	39	33	41
128	Ribose-phosphate pyrophosphokinase 1 OS=Homo sapiens OX=9606 GN=PRPS1 PE=1 SV=2	PRPS1_HUMAN	PRPS1	35 kDa	25	23	32	30	42	37	22	28	35	35	28	39
129	Basigin OS=Homo sapiens OX=9606 GN=BSG PE=1 SV=2	BASI_HUMAN	BSG	42 kDa	41	37	44	42	39	38	39	35	41	35	35	37
130	Atlastin-3 OS=Homo sapiens OX=9606 GN=ATL3 PE=1 SV=1	ATLA3_HUMAN	ATL3	61 kDa	38	35	36	30	33	27	34	29	33	32	30	31
131	Cell division control protein 42 homolog OS=Homo sapiens OX=9606 GN=CDC42 PE=1 SV=2	CDC42_HUMAN	CDC42	21 kDa	38	37	34	39	39	32	35	35	36	31	36	32
132	26S proteasome regulatory subunit 8 OS=Homo sapiens OX=9606 GN=PSMC5 PE=1 SV=1	PRS8_HUMAN	PSMC5	46 kDa	28	40	39	36	40	34	24	33	32	32	30	29
133	26S proteasome non-ATPase regulatory subunit 6 OS=Homo sapiens OX=9606 GN=PSMD6 PE=1 SV=1	PSMD6_HUMAN	PSMD6	46 kDa	25	34	31	37	39	40	23	30	24	28	34	32
134	Endoplasmin OS=Homo sapiens OX=9606 GN=HSP90B1 PE=1 SV=1	ENPL_HUMAN	HSP90B1	92 kDa	34	28	27	25	31	31	35	35	31	31	27	42
135	Dynactin subunit 1 OS=Homo sapiens OX=9606 GN=DCTN1 PE=1 SV=3	DCTN1_HUMAN	DCTN1	142 kDa	13	29	25	25	35	24	17	34	27	35	42	38
136	ATP-dependent 6-phosphofructokinase, muscle type OS=Homo sapiens OX=9606 GN=PFKM PE=1 SV=2	PFKAM_HUMAN	PFKM	85 kDa	21	29	27	39	41	43	20	29	30	41	32	45
137	Endoplasmic reticulum chaperone BiP OS=Homo sapiens OX=9606 GN=HSPA5 PE=1 SV=2	BIP_HUMAN	HSPA5	72 kDa	26	33	34	27	31	31	42	49	34	38	41	34
138	Ras-related protein Rab-10 OS=Homo sapiens OX=9606 GN=RAB10 PE=1 SV=1	RAB10_HUMAN	RAB10	23 kDa	36	40	33	31	35	33	19	27	24	24	27	21
139	Proteasome subunit alpha type-6 OS=Homo sapiens OX=9606 GN=PSMA6 PE=1 SV=1	PSA6_HUMAN	PSMA6	27 kDa	22	38	33	34	37	28	29	32	34	30	28	25
140	Proteasome subunit alpha type-2 OS=Homo sapiens OX=9606 GN=PSMA2 PE=1 SV=2	PSA2_HUMAN	PSMA2	26 kDa	35	33	31	35	41	31	25	24	30	33	26	35
141	Coiled-coil and C2 domain-containing protein 1A OS=Homo sapiens OX=9606 GN=CC2D1A PE=1 SV=1	C2D1A_HUMAN	CC2D1A	104 kDa	30	35	39	29	30	30	19	21	22	20	18	29
142	Complement receptor type 1 OS=Homo sapiens OX=9606 GN=CR1 PE=1 SV=3	CR1_HUMAN	CR1	224 kDa	12	25	24	26	28	24	43	38	41	36	32	31
143	ATP-dependent 6-phosphofructokinase, liver type OS=Homo sapiens OX=9606 GN=PFKL PE=1 SV=6	PFKAL_HUMAN	PFKL	85 kDa	33	34	35	42	35	41	25	38	37	39	35	36
144	Proteasome subunit beta type-2 OS=Homo sapiens OX=9606 GN=PSMB2 PE=1 SV=1	PSB2_HUMAN	PSMB2	23 kDa	26	35	27	30	35	21	26	25	21	30	28	24
145	GTPase KRas OS=Homo sapiens OX=9606 GN=KRAS PE=1 SV=1	RASK_HUMAN	KRAS	22 kDa	38	31	32	35	34	27	31	32	31	33	27	34
146	Proteasome adapter and scaffold protein ECM29 OS=Homo sapiens OX=9606 GN=ECPAS PE=1 SV=2	ECM29_HUMAN	ECPAS	204 kDa	22	30	28	31	34	40	19	27	13	21	23	24
147	26S proteasome regulatory subunit 4 OS=Homo sapiens OX=9606 GN=PSMC1 PE=1 SV=1	PRS4_HUMAN	PSMC1	49 kDa	32	28	35	41	44	34	27	31	33	34	26	32
148	Flavin reductase (NADPH) OS=Homo sapiens OX=9606 GN=BLVRB PE=1 SV=3	BLVRB_HUMAN	BLVRB	22 kDa	34	32	31	27	29	33	36	31	34	35	30	29
149	Protein disulfide-isomerase A3 OS=Homo sapiens OX=9606 GN=PDIA3 PE=1 SV=4	PDIA3_HUMAN	PDIA3	57 kDa	30	24	25	23	27	27	45	36	38	37	36	35
150	Immunoglobulin heavy constant gamma 2 OS=Homo sapiens OX=9606 GN=IGHG2 PE=1 SV=2	IGHG2_HUMAN	IGHG2	36 kDa	49	49	39	46	45	55	41	43	35	42	52	43
151	Proteasome subunit alpha type-4 OS=Homo sapiens OX=9606 GN=PSMA4 PE=1 SV=1	PSA4_HUMAN	PSMA4	29 kDa	23	23	25	28	31	25	27	26	19	31	28	22
152	Calnexin OS=Homo sapiens OX=9606 GN=CANX PE=1 SV=2	CALX_HUMAN	CANX	68 kDa	22	23	22	17	18	15	38	35	34	34	37	38
153	Deoxyribose-phosphate aldolase OS=Homo sapiens OX=9606 GN=DERA PE=1 SV=2	DEOC_HUMAN	DERA	35 kDa	29	32	30	30	35	35	23	22	19	24	19	26
154	26S proteasome non-ATPase regulatory subunit 5 OS=Homo sapiens OX=9606 GN=PSMD5 PE=1 SV=3	PSMD5_HUMAN	PSMD5	56 kDa	24	28	25	26	33	34	16	34	18	22	34	37
155	Alpha-centractin OS=Homo sapiens OX=9606 GN=ACTR1A PE=1 SV=1	ACTZ_HUMAN	ACTR1A	43 kDa	18	29	30	31	30	23	29	31	29	28	28	31
156	Copine-3 OS=Homo sapiens OX=9606 GN=CPNE3 PE=1 SV=1	CPNE3_HUMAN	CPNE3	60 kDa	17	27	20	13	21	15	35	35	37	27	36	17
157	Proteasome subunit beta type-7 OS=Homo sapiens OX=9606 GN=PSMB7 PE=1 SV=1	PSB7_HUMAN	PSMB7	30 kDa	17	32	29	32	42	21	16	20	12	18	20	21
158	Phosphatidylinositide phosphatase SAC1 OS=Homo sapiens OX=9606 GN=SACM1L PE=1 SV=2	SAC1_HUMAN	SACM1L	67 kDa	26	20	24	22	20	21	26	23	29	24	23	27
159	Copper-transporting ATPase 1 OS=Homo sapiens OX=9606 GN=ATP7A PE=1 SV=4	ATP7A_HUMAN	ATP7A	163 kDa	22	20	24	19	15	21	30	27	31	29	33	27
160	Glycophorin-A OS=Homo sapiens OX=9606 GN=GYPA PE=1 SV=2	GLPA_HUMAN	GYPA	16 kDa	32	27	28	25	28	26	29	27	28	30	27	27
161	26S proteasome non-ATPase regulatory subunit 11 OS=Homo sapiens OX=9606 GN=PSMD11 PE=1 SV=3	PSD11_HUMAN	PSMD11	47 kDa	26	32	26	29	29	23	23	29	18	21	24	24
162	Tensin-1 OS=Homo sapiens OX=9606 GN=TNS1 PE=1 SV=2	TENS1_HUMAN	TNS1	186 kDa	24	24	20	26	31	26	24	26	27	27	30	23
163	Plasma membrane calcium-transporting ATPase 1 OS=Homo sapiens OX=9606 GN=ATP2B1 PE=1 SV=4	AT2B1_HUMAN	ATP2B1	135 kDa	60	56	54	62	53	62	62	55	55	62	64	68
164	Hemoglobin subunit delta OS=Homo sapiens OX=9606 GN=HBD PE=1 SV=2	HBD_HUMAN	HBD	16 kDa	57	69	66	62	64	76	85	77	80	66	61	63
165	ATP-binding cassette sub-family G member 2 OS=Homo sapiens OX=9606 GN=ABCG2 PE=1 SV=3	ABCG2_HUMAN	ABCG2	72 kDa	28	24	24	25	21	31	29	31	27	27	27	27
166	Calpastatin OS=Homo sapiens OX=9606 GN=CAST PE=1 SV=4	ICAL_HUMAN	CAST	77 kDa	20	24	24	23	32	24	29	36	24	21	34	22
167	TBC1 domain family member 24 OS=Homo sapiens OX=9606 GN=TBC1D24 PE=1 SV=2	TBC24_HUMAN	TBC1D24	63 kDa	17	21	25	22	27	22	17	30	31	24	29	30
168	Intercellular adhesion molecule 4 OS=Homo sapiens OX=9606 GN=ICAM4 PE=1 SV=1	ICAM4_HUMAN	ICAM4	29 kDa	17	23	27	20	27	25	22	25	29	28	28	28
169	Annexin A7 OS=Homo sapiens OX=9606 GN=ANXA7 PE=1 SV=3	ANXA7_HUMAN	ANXA7	53 kDa	29	27	25	25	29	23	23	27	21	21	24	24
170	Equilibrative nucleoside transporter 1 OS=Homo sapiens OX=9606 GN=SLC29A1 PE=1 SV=3	S29A1_HUMAN	SLC29A1	50 kDa	19	17	20	17	16	16	29	21	25	24	27	31
171	Guanine nucleotide-binding protein subunit alpha-13 OS=Homo sapiens OX=9606 GN=GNA13 PE=1 SV=2	GNA13_HUMAN	GNA13	44 kDa	26	19	24	22	25	21	24	15	16	18	20	22
172	Gamma-adducin OS=Homo sapiens OX=9606 GN=ADD3 PE=1 SV=1	ADDG_HUMAN	ADD3	79 kDa	19	15	15	18	16	16	23	25	24	24	24	23
173	Cullin-1 OS=Homo sapiens OX=9606 GN=CUL1 PE=1 SV=2	CUL1_HUMAN	CUL1	90 kDa	15	27	21	23	33	22	14	24	19	21	28	26
174	Proteasome subunit alpha type-3 OS=Homo sapiens OX=9606 GN=PSMA3 PE=1 SV=2	PSA3_HUMAN	PSMA3	28 kDa	19	25	24	24	32	27	27	27	16	16	25	25
175	Importin subunit beta-1 OS=Homo sapiens OX=9606 GN=KPNB1 PE=1 SV=2	IMB1_HUMAN	KPNB1	97 kDa	24	23	23	19	32	22	25	31	18	17	20	26
176	Methyltransferase-like protein 7A OS=Homo sapiens OX=9606 GN=METTL7A PE=1 SV=1	MET7A_HUMAN	METTL7A	28 kDa	16	12	17	19	17	19	24	27	27	25	29	36
177	Ubiquitin carboxyl-terminal hydrolase 15 OS=Homo sapiens OX=9606 GN=USP15 PE=1 SV=3	UBP15_HUMAN	USP15	112 kDa	21	21	32	15	35	26	21	25	11	16	23	25
178	Annexin A4 OS=Homo sapiens OX=9606 GN=ANXA4 PE=1 SV=4	ANXA4_HUMAN	ANXA4	36 kDa	23	19	27	17	19	18	23	24	27	30	20	18
179	Casein kinase I isoform alpha OS=Homo sapiens OX=9606 GN=CSNK1A1 PE=1 SV=2	KC1A_HUMAN	CSNK1A1	39 kDa	21	23	25	19	24	26	37	27	24	25	27	25
180	Complement decay-accelerating factor OS=Homo sapiens OX=9606 GN=CD55 PE=1 SV=4	DAF_HUMAN	CD55	41 kDa	21	21	20	27	21	23	22	23	23	26	28	22

181	Ubiquitin-60S ribosomal protein L40 OS=Homo sapiens OX=9606 GN=UBA52 PE=1 SV=2	RL40_HUMAN (+3)	UBA52	15 kDa	24	21	23	27	22	20	21	17	19	18	18	18
182	AP-2 complex subunit alpha-2 OS=Homo sapiens OX=9606 GN=AP2A2 PE=1 SV=2	AP2A2_HUMAN	AP2A2	104 kDa	45	51	49	52	54	63	37	37	34	41	41	41
183	26S proteasome regulatory subunit 10B OS=Homo sapiens OX=9606 GN=PSMC6 PE=1 SV=1	PRS10_HUMAN	PSMC6	44 kDa	23	24	26	26	24	25	23	24	18	23	21	22
184	Radixin OS=Homo sapiens OX=9606 GN=RDX PE=1 SV=1	RADI_HUMAN	RDX	69 kDa	33	31	35	31	37	33	39	31	39	35	30	32
185	14-3-3 protein epsilon OS=Homo sapiens OX=9606 GN=YWHAE PE=1 SV=1	1433E_HUMAN	YWHAE	29 kDa	17	20	23	19	22	19	31	32	20	22	24	20
186	WD repeat-containing protein 81 OS=Homo sapiens OX=9606 GN=WDR81 PE=1 SV=2	WDR81_HUMAN	WDR81	212 kDa	18	25	19	25	31	27	8	17	11	18	16	20
187	Importin-5 OS=Homo sapiens OX=9606 GN=IPO5 PE=1 SV=4	IPO5_HUMAN	IPO5	124 kDa	24	27	24	25	39	44	6	13	6	5	8	13
188	Vesicle-associated membrane protein-associated protein A OS=Homo sapiens OX=9606 GN=VAPA PE=1 SV=3	VAPA_HUMAN	VAPA	28 kDa	16	22	26	19	19	21	25	27	20	28	25	15
189	Phosphatidylinositol 4-kinase type 2-alpha OS=Homo sapiens OX=9606 GN=PI4K2A PE=1 SV=1	P4K2A_HUMAN	PI4K2A	54 kDa	21	11	21	23	24	18	22	15	27	22	25	21
190	Fatty acid synthase OS=Homo sapiens OX=9606 GN=FASN PE=1 SV=3	FAS_HUMAN	FASN	273 kDa	19	27	13	20	36	27	11	24	7	6	17	17
191	Unconventional myosin-XVIIIa OS=Homo sapiens OX=9606 GN=MYO18A PE=1 SV=3	MY18A_HUMAN	MYO18A	233 kDa	7	26	17	21	29	19	13	20	14	21	27	23
192	Myosin light chain 4 OS=Homo sapiens OX=9606 GN=MYL4 PE=1 SV=3	MYL4_HUMAN	MYL4	22 kDa	22	27	31	32	32	29	19	17	17	24	21	20
193	26S proteasome non-ATPase regulatory subunit 14 OS=Homo sapiens OX=9606 GN=PSMD14 PE=1 SV=1	PSDE_HUMAN	PSMD14	35 kDa	17	19	17	21	22	20	13	23	12	13	15	18
194	Neutral cholesterol ester hydrolase 1 OS=Homo sapiens OX=9606 GN=NCEH1 PE=1 SV=3	NCEH1_HUMAN	NCEH1	46 kDa	21	16	20	17	14	20	26	21	25	23	25	25
195	Immunoglobulin heavy constant gamma 3 OS=Homo sapiens OX=9606 GN=IGHG3 PE=1 SV=2	IGHG3_HUMAN	IGHG3	41 kDa	49	56	45	52	56	60	52	56	59	55	61	51
196	Long-chain-fatty-acid--CoA ligase 3 OS=Homo sapiens OX=9606 GN=ACSL3 PE=1 SV=3	ACSL3_HUMAN	ACSL3	80 kDa	18	15	17	13	13	18	28	27	24	23	22	24
197	Ras-related C3 botulinum toxin substrate 1 OS=Homo sapiens OX=9606 GN=RAC1 PE=1 SV=1	RAC1_HUMAN	RAC1	21 kDa	29	24	27	27	24	26	16	14	20	22	17	23
198	Protein XRP2 OS=Homo sapiens OX=9606 GN=RP2 PE=1 SV=4	XRP2_HUMAN	RP2	40 kDa	15	15	17	13	16	13	22	18	21	26	18	16
199	26S proteasome non-ATPase regulatory subunit 12 OS=Homo sapiens OX=9606 GN=PSMD12 PE=1 SV=3	PSD12_HUMAN	PSMD12	53 kDa	23	24	27	23	27	26	12	20	14	18	18	21
200	Vesicular integral-membrane protein VIP36 OS=Homo sapiens OX=9606 GN=LMAN2 PE=1 SV=1	LMAN2_HUMAN	LMAN2	40 kDa	30	17	15	17	10	21	18	16	15	15	13	17
201	Ras-related protein Rab-8A OS=Homo sapiens OX=9606 GN=RAB8A PE=1 SV=1	RAB8A_HUMAN	RAB8A	24 kDa	28	24	20	31	29	28	25	28	23	25	24	17
202	Myosin regulatory light chain 12B OS=Homo sapiens OX=9606 GN=MYL12B PE=1 SV=2	ML12B_HUMAN	MYL12B	20 kDa	18	37	26	44	43	25	12	12	12	9	15	12
203	Beta-2-glycoprotein 1 OS=Homo sapiens OX=9606 GN=APOH PE=1 SV=3	APOH_HUMAN	APOH	38 kDa	23	16	15	23	17	17	18	10	15	13	19	21
204	COP9 signalosome complex subunit 4 OS=Homo sapiens OX=9606 GN=COPS4 PE=1 SV=1	CSN4_HUMAN	COPS4	46 kDa	17	20	16	11	20	19	18	26	13	15	17	18
205	Endoplasmic reticulum resident protein 44 OS=Homo sapiens OX=9606 GN=ERP44 PE=1 SV=1	ERP44_HUMAN	ERP44	47 kDa	20	11	20	19	24	17	19	19	17	18	17	24
206	Peptidyl-prolyl cis-trans isomerase FKBP3 OS=Homo sapiens OX=9606 GN=FKBP3 PE=1 SV=1	FKBP3_HUMAN	FKBP3	25 kDa	27	17	25	25	24	25	17	18	13	16	13	17
207	26S proteasome non-ATPase regulatory subunit 7 OS=Homo sapiens OX=9606 GN=PSMD7 PE=1 SV=2	PSMD7_HUMAN	PSMD7	37 kDa	15	23	23	19	26	27	14	20	15	16	20	21
208	COP9 signalosome complex subunit 2 OS=Homo sapiens OX=9606 GN=COPS2 PE=1 SV=1	CSN2_HUMAN	COPS2	52 kDa	13	20	12	15	20	21	19	19	15	21	15	19
209	LanC-like protein 2 OS=Homo sapiens OX=9606 GN=LANCL2 PE=1 SV=1	LANC2_HUMAN	LANCL2	51 kDa	5	18	22	20	23	22	17	17	18	25	25	31
210	Intermediate conductance calcium-activated potassium channel protein 4 OS=Homo sapiens OX=9606 GN=KCNN4 PE=1 SV=1	KCNN4_HUMAN	KCNN4	48 kDa	21	20	14	25	22	19	19	18	20	21	17	19
211	Blood group Rh(D) polypeptide OS=Homo sapiens OX=9606 GN=RHD PE=1 SV=3	RHD_HUMAN	RHD	45 kDa	39	34	34	36	32	31	26	37	36	40	29	35
212	Actin, alpha cardiac muscle 1 OS=Homo sapiens OX=9606 GN=ACTC1 PE=1 SV=1	ACTC_HUMAN	ACTC1	42 kDa	63	71	63	66	71	70	84	78	81	75	91	72
213	Calpain-1 catalytic subunit OS=Homo sapiens OX=9606 GN=CAPN1 PE=1 SV=1	CAN1_HUMAN	CAPN1	82 kDa	13	17	19	13	18	14	11	17	10	11	7	14
214	Immunoglobulin heavy constant mu OS=Homo sapiens OX=9606 GN=IGHM PE=1 SV=4	IGHM_HUMAN	IGHM	49 kDa	10	16	13	15	20	18	18	20	19	16	25	19
215	Guanine nucleotide-binding protein G(q) subunit alpha OS=Homo sapiens OX=9606 GN=GNAQ PE=1 SV=4	GNAQ_HUMAN	GNAQ	42 kDa	16	14	18	21	17	19	20	19	20	19	21	20
216	Syntaxin-7 OS=Homo sapiens OX=9606 GN=STX7 PE=1 SV=4	STX7_HUMAN	STX7	30 kDa	15	25	22	21	21	19	19	18	23	23	18	20
217	Urea transporter 1 OS=Homo sapiens OX=9606 GN=SLC14A1 PE=1 SV=2	UT1_HUMAN	SLC14A1	43 kDa	14	10	12	13	13	16	18	19	14	18	16	19
218	Galectin-3 OS=Homo sapiens OX=9606 GN=LGALS3 PE=1 SV=5	LEG3_HUMAN	LGALS3	26 kDa	18	17	15	14	14	19	19	20	17	20	19	13
219	Phospholipid transfer protein C2CD2L OS=Homo sapiens OX=9606 GN=C2CD2L PE=1 SV=3	C2C2L_HUMAN	C2CD2L	76 kDa	15	19	19	20	19	20	22	14	15	12	19	18
220	Dynactin subunit 2 OS=Homo sapiens OX=9606 GN=DCTN2 PE=1 SV=4	DCTN2_HUMAN	DCTN2	44 kDa	6	21	13	18	18	9	13	19	16	17	24	17
221	G protein-coupled receptor kinase 6 OS=Homo sapiens OX=9606 GN=GRK6 PE=1 SV=2	GRK6_HUMAN	GRK6	66 kDa	17	12	19	14	12	18	16	15	15	14	12	15
222	Immunoglobulin lambda constant 2 OS=Homo sapiens OX=9606 GN=IGLC2 PE=1 SV=1	IGLC2_HUMAN (+1)	IGLC2	11 kDa	23	14	13	16	22	19	16	16	15	17	19	22
223	Protein arginine N-methyltransferase 5 OS=Homo sapiens OX=9606 GN=PRMT5 PE=1 SV=4	ANM5_HUMAN	PRMT5	73 kDa	7	21	15	12	21	16	11	13	8	8	10	8
224	Gamma-aminobutyric acid receptor-associated protein-like 2 OS=Homo sapiens OX=9606 GN=GABARAPL2 PE=1 SV=1	GBRL2_HUMAN	GABARAPL2	14 kDa	15	16	16	14	17	15	9	17	17	16	14	14
225	Heat shock 70 kDa protein 4 OS=Homo sapiens OX=9606 GN=HSPA4 PE=1 SV=4	HSP74_HUMAN	HSPA4	94 kDa	10	20	17	11	26	15	21	24	18	17	15	12
226	Ras-related protein Rab-5C OS=Homo sapiens OX=9606 GN=RAB5C PE=1 SV=2	RAB5C_HUMAN	RAB5C	23 kDa	15	17	14	15	16	15	21	22	19	19	16	14
227	Serine/threonine-protein kinase WNK1 OS=Homo sapiens OX=9606 GN=WNK1 PE=1 SV=2	WNK1_HUMAN	WNK1	251 kDa	5	13	16	14	17	10	9	17	12	15	20	20
228	Solute carrier family 40 member 1 OS=Homo sapiens OX=9606 GN=SLC40A1 PE=1 SV=1	S40A1_HUMAN	SLC40A1	63 kDa	16	14	18	20	16	18	24	19	19	18	18	17
229	Peptidyl-prolyl cis-trans isomerase B OS=Homo sapiens OX=9606 GN=PPIB PE=1 SV=2	PPIB_HUMAN	PPIB	24 kDa	11	14	13	14	13	10	15	17	15	19	16	18
230	F-actin-capping protein subunit beta OS=Homo sapiens OX=9606 GN=CAPZB PE=1 SV=4	CAPZB_HUMAN	CAPZB	31 kDa	11	15	17	20	19	20	17	18	13	20	18	16
231	Very-long-chain 3-oxoacyl-CoA reductase OS=Homo sapiens OX=9606 GN=HSD17B12 PE=1 SV=2	DHB12_HUMAN	HSD17B12	34 kDa	15	14	16	16	17	15	20	18	20	21	15	19
232	SH3 domain-binding glutamic acid-rich-like protein OS=Homo sapiens OX=9606 GN=SH3BGRL PE=1 SV=1	SH3L1_HUMAN	SH3BGRL	13 kDa	22	18	21	21	20	15	15	16	19	18	17	19
233	WD repeat-containing protein 91 OS=Homo sapiens OX=9606 GN=WDR91 PE=1 SV=2	WDR91_HUMAN	WDR91	83 kDa	15	18	17	13	22	26	5	16	9	14	17	19
234	Carbonic anhydrase 1 OS=Homo sapiens OX=9606 GN=CA1 PE=1 SV=2	CAH1_HUMAN	CA1	29 kDa	18	20	16	17	16	15	21	24	22	17	10	13
235	Peroxiredoxin-1 OS=Homo sapiens OX=9606 GN=PRDX1 PE=1 SV=1	PRDX1_HUMAN	PRDX1	22 kDa	19	21	16	19	26	23	23	24	15	14	19	19
236	Guanine nucleotide-binding protein G(I)/G(S)/G(T) subunit beta-2 OS=Homo sapiens OX=9606 GN=GNB2 PE=1 SV=3	GBB2_HUMAN	GNB2	37 kDa	53	44	44	45	45	29	28	38	44	41	44	59
237	BMP-2-inducible protein kinase OS=Homo sapiens OX=9606 GN=BMP2K PE=1 SV=2	BMP2K_HUMAN	BMP2K	129 kDa	16	12	11	14	13	14	10	17	12	14	14	19
238	Carnitine O-palmitoyltransferase 1, liver isoform OS=Homo sapiens OX=9606 GN=CPT1A PE=1 SV=2	CPT1A_HUMAN	CPT1A	88 kDa	6	11	8	5	10	7	22	19	22	22	22	19
239	Junctional adhesion molecule A OS=Homo sapiens OX=9606 GN=F11R PE=1 SV=1	JAM1_HUMAN	F11R	33 kDa	14	16	17	14	13	16	16	15	18	24	18	19
240	DnaJ homolog subfamily B member 1 OS=Homo sapiens OX=9606 GN=DNAJB1 PE=1 SV=4	DNJB1_HUMAN	DNAJB1	38 kDa	13	11	17	14	17	16	13	15	14	11	15	15
241	Moesin OS=Homo sapiens OX=9606 GN=MSN PE=1 SV=3	MOES_HUMAN	MSN	68 kDa	35	29	34	31	29	29	31	22	36	23	26	30
242	Ubiquitin carboxyl-terminal hydrolase 14 OS=Homo sapiens OX=9606 GN=USP14 PE=1 SV=3	UBP14_HUMAN	USP14	56 kDa	12	19	11	13	22	17	12	17	17	10	15	17
243	Vesicle-fusing ATPase OS=Homo sapiens OX=9606 GN=NSF PE=1 SV=3	NSF_HUMAN	NSF	83 kDa	11	10	15	7	18	22	13	15	11	7	10	14
244	CB1 cannabinoid receptor-interacting protein 1 OS=Homo sapiens OX=9606 GN=CNRIP1 PE=1 SV=1	CNRP1_HUMAN	CNRIP1	19 kDa	5	17	16	18	18	17	9	14	17	17	16	18
245	AP-1 complex subunit beta-1 OS=Homo sapiens OX=9606 GN=AP1B1 PE=1 SV=2	AP1B1_HUMAN	AP1B1	105 kDa	46	55	52	52	59	53	37	42	46	41	47	33
246	COP9 signalosome complex subunit 3 OS=Homo sapiens OX=9606 GN=COPS3 PE=1 SV=3	CSN3_HUMAN	COPS3	48 kDa	15	14	13	12	16	16	19	16	12	14	17	11
247	Transforming protein RhoA OS=Homo sapiens OX=9606 GN=RHOA PE=1 SV=1	RHOA_HUMAN	RHOA	22 kDa	16	12	16	18	18	13	11	15	12	15	9	16
248	Syntaxin-binding protein 3 OS=Homo sapiens OX=9606 GN=STXBP3 PE=1 SV=2	STXB3_HUMAN	STXBP3	68 kDa	9	11	8	12	12	10	20	19	24	19	15	17
249	Guanine nucleotide-binding protein G(k) subunit alpha OS=Homo sapiens OX=9606 GN=GNAI3 PE=1 SV=3	GNAI3_HUMAN	GNAI3	41 kDa	60	62	54	61	54	53	65	68	57	58	54	64
250	Monocarboxylate transporter 1 OS=Homo sapiens OX=9606 GN=SLC16A1 PE=1 SV=3	MOT1_HUMAN	SLC16A1	54 kDa	15	12	17	16	12	13	16	12	12	13	11	10
251	Small integral membrane protein 1 OS=Homo sapiens OX=9606 GN=SMIM1 PE=1 SV=1	SMIM1_HUMAN	SMIM1	9 kDa	13	15	13	8	14	11	25	21	13	14	18	14
252	Phosphoribosyl pyrophosphate synthase-associated protein 2 OS=Homo sapiens OX=9606 GN=PRPSAP2 PE=1 SV=1	KPRB_HUMAN	PRPSAP2	41 kDa	10	16	10	11	15	18	9	13	14	12	15	13
253	Ras-related protein Rab-21 OS=Homo sapiens OX=9606 GN=RAB21 PE=1 SV=3	RAB21_HUMAN	RAB21	24 kDa	14	11	14	15	12	14	14	14	19	15	16	16
254	Zinc transporter 1 OS=Homo sapiens OX=9606 GN=SLC30A1 PE=1 SV=3	ZNT1_HUMAN	SLC30A1	55 kDa	9	14	12	13	12	11	13	15	12	18	14	14
255	Ras-related protein Rab-7a OS=Homo sapiens OX=9606 GN=RAB7A PE=1 SV=1	RAB7A_HUMAN	RAB7A	23 kDa	12	12	13	15	10	14	12	15	15	15	17	13
256	Serine/threonine-protein phosphatase 6 catalytic subunit OS=Homo sapiens OX=9606 GN=PPP6C PE=1 SV=1	PPP6_HUMAN	PPP6C	35 kDa	10	13	16	16	18	13	8	9	13	12	10	18
257	Calmodulin-1 OS=Homo sapiens OX=9606 GN=CALM1 PE=1 SV=1	CALM1_HUMAN (+2)	CALM1	17 kDa	11	11	12	10	9	14	19	15	14	17	15	10
258	Serine/threonine-protein phosphatase 6 regulatory subunit 1 OS=Homo sapiens OX=9606 GN=PPP6R1 PE=1 SV=5	PP6R1_HUMAN	PPP6R1	97 kDa	13	20	15	14	19	16	9	13	10	11	10	13
259	Hsc70-interacting protein OS=Homo sapiens OX=9606 GN=ST13 PE=1 SV=2	F10A1_HUMAN	ST13	41 kDa	14	9	10	9	14	11	12	13	10	12	8	10
260	SH3 domain-binding glutamic acid-rich-like protein 2 OS=Homo sapiens OX=9606 GN=SH3BGRL2 PE=1 SV=2	SH3L2_HUMAN	SH3BGRL2	12 kDa	7	6	10	9	9	5	15	19	19	14	18	12
261	Phospholipid-transporting ATPase IG OS=Homo sapiens OX=9606 GN=ATP11C PE=1 SV=3	AT11C_HUMAN	ATP11C	129 kDa	8	10	10	8	13	8	13	13	11	10	13	10
262	COP9 signalosome complex subunit 1 OS=Homo sapiens OX=9606 GN=GPS1 PE=1 SV=4	CSN1_HUMAN	GPS1	56 kDa	11	12	9	8	16	16	15	19	8	9	15	15
263	26S proteasome non-ATPase regulatory subunit 8 OS=Homo sapiens OX=9606 GN=PSMD8 PE=1 SV=2	PSMD8_HUMAN	PSMD8	40 kDa	11	12	7	14	10	14	9	9	7	6	7	11
264	RuvB-like 2 OS=Homo sapiens OX=9606 GN=RUVBL2 PE=1 SV=3	RUVB2_HUMAN	RUVBL2	51 kDa	12	16	15	16	18	25	8	14	7	6	9	12
265	Membrane transport protein XK OS=Homo sapiens OX=9606 GN=XK PE=1 SV=5	XK_HUMAN	XK	51 kDa	7	9	9	9	10	7	11	7	11	10	10	12
266	MAP/microtubule affinity-regulating kinase 3 OS=Homo sapiens OX=9606 GN=MARK3 PE=1 SV=5	MARK3_HUMAN	MARK3	84 kDa	15	14	9	12	18	12	17	14	13	14	13	9
267	Protein diaphanous homolog 1 OS=Homo sapiens OX=9606 GN=DIAPH1 PE=1 SV=2	DIAP1_HUMAN	DIAPH1	141 kDa	7	12	6	5	6	9	14	23	10	13	20	15
268	Beta-actin-like protein 2 OS=Homo sapiens OX=9606 GN=ACTBL2 PE=1 SV=2	ACTBL_HUMAN	ACTBL2	42 kDa	38	38	36	38	37	41	45	39	43	43	45	46
269	Heat shock protein HSP 90-beta OS=Homo sapiens OX=9606 GN=HSP90AB1 PE=1 SV=4	HS90B_HUMAN	HSP90AB1	83 kDa	31	39	34	34	50	38	27	31	18	24	32	22
270	Malignant T-cell-amplified sequence 1 OS=Homo sapiens OX=9606 GN=MCTS1 PE=1 SV=1	MCTS1_HUMAN	MCTS1	21 kDa	9	14	12	15	12	14	6	5	12	10	7	13
271	ATP-dependent RNA helicase A OS=Homo sapiens OX=9606 GN=DHX9 PE=1 SV=4	DHX9_HUMAN	DHX9	141 kDa	11	15	13	15	20	18	5	8	11	10	9	12
272	Syntaxin-binding protein 2 OS=Homo sapiens OX=9606 GN=STXBP2 PE=1 SV=2	STXB2_HUMAN	STXBP2	66 kDa	6	12	6	6	9	8	20	18	16	21	17	15

273	Cytoskeleton-associated protein 5 OS=Homo sapiens OX=9606 GN=CKAP5 PE=1 SV=3	CKAP5_HUMAN	CKAP5	226 kDa	20	13	18	21	18	19	5	2	4	4	3	7
274	Fructosamine-3-kinase OS=Homo sapiens OX=9606 GN=FN3K PE=1 SV=1	FN3K_HUMAN	FN3K	35 kDa	13	13	15	9	12	15	10	17	13	12	12	14
275	Multiple inositol polyphosphate phosphatase 1 OS=Homo sapiens OX=9606 GN=MINPP1 PE=1 SV=1	MINP1_HUMAN	MINPP1	55 kDa	11	10	9	9	6	9	15	15	12	12	13	10
276	Transmembrane protein 222 OS=Homo sapiens OX=9606 GN=TMEM222 PE=1 SV=2	TM222_HUMAN	TMEM222	23 kDa	13	15	11	14	13	11	20	19	13	19	18	15
277	F-actin-capping protein subunit alpha-1 OS=Homo sapiens OX=9606 GN=CAPZA1 PE=1 SV=3	CAZA1_HUMAN	CAPZA1	33 kDa	6	12	10	11	18	14	12	8	11	18	15	15
278	Phosphatidylinositol-binding clathrin assembly protein OS=Homo sapiens OX=9606 GN=PICALM PE=1 SV=2	PICAL_HUMAN	PICALM	71 kDa	12	10	11	10	10	10	9	14	15	16	14	9
279	Vesicle-trafficking protein SEC22b OS=Homo sapiens OX=9606 GN=SEC22B PE=1 SV=4	SC22B_HUMAN	SEC22B	25 kDa	12	10	11	13	14	14	14	10	12	10	14	18
280	Epoxide hydrolase 1 OS=Homo sapiens OX=9606 GN=EPHX1 PE=1 SV=1	HYEP_HUMAN	EPHX1	53 kDa	3	9	10	5	7	6	11	11	10	12	14	9
281	Protein disulfide-isomerase A6 OS=Homo sapiens OX=9606 GN=PDIA6 PE=1 SV=1	PDIA6_HUMAN	PDIA6	48 kDa	5	8	8	8	10	8	13	15	13	13	14	14
282	DNA damage-binding protein 1 OS=Homo sapiens OX=9606 GN=DDB1 PE=1 SV=1	DDB1_HUMAN	DDB1	127 kDa	5	5	7	3	13	10	16	20	9	8	11	8
283	Hydroxyacylglutathione hydrolase, mitochondrial OS=Homo sapiens OX=9606 GN=HAGH PE=1 SV=2	GLO2_HUMAN	HAGH	34 kDa	14	13	12	11	9	11	12	11	15	15	7	9
284	L-lactate dehydrogenase B chain OS=Homo sapiens OX=9606 GN=LDHB PE=1 SV=2	LDHB_HUMAN	LDHB	37 kDa	10	8	11	8	14	15	12	14	8	8	8	10
285	Ras-related protein Rab-2B OS=Homo sapiens OX=9606 GN=RAB2B PE=1 SV=1	RAB2B_HUMAN	RAB2B	24 kDa	13	10	11	16	13	16	12	9	10	6	10	13
286	Multidrug resistance-associated protein 5 OS=Homo sapiens OX=9606 GN=ABCC5 PE=1 SV=2	MRP5_HUMAN	ABCC5	161 kDa	11	12	14	14	14	13	15	9	8	11	9	14
287	ATP-binding cassette sub-family E member 1 OS=Homo sapiens OX=9606 GN=ABCE1 PE=1 SV=1	ABCE1_HUMAN	ABCE1	67 kDa	10	8	16	12	12	10	10	11	10	7	7	7
288	Phosphoglycerate kinase 1 OS=Homo sapiens OX=9606 GN=PGK1 PE=1 SV=3	PGK1_HUMAN	PGK1	45 kDa	3	10	13	12	12	12	3	15	17	18	12	16
289	Thioredoxin domain-containing protein 5 OS=Homo sapiens OX=9606 GN=TXNDC5 PE=1 SV=2	TXND5_HUMAN	TXNDC5	48 kDa	6	9	10	8	11	10	19	13	15	15	16	13
290	Phospholipid hydroperoxide glutathione peroxidase OS=Homo sapiens OX=9606 GN=GPX4 PE=1 SV=3	GPX4_HUMAN	GPX4	22 kDa	10	5	10	7	8	10	10	8	8	8	8	14
291	Exportin-7 OS=Homo sapiens OX=9606 GN=XPO7 PE=1 SV=3	XPO7_HUMAN	XPO7	124 kDa	8	13	7	7	18	17	9	18	6	6	14	11
292	Glucosidase 2 subunit beta OS=Homo sapiens OX=9606 GN=PRKCSH PE=1 SV=2	GLU2B_HUMAN	PRKCSH	59 kDa	5	6	8	7	8	8	15	17	14	15	15	16
293	Band 4.1-like protein 2 OS=Homo sapiens OX=9606 GN=EPB41L2 PE=1 SV=1	E41L2_HUMAN	EPB41L2	113 kDa	11	8	6	9	9	7	9	7	11	12	6	9
294	Syntaxin-4 OS=Homo sapiens OX=9606 GN=STX4 PE=1 SV=2	STX4_HUMAN	STX4	34 kDa	11	9	8	7	9	9	15	14	14	13	15	12
295	E3 ubiquitin-protein ligase RNF123 OS=Homo sapiens OX=9606 GN=RNF123 PE=1 SV=1	RN123_HUMAN	RNF123	149 kDa	7	11	9	3	19	14	13	20	8	6	11	17
296	Staphylococcal nuclease domain-containing protein 1 OS=Homo sapiens OX=9606 GN=SND1 PE=1 SV=1	SND1_HUMAN	SND1	102 kDa	14	12	21	17	21	18	3	0	0	0	0	0
297	Serine/threonine-protein kinase OSR1 OS=Homo sapiens OX=9606 GN=OXSR1 PE=1 SV=1	OXSR1_HUMAN	OXSR1	58 kDa	9	13	8	7	16	14	4	11	9	10	12	12
298	CD44 antigen OS=Homo sapiens OX=9606 GN=CD44 PE=1 SV=3	CD44_HUMAN	CD44	82 kDa	12	6	6	13	5	8	15	9	11	10	11	14
299	EH domain-binding protein 1-like protein 1 OS=Homo sapiens OX=9606 GN=EHBP1L1 PE=1 SV=2	EH1L1_HUMAN	EHBP1L1	162 kDa	4	11	10	12	11	5	11	18	10	9	11	9
300	Nucleosome assembly protein 1-like 1 OS=Homo sapiens OX=9606 GN=NAP1L1 PE=1 SV=1	NP1L1_HUMAN	NAP1L1	45 kDa	16	19	15	16	15	18	13	19	14	14	16	14
301	Very-long-chain (3R)-3-hydroxyacyl-CoA dehydratase 3 OS=Homo sapiens OX=9606 GN=HACD3 PE=1 SV=2	HACD3_HUMAN	HACD3	43 kDa	8	11	8	9	9	5	13	10	13	11	11	13
302	Pyrroline-5-carboxylate reductase 3 OS=Homo sapiens OX=9606 GN=PYCR3 PE=1 SV=3	P5CR3_HUMAN	PYCR3	29 kDa	8	10	11	10	15	17	7	9	12	13	7	14
303	Small membrane A-kinase anchor protein OS=Homo sapiens OX=9606 GN=C2orf88 PE=1 SV=2	SMAKA_HUMAN	C2orf88	11 kDa	14	13	13	10	9	8	11	11	9	9	10	8
304	V-type proton ATPase catalytic subunit A OS=Homo sapiens OX=9606 GN=ATP6V1A PE=1 SV=2	VATA_HUMAN	ATP6V1A	68 kDa	8	13	11	8	13	17	9	10	10	11	10	10
305	Alpha-soluble NSF attachment protein OS=Homo sapiens OX=9606 GN=NAPA PE=1 SV=3	SNAA_HUMAN	NAPA	33 kDa	5	11	10	15	16	15	8	11	7	9	9	11
306	RuvB-like 1 OS=Homo sapiens OX=9606 GN=RUVBL1 PE=1 SV=1	RUVB1_HUMAN	RUVBL1	50 kDa	13	10	13	8	16	15	7	7	6	5	12	9
307	Phenylalanine--tRNA ligase beta subunit OS=Homo sapiens OX=9606 GN=FARSB PE=1 SV=3	SYFB_HUMAN	FARSB	66 kDa	13	12	16	16	17	15	4	4	0	2	0	8
308	V-type proton ATPase 116 kDa subunit a isoform 1 OS=Homo sapiens OX=9606 GN=ATP6V0A1 PE=1 SV=3	VPP1_HUMAN	ATP6V0A1	96 kDa	6	4	6	2	0	5	12	14	18	16	19	11
309	Calcium-binding protein 39 OS=Homo sapiens OX=9606 GN=CAB39 PE=1 SV=1	CAB39_HUMAN	CAB39	40 kDa	5	9	7	10	8	8	8	10	8	10	6	9
310	V-type proton ATPase subunit B, brain isoform OS=Homo sapiens OX=9606 GN=ATP6V1B2 PE=1 SV=3	VATB2_HUMAN	ATP6V1B2	57 kDa	11	14	9	10	13	10	14	13	7	5	12	9
311	Hemoglobin subunit gamma-2 OS=Homo sapiens OX=9606 GN=HBG2 PE=1 SV=2	HBG2_HUMAN	HBG2	16 kDa	15	18	13	16	14	14	24	21	25	18	13	13
312	AP-2 complex subunit sigma OS=Homo sapiens OX=9606 GN=AP2S1 PE=1 SV=2	AP2S1_HUMAN	AP2S1	17 kDa	9	11	11	12	12	13	8	12	7	8	6	7
313	RNA 3'-terminal phosphate cyclase OS=Homo sapiens OX=9606 GN=RTCA PE=1 SV=1	RTCA_HUMAN	RTCA	39 kDa	9	10	10	9	10	10	6	8	9	9	7	12
314	Diacylglycerol kinase alpha OS=Homo sapiens OX=9606 GN=DGKA PE=1 SV=3	DGKA_HUMAN	DGKA	83 kDa	0	5	8	10	10	7	4	9	8	10	14	11
315	Protein DDI1 homolog 2 OS=Homo sapiens OX=9606 GN=DDI2 PE=1 SV=1	DDI2_HUMAN	DDI2	45 kDa	12	14	10	9	11	11	12	14	11	10	11	11
316	Perilipin-3 OS=Homo sapiens OX=9606 GN=PLIN3 PE=1 SV=3	PLIN3_HUMAN	PLIN3	47 kDa	3	8	6	7	6	14	6	8	10	13	16	10
317	Reticulon-3 OS=Homo sapiens OX=9606 GN=RTN3 PE=1 SV=2	RTN3_HUMAN	RTN3	113 kDa	14	6	11	16	14	12	9	9	12	10	8	12
318	Retinal dehydrogenase 1 OS=Homo sapiens OX=9606 GN=ALDH1A1 PE=1 SV=2	AL1A1_HUMAN	ALDH1A1	55 kDa	6	7	6	4	13	10	18	19	7	7	13	8
319	Protein VAC14 homolog OS=Homo sapiens OX=9606 GN=VAC14 PE=1 SV=1	VAC14_HUMAN	VAC14	88 kDa	2	6	10	8	11	11	6	9	6	12	14	17
320	Saccharopine dehydrogenase-like oxidoreductase OS=Homo sapiens OX=9606 GN=SCCPDH PE=1 SV=1	SCPDL_HUMAN	SCCPDH	47 kDa	10	11	7	8	6	11	8	10	11	15	14	12
321	Reticulon-4 OS=Homo sapiens OX=9606 GN=RTN4 PE=1 SV=2	RTN4_HUMAN	RTN4	130 kDa	13	14	14	12	16	16	8	7	6	7	8	5
322	STE20-related kinase adapter protein beta OS=Homo sapiens OX=9606 GN=STRADB PE=1 SV=1	STRAB_HUMAN	STRADB	47 kDa	6	7	6	6	7	6	15	8	12	11	10	11
323	Transmembrane emp24 domain-containing protein 10 OS=Homo sapiens OX=9606 GN=TMED10 PE=1 SV=2	TMEDA_HUMAN	TMED10	25 kDa	9	10	8	9	11	10	9	9	9	12	12	9
324	Calpain small subunit 1 OS=Homo sapiens OX=9606 GN=CAPNS1 PE=1 SV=1	CPNS1_HUMAN	CAPNS1	28 kDa	7	9	10	10	13	9	7	8	5	5	7	11
325	Ras-related protein Rab-18 OS=Homo sapiens OX=9606 GN=RAB18 PE=1 SV=1	RAB18_HUMAN	RAB18	23 kDa	9	9	9	11	6	8	11	12	7	7	14	8
326	E3 ubiquitin-protein ligase KCMF1 OS=Homo sapiens OX=9606 GN=KCMF1 PE=1 SV=2	KCMF1_HUMAN	KCMF1	42 kDa	4	10	12	8	9	8	10	10	11	15	12	11
327	Transcriptional activator protein Pur-alpha OS=Homo sapiens OX=9606 GN=PURA PE=1 SV=2	PURA_HUMAN	PURA	35 kDa	10	13	9	11	10	13	11	13	8	12	14	9
328	Vesicle-associated membrane protein-associated protein B/C OS=Homo sapiens OX=9606 GN=VAPB PE=1 SV=3	VAPB_HUMAN	VAPB	27 kDa	10	18	13	13	10	11	9	10	11	10	15	12
329	Solute carrier family 2, facilitated glucose transporter member 14 OS=Homo sapiens OX=9606 GN=SLC2A14 PE=2 SV=1	GTR14_HUMAN	SLC2A14	56 kDa	6	4	7	6	8	5	11	6	11	9	6	11
330	Heat shock protein beta-1 OS=Homo sapiens OX=9606 GN=HSPB1 PE=1 SV=2	HSPB1_HUMAN	HSPB1	23 kDa	8	15	14	12	15	16	5	6	7	6	5	5
331	COP9 signalosome complex subunit 6 OS=Homo sapiens OX=9606 GN=COPS6 PE=1 SV=1	CSN6_HUMAN	COPS6	36 kDa	6	3	7	8	11	6	15	16	7	6	8	7
332	Phenylalanine--tRNA ligase alpha subunit OS=Homo sapiens OX=9606 GN=FARSA PE=1 SV=3	SYFA_HUMAN	FARSA	58 kDa	11	8	13	16	11	14	6	2	3	4	4	6
333	Leucine-rich repeat-containing protein 57 OS=Homo sapiens OX=9606 GN=LRRC57 PE=1 SV=1	LRC57_HUMAN	LRRC57	27 kDa	7	9	6	9	7	5	9	8	6	5	3	6
334	Arf-GAP with SH3 domain, ANK repeat and PH domain-containing protein 1 OS=Homo sapiens OX=9606 GN=ASAP1 PE=1 SV=4	ASAP1_HUMAN	ASAP1	126 kDa	8	9	10	9	13	10	2	7	3	7	6	5
335	Annexin A11 OS=Homo sapiens OX=9606 GN=ANXA11 PE=1 SV=1	ANX11_HUMAN	ANXA11	54 kDa	5	6	6	9	9	7	11	10	9	7	8	9
336	GMP reductase 1 OS=Homo sapiens OX=9606 GN=GMPR PE=1 SV=1	GMPR1_HUMAN	GMPR	37 kDa	12	12	9	10	12	11	9	9	9	7	10	11
337	Large neutral amino acids transporter small subunit 3 OS=Homo sapiens OX=9606 GN=SLC43A1 PE=1 SV=1	LAT3_HUMAN	SLC43A1	61 kDa	10	11	8	10	10	6	13	14	9	9	11	8
338	Phosphatidate phosphatase LPIN2 OS=Homo sapiens OX=9606 GN=LPIN2 PE=1 SV=1	LPIN2_HUMAN	LPIN2	99 kDa	3	5	4	4	5	8	12	13	7	9	14	9
339	Solute carrier family 12 member 7 OS=Homo sapiens OX=9606 GN=SLC12A7 PE=1 SV=3	S12A7_HUMAN	SLC12A7	119 kDa	12	8	9	7	8	2	11	3	5	7	6	6
340	Adenylosuccinate lyase OS=Homo sapiens OX=9606 GN=ADSL PE=1 SV=2	PUR8_HUMAN	ADSL	55 kDa	3	6	7	16	14	13	5	7	8	10	10	7
341	Large neutral amino acids transporter small subunit 4 OS=Homo sapiens OX=9606 GN=SLC43A2 PE=1 SV=1	LAT4_HUMAN	SLC43A2	63 kDa	7	7	5	5	6	7	6	6	7	9	4	10
342	Protein ERGIC-53 OS=Homo sapiens OX=9606 GN=LMAN1 PE=1 SV=2	LMAN1_HUMAN	LMAN1	58 kDa	10	9	11	10	11	7	11	7	5	11	9	7
343	Methylosome protein 50 OS=Homo sapiens OX=9606 GN=WDR77 PE=1 SV=1	MEP50_HUMAN	WDR77	37 kDa	5	9	7	8	14	7	5	8	4	4	11	9
344	Leukocyte surface antigen CD47 OS=Homo sapiens OX=9606 GN=CD47 PE=1 SV=1	CD47_HUMAN	CD47	35 kDa	9	9	10	9	8	7	7	5	10	8	9	7
345	Ecto-ADP-ribosyltransferase 4 OS=Homo sapiens OX=9606 GN=ART4 PE=2 SV=2	NAR4_HUMAN	ART4	36 kDa	8	8	6	8	7	11	7	6	6	9	6	13
346	Phosphatidylinositol 4-phosphate 5-kinase type-1 alpha OS=Homo sapiens OX=9606 GN=PIP5K1A PE=1 SV=1	PI51A_HUMAN	PIP5K1A	63 kDa	7	9	7	9	8	6	9	12	8	6	10	8
347	COP9 signalosome complex subunit 5 OS=Homo sapiens OX=9606 GN=COPS5 PE=1 SV=4	CSN5_HUMAN	COPS5	38 kDa	5	8	9	7	10	8	10	10	5	6	5	8
348	Surfeit locus protein 4 OS=Homo sapiens OX=9606 GN=SURF4 PE=1 SV=3	SURF4_HUMAN	SURF4	30 kDa	7	7	14	8	9	6	11	8	7	7	8	6
349	Serine/threonine-protein phosphatase 2A 65 kDa regulatory subunit A alpha isoform OS=Homo sapiens OX=9606 GN=PPP2R1A PE=1 SV=4	2AAA_HUMAN	PPP2R1A	65 kDa	10	10	10	6	11	9	11	10	8	3	6	11
350	Rabankyrin-5 OS=Homo sapiens OX=9606 GN=ANKFY1 PE=1 SV=2	ANFY1_HUMAN	ANKFY1	128 kDa	5	12	10	4	11	8	10	20	3	8	10	5
351	26S proteasome non-ATPase regulatory subunit 4 OS=Homo sapiens OX=9606 GN=PSMD4 PE=1 SV=1	PSMD4_HUMAN	PSMD4	41 kDa	7	9	10	10	15	9	7	15	7	9	11	10
352	Vesicle-associated membrane protein 3 OS=Homo sapiens OX=9606 GN=VAMP3 PE=1 SV=3	VAMP3_HUMAN	VAMP3	11 kDa	13	10	9	13	8	9	9	7	7	8	8	11
353	Nicastrin OS=Homo sapiens OX=9606 GN=NCSTN PE=1 SV=2	NICA_HUMAN	NCSTN	78 kDa	8	11	6	10	7	7	11	8	6	4	10	11
354	CDGSH iron-sulfur domain-containing protein 2 OS=Homo sapiens OX=9606 GN=CISD2 PE=1 SV=1	CISD2_HUMAN	CISD2	15 kDa	6	9	6	5	4	6	10	10	7	7	8	9
355	Solute carrier family 2, facilitated glucose transporter member 4 OS=Homo sapiens OX=9606 GN=SLC2A4 PE=1 SV=1	GLUT4_HUMAN	SLC2A4	55 kDa	4	11	9	5	7	9	13	13	11	13	13	7
356	DnaJ homolog subfamily B member 4 OS=Homo sapiens OX=9606 GN=DNAJB4 PE=1 SV=1	DNJB4_HUMAN	DNAJB4	38 kDa	12	7	7	11	10	9	0	2	7	6	7	9
357	NSFL1 cofactor p47 OS=Homo sapiens OX=9606 GN=NSFL1C PE=1 SV=2	NSF1C_HUMAN	NSFL1C	41 kDa	3	7	6	6	10	9	6	8	5	5	5	7
358	Fructose-bisphosphate aldolase C OS=Homo sapiens OX=9606 GN=ALDOC PE=1 SV=2	ALDOC_HUMAN	ALDOC	39 kDa	29	28	26	31	29	33	25	26	31	31	30	33
359	Solute carrier family 43 member 3 OS=Homo sapiens OX=9606 GN=SLC43A3 PE=1 SV=2	S43A3_HUMAN	SLC43A3	55 kDa	8	3	6	6	5	7	7	3	6	6	5	8
360	Protein THEM6 OS=Homo sapiens OX=9606 GN=THEM6 PE=1 SV=2	THEM6_HUMAN	THEM6	24 kDa	2	4	3	3	3	2	8	8	9	9	7	3
361	Complement C4-B OS=Homo sapiens OX=9606 GN=C4B PE=1 SV=2	CO4B_HUMAN	C4B	193 kDa	9	11	4	4	6	3	8	12	13	16	13	12
362	Thioredoxin-related transmembrane protein 1 OS=Homo sapiens OX=9606 GN=TMX1 PE=1 SV=1	TMX1_HUMAN	TMX1	32 kDa	6	7	8	5	6	6	8	7	9	10	7	13
363	Transmembrane emp24 domain-containing protein 7 OS=Homo sapiens OX=9606 GN=TMED7 PE=1 SV=2	TMED7_HUMAN	TMED7	25 kDa	14	10	12	10	6	8	6	6	8	8	9	12
364	Hypoxia up-regulated protein 1 OS=Homo sapiens OX=9606 GN=HYOU1 PE=1 SV=1	HYOU1_HUMAN	HYOU1	111 kDa	4	3	4	0	0	0	11	14	12	7	11	8

365	GTP-binding nuclear protein Ran OS=Homo sapiens OX=9606 GN=RAN PE=1 SV=3	RAN_HUMAN	RAN	24 kDa	9	6	10	7	13	11	5	8	7	7	9	10
366	Calcium and integrin-binding protein 1 OS=Homo sapiens OX=9606 GN=CIB1 PE=1 SV=4	CIB1_HUMAN	CIB1	22 kDa	3	8	6	7	5	6	10	11	9	9	8	9
367	CD59 glycoprotein OS=Homo sapiens OX=9606 GN=CD59 PE=1 SV=1	CD59_HUMAN	CD59	14 kDa	6	4	7	7	6	4	8	9	11	8	7	7
368	Lymphocyte function-associated antigen 3 OS=Homo sapiens OX=9606 GN=CD58 PE=1 SV=1	LFA3_HUMAN	CD58	28 kDa	11	9	9	8	9	7	8	11	10	7	10	7
369	Vasodilator-stimulated phosphoprotein OS=Homo sapiens OX=9606 GN=VASP PE=1 SV=3	VASP_HUMAN	VASP	40 kDa	6	6	7	7	8	7	10	8	7	6	4	7
370	Extended synaptotagmin-1 OS=Homo sapiens OX=9606 GN=ESYT1 PE=1 SV=1	ESYT1_HUMAN	ESYT1	123 kDa	6	4	8	3	4	7	11	7	12	12	12	9
371	Transmembrane emp24 domain-containing protein 2 OS=Homo sapiens OX=9606 GN=TMED2 PE=1 SV=1	TMED2_HUMAN	TMED2	23 kDa	12	7	8	8	9	5	12	9	9	9	8	5
372	14-3-3 protein zeta/delta OS=Homo sapiens OX=9606 GN=YWHAZ PE=1 SV=1	1433Z_HUMAN	YWHAZ	28 kDa	10	15	13	9	12	9	18	18	19	15	16	15
373	MAGUK p55 subfamily member 6 OS=Homo sapiens OX=9606 GN=MPP6 PE=1 SV=2	MPP6_HUMAN	MPP6	61 kDa	6	3	4	6	3	3	11	9	5	7	5	8
374	Serine/threonine-protein phosphatase 6 regulatory ankyrin repeat subunit A OS=Homo sapiens OX=9606 GN=ANKRD28 PE=1 SV=5	ANR28_HUMAN	ANKRD28	113 kDa	8	6	8	8	9	12	3	8	0	4	6	7
375	F-box only protein 7 OS=Homo sapiens OX=9606 GN=FBXO7 PE=1 SV=1	FBX7_HUMAN	FBXO7	59 kDa	6	5	5	0	7	7	10	7	6	4	4	4
376	Large proline-rich protein BAG6 OS=Homo sapiens OX=9606 GN=BAG6 PE=1 SV=2	BAG6_HUMAN	BAG6	119 kDa	6	10	10	18	15	15	0	0	0	0	2	0
377	Chitinase domain-containing protein 1 OS=Homo sapiens OX=9606 GN=CHID1 PE=1 SV=1	CHID1_HUMAN	CHID1	45 kDa	2	7	5	7	3	4	9	7	9	6	6	9
378	Phospholipid scramblase 1 OS=Homo sapiens OX=9606 GN=PLSCR1 PE=1 SV=1	PLS1_HUMAN	PLSCR1	35 kDa	6	10	9	7	8	4	7	8	8	9	9	9
379	Receptor expression-enhancing protein 5 OS=Homo sapiens OX=9606 GN=REEP5 PE=1 SV=3	REEP5_HUMAN	REEP5	21 kDa	9	8	9	8	7	8	7	10	8	10	4	5
380	Peptidyl-prolyl cis-trans isomerase NIMA-interacting 4 OS=Homo sapiens OX=9606 GN=PIN4 PE=1 SV=1	PIN4_HUMAN	PIN4	14 kDa	9	7	8	11	7	11	3	6	6	6	3	6
381	Galectin-9 OS=Homo sapiens OX=9606 GN=LGALS9 PE=1 SV=2	LEG9_HUMAN	LGALS9	40 kDa	4	6	8	6	9	6	7	11	12	10	11	11
382	Ubiquitin carboxyl-terminal hydrolase 5 OS=Homo sapiens OX=9606 GN=USP5 PE=1 SV=2	UBP5_HUMAN	USP5	96 kDa	6	4	7	3	9	5	9	11	3	3	6	10
383	Serine hydroxymethyltransferase, cytosolic OS=Homo sapiens OX=9606 GN=SHMT1 PE=1 SV=1	GLYC_HUMAN	SHMT1	53 kDa	13	8	11	10	5	8	7	7	9	5	7	4
384	Membrane-associated progesterone receptor component 2 OS=Homo sapiens OX=9606 GN=PGRMC2 PE=1 SV=1	PGRC2_HUMAN	PGRMC2	24 kDa	5	7	9	6	8	5	8	11	9	8	7	8
385	Vesicle transport protein SEC20 OS=Homo sapiens OX=9606 GN=BNIP1 PE=1 SV=3	SEC20_HUMAN	BNIP1	26 kDa	8	5	8	4	7	6	8	5	5	7	3	4
386	Synaptosomal-associated protein 23 OS=Homo sapiens OX=9606 GN=SNAP23 PE=1 SV=1	SNP23_HUMAN	SNAP23	23 kDa	5	5	6	7	6	3	10	10	9	10	10	6
387	Neuroblastoma-amplified sequence OS=Homo sapiens OX=9606 GN=NBAS PE=1 SV=2	NBAS_HUMAN	NBAS	269 kDa	9	9	9	11	7	10	5	3	3	5	4	4
388	Transmembrane and coiled-coil domains protein 2 OS=Homo sapiens OX=9606 GN=TMCC2 PE=1 SV=3	TMCC2_HUMAN	TMCC2	77 kDa	8	8	6	5	11	7	6	9	10	6	6	6
389	Proliferation-associated protein 2G4 OS=Homo sapiens OX=9606 GN=PA2G4 PE=1 SV=3	PA2G4_HUMAN	PA2G4	44 kDa	4	4	7	10	14	8	3	4	4	3	2	7
390	Interferon-induced protein with tetratricopeptide repeats 5 OS=Homo sapiens OX=9606 GN=IFIT5 PE=1 SV=1	IFIT5_HUMAN	IFIT5	56 kDa	4	6	6	5	3	8	5	9	7	9	8	10
391	Transmembrane 9 superfamily member 3 OS=Homo sapiens OX=9606 GN=TM9SF3 PE=1 SV=2	TM9S3_HUMAN	TM9SF3	68 kDa	8	7	12	11	10	9	5	6	6	7	5	4
392	Endoplasmic reticulum resident protein 29 OS=Homo sapiens OX=9606 GN=ERP29 PE=1 SV=4	ERP29_HUMAN	ERP29	29 kDa	4	5	5	4	4	5	12	9	12	8	10	7
393	Metallo-beta-lactamase domain-containing protein 2 OS=Homo sapiens OX=9606 GN=MBLAC2 PE=1 SV=3	MBLC2_HUMAN	MBLAC2	31 kDa	6	7	7	6	7	5	9	7	11	8	9	9
394	Protein lifeguard 3 OS=Homo sapiens OX=9606 GN=TMBIM1 PE=1 SV=2	LFG3_HUMAN	TMBIM1	35 kDa	10	5	6	7	8	7	9	6	7	7	7	8
395	Eukaryotic translation initiation factor 2 subunit 3 OS=Homo sapiens OX=9606 GN=EIF2S3 PE=1 SV=3	IF2G_HUMAN	EIF2S3	51 kDa	5	7	8	7	8	8	7	8	4	5	9	9
396	Obg-like ATPase 1 OS=Homo sapiens OX=9606 GN=OLA1 PE=1 SV=2	OLA1_HUMAN	OLA1	45 kDa	6	8	5	3	6	5	3	3	5	0	0	4
397	Eukaryotic translation initiation factor 2 subunit 1 OS=Homo sapiens OX=9606 GN=EIF2S1 PE=1 SV=3	IF2A_HUMAN	EIF2S1	36 kDa	3	8	8	7	8	8	4	5	0	4	10	10
398	Ras-related protein Ral-A OS=Homo sapiens OX=9606 GN=RALA PE=1 SV=1	RALA_HUMAN	RALA	24 kDa	6	3	5	5	6	4	4	4	7	3	3	5
399	Tyrosine-protein kinase SYK OS=Homo sapiens OX=9606 GN=SYK PE=1 SV=1	KSYK_HUMAN	SYK	72 kDa	4	3	5	5	4	5	2	6	6	10	6	8
400	Myosin light polypeptide 6 OS=Homo sapiens OX=9606 GN=MYL6 PE=1 SV=2	MYL6_HUMAN	MYL6	17 kDa	5	12	8	11	11	11	4	4	3	4	8	5
401	Endoplasmic reticulum-Golgi intermediate compartment protein 1 OS=Homo sapiens OX=9606 GN=ERGIC1 PE=1 SV=1	ERGI1_HUMAN	ERGIC1	33 kDa	5	5	7	7	6	5	9	0	6	4	5	6
402	Multifunctional protein ADE2 OS=Homo sapiens OX=9606 GN=PAICS PE=1 SV=3	PUR6_HUMAN	PAICS	47 kDa	3	0	2	2	2	6	11	7	4	5	6	12
403	Thioredoxin-related transmembrane protein 4 OS=Homo sapiens OX=9606 GN=TMX4 PE=1 SV=1	TMX4_HUMAN	TMX4	39 kDa	3	7	7	0	6	6	10	10	11	7	8	7
404	Programmed cell death protein 6 OS=Homo sapiens OX=9606 GN=PDCD6 PE=1 SV=1	PDCD6_HUMAN	PDCD6	22 kDa	0	4	4	5	5	5	6	8	8	7	7	5
405	Clathrin light chain A OS=Homo sapiens OX=9606 GN=CLTA PE=1 SV=1	CLCA_HUMAN	CLTA	27 kDa	0	5	2	0	5	2	3	3	3	3	4	3
406	Immunoglobulin heavy constant alpha 1 OS=Homo sapiens OX=9606 GN=IGHA1 PE=1 SV=2	IGHA1_HUMAN	IGHA1	38 kDa	9	8	6	4	7	5	4	7	6	4	7	6
407	COP9 signalosome complex subunit 8 OS=Homo sapiens OX=9606 GN=COPS8 PE=1 SV=1	CSN8_HUMAN	COPS8	23 kDa	3	6	5	3	9	4	3	9	6	6	6	4
408	Nuclear receptor-binding protein OS=Homo sapiens OX=9606 GN=NRBP1 PE=1 SV=1	NRBP_HUMAN	NRBP1	60 kDa	5	8	5	5	7	6	0	7	2	4	5	3
409	Ras-related protein Rab-5B OS=Homo sapiens OX=9606 GN=RAB5B PE=1 SV=1	RAB5B_HUMAN	RAB5B	24 kDa	9	14	15	16	15	14	17	17	15	15	12	15
410	14-3-3 protein gamma OS=Homo sapiens OX=9606 GN=YWHAG PE=1 SV=2	1433G_HUMAN	YWHAG	28 kDa	11	11	15	8	13	14	13	15	12	11	12	11
411	Microtubule-associated protein RP/EB family member 1 OS=Homo sapiens OX=9606 GN=MAPRE1 PE=1 SV=3	MARE1_HUMAN	MAPRE1	30 kDa	4	6	5	5	6	4	3	4	4	3	2	3
412	Transmembrane emp24 domain-containing protein 9 OS=Homo sapiens OX=9606 GN=TMED9 PE=1 SV=2	TMED9_HUMAN	TMED9	27 kDa	3	7	4	5	4	5	4	3	4	5	5	4
413	ADP-ribosylation factor 6 OS=Homo sapiens OX=9606 GN=ARF6 PE=1 SV=2	ARF6_HUMAN	ARF6	20 kDa	6	6	6	8	4	6	4	6	7	7	8	5
414	Centromere/kinetochore protein zw10 homolog OS=Homo sapiens OX=9606 GN=ZW10 PE=1 SV=3	ZW10_HUMAN	ZW10	89 kDa	3	7	4	5	5	8	4	0	4	4	7	3
415	Transmembrane protein 56 OS=Homo sapiens OX=9606 GN=TMEM56 PE=1 SV=1	TMM56_HUMAN	TMEM56	30 kDa	3	8	3	6	8	4	4	0	4	7	3	6
416	Transmembrane channel-like protein 8 OS=Homo sapiens OX=9606 GN=TMC8 PE=1 SV=1	TMC8_HUMAN	TMC8	82 kDa	5	3	8	6	4	6	7	5	4	5	6	5
417	Retinoic acid receptor responder protein 2 OS=Homo sapiens OX=9606 GN=RARRES2 PE=1 SV=1	RARR2_HUMAN	RARRES2	19 kDa	4	6	3	8	5	6	7	9	8	6	5	6
418	Guanine nucleotide-binding protein subunit alpha-11 OS=Homo sapiens OX=9606 GN=GNA11 PE=1 SV=2	GNA11_HUMAN	GNA11	42 kDa	7	8	8	9	10	13	10	12	14	9	14	15
419	Nectin-1 OS=Homo sapiens OX=9606 GN=NECTIN1 PE=1 SV=3	NECT1_HUMAN	NECTIN1	57 kDa	5	3	2	3	3	3	8	8	6	9	11	10
420	Serine/threonine-protein phosphatase 6 regulatory subunit 3 OS=Homo sapiens OX=9606 GN=PPP6R3 PE=1 SV=2	PP6R3_HUMAN	PPP6R3	98 kDa	4	8	0	0	4	6	5	10	3	4	6	7
421	Atypical chemokine receptor 1 OS=Homo sapiens OX=9606 GN=ACKR1 PE=1 SV=3	ACKR1_HUMAN	ACKR1	36 kDa	0	0	0	0	0	0	0	0	7	0	0	0
422	Purine nucleoside phosphorylase OS=Homo sapiens OX=9606 GN=PNP PE=1 SV=2	PNPH_HUMAN	PNP	32 kDa	4	3	0	0	10	2	12	9	6	4	4	7
423	Ras-related protein Rab-1A OS=Homo sapiens OX=9606 GN=RAB1A PE=1 SV=3	RAB1A_HUMAN	RAB1A	23 kDa	8	10	11	15	11	12	9	12	9	9	14	6
424	Adipocyte plasma membrane-associated protein OS=Homo sapiens OX=9606 GN=APMAP PE=1 SV=2	APMAP_HUMAN	APMAP	46 kDa	5	2	6	3	3	5	7	7	3	5	8	5
425	Mannose-P-dolichol utilization defect 1 protein OS=Homo sapiens OX=9606 GN=MPDU1 PE=1 SV=2	MPU1_HUMAN	MPDU1	27 kDa	0	5	4	4	4	3	4	0	5	4	5	6
426	14-3-3 protein beta/alpha OS=Homo sapiens OX=9606 GN=YWHAB PE=1 SV=3	1433B_HUMAN	YWHAB	28 kDa	13	14	14	11	16	11	16	17	16	15	13	14
427	ATP-binding cassette sub-family A member 7 OS=Homo sapiens OX=9606 GN=ABCA7 PE=1 SV=3	ABCA7_HUMAN	ABCA7	234 kDa	4	3	2	2	2	3	12	4	10	7	6	10
428	Protein-glutamine gamma-glutamyltransferase 2 OS=Homo sapiens OX=9606 GN=TGM2 PE=1 SV=2	TGM2_HUMAN	TGM2	77 kDa	3	3	2	2	9	5	8	6	5	2	3	7
429	Keratin, type I cytoskeletal 14 OS=Homo sapiens OX=9606 GN=KRT14 PE=1 SV=4	K1C14_HUMAN	KRT14	52 kDa	5	5	5	4	5	4	9	9	0	7	6	5
430	Actin-related protein 10 OS=Homo sapiens OX=9606 GN=ACTR10 PE=1 SV=1	ARP10_HUMAN	ACTR10	46 kDa	4	5	4	7	4	5	5	5	4	6	5	4
431	Tropomyosin alpha-1 chain OS=Homo sapiens OX=9606 GN=TPM1 PE=1 SV=2	TPM1_HUMAN	TPM1	33 kDa	19	22	20	21	19	18	27	23	23	25	19	20
432	Vacuolar protein sorting-associated protein 45 OS=Homo sapiens OX=9606 GN=VPS45 PE=1 SV=1	VPS45_HUMAN	VPS45	65 kDa	3	5	4	7	3	5	6	7	4	6	5	4
433	Vesicle transport protein GOT1B OS=Homo sapiens OX=9606 GN=GOLT1B PE=1 SV=1	GOT1B_HUMAN	GOLT1B	15 kDa	5	5	8	5	7	3	4	6	6	7	6	4
434	Lysophospholipid acyltransferase 2 OS=Homo sapiens OX=9606 GN=MBOAT2 PE=2 SV=2	MBOA2_HUMAN	MBOAT2	60 kDa	6	0	4	5	2	0	3	3	6	4	4	5
435	Protein GUCD1 OS=Homo sapiens OX=9606 GN=GUCD1 PE=1 SV=2	GUCD1_HUMAN	GUCD1	27 kDa	4	3	5	3	4	4	7	6	7	6	8	7
436	Ras-related protein Rab-14 OS=Homo sapiens OX=9606 GN=RAB14 PE=1 SV=4	RAB14_HUMAN	RAB14	24 kDa	11	7	9	11	11	11	7	0	4	4	6	3
437	Casein kinase I isoform gamma-3 OS=Homo sapiens OX=9606 GN=CSNK1G3 PE=1 SV=2	KC1G3_HUMAN	CSNK1G3	51 kDa	3	3	5	0	0	2	6	4	3	5	4	2
438	Guanine nucleotide-binding protein subunit beta-4 OS=Homo sapiens OX=9606 GN=GNB4 PE=1 SV=3	GBB4_HUMAN	GNB4	38 kDa	21	14	18	19	20	15	16	18	17	20	22	21
439	COP9 signalosome complex subunit 7a OS=Homo sapiens OX=9606 GN=COPS7A PE=1 SV=1	CSN7A_HUMAN	COPS7A	30 kDa	2	7	6	3	7	7	4	4	4	5	11	7
440	COP9 signalosome complex subunit 7b OS=Homo sapiens OX=9606 GN=COPS7B PE=1 SV=1	CSN7B_HUMAN	COPS7B	30 kDa	4	5	8	7	4	5	3	6	4	4	0	5
441	Ubiquitin-associated domain-containing protein 1 OS=Homo sapiens OX=9606 GN=UBAC1 PE=1 SV=1	UBAC1_HUMAN	UBAC1	45 kDa	3	2	4	2	7	5	8	5	2	0	6	6
442	Uncharacterized protein C1orf198 OS=Homo sapiens OX=9606 GN=C1orf198 PE=1 SV=1	CA198_HUMAN	C1orf198	36 kDa	7	5	6	5	7	3	4	3	4	5	5	5
443	Methanethiol oxidase OS=Homo sapiens OX=9606 GN=SELENBP1 PE=1 SV=2	SBP1_HUMAN	SELENBP1	52 kDa	4	2	5	6	6	8	3	3	5	4	0	2
444	Cullin-3 OS=Homo sapiens OX=9606 GN=CUL3 PE=1 SV=2	CUL3_HUMAN	CUL3	89 kDa	0	2	4	6	13	9	0	5	0	2	0	6
445	Atlastin-2 OS=Homo sapiens OX=9606 GN=ATL2 PE=1 SV=2	ATLA2_HUMAN	ATL2	66 kDa	6	7	7	10	6	6	4	4	5	5	3	8
446	Ras-related protein Rap-1b OS=Homo sapiens OX=9606 GN=RAP1B PE=1 SV=1	RAP1B_HUMAN	RAP1B	21 kDa	96	65	63	68	54	46	52	47	50	43	42	34
447	Sec1 family domain-containing protein 2 OS=Homo sapiens OX=9606 GN=SCFD2 PE=1 SV=2	SCFD2_HUMAN	SCFD2	75 kDa	4	7	6	7	6	6	4	0	5	3	4	7
448	ADP-ribosylation factor-like protein 15 OS=Homo sapiens OX=9606 GN=ARL15 PE=1 SV=1	ARL15_HUMAN	ARL15	23 kDa	3	4	3	4	5	0	9	5	5	5	4	5
449	Tyrosine--tRNA ligase, cytoplasmic OS=Homo sapiens OX=9606 GN=YARS PE=1 SV=4	SYYC_HUMAN	YARS	59 kDa	0	0	0	4	2	3	3	5	3	9	12	8
450	RELT-like protein 1 OS=Homo sapiens OX=9606 GN=RELL1 PE=1 SV=1	RELL1_HUMAN	RELL1	29 kDa	6	4	5	3	7	5	5	4	4	6	7	4
451	Dual specificity mitogen-activated protein kinase kinase 3 OS=Homo sapiens OX=9606 GN=MAP2K3 PE=1 SV=2	MP2K3_HUMAN	MAP2K3	39 kDa	0	3	3	6	6	11	2	4	2	4	3	3
452	Cullin-4A OS=Homo sapiens OX=9606 GN=CUL4A PE=1 SV=3	CUL4A_HUMAN	CUL4A	88 kDa	3	3	3	3	0	6	0	12	4	3	7	5
453	Endophilin-B2 OS=Homo sapiens OX=9606 GN=SH3GLB2 PE=1 SV=1	SHLB2_HUMAN	SH3GLB2	44 kDa	0	2	3	3	6	5	0	6	5	8	6	6
454	Extended synaptotagmin-2 OS=Homo sapiens OX=9606 GN=ESYT2 PE=1 SV=1	ESYT2_HUMAN	ESYT2	102 kDa	0	3	3	2	4	5	7	7	3	9	7	7
455	Cell cycle control protein 50A OS=Homo sapiens OX=9606 GN=TMEM30A PE=1 SV=1	CC50A_HUMAN	TMEM30A	41 kDa	2	2	2	4	0	3	7	5	8	6	4	6
456	Keratin, type II cytoskeletal 6A OS=Homo sapiens OX=9606 GN=KRT6A PE=1 SV=3	K2C6A_HUMAN	KRT6A	60 kDa	7	9	0	10	4	0	13	15	0	9	10	6

457	CSC1-like protein 2 OS=Homo sapiens OX=9606 GN=TMEM63B PE=1 SV=1	CSCL2_HUMAN	TMEM63B	95 kDa	0	0	0	0	0	0	7	5	3	7	5	5
458	F-actin-capping protein subunit alpha-2 OS=Homo sapiens OX=9606 GN=CAPZA2 PE=1 SV=3	CAZA2_HUMAN	CAPZA2	33 kDa	6	3	4	2	4	6	4	6	4	3	4	7
459	Dynactin subunit 4 OS=Homo sapiens OX=9606 GN=DCTN4 PE=1 SV=1	DCTN4_HUMAN	DCTN4	52 kDa	0	6	2	4	5	6	2	4	4	4	0	4
460	Zinc finger ZZ-type and EF-hand domain-containing protein 1 OS=Homo sapiens OX=9606 GN=ZZEF1 PE=1 SV=6	ZZEF1_HUMAN	ZZEF1	331 kDa	0	7	4	3	6	7	0	4	2	0	2	2
461	Phosphoribosyl pyrophosphate synthase-associated protein 1 OS=Homo sapiens OX=9606 GN=PRPSAP1 PE=1 SV=2	KPRA_HUMAN	PRPSAP1	39 kDa	8	10	9	9	10	10	9	10	10	11	11	10
462	Histone-arginine methyltransferase CARM1 OS=Homo sapiens OX=9606 GN=CARM1 PE=1 SV=3	CARM1_HUMAN	CARM1	66 kDa	0	6	0	0	5	4	0	4	0	0	6	3
463	Catechol O-methyltransferase OS=Homo sapiens OX=9606 GN=COMT PE=1 SV=2	COMT_HUMAN	COMT	30 kDa	0	0	4	0	0	2	5	5	9	5	6	7
464	MICAL-like protein 2 OS=Homo sapiens OX=9606 GN=MICALL2 PE=1 SV=1	MILK2_HUMAN	MICALL2	98 kDa	0	2	3	0	3	5	3	4	4	6	6	4
465	Ras-related protein Rab-8B OS=Homo sapiens OX=9606 GN=RAB8B PE=1 SV=2	RAB8B_HUMAN	RAB8B	24 kDa	17	16	14	23	20	18	19	25	18	18	21	15
466	Charged multivesicular body protein 6 OS=Homo sapiens OX=9606 GN=CHMP6 PE=1 SV=3	CHMP6_HUMAN	CHMP6	23 kDa	5	3	5	5	0	5	4	6	4	6	4	8
467	E3 ubiquitin-protein ligase HECTD3 OS=Homo sapiens OX=9606 GN=HECTD3 PE=1 SV=1	HECD3_HUMAN	HECTD3	97 kDa	4	6	8	6	6	11	2	3	0	2	6	4
468	Epidermal growth factor receptor substrate 15 OS=Homo sapiens OX=9606 GN=EPS15 PE=1 SV=2	EPS15_HUMAN	EPS15	99 kDa	0	6	5	0	4	2	4	7	0	0	4	6
469	Fermitin family homolog 3 OS=Homo sapiens OX=9606 GN=FERMT3 PE=1 SV=1	URP2_HUMAN	FERMT3	76 kDa	3	3	5	2	7	5	0	2	0	4	0	2
470	Carbonic anhydrase 2 OS=Homo sapiens OX=9606 GN=CA2 PE=1 SV=2	CAH2_HUMAN	CA2	29 kDa	2	5	4	0	0	4	6	5	6	3	2	2
471	Golgin subfamily A member 7 OS=Homo sapiens OX=9606 GN=GOLGA7 PE=1 SV=2	GOGA7_HUMAN	GOLGA7	16 kDa	5	5	5	6	7	4	5	5	5	5	3	5
472	UTP--glucose-1-phosphate uridylyltransferase OS=Homo sapiens OX=9606 GN=UGP2 PE=1 SV=5	UGPA_HUMAN	UGP2	57 kDa	5	5	6	0	4	4	7	8	4	3	5	2
473	Cytochrome b5 type B OS=Homo sapiens OX=9606 GN=CYB5B PE=1 SV=3	CYB5B_HUMAN	CYB5B	17 kDa	5	0	3	5	3	0	6	5	7	6	3	7
474	Glutathione peroxidase 1 OS=Homo sapiens OX=9606 GN=GPX1 PE=1 SV=4	GPX1_HUMAN	GPX1	22 kDa	3	5	8	4	7	3	0	0	0	2	2	3
475	Dehydrogenase/reductase SDR family member 7B OS=Homo sapiens OX=9606 GN=DHRS7B PE=1 SV=2	DRS7B_HUMAN	DHRS7B	35 kDa	3	3	4	4	5	3	6	6	8	7	8	5
476	Syntaxin-2 OS=Homo sapiens OX=9606 GN=STX2 PE=1 SV=3	STX2_HUMAN	STX2	33 kDa	2	0	0	0	2	0	8	6	5	6	7	4
477	Ubiquitin carboxyl-terminal hydrolase isozyme L5 OS=Homo sapiens OX=9606 GN=UCHL5 PE=1 SV=3	UCHL5_HUMAN	UCHL5	38 kDa	3	3	0	0	3	0	4	7	3	6	8	4
478	Disco-interacting protein 2 homolog B OS=Homo sapiens OX=9606 GN=DIP2B PE=1 SV=3	DIP2B_HUMAN	DIP2B	171 kDa	6	4	2	0	3	0	4	3	0	4	2	0
479	Small VCP/p97-interacting protein OS=Homo sapiens OX=9606 GN=SVIP PE=1 SV=1	SVIP_HUMAN	SVIP	8 kDa	5	5	5	6	5	3	6	6	5	6	5	6
480	Thioredoxin-like protein 1 OS=Homo sapiens OX=9606 GN=TXNL1 PE=1 SV=3	TXNL1_HUMAN	TXNL1	32 kDa	0	6	4	0	4	4	7	3	4	4	4	0
481	Ubiquitin-like modifier-activating enzyme 1 OS=Homo sapiens OX=9606 GN=UBA1 PE=1 SV=3	UBA1_HUMAN	UBA1	118 kDa	4	3	3	3	7	7	6	4	0	3	3	3
482	Transmembrane protein 14C OS=Homo sapiens OX=9606 GN=TMEM14C PE=1 SV=1	TM14C_HUMAN	TMEM14C	12 kDa	0	4	0	4	0	3	5	4	5	4	5	4
483	SUMO-conjugating enzyme UBC9 OS=Homo sapiens OX=9606 GN=UBE2I PE=1 SV=1	UBC9_HUMAN	UBE2I	18 kDa	4	3	3	5	0	4	0	3	4	4	3	2
484	Guanine nucleotide-binding protein G(I)/G(S)/G(O) subunit gamma-5 OS=Homo sapiens OX=9606 GN=GNG5 PE=1 SV=3	GBG5_HUMAN	GNG5	7 kDa	4	3	3	4	5	5	5	5	5	2	3	5
485	ER lumen protein-retaining receptor 1 OS=Homo sapiens OX=9606 GN=KDELR1 PE=1 SV=1	ERD21_HUMAN	KDELR1	25 kDa	6	5	6	6	8	6	7	0	0	4	4	6
486	Sorting nexin-2 OS=Homo sapiens OX=9606 GN=SNX2 PE=1 SV=2	SNX2_HUMAN	SNX2	58 kDa	0	5	3	4	3	5	2	4	5	7	6	5
487	Clathrin light chain B OS=Homo sapiens OX=9606 GN=CLTB PE=1 SV=1	CLCB_HUMAN	CLTB	25 kDa	0	5	0	2	4	2	0	3	3	3	3	2
488	Phospholipid scramblase 4 OS=Homo sapiens OX=9606 GN=PLSCR4 PE=1 SV=2	PLS4_HUMAN	PLSCR4	37 kDa	6	3	5	3	4	4	5	5	9	7	4	5
489	ADP-ribosylation factor 1 OS=Homo sapiens OX=9606 GN=ARF1 PE=1 SV=2	ARF1_HUMAN	ARF1	21 kDa	3	3	5	6	5	6	3	4	3	4	5	5
490	V-type proton ATPase subunit d 1 OS=Homo sapiens OX=9606 GN=ATP6V0D1 PE=1 SV=1	VA0D1_HUMAN	ATP6V0D1	40 kDa	0	3	6	2	3	3	5	5	6	7	6	6
491	Rab3 GTPase-activating protein catalytic subunit OS=Homo sapiens OX=9606 GN=RAB3GAP1 PE=1 SV=3	RB3GP_HUMAN	RAB3GAP1	111 kDa	4	6	7	6	4	5	0	2	0	4	2	3
492	Glycerophosphodiester phosphodiesterase 1 OS=Homo sapiens OX=9606 GN=GDE1 PE=1 SV=1	GDE1_HUMAN	GDE1	38 kDa	0	5	5	5	3	4	3	4	4	4	4	3
493	PRA1 family protein 3 OS=Homo sapiens OX=9606 GN=ARL6IP5 PE=1 SV=1	PRAF3_HUMAN	ARL6IP5	22 kDa	0	5	5	5	4	4	4	4	4	4	0	4
494	ADP-ribosyl cyclase/cyclic ADP-ribose hydrolase 1 OS=Homo sapiens OX=9606 GN=CD38 PE=1 SV=2	CD38_HUMAN	CD38	34 kDa	0	2	0	0	0	2	0	0	3	4	3	3
495	V-type proton ATPase subunit H OS=Homo sapiens OX=9606 GN=ATP6V1H PE=1 SV=1	VATH_HUMAN	ATP6V1H	56 kDa	4	0	3	0	7	2	3	5	0	0	0	0
496	Serotransferrin OS=Homo sapiens OX=9606 GN=TF PE=1 SV=3	TRFE_HUMAN	TF	77 kDa	9	0	5	4	4	4	7	3	3	0	3	3
497	Selenocysteine-specific elongation factor OS=Homo sapiens OX=9606 GN=EEFSEC PE=1 SV=4	SELB_HUMAN	EEFSEC	65 kDa	11	2	7	8	8	7	0	3	0	0	2	2
498	UV excision repair protein RAD23 homolog A OS=Homo sapiens OX=9606 GN=RAD23A PE=1 SV=1	RD23A_HUMAN	RAD23A	40 kDa	3	3	3	0	6	0	7	6	3	0	2	2
499	Cytochrome b reductase 1 OS=Homo sapiens OX=9606 GN=CYBRD1 PE=1 SV=1	CYBR1_HUMAN	CYBRD1	32 kDa	4	0	0	0	0	0	6	0	0	0	4	0
500	Rab GDP dissociation inhibitor beta OS=Homo sapiens OX=9606 GN=GDI2 PE=1 SV=2	GDIB_HUMAN	GDI2	51 kDa	2	4	4	3	3	4	3	3	4	2	2	3
501	GTPase HRas OS=Homo sapiens OX=9606 GN=HRAS PE=1 SV=1	RASH_HUMAN	HRAS	21 kDa	28	26	31	28	30	23	0	30	32	30	26	32
502	AMP deaminase 3 OS=Homo sapiens OX=9606 GN=AMPD3 PE=1 SV=1	AMPD3_HUMAN	AMPD3	89 kDa	3	3	2	5	9	7	0	0	3	4	2	4
503	Nucleobindin-2 OS=Homo sapiens OX=9606 GN=NUCB2 PE=1 SV=3	NUCB2_HUMAN	NUCB2	50 kDa	6	4	5	3	2	3	3	0	3	2	0	0
504	Importin subunit alpha-7 OS=Homo sapiens OX=9606 GN=KPNA6 PE=1 SV=1	IMA7_HUMAN	KPNA6	60 kDa	3	3	3	2	3	2	2	6	2	3	4	6
505	Aspartyl aminopeptidase OS=Homo sapiens OX=9606 GN=DNPEP PE=1 SV=1	DNPEP_HUMAN	DNPEP	52 kDa	2	3	3	4	8	7	3	5	3	0	2	2
506	Phosphatidylserine lipase ABHD16A OS=Homo sapiens OX=9606 GN=ABHD16A PE=1 SV=3	ABHGA_HUMAN	ABHD16A	63 kDa	0	2	5	2	3	3	7	6	4	3	4	4
507	Clusterin OS=Homo sapiens OX=9606 GN=CLU PE=1 SV=1	CLUS_HUMAN	CLU	52 kDa	0	0	4	0	4	4	6	4	4	5	4	5
508	Ras-related protein Rap-2a OS=Homo sapiens OX=9606 GN=RAP2A PE=1 SV=1	RAP2A_HUMAN	RAP2A	21 kDa	30	26	25	26	28	26	26	32	27	25	27	28
509	Phospholipase D1 OS=Homo sapiens OX=9606 GN=PLD1 PE=1 SV=1	PLD1_HUMAN	PLD1	124 kDa	0	3	3	7	3	6	2	3	7	3	3	4
510	Rab3 GTPase-activating protein non-catalytic subunit OS=Homo sapiens OX=9606 GN=RAB3GAP2 PE=1 SV=1	RBGPR_HUMAN	RAB3GAP2	156 kDa	3	0	3	2	5	8	4	3	3	2	2	3
511	Dolichyl-diphosphooligosaccharide--protein glycosyltransferase subunit 1 OS=Homo sapiens OX=9606 GN=RPN1 PE=1 SV=1	RPN1_HUMAN	RPN1	69 kDa	4	3	8	5	4	4	5	3	0	3	3	6
512	Pyridoxal phosphate homeostasis protein OS=Homo sapiens OX=9606 GN=PLPBP PE=1 SV=1	PLPHP_HUMAN	PLPBP	30 kDa	0	3	5	4	5	6	0	2	8	5	2	9
513	Ras-related protein Rab-5A OS=Homo sapiens OX=9606 GN=RAB5A PE=1 SV=2	RAB5A_HUMAN	RAB5A	24 kDa	13	10	13	12	10	11	12	13	8	12	10	11
514	Very long-chain acyl-CoA synthetase OS=Homo sapiens OX=9606 GN=SLC27A2 PE=1 SV=2	S27A2_HUMAN	SLC27A2	70 kDa	3	3	5	0	2	2	5	4	5	5	6	4
515	Adenylyl cyclase-associated protein 1 OS=Homo sapiens OX=9606 GN=CAP1 PE=1 SV=5	CAP1_HUMAN	CAP1	52 kDa	0	5	0	0	2	6	2	4	0	2	0	2
516	Septin-2 OS=Homo sapiens OX=9606 GN=SEPT2 PE=1 SV=1	SEPT2_HUMAN	44076	41 kDa	2	2	6	5	6	5	5	3	3	3	6	4
517	BAG family molecular chaperone regulator 2 OS=Homo sapiens OX=9606 GN=BAG2 PE=1 SV=1	BAG2_HUMAN	BAG2	24 kDa	0	0	2	0	0	0	2	4	6	7	6	7
518	Casein kinase II subunit alpha OS=Homo sapiens OX=9606 GN=CSNK2A1 PE=1 SV=1	CSK21_HUMAN	CSNK2A1	45 kDa	0	0	0	0	2	0	0	0	5	4	0	0
519	Sorting nexin-22 OS=Homo sapiens OX=9606 GN=SNX22 PE=1 SV=1	SNX22_HUMAN	SNX22	22 kDa	0	3	0	0	3	2	0	0	0	0	4	0
520	Hepatocyte growth factor-regulated tyrosine kinase substrate OS=Homo sapiens OX=9606 GN=HGS PE=1 SV=1	HGS_HUMAN	HGS	86 kDa	5	3	5	0	2	0	3	6	5	2	4	3
521	Ras-related protein Rab-1B OS=Homo sapiens OX=9606 GN=RAB1B PE=1 SV=1	RAB1B_HUMAN	RAB1B	22 kDa	0	0	0	0	0	0	0	0	0	0	17	10
522	Importin-9 OS=Homo sapiens OX=9606 GN=IPO9 PE=1 SV=3	IPO9_HUMAN	IPO9	116 kDa	2	3	5	3	4	4	0	5	4	0	2	0
523	Serine/threonine-protein phosphatase 2A catalytic subunit beta isoform OS=Homo sapiens OX=9606 GN=PPP2CB PE=1 SV=1	PP2AB_HUMAN	PPP2CB	36 kDa	0	0	0	0	5	6	3	4	0	0	3	4
524	Tubulin alpha-1B chain OS=Homo sapiens OX=9606 GN=TUBA1B PE=1 SV=1	TBA1B_HUMAN	TUBA1B	50 kDa	0	2	6	6	7	7	0	0	0	0	0	0
525	Retinol dehydrogenase 11 OS=Homo sapiens OX=9606 GN=RDH11 PE=1 SV=2	RDH11_HUMAN	RDH11	35 kDa	2	5	4	5	3	4	3	3	4	5	3	4
526	O-phosphoseryl-tRNA(Sec) selenium transferase OS=Homo sapiens OX=9606 GN=SEPSECS PE=1 SV=2	SPCS_HUMAN	SEPSECS	56 kDa	0	8	5	6	6	4	0	2	2	3	4	7
527	Eukaryotic translation initiation factor 5A-1 OS=Homo sapiens OX=9606 GN=EIF5A PE=1 SV=2	IF5A1_HUMAN	EIF5A	17 kDa	0	0	4	0	3	5	0	5	6	7	2	0
528	CAD protein OS=Homo sapiens OX=9606 GN=CAD PE=1 SV=3	PYR1_HUMAN	CAD	243 kDa	0	2	2	0	5	4	0	2	0	0	0	4
529	Leucine-rich repeat-containing protein 59 OS=Homo sapiens OX=9606 GN=LRRC59 PE=1 SV=1	LRC59_HUMAN	LRRC59	35 kDa	6	2	4	4	2	2	5	2	0	0	2	0
530	Gamma-aminobutyric acid receptor-associated protein-like 1 OS=Homo sapiens OX=9606 GN=GABARAPL1 PE=1 SV=1	GBRL1_HUMAN	GABARAPL1	14 kDa	4	7	6	4	4	6	0	4	2	4	0	3
531	GTP-binding protein 1 OS=Homo sapiens OX=9606 GN=GTPBP1 PE=1 SV=3	GTPB1_HUMAN	GTPBP1	72 kDa	0	3	3	5	4	7	2	0	0	2	2	5
532	Protein kish-A OS=Homo sapiens OX=9606 GN=TMEM167A PE=1 SV=1	KISHA_HUMAN	TMEM167A	8 kDa	0	6	0	0	0	0	5	4	0	0	0	0
533	Cathepsin E OS=Homo sapiens OX=9606 GN=CTSE PE=1 SV=3	CATE_HUMAN	CTSE	43 kDa	0	0	0	0	3	0	5	6	5	2	3	2
534	DnaJ homolog subfamily C member 5 OS=Homo sapiens OX=9606 GN=DNAJC5 PE=1 SV=1	DNJC5_HUMAN	DNAJC5	22 kDa	0	0	0	0	0	0	6	3	2	0	3	3
535	Sarcoplasmic/endoplasmic reticulum calcium ATPase 2 OS=Homo sapiens OX=9606 GN=ATP2A2 PE=1 SV=1	AT2A2_HUMAN	ATP2A2	115 kDa	0	0	3	0	0	0	0	2	5	6	5	7
536	Elongation factor 2 OS=Homo sapiens OX=9606 GN=EEF2 PE=1 SV=4	EF2_HUMAN	EEF2	95 kDa	0	0	2	6	8	6	0	0	0	0	2	3
537	Signal transducing adapter molecule 1 OS=Homo sapiens OX=9606 GN=STAM PE=1 SV=3	STAM1_HUMAN	STAM	59 kDa	4	3	0	0	0	2	6	4	3	5	3	3
538	Dynactin subunit 3 OS=Homo sapiens OX=9606 GN=DCTN3 PE=1 SV=1	DCTN3_HUMAN	DCTN3	21 kDa	0	0	0	0	0	4	0	0	2	2	3	0
539	Nicotinamide/nicotinic acid mononucleotide adenylyltransferase 3 OS=Homo sapiens OX=9606 GN=NMNAT3 PE=1 SV=2	NMNA3_HUMAN	NMNAT3	28 kDa	0	0	0	0	8	4	0	0	0	3	5	2
540	Ferritin heavy chain OS=Homo sapiens OX=9606 GN=FTH1 PE=1 SV=2	FRIH_HUMAN	FTH1	21 kDa	6	7	6	7	5	3	0	0	0	0	0	0
541	Presenilin-1 OS=Homo sapiens OX=9606 GN=PSEN1 PE=1 SV=1	PSN1_HUMAN	PSEN1	53 kDa	2	3	0	4	0	3	7	5	3	7	3	3
542	Ribonuclease inhibitor OS=Homo sapiens OX=9606 GN=RNH1 PE=1 SV=2	RINI_HUMAN	RNH1	50 kDa	0	2	3	0	5	8	4	2	3	0	3	4
543	IST1 homolog OS=Homo sapiens OX=9606 GN=IST1 PE=1 SV=1	IST1_HUMAN	IST1	40 kDa	0	2	3	0	3	3	0	4	3	3	5	3
544	Serine/threonine-protein phosphatase 5 OS=Homo sapiens OX=9606 GN=PPP5C PE=1 SV=1	PPP5_HUMAN	PPP5C	57 kDa	2	6	5	6	5	4	0	3	4	3	3	2
545	Oxysterol-binding protein 2 OS=Homo sapiens OX=9606 GN=OSBP2 PE=1 SV=2	OSBP2_HUMAN	OSBP2	101 kDa	0	0	3	4	3	4	2	4	0	3	4	5
546	Protein S100-A4 OS=Homo sapiens OX=9606 GN=S100A4 PE=1 SV=1	S10A4_HUMAN	S100A4	12 kDa	0	0	0	0	4	0	0	0	3	2	0	0
547	Septin-7 OS=Homo sapiens OX=9606 GN=SEPT7 PE=1 SV=2	SEPT7_HUMAN	44081	51 kDa	0	4	2	3	4	4	4	5	3	2	3	4
548	Acidic leucine-rich nuclear phosphoprotein 32 family member A OS=Homo sapiens OX=9606 GN=ANP32A PE=1 SV=1	AN32A_HUMAN	ANP32A	29 kDa	2	0	0	0	0	0	0	2	3	5	4	5

549	Hemoglobin subunit mu OS=Homo sapiens OX=9606 GN=HBM PE=1 SV=1	HBM_HUMAN	HBM	16 kDa	0	0	7	5	9	10	0	0	0	0	0	0
550	Sodium/potassium-transporting ATPase subunit beta-3 OS=Homo sapiens OX=9606 GN=ATP1B3 PE=1 SV=1	AT1B3_HUMAN	ATP1B3	32 kDa	4	5	2	3	6	0	4	0	0	2	0	0
551	Porphobilinogen deaminase OS=Homo sapiens OX=9606 GN=HMBS PE=1 SV=2	HEM3_HUMAN	HMBS	39 kDa	0	2	2	2	2	4	0	0	2	0	4	5
552	Apolipoprotein A-I OS=Homo sapiens OX=9606 GN=APOA1 PE=1 SV=1	APOA1_HUMAN	APOA1	31 kDa	3	4	2	4	2	4	3	4	3	3	3	2
553	CD2-associated protein OS=Homo sapiens OX=9606 GN=CD2AP PE=1 SV=1	CD2AP_HUMAN	CD2AP	71 kDa	2	4	0	5	4	0	0	3	0	0	2	2
554	CD99 antigen OS=Homo sapiens OX=9606 GN=CD99 PE=1 SV=1	CD99_HUMAN	CD99	19 kDa	4	4	3	0	5	3	4	4	3	4	4	4
555	Choline transporter-like protein 1 OS=Homo sapiens OX=9606 GN=SLC44A1 PE=1 SV=1	CTL1_HUMAN	SLC44A1	73 kDa	2	6	0	2	2	0	3	4	3	4	2	0
556	Glucose-6-phosphate exchanger SLC37A4 OS=Homo sapiens OX=9606 GN=SLC37A4 PE=1 SV=1	G6PT1_HUMAN	SLC37A4	46 kDa	0	3	3	0	0	0	4	2	6	4	5	2
557	PITH domain-containing protein 1 OS=Homo sapiens OX=9606 GN=PITHD1 PE=1 SV=1	PITH1_HUMAN	PITHD1	24 kDa	3	0	2	3	4	3	0	2	0	0	0	0
558	Zinc finger CCCH-type antiviral protein 1 OS=Homo sapiens OX=9606 GN=ZC3HAV1 PE=1 SV=3	ZCCHV_HUMAN	ZC3HAV1	101 kDa	3	0	3	5	5	4	3	0	2	0	2	0
559	Tubulin beta-4B chain OS=Homo sapiens OX=9606 GN=TUBB4B PE=1 SV=1	TBB4B_HUMAN	TUBB4B	50 kDa	2	0	6	7	4	13	0	0	0	0	0	0
560	Eukaryotic translation initiation factor 4 gamma 1 OS=Homo sapiens OX=9606 GN=EIF4G1 PE=1 SV=4	IF4G1_HUMAN	EIF4G1	175 kDa	0	2	3	3	6	3	0	4	0	2	3	2
561	Peroxiredoxin-4 OS=Homo sapiens OX=9606 GN=PRDX4 PE=1 SV=1	PRDX4_HUMAN	PRDX4	31 kDa	0	3	5	0	0	0	6	0	6	4	4	3
562	Serpin H1 OS=Homo sapiens OX=9606 GN=SERPINH1 PE=1 SV=2	SERPH_HUMAN	SERPINH1	46 kDa	3	3	3	4	3	3	4	2	2	4	3	4
563	Mannosyl-oligosaccharide glucosidase OS=Homo sapiens OX=9606 GN=MOGS PE=1 SV=5	MOGS_HUMAN	MOGS	92 kDa	2	0	0	0	2	0	3	4	0	4	2	5
564	Importin subunit alpha-3 OS=Homo sapiens OX=9606 GN=KPNA4 PE=1 SV=1	IMA3_HUMAN	KPNA4	58 kDa	0	3	3	5	5	5	3	5	3	0	4	3
565	Serine/threonine-protein kinase STK11 OS=Homo sapiens OX=9606 GN=STK11 PE=1 SV=1	STK11_HUMAN	STK11	49 kDa	0	5	0	0	0	0	7	4	3	4	6	4
566	MAGUK p55 subfamily member 7 OS=Homo sapiens OX=9606 GN=MPP7 PE=1 SV=1	MPP7_HUMAN	MPP7	66 kDa	2	0	0	0	0	0	6	5	2	3	0	2
567	Protein RER1 OS=Homo sapiens OX=9606 GN=RER1 PE=1 SV=1	RER1_HUMAN	RER1	23 kDa	3	4	4	2	3	0	0	0	2	4	3	2
568	Peroxiredoxin-6 OS=Homo sapiens OX=9606 GN=PRDX6 PE=1 SV=3	PRDX6_HUMAN	PRDX6	25 kDa	0	0	0	0	0	0	2	4	4	3	2	0
569	TSC22 domain family protein 4 OS=Homo sapiens OX=9606 GN=TSC22D4 PE=1 SV=2	T22D4_HUMAN	TSC22D4	41 kDa	0	8	0	4	6	3	0	5	0	3	4	0
570	Vacuolar protein-sorting-associated protein 25 OS=Homo sapiens OX=9606 GN=VPS25 PE=1 SV=1	VPS25_HUMAN	VPS25	21 kDa	0	3	0	0	0	2	0	4	3	3	3	3
571	1-acyl-sn-glycerol-3-phosphate acyltransferase alpha OS=Homo sapiens OX=9606 GN=AGPAT1 PE=1 SV=2	PLCA_HUMAN	AGPAT1	32 kDa	3	0	0	0	2	0	0	0	0	0	0	0
572	Endoplasmic reticulum-Golgi intermediate compartment protein 2 OS=Homo sapiens OX=9606 GN=ERGIC2 PE=1 SV=2	ERGI2_HUMAN	ERGIC2	43 kDa	5	0	3	2	0	4	0	3	2	3	0	0
573	Dolichyl-diphosphooligosaccharide--protein glycosyltransferase subunit 2 OS=Homo sapiens OX=9606 GN=RPN2 PE=1 SV=3	RPN2_HUMAN	RPN2	69 kDa	0	0	0	3	0	0	2	4	5	5	3	5
574	Neuroplastin OS=Homo sapiens OX=9606 GN=NPTN PE=1 SV=2	NPTN_HUMAN	NPTN	44 kDa	0	0	0	0	0	0	6	0	3	0	0	4
575	EH domain-containing protein 1 OS=Homo sapiens OX=9606 GN=EHD1 PE=1 SV=2	EHD1_HUMAN	EHD1	61 kDa	0	2	4	2	3	0	0	0	0	2	3	2
576	Protein-L-isoaspartate(D-aspartate) O-methyltransferase OS=Homo sapiens OX=9606 GN=PCMT1 PE=1 SV=4	PIMT_HUMAN	PCMT1	25 kDa	0	3	4	4	3	3	3	3	2	5	2	4
577	Transcription elongation factor A protein 1 OS=Homo sapiens OX=9606 GN=TCEA1 PE=1 SV=2	TCEA1_HUMAN	TCEA1	34 kDa	0	0	2	2	3	0	0	4	5	7	4	4
578	Y-box-binding protein 3 OS=Homo sapiens OX=9606 GN=YBX3 PE=1 SV=4	YBOX3_HUMAN	YBX3	40 kDa	0	0	0	2	4	0	4	3	0	0	4	2
579	Lysophospholipid acyltransferase 5 OS=Homo sapiens OX=9606 GN=LPCAT3 PE=1 SV=1	MBOA5_HUMAN	LPCAT3	56 kDa	0	2	2	0	0	0	2	3	0	5	0	3
580	Phosphatidylinositol 3,4,5-trisphosphate 5-phosphatase 1 OS=Homo sapiens OX=9606 GN=INPP5D PE=1 SV=2	SHIP1_HUMAN	INPP5D	133 kDa	4	2	0	2	4	2	4	0	0	2	3	5
581	Charged multivesicular body protein 1b OS=Homo sapiens OX=9606 GN=CHMP1B PE=1 SV=1	CHM1B_HUMAN	CHMP1B	22 kDa	0	2	0	4	2	0	3	3	4	0	3	2
582	Endoplasmic reticulum junction formation protein lunapark OS=Homo sapiens OX=9606 GN=LNPK PE=1 SV=2	LNP_HUMAN	LNPK	48 kDa	0	0	4	0	4	0	4	3	3	0	3	2
583	C-C motif chemokine 14 OS=Homo sapiens OX=9606 GN=CCL14 PE=1 SV=1	CCL14_HUMAN	CCL14	11 kDa	5	0	0	5	0	2	0	4	0	0	0	0
584	Cysteine-rich and transmembrane domain-containing protein 1 OS=Homo sapiens OX=9606 GN=CYSTM1 PE=1 SV=1	CYTM1_HUMAN	CYSTM1	11 kDa	0	0	0	0	0	0	4	4	0	0	0	0
585	Kinesin-1 heavy chain OS=Homo sapiens OX=9606 GN=KIF5B PE=1 SV=1	KINH_HUMAN	KIF5B	110 kDa	0	0	0	0	4	0	0	0	0	0	0	0
586	Heterogeneous nuclear ribonucleoprotein Q OS=Homo sapiens OX=9606 GN=SYNCRIP PE=1 SV=2	HNRPQ_HUMAN	SYNCRIP	70 kDa	2	4	4	0	5	3	0	4	0	0	3	3
587	60S acidic ribosomal protein P2 OS=Homo sapiens OX=9606 GN=RPLP2 PE=1 SV=1	RLA2_HUMAN	RPLP2	12 kDa	0	5	5	4	6	3	0	0	2	3	2	5
588	Gephyrin OS=Homo sapiens OX=9606 GN=GPHN PE=1 SV=1	GEPH_HUMAN	GPHN	80 kDa	0	0	2	4	5	0	0	3	3	0	4	3
589	DnaJ homolog subfamily B member 2 OS=Homo sapiens OX=9606 GN=DNAJB2 PE=1 SV=3	DNJB2_HUMAN	DNAJB2	36 kDa	0	0	0	0	2	6	3	0	0	2	3	9
590	Ran-binding protein 10 OS=Homo sapiens OX=9606 GN=RANBP10 PE=1 SV=1	RBP10_HUMAN	RANBP10	67 kDa	0	4	0	3	0	0	0	3	2	2	0	3
591	Bleomycin hydrolase OS=Homo sapiens OX=9606 GN=BLMH PE=1 SV=1	BLMH_HUMAN	BLMH	53 kDa	0	3	3	0	2	0	4	2	0	0	3	0
592	Major facilitator superfamily domain-containing protein 2B OS=Homo sapiens OX=9606 GN=MFSD2B PE=1 SV=4	MFS2B_HUMAN	MFSD2B	54 kDa	0	0	0	0	0	3	3	5	3	5	0	0
593	Protein disulfide-isomerase A4 OS=Homo sapiens OX=9606 GN=PDIA4 PE=1 SV=2	PDIA4_HUMAN	PDIA4	73 kDa	0	2	0	0	0	0	5	0	3	0	2	2
594	Copper-transporting ATPase 2 OS=Homo sapiens OX=9606 GN=ATP7B PE=1 SV=4	ATP7B_HUMAN	ATP7B	157 kDa	0	2	2	0	2	0	0	0	0	3	6	4
595	Transmembrane 9 superfamily member 2 OS=Homo sapiens OX=9606 GN=TM9SF2 PE=1 SV=1	TM9S2_HUMAN	TM9SF2	76 kDa	3	0	0	0	3	2	0	2	2	3	0	0
596	High affinity cationic amino acid transporter 1 OS=Homo sapiens OX=9606 GN=SLC7A1 PE=1 SV=1	CTR1_HUMAN	SLC7A1	68 kDa	0	0	0	0	2	3	3	2	4	2	2	3
597	Proteolipid protein 2 OS=Homo sapiens OX=9606 GN=PLP2 PE=1 SV=1	PLP2_HUMAN	PLP2	17 kDa	3	3	4	4	3	4	3	4	2	0	5	3
598	Proteasome assembly chaperone 1 OS=Homo sapiens OX=9606 GN=PSMG1 PE=1 SV=1	PSMG1_HUMAN	PSMG1	33 kDa	0	4	2	0	4	2	0	0	0	0	0	3
599	NIF3-like protein 1 OS=Homo sapiens OX=9606 GN=NIF3L1 PE=1 SV=2	NIF3L_HUMAN	NIF3L1	42 kDa	0	0	0	0	3	0	3	3	2	0	0	0
600	Transmembrane emp24 domain-containing protein 1 OS=Homo sapiens OX=9606 GN=TMED1 PE=1 SV=1	TMED1_HUMAN	TMED1	25 kDa	4	2	4	2	3	3	4	2	0	0	0	0
601	Beta-2-microglobulin OS=Homo sapiens OX=9606 GN=B2M PE=1 SV=1	B2MG_HUMAN	B2M	14 kDa	0	0	0	0	0	0	6	4	4	4	5	8
602	DNA-dependent protein kinase catalytic subunit OS=Homo sapiens OX=9606 GN=PRKDC PE=1 SV=3	PRKDC_HUMAN	PRKDC	469 kDa	0	0	5	3	6	4	0	0	0	0	0	0
603	Ubiquitin-fold modifier 1 OS=Homo sapiens OX=9606 GN=UFM1 PE=1 SV=1	UFM1_HUMAN	UFM1	9 kDa	2	0	0	0	3	3	3	2	0	3	3	2
604	Beta-arrestin-2 OS=Homo sapiens OX=9606 GN=ARRB2 PE=1 SV=2	ARRB2_HUMAN	ARRB2	46 kDa	0	0	2	0	0	0	0	0	3	0	0	3
605	Proteasome inhibitor PI31 subunit OS=Homo sapiens OX=9606 GN=PSMF1 PE=1 SV=2	PSMF1_HUMAN	PSMF1	30 kDa	0	2	0	0	2	2	0	3	0	0	0	0
606	Synaptogyrin-2 OS=Homo sapiens OX=9606 GN=SYNGR2 PE=1 SV=1	SNG2_HUMAN	SYNGR2	25 kDa	0	0	0	0	2	3	0	0	0	0	0	0
607	60S ribosomal protein L18 OS=Homo sapiens OX=9606 GN=RPL18 PE=1 SV=2	RL18_HUMAN	RPL18	22 kDa	0	3	2	0	3	3	0	0	0	0	0	0
608	Long-chain-fatty-acid--CoA ligase 5 OS=Homo sapiens OX=9606 GN=ACSL5 PE=1 SV=1	ACSL5_HUMAN	ACSL5	76 kDa	0	5	6	0	6	6	0	3	5	4	0	6
609	C-C chemokine receptor-like 2 OS=Homo sapiens OX=9606 GN=CCRL2 PE=1 SV=2	CCRL2_HUMAN	CCRL2	40 kDa	0	0	0	0	0	0	0	2	0	4	0	0
610	Ras-related protein Rab-2A OS=Homo sapiens OX=9606 GN=RAB2A PE=1 SV=1	RAB2A_HUMAN	RAB2A	24 kDa	0	8	8	10	0	12	0	0	0	0	0	0
611	Serine--tRNA ligase, cytoplasmic OS=Homo sapiens OX=9606 GN=SARS PE=1 SV=3	SYSC_HUMAN	SARS	59 kDa	0	0	3	4	10	2	0	0	0	0	0	0
612	Epidermal growth factor receptor substrate 15-like 1 OS=Homo sapiens OX=9606 GN=EPS15L1 PE=1 SV=1	EP15R_HUMAN	EPS15L1	94 kDa	0	0	2	2	0	2	0	2	3	0	4	2
613	CDP-diacylglycerol--inositol 3-phosphatidyltransferase OS=Homo sapiens OX=9606 GN=CDIPT PE=1 SV=1	CDIPT_HUMAN	CDIPT	24 kDa	3	0	0	2	0	0	0	4	4	3	3	4
614	Synaptophysin-like protein 1 OS=Homo sapiens OX=9606 GN=SYPL1 PE=1 SV=1	SYPL1_HUMAN	SYPL1	29 kDa	2	0	3	0	3	4	0	0	0	0	2	0
615	Myeloid-associated differentiation marker OS=Homo sapiens OX=9606 GN=MYADM PE=1 SV=2	MYADM_HUMAN	MYADM	35 kDa	0	0	0	0	0	0	3	0	0	0	4	0
616	Immunoglobulin kappa variable 3-20 OS=Homo sapiens OX=9606 GN=IGKV3-20 PE=1 SV=2	KV320_HUMAN	IGKV3-20	13 kDa	0	0	0	0	3	3	0	4	3	0	0	4
617	Glucose-induced degradation protein 8 homolog OS=Homo sapiens OX=9606 GN=GID8 PE=1 SV=1	GID8_HUMAN	GID8	27 kDa	0	2	3	0	4	5	3	0	0	0	4	0
618	Oxysterol-binding protein-related protein 8 OS=Homo sapiens OX=9606 GN=OSBPL8 PE=1 SV=3	OSBL8_HUMAN	OSBPL8	101 kDa	0	0	0	0	0	0	7	5	5	3	3	2
619	Unconventional myosin-Ic OS=Homo sapiens OX=9606 GN=MYO1C PE=1 SV=4	MYO1C_HUMAN	MYO1C	122 kDa	0	0	0	0	0	2	2	0	0	5	0	2
620	Palmitoyltransferase ZDHHC20 OS=Homo sapiens OX=9606 GN=ZDHHC20 PE=1 SV=1	ZDH20_HUMAN	ZDHHC20	42 kDa	0	0	0	3	0	0	0	0	4	0	0	0
621	Alpha-synuclein OS=Homo sapiens OX=9606 GN=SNCA PE=1 SV=1	SYUA_HUMAN	SNCA	14 kDa	2	3	2	0	3	0	3	3	4	4	0	2
622	Endoplasmic reticulum aminopeptidase 1 OS=Homo sapiens OX=9606 GN=ERAP1 PE=1 SV=3	ERAP1_HUMAN	ERAP1	107 kDa	2	0	0	0	0	0	2	3	4	0	0	0
623	Syntaxin-6 OS=Homo sapiens OX=9606 GN=STX6 PE=1 SV=1	STX6_HUMAN	STX6	29 kDa	4	0	3	2	0	3	4	0	3	2	0	0
624	Immunoglobulin heavy constant gamma 4 OS=Homo sapiens OX=9606 GN=IGHG4 PE=1 SV=1	IGHG4_HUMAN	IGHG4	36 kDa	36	40	0	0	0	0	42	44	0	40	0	0
625	Sn1-specific diacylglycerol lipase beta OS=Homo sapiens OX=9606 GN=DAGLB PE=1 SV=2	DGLB_HUMAN	DAGLB	74 kDa	2	0	2	2	2	0	4	0	0	0	4	4
626	Dolichyl-phosphate beta-glucosyltransferase OS=Homo sapiens OX=9606 GN=ALG5 PE=1 SV=1	ALG5_HUMAN	ALG5	37 kDa	2	0	0	0	0	0	0	0	2	0	0	0
627	Solute carrier family 12 member 6 OS=Homo sapiens OX=9606 GN=SLC12A6 PE=1 SV=2	S12A6_HUMAN	SLC12A6	128 kDa	0	2	4	2	0	0	0	0	0	0	2	0
628	Hornerin OS=Homo sapiens OX=9606 GN=HRNR PE=1 SV=2	HORN_HUMAN	HRNR	282 kDa	0	4	0	0	0	3	3	5	0	5	0	0
629	Protein YIF1A OS=Homo sapiens OX=9606 GN=YIF1A PE=1 SV=2	YIF1A_HUMAN	YIF1A	32 kDa	4	0	5	5	4	3	0	0	0	0	0	2
630	Transmembrane channel-like protein 6 OS=Homo sapiens OX=9606 GN=TMC6 PE=1 SV=2	TMC6_HUMAN	TMC6	90 kDa	0	0	0	0	0	3	3	3	4	0	0	3
631	Adaptin ear-binding coat-associated protein 2 OS=Homo sapiens OX=9606 GN=NECAP2 PE=1 SV=1	NECP2_HUMAN	NECAP2	28 kDa	0	0	0	0	0	3	0	0	2	0	0	0
632	Sorting nexin-9 OS=Homo sapiens OX=9606 GN=SNX9 PE=1 SV=1	SNX9_HUMAN	SNX9	67 kDa	0	2	0	4	4	4	0	0	0	0	0	0
633	MARVEL domain-containing protein 2 OS=Homo sapiens OX=9606 GN=MARVELD2 PE=1 SV=2	MALD2_HUMAN	MARVELD2	64 kDa	3	3	0	0	0	2	3	3	2	0	3	0
634	Secretory carrier-associated membrane protein 4 OS=Homo sapiens OX=9606 GN=SCAMP4 PE=1 SV=1	SCAM4_HUMAN	SCAMP4	26 kDa	2	0	2	3	0	2	4	3	2	3	2	0
635	Casein kinase I isoform gamma-2 OS=Homo sapiens OX=9606 GN=CSNK1G2 PE=1 SV=1	KC1G2_HUMAN	CSNK1G2	47 kDa	6	0	0	0	0	0	0	0	0	0	0	0
636	Charged multivesicular body protein 2a OS=Homo sapiens OX=9606 GN=CHMP2A PE=1 SV=1	CHM2A_HUMAN	CHMP2A	25 kDa	0	3	2	2	2	0	0	0	0	0	0	2
637	UBX domain-containing protein 1 OS=Homo sapiens OX=9606 GN=UBXN1 PE=1 SV=2	UBXN1_HUMAN	UBXN1	33 kDa	0	0	0	2	2	0	2	2	2	3	0	2
638	CD82 antigen OS=Homo sapiens OX=9606 GN=CD82 PE=1 SV=1	CD82_HUMAN	CD82	30 kDa	0	4	4	2	2	3	2	0	0	0	0	0
639	Protein lin-7 homolog C OS=Homo sapiens OX=9606 GN=LIN7C PE=1 SV=1	LIN7C_HUMAN	LIN7C	22 kDa	0	0	0	2	0	2	0	0	0	0	2	2
640	Ubiquitin carboxyl-terminal hydrolase 7 OS=Homo sapiens OX=9606 GN=USP7 PE=1 SV=2	UBP7_HUMAN	USP7	128 kDa	0	0	0	0	0	2	0	0	0	0	2	3

641	Adenosylhomocysteinase OS=Homo sapiens OX=9606 GN=AHCY PE=1 SV=4	SAHH_HUMAN	AHCY	48 kDa	2	3	0	0	4	0	4	5	0	0	0	0
642	Dolichyldiphosphatase 1 OS=Homo sapiens OX=9606 GN=DOLPP1 PE=2 SV=1	DOPP1_HUMAN	DOLPP1	27 kDa	0	0	0	0	2	0	2	0	0	0	0	3
643	Protein PRRC1 OS=Homo sapiens OX=9606 GN=PRRC1 PE=1 SV=1	PRRC1_HUMAN	PRRC1	47 kDa	0	0	0	4	0	3	0	0	0	0	0	2
644	N-acyl-phosphatidylethanolamine-hydrolyzing phospholipase D OS=Homo sapiens OX=9606 GN=NAPEPLD PE=1 SV=2	NAPEP_HUMAN	NAPEPLD	46 kDa	0	3	4	4	0	2	0	0	2	0	0	0
645	Cullin-2 OS=Homo sapiens OX=9606 GN=CUL2 PE=1 SV=2	CUL2_HUMAN	CUL2	87 kDa	0	2	0	2	4	3	0	2	0	0	3	2
646	Disks large homolog 1 OS=Homo sapiens OX=9606 GN=DLG1 PE=1 SV=2	DLG1_HUMAN	DLG1	100 kDa	2	0	0	0	4	3	0	0	0	0	0	2
647	Glycogen [starch] synthase, muscle OS=Homo sapiens OX=9606 GN=GYS1 PE=1 SV=2	GYS1_HUMAN	GYS1	84 kDa	0	3	0	0	0	3	0	0	0	2	0	0
648	Sec1 family domain-containing protein 1 OS=Homo sapiens OX=9606 GN=SCFD1 PE=1 SV=4	SCFD1_HUMAN	SCFD1	72 kDa	5	0	2	0	0	6	0	0	0	0	0	0
649	Rho-associated protein kinase 2 OS=Homo sapiens OX=9606 GN=ROCK2 PE=1 SV=4	ROCK2_HUMAN	ROCK2	161 kDa	0	0	2	2	0	2	0	0	0	0	0	0
650	60S acidic ribosomal protein P0 OS=Homo sapiens OX=9606 GN=RPLP0 PE=1 SV=1	RLA0_HUMAN	RPLP0	34 kDa	0	0	0	5	2	3	2	0	0	0	0	2
651	Immunoglobulin lambda-1 light chain OS=Homo sapiens OX=9606 PE=1 SV=1	IGL1_HUMAN (+1)		23 kDa	0	0	0	0	0	0	12	0	0	0	0	14
652	Keratin, type II cytoskeletal 5 OS=Homo sapiens OX=9606 GN=KRT5 PE=1 SV=3	K2C5_HUMAN	KRT5	62 kDa	0	9	0	0	0	0	9	15	0	9	0	0
653	Protein FAM234A OS=Homo sapiens OX=9606 GN=FAM234A PE=1 SV=1	F234A_HUMAN	FAM234A	60 kDa	0	0	0	0	0	0	7	0	2	0	3	0
654	Serine/threonine-protein kinase TAO3 OS=Homo sapiens OX=9606 GN=TAOK3 PE=1 SV=2	TAOK3_HUMAN	TAOK3	105 kDa	2	0	0	4	6	2	0	0	0	2	0	2
655	Syntaxin-16 OS=Homo sapiens OX=9606 GN=STX16 PE=1 SV=3	STX16_HUMAN	STX16	37 kDa	0	2	0	0	0	3	3	4	2	2	5	2
656	Guanine nucleotide-binding protein G(I)/G(S)/G(O) subunit gamma-2 OS=Homo sapiens OX=9606 GN=GNG2 PE=1 SV=2	GBG2_HUMAN	GNG2	8 kDa	0	0	2	0	0	0	0	0	0	0	0	0
657	Actin-related protein 2/3 complex subunit 1A OS=Homo sapiens OX=9606 GN=ARPC1A PE=2 SV=2	ARC1A_HUMAN	ARPC1A	42 kDa	0	2	3	0	3	0	0	0	0	0	0	2
658	Platelet basic protein OS=Homo sapiens OX=9606 GN=PPBP PE=1 SV=3	CXCL7_HUMAN	PPBP	14 kDa	4	2	0	0	2	0	0	3	0	3	2	0
659	S-phase kinase-associated protein 1 OS=Homo sapiens OX=9606 GN=SKP1 PE=1 SV=2	SKP1_HUMAN	SKP1	19 kDa	0	0	0	2	0	3	0	0	0	0	0	0
660	Voltage-gated potassium channel subunit beta-2 OS=Homo sapiens OX=9606 GN=KCNAB2 PE=1 SV=2	KCAB2_HUMAN	KCNAB2	41 kDa	0	3	3	3	0	2	0	2	0	0	0	0
661	ATPase ASNA1 OS=Homo sapiens OX=9606 GN=ASNA1 PE=1 SV=2	ASNA_HUMAN	ASNA1	39 kDa	0	0	3	0	3	3	0	0	0	0	0	0
662	60S ribosomal protein L4 OS=Homo sapiens OX=9606 GN=RPL4 PE=1 SV=5	RL4_HUMAN	RPL4	48 kDa	0	0	0	5	0	3	0	0	0	0	0	0
663	E3 ubiquitin/ISG15 ligase TRIM25 OS=Homo sapiens OX=9606 GN=TRIM25 PE=1 SV=2	TRI25_HUMAN	TRIM25	71 kDa	2	4	3	3	3	6	0	0	0	0	0	0
664	Cyclin-Y OS=Homo sapiens OX=9606 GN=CCNY PE=1 SV=2	CCNY_HUMAN	CCNY	39 kDa	0	0	0	0	0	0	0	0	0	2	3	0
665	Actin-related protein 3 OS=Homo sapiens OX=9606 GN=ACTR3 PE=1 SV=3	ARP3_HUMAN	ACTR3	47 kDa	0	0	3	3	3	4	0	0	2	0	0	4
666	Solute carrier family 22 member 23 OS=Homo sapiens OX=9606 GN=SLC22A23 PE=1 SV=2	S22AN_HUMAN	SLC22A23	74 kDa	0	0	0	0	0	0	0	0	3	0	0	2
667	26S proteasome non-ATPase regulatory subunit 9 OS=Homo sapiens OX=9606 GN=PSMD9 PE=1 SV=3	PSMD9_HUMAN	PSMD9	25 kDa	0	0	0	0	2	4	3	0	3	2	0	0
668	Ribonucleoside-diphosphate reductase large subunit OS=Homo sapiens OX=9606 GN=RRM1 PE=1 SV=1	RIR1_HUMAN	RRM1	90 kDa	0	0	3	5	6	3	0	0	0	0	0	0
669	Ribosome maturation protein SBDS OS=Homo sapiens OX=9606 GN=SBDS PE=1 SV=4	SBDS_HUMAN	SBDS	29 kDa	0	0	0	0	0	0	0	3	0	0	4	0
670	Cyclin-dependent kinase 2 OS=Homo sapiens OX=9606 GN=CDK2 PE=1 SV=2	CDK2_HUMAN	CDK2	34 kDa	0	3	0	0	2	2	0	0	0	0	3	3
671	Reticulophagy regulator 3 OS=Homo sapiens OX=9606 GN=RETREG3 PE=1 SV=1	RETR3_HUMAN	RETREG3	51 kDa	0	3	4	3	3	2	0	0	0	0	0	0
672	CTP synthase 2 OS=Homo sapiens OX=9606 GN=CTPS2 PE=1 SV=1	PYRG2_HUMAN	CTPS2	66 kDa	0	0	0	2	3	5	0	0	0	0	4	0
673	Elongation factor 1-gamma OS=Homo sapiens OX=9606 GN=EEF1G PE=1 SV=3	EF1G_HUMAN	EEF1G	50 kDa	0	7	2	4	6	0	0	0	0	0	0	0
674	Methylosome subunit pICln OS=Homo sapiens OX=9606 GN=CLNS1A PE=1 SV=1	ICLN_HUMAN	CLNS1A	26 kDa	2	0	0	3	3	3	0	0	0	0	0	0
675	Coiled-coil domain-containing protein 124 OS=Homo sapiens OX=9606 GN=CCDC124 PE=1 SV=1	CC124_HUMAN	CCDC124	26 kDa	0	3	2	3	3	0	0	0	0	0	3	0
676	WD repeat-containing protein 26 OS=Homo sapiens OX=9606 GN=WDR26 PE=1 SV=3	WDR26_HUMAN	WDR26	72 kDa	0	0	0	0	4	0	0	0	2	0	3	0
677	PQ-loop repeat-containing protein 1 OS=Homo sapiens OX=9606 GN=PQLC1 PE=1 SV=1	PQLC1_HUMAN	PQLC1	30 kDa	2	0	0	0	0	0	2	0	0	0	2	0
678	H(+)/Cl(-) exchange transporter 3 OS=Homo sapiens OX=9606 GN=CLCN3 PE=1 SV=2	CLCN3_HUMAN	CLCN3	91 kDa	0	0	0	0	0	0	3	2	0	0	0	0
679	Bisphosphoglycerate mutase OS=Homo sapiens OX=9606 GN=BPGM PE=1 SV=2	PMGE_HUMAN	BPGM	30 kDa	0	0	0	0	3	0	4	3	2	0	0	0
680	Ran GTPase-activating protein 1 OS=Homo sapiens OX=9606 GN=RANGAP1 PE=1 SV=1	RAGP1_HUMAN	RANGAP1	64 kDa	0	0	0	0	2	3	2	3	0	0	0	0
681	Transferrin receptor protein 1 OS=Homo sapiens OX=9606 GN=TFRC PE=1 SV=2	TFR1_HUMAN	TFRC	85 kDa	5	0	6	2	0	2	0	0	0	0	0	0
682	Ephrin-B1 OS=Homo sapiens OX=9606 GN=EFNB1 PE=1 SV=1	EFNB1_HUMAN	EFNB1	38 kDa	0	0	0	0	0	0	2	0	0	0	0	0
683	RNA-binding motif protein, X chromosome OS=Homo sapiens OX=9606 GN=RBMX PE=1 SV=3	RBMX_HUMAN	RBMX	42 kDa	2	0	0	3	0	2	2	0	0	0	0	0
684	Microsomal glutathione S-transferase 2 OS=Homo sapiens OX=9606 GN=MGST2 PE=1 SV=1	MGST2_HUMAN	MGST2	17 kDa	3	0	0	2	0	2	0	0	3	0	0	0
685	V-type proton ATPase subunit G 1 OS=Homo sapiens OX=9606 GN=ATP6V1G1 PE=1 SV=3	VATG1_HUMAN	ATP6V1G1	14 kDa	0	0	0	0	4	3	3	0	0	0	0	0
686	Deoxyribonuclease-1-like 1 OS=Homo sapiens OX=9606 GN=DNASE1L1 PE=1 SV=1	DNSL1_HUMAN	DNASE1L1	34 kDa	0	0	0	0	2	0	0	0	0	0	0	0
687	Tumor necrosis factor receptor superfamily member 6 OS=Homo sapiens OX=9606 GN=FAS PE=1 SV=1	TNR6_HUMAN	FAS	38 kDa	0	0	0	0	0	0	2	2	0	3	4	0
688	UPF0449 protein C19orf25 OS=Homo sapiens OX=9606 GN=C19orf25 PE=1 SV=2	CS025_HUMAN	C19orf25	13 kDa	0	0	0	0	2	0	0	0	0	0	0	0
689	Mitochondrial fission 1 protein OS=Homo sapiens OX=9606 GN=FIS1 PE=1 SV=2	FIS1_HUMAN	FIS1	17 kDa	0	0	0	0	0	0	0	0	0	0	2	0
690	Autophagy-related protein 9A OS=Homo sapiens OX=9606 GN=ATG9A PE=1 SV=3	ATG9A_HUMAN	ATG9A	94 kDa	0	0	0	0	0	0	2	0	0	0	2	0
691	Ubiquitin-like protein 4A OS=Homo sapiens OX=9606 GN=UBL4A PE=1 SV=1	UBL4A_HUMAN	UBL4A	18 kDa	3	3	0	0	0	3	0	0	0	0	0	0
692	Palmitoyltransferase ZDHHC3 OS=Homo sapiens OX=9606 GN=ZDHHC3 PE=1 SV=2	ZDHC3_HUMAN	ZDHHC3	34 kDa	0	0	0	0	0	3	0	0	0	0	0	0
693	Synaptic vesicle membrane protein VAT-1 homolog OS=Homo sapiens OX=9606 GN=VAT1 PE=1 SV=2	VAT1_HUMAN	VAT1	42 kDa	0	0	0	2	0	3	0	0	0	3	2	2
694	Transmembrane emp24 domain-containing protein 5 OS=Homo sapiens OX=9606 GN=TMED5 PE=1 SV=1	TMED5_HUMAN	TMED5	26 kDa	0	2	0	0	0	0	0	2	0	2	2	0
695	Emerin OS=Homo sapiens OX=9606 GN=EMD PE=1 SV=1	EMD_HUMAN	EMD	29 kDa	0	2	2	0	2	0	4	0	2	0	2	0
696	Immunoglobulin heavy variable 3-7 OS=Homo sapiens OX=9606 GN=IGHV3-7 PE=1 SV=2	HV307_HUMAN	IGHV3-7	13 kDa	0	0	0	0	3	0	3	0	0	0	0	0
697	DnaJ homolog subfamily A member 4 OS=Homo sapiens OX=9606 GN=DNAJA4 PE=1 SV=1	DNJA4_HUMAN	DNAJA4	45 kDa	0	0	0	4	5	6	0	0	0	0	0	3
698	Complement C3 OS=Homo sapiens OX=9606 GN=C3 PE=1 SV=2	CO3_HUMAN	C3	187 kDa	0	0	0	0	2	2	0	0	0	0	0	5
699	Casein kinase I isoform gamma-1 OS=Homo sapiens OX=9606 GN=CSNK1G1 PE=1 SV=1	KC1G1_HUMAN	CSNK1G1	49 kDa	0	0	0	0	0	0	6	4	5	0	4	0
700	ADP-dependent glucokinase OS=Homo sapiens OX=9606 GN=ADPGK PE=1 SV=1	ADPGK_HUMAN	ADPGK	54 kDa	0	0	0	0	0	0	0	0	2	0	2	0
701	Heterogeneous nuclear ribonucleoprotein D0 OS=Homo sapiens OX=9606 GN=HNRNPD PE=1 SV=1	HNRPD_HUMAN	HNRNPD	38 kDa	0	0	0	0	0	3	0	0	0	0	0	0
702	Immunoglobulin kappa variable 3-15 OS=Homo sapiens OX=9606 GN=IGKV3-15 PE=1 SV=2	KV315_HUMAN	IGKV3-15	12 kDa	0	0	0	3	0	0	0	0	0	0	0	0
703	Elongin-C OS=Homo sapiens OX=9606 GN=ELOC PE=1 SV=1	ELOC_HUMAN	ELOC	12 kDa	0	0	0	0	3	0	0	0	0	0	0	0
704	Thioredoxin OS=Homo sapiens OX=9606 GN=TXN PE=1 SV=3	THIO_HUMAN	TXN	12 kDa	0	0	0	0	0	3	0	0	0	0	0	0
705	Optineurin OS=Homo sapiens OX=9606 GN=OPTN PE=1 SV=3	OPTN_HUMAN	OPTN	66 kDa	0	0	0	0	0	0	4	2	0	2	3	2
706	Leucyl-cystinyl aminopeptidase OS=Homo sapiens OX=9606 GN=LNPEP PE=1 SV=3	LCAP_HUMAN	LNPEP	117 kDa	0	0	0	0	0	0	0	0	0	0	2	0
707	Vacuolar protein sorting-associated protein 13C OS=Homo sapiens OX=9606 GN=VPS13C PE=1 SV=1	VP13C_HUMAN	VPS13C	422 kDa	0	2	0	0	0	0	0	0	0	0	0	0
708	Peptidyl-prolyl cis-trans isomerase FKBP2 OS=Homo sapiens OX=9606 GN=FKBP2 PE=1 SV=2	FKBP2_HUMAN	FKBP2	16 kDa	0	0	0	0	0	0	3	4	2	3	2	0
709	Peflin OS=Homo sapiens OX=9606 GN=PEF1 PE=1 SV=1	PEF1_HUMAN	PEF1	30 kDa	2	0	2	0	0	2	0	0	0	0	0	0
710	Polyadenylate-binding protein 1 OS=Homo sapiens OX=9606 GN=PABPC1 PE=1 SV=2	PABP1_HUMAN	PABPC1	71 kDa	0	0	0	0	2	0	0	0	0	0	2	2
711	Peptidyl-prolyl cis-trans isomerase A OS=Homo sapiens OX=9606 GN=PPIA PE=1 SV=2	PPIA_HUMAN	PPIA	18 kDa	0	0	2	0	0	0	0	3	0	0	0	0
712	Claudin domain-containing protein 1 OS=Homo sapiens OX=9606 GN=CLDND1 PE=1 SV=1	CLDN1_HUMAN	CLDND1	29 kDa	0	0	0	0	0	0	2	3	2	0	3	2
713	Structural maintenance of chromosomes protein 4 OS=Homo sapiens OX=9606 GN=SMC4 PE=1 SV=2	SMC4_HUMAN	SMC4	147 kDa	0	2	2	0	3	4	0	0	0	0	0	3
714	60S ribosomal protein L12 OS=Homo sapiens OX=9606 GN=RPL12 PE=1 SV=1	RL12_HUMAN	RPL12	18 kDa	0	0	3	0	3	2	4	0	0	0	0	0
715	Rab-interacting lysosomal protein OS=Homo sapiens OX=9606 GN=RILP PE=1 SV=1	RILP_HUMAN	RILP	44 kDa	0	0	0	0	3	3	0	0	0	0	0	0
716	Calumenin OS=Homo sapiens OX=9606 GN=CALU PE=1 SV=2	CALU_HUMAN	CALU	37 kDa	0	0	0	0	0	0	4	0	0	2	0	0
717	B-cell receptor-associated protein 29 OS=Homo sapiens OX=9606 GN=BCAP29 PE=1 SV=2	BAP29_HUMAN	BCAP29	28 kDa	0	0	0	0	0	0	2	0	0	2	0	0
718	Secretory carrier-associated membrane protein 2 OS=Homo sapiens OX=9606 GN=SCAMP2 PE=1 SV=2	SCAM2_HUMAN	SCAMP2	37 kDa	0	0	0	0	0	0	0	0	0	0	3	0
719	Vacuolar protein sorting-associated protein 28 homolog OS=Homo sapiens OX=9606 GN=VPS28 PE=1 SV=1	VPS28_HUMAN	VPS28	25 kDa	0	0	0	0	4	3	0	2	0	0	3	0
720	Programmed cell death protein 5 OS=Homo sapiens OX=9606 GN=PDCD5 PE=1 SV=3	PDCD5_HUMAN	PDCD5	14 kDa	0	0	0	0	2	0	0	0	0	0	0	0
721	Tubulin--tyrosine ligase-like protein 12 OS=Homo sapiens OX=9606 GN=TTLL12 PE=1 SV=2	TTL12_HUMAN	TTLL12	74 kDa	0	0	0	0	3	4	0	0	0	0	0	0
722	Armadillo repeat-containing protein 8 OS=Homo sapiens OX=9606 GN=ARMC8 PE=1 SV=2	ARMC8_HUMAN	ARMC8	76 kDa	0	2	0	0	0	0	0	0	0	0	0	0
723	Tyrosine-protein kinase Yes OS=Homo sapiens OX=9606 GN=YES1 PE=1 SV=3	YES_HUMAN	YES1	61 kDa	2	0	0	0	2	0	0	0	0	0	0	0
724	Syntaxin-18 OS=Homo sapiens OX=9606 GN=STX18 PE=1 SV=1	STX18_HUMAN	STX18	39 kDa	0	2	0	0	0	0	0	0	0	0	0	0
725	E3 ubiquitin-protein ligase TRIM58 OS=Homo sapiens OX=9606 GN=TRIM58 PE=2 SV=2	TRI58_HUMAN	TRIM58	55 kDa	0	0	0	0	4	8	0	0	0	0	0	0
726	Zinc transporter 7 OS=Homo sapiens OX=9606 GN=SLC30A7 PE=2 SV=1	ZNT7_HUMAN	SLC30A7	42 kDa	0	0	0	0	0	0	3	0	0	0	3	0
727	DDB1- and CUL4-associated factor 6 OS=Homo sapiens OX=9606 GN=DCAF6 PE=1 SV=1	DCAF6_HUMAN	DCAF6	96 kDa	0	0	0	0	0	0	0	0	2	0	2	0
728	Protein argonaute-1 OS=Homo sapiens OX=9606 GN=AGO1 PE=1 SV=3	AGO1_HUMAN	AGO1	97 kDa	56	0	0	0	0	0	0	0	0	0	0	0
729	F-box/LRR-repeat protein 20 OS=Homo sapiens OX=9606 GN=FBXL20 PE=1 SV=2	FXL20_HUMAN	FBXL20	48 kDa	0	0	0	0	0	0	0	2	3	3	0	2
730	Pyruvate kinase PKLR OS=Homo sapiens OX=9606 GN=PKLR PE=1 SV=2	KPYR_HUMAN	PKLR	62 kDa	0	0	0	0	0	2	0	2	0	0	0	0
731	Tumor protein D54 OS=Homo sapiens OX=9606 GN=TPD52L2 PE=1 SV=2	TPD54_HUMAN	TPD52L2	22 kDa	0	3	0	0	4	2	0	0	0	0	0	0
732	Charged multivesicular body protein 4b OS=Homo sapiens OX=9606 GN=CHMP4B PE=1 SV=1	CHM4B_HUMAN	CHMP4B	25 kDa	0	0	0	0	2	5	0	0	0	0	0	5

733	VIP36-like protein OS=Homo sapiens OX=9606 GN=LMAN2L PE=1 SV=1	LMA2L_HUMAN	LMAN2L	40 kDa	0	0	0	0	0	0	0	0	0	0	0	2
734	Condensin complex subunit 3 OS=Homo sapiens OX=9606 GN=NCAPG PE=1 SV=1	CND3_HUMAN	NCAPG	114 kDa	0	0	2	0	3	3	0	3	0	0	0	0
735	Protein canopy homolog 2 OS=Homo sapiens OX=9606 GN=CNPY2 PE=1 SV=1	CNPY2_HUMAN	CNPY2	21 kDa	0	0	0	0	0	0	0	0	0	3	0	0
736	Translocon-associated protein subunit alpha OS=Homo sapiens OX=9606 GN=SSR1 PE=1 SV=3	SSRA_HUMAN	SSR1	32 kDa	0	2	0	0	0	0	2	0	2	0	2	3
737	26S proteasome non-ATPase regulatory subunit 10 OS=Homo sapiens OX=9606 GN=PSMD10 PE=1 SV=1	PSD10_HUMAN	PSMD10	24 kDa	0	0	0	0	2	0	0	0	0	0	5	2
738	Calcium-binding and coiled-coil domain-containing protein 1 OS=Homo sapiens OX=9606 GN=CALCOCO1 PE=1 SV=2	CACO1_HUMAN	CALCOCO1	77 kDa	0	0	2	2	0	4	0	0	0	0	0	0
739	Vitamin K epoxide reductase complex subunit 1-like protein 1 OS=Homo sapiens OX=9606 GN=VKORC1L1 PE=1 SV=2	VKORL_HUMAN	VKORC1L1	20 kDa	0	0	0	0	0	0	0	0	0	0	2	0
740	Putative PIP5K1A and PSMD4-like protein OS=Homo sapiens OX=9606 GN=PIPSL PE=5 SV=1	PIPSL_HUMAN	PIPSL	95 kDa	0	0	0	6	8	0	0	0	0	0	0	0
741	Small glutamine-rich tetratricopeptide repeat-containing protein alpha OS=Homo sapiens OX=9606 GN=SGTA PE=1 SV=1	SGTA_HUMAN	SGTA	34 kDa	0	0	0	0	0	0	0	0	0	0	0	2
742	Cytokine receptor-like factor 3 OS=Homo sapiens OX=9606 GN=CRLF3 PE=1 SV=2	CRLF3_HUMAN	CRLF3	50 kDa	0	0	0	0	2	0	0	0	0	0	2	0
743	Structural maintenance of chromosomes protein 2 OS=Homo sapiens OX=9606 GN=SMC2 PE=1 SV=2	SMC2_HUMAN	SMC2	136 kDa	0	0	0	0	0	0	0	0	0	0	3	0
744	Casein kinase II subunit beta OS=Homo sapiens OX=9606 GN=CSNK2B PE=1 SV=1	CSK2B_HUMAN	CSNK2B	25 kDa	0	0	0	0	0	0	3	2	0	0	3	0
745	Calcium-regulated heat-stable protein 1 OS=Homo sapiens OX=9606 GN=CARHSP1 PE=1 SV=2	CHSP1_HUMAN	CARHSP1	16 kDa	0	0	0	0	2	0	0	0	0	0	0	0
746	Quinone oxidoreductase OS=Homo sapiens OX=9606 GN=CRYZ PE=1 SV=1	QOR_HUMAN	CRYZ	35 kDa	0	0	0	4	2	2	0	0	0	0	0	0
747	Apolipoprotein L3 OS=Homo sapiens OX=9606 GN=APOL3 PE=1 SV=3	APOL3_HUMAN	APOL3	44 kDa	0	0	0	0	0	0	0	2	0	0	0	2
748	Derlin-1 OS=Homo sapiens OX=9606 GN=DERL1 PE=1 SV=1	DERL1_HUMAN	DERL1	29 kDa	0	0	0	0	0	0	0	2	0	0	0	0
749	DCN1-like protein 1 OS=Homo sapiens OX=9606 GN=DCUN1D1 PE=1 SV=1	DCNL1_HUMAN	DCUN1D1	30 kDa	0	0	0	0	2	0	0	0	0	0	0	0
750	Proteasomal ubiquitin receptor ADRM1 OS=Homo sapiens OX=9606 GN=ADRM1 PE=1 SV=2	ADRM1_HUMAN	ADRM1	42 kDa	0	0	0	2	0	0	2	2	0	0	2	0
751	Ras-related protein Rab-11B OS=Homo sapiens OX=9606 GN=RAB11B PE=1 SV=4	RB11B_HUMAN	RAB11B	24 kDa	2	0	2	0	0	0	4	0	0	0	0	0
752	Tether containing UBX domain for GLUT4 OS=Homo sapiens OX=9606 GN=ASPSCR1 PE=1 SV=1	ASPC1_HUMAN	ASPSCR1	60 kDa	0	0	0	0	0	0	0	0	0	2	0	0
753	Myeloid-derived growth factor OS=Homo sapiens OX=9606 GN=MYDGF PE=1 SV=1	MYDGF_HUMAN	MYDGF	19 kDa	0	0	0	0	0	0	3	2	0	3	0	0
754	V-type proton ATPase subunit E 1 OS=Homo sapiens OX=9606 GN=ATP6V1E1 PE=1 SV=1	VATE1_HUMAN	ATP6V1E1	26 kDa	0	0	0	0	3	2	4	0	0	0	0	0
755	E3 ubiquitin-protein ligase rififylin OS=Homo sapiens OX=9606 GN=RFFL PE=1 SV=1	RFFL_HUMAN	RFFL	41 kDa	0	0	0	0	0	0	4	0	0	0	2	0
756	Arf-GAP domain and FG repeat-containing protein 1 OS=Homo sapiens OX=9606 GN=AGFG1 PE=1 SV=2	AGFG1_HUMAN	AGFG1	58 kDa	0	0	0	0	0	0	0	0	0	2	0	0
757	Type 1 phosphatidylinositol 4,5-bisphosphate 4-phosphatase OS=Homo sapiens OX=9606 GN=PIP4P1 PE=1 SV=1	PP4P1_HUMAN	PIP4P1	29 kDa	0	0	0	0	0	0	2	0	2	0	0	0
758	RING finger and SPRY domain-containing protein 1 OS=Homo sapiens OX=9606 GN=RSPRY1 PE=1 SV=1	RSPRY_HUMAN	RSPRY1	64 kDa	0	0	0	0	0	0	2	2	2	0	0	0
759	Interferon-stimulated gene 20 kDa protein OS=Homo sapiens OX=9606 GN=ISG20 PE=1 SV=2	ISG20_HUMAN	ISG20	20 kDa	0	0	2	2	0	0	0	0	0	2	0	2
760	Vacuolar protein-sorting-associated protein 36 OS=Homo sapiens OX=9606 GN=VPS36 PE=1 SV=1	VPS36_HUMAN	VPS36	44 kDa	0	0	0	2	0	0	0	0	0	0	0	2
761	Cysteine-rich hydrophobic domain-containing protein 2 OS=Homo sapiens OX=9606 GN=CHIC2 PE=1 SV=1	CHIC2_HUMAN	CHIC2	19 kDa	0	2	0	0	0	0	0	0	0	0	0	0
762	Eukaryotic peptide chain release factor subunit 1 OS=Homo sapiens OX=9606 GN=ETF1 PE=1 SV=3	ERF1_HUMAN	ETF1	49 kDa	0	0	0	3	0	0	0	0	0	0	0	0
763	Signal peptidase complex catalytic subunit SEC11A OS=Homo sapiens OX=9606 GN=SEC11A PE=1 SV=1	SC11A_HUMAN	SEC11A	21 kDa	0	0	0	0	0	0	0	0	0	0	0	3
764	Signal peptidase complex subunit 2 OS=Homo sapiens OX=9606 GN=SPCS2 PE=1 SV=3	SPCS2_HUMAN	SPCS2	25 kDa	0	0	0	0	0	0	0	2	0	0	0	0
765	Eukaryotic translation initiation factor 2 subunit 2 OS=Homo sapiens OX=9606 GN=EIF2S2 PE=1 SV=2	IF2B_HUMAN	EIF2S2	38 kDa	0	0	0	2	0	0	0	0	0	0	0	0
766	Vesicle transport protein USE1 OS=Homo sapiens OX=9606 GN=USE1 PE=1 SV=2	USE1_HUMAN	USE1	29 kDa	2	0	0	0	0	0	0	0	0	0	0	0
767	Lck-interacting transmembrane adapter 1 OS=Homo sapiens OX=9606 GN=LIME1 PE=1 SV=1	LIME1_HUMAN	LIME1	31 kDa	0	0	0	0	0	0	3	0	0	0	3	0
768	Erbin OS=Homo sapiens OX=9606 GN=ERBIN PE=1 SV=2	ERBIN_HUMAN	ERBIN	158 kDa	2	0	0	0	0	0	0	0	0	0	0	0
769	Casein kinase II subunit alpha' OS=Homo sapiens OX=9606 GN=CSNK2A2 PE=1 SV=1	CSK22_HUMAN	CSNK2A2	41 kDa	0	2	0	0	3	0	0	0	0	0	0	0
770	BRISC complex subunit Abraxas 2 OS=Homo sapiens OX=9606 GN=ABRAXAS2 PE=1 SV=2	ABRX2_HUMAN	ABRAXAS2	47 kDa	0	2	0	0	2	0	0	0	0	0	0	0
771	Ethylmalonyl-CoA decarboxylase OS=Homo sapiens OX=9606 GN=ECHDC1 PE=1 SV=2	ECHD1_HUMAN	ECHDC1	34 kDa	0	0	0	0	0	2	0	0	0	0	0	0
772	Pterin-4-alpha-carbinolamine dehydratase 2 OS=Homo sapiens OX=9606 GN=PCBD2 PE=1 SV=4	PHS2_HUMAN	PCBD2	14 kDa	0	0	0	0	0	0	0	0	0	3	0	4
773	Protein S100-A6 OS=Homo sapiens OX=9606 GN=S100A6 PE=1 SV=1	S10A6_HUMAN	S100A6	10 kDa	0	0	0	0	0	2	0	0	0	0	0	0
774	Translocon-associated protein subunit delta OS=Homo sapiens OX=9606 GN=SSR4 PE=1 SV=1	SSRD_HUMAN	SSR4	19 kDa	0	0	0	0	0	0	0	0	2	0	0	0
775	ELMO domain-containing protein 2 OS=Homo sapiens OX=9606 GN=ELMOD2 PE=1 SV=1	ELMD2_HUMAN	ELMOD2	35 kDa	0	0	0	0	0	0	0	2	2	0	0	2
776	Coronin-1C OS=Homo sapiens OX=9606 GN=CORO1C PE=1 SV=1	COR1C_HUMAN	CORO1C	53 kDa	0	0	0	0	0	2	0	0	0	0	0	0
777	Prosaposin OS=Homo sapiens OX=9606 GN=PSAP PE=1 SV=2	SAP_HUMAN	PSAP	58 kDa	0	0	0	0	0	0	2	0	0	0	0	0
778	Alpha/beta hydrolase domain-containing protein 17B OS=Homo sapiens OX=9606 GN=ABHD17B PE=1 SV=1	AB17B_HUMAN	ABHD17B	32 kDa	0	0	0	0	0	0	0	0	0	2	0	0
779	Probable phospholipid-transporting ATPase IF OS=Homo sapiens OX=9606 GN=ATP11B PE=1 SV=2	AT11B_HUMAN	ATP11B	134 kDa	0	0	0	2	0	0	0	0	0	0	0	2
780	Erlin-2 OS=Homo sapiens OX=9606 GN=ERLIN2 PE=1 SV=1	ERLN2_HUMAN	ERLIN2	38 kDa	0	0	0	0	0	0	2	2	0	0	0	0
781	Condensin complex subunit 2 OS=Homo sapiens OX=9606 GN=NCAPH PE=1 SV=3	CND2_HUMAN	NCAPH	83 kDa	0	2	0	0	0	0	0	0	0	0	0	0
782	Eukaryotic translation initiation factor 4B OS=Homo sapiens OX=9606 GN=EIF4B PE=1 SV=2	IF4B_HUMAN	EIF4B	69 kDa	0	0	0	2	0	0	3	0	0	0	0	0
783	RAD50-interacting protein 1 OS=Homo sapiens OX=9606 GN=RINT1 PE=1 SV=1	RINT1_HUMAN	RINT1	91 kDa	0	0	2	2	0	0	0	0	0	0	0	0
784	Receptor of activated protein C kinase 1 OS=Homo sapiens OX=9606 GN=RACK1 PE=1 SV=3	RACK1_HUMAN	RACK1	35 kDa	2	0	0	0	0	0	0	0	0	0	0	0
785	Thioredoxin reductase-like selenoprotein T OS=Homo sapiens OX=9606 GN=SELENOT PE=1 SV=2	SELT_HUMAN	SELENOT	22 kDa	0	0	0	0	0	0	2	0	0	0	0	0
786	Protein EFR3 homolog A OS=Homo sapiens OX=9606 GN=EFR3A PE=1 SV=2	EFR3A_HUMAN	EFR3A	93 kDa	0	0	0	3	0	0	0	0	0	0	0	0
787	Regulator of nonsense transcripts 3B OS=Homo sapiens OX=9606 GN=UPF3B PE=1 SV=1	REN3B_HUMAN	UPF3B	58 kDa	0	0	0	2	0	0	0	0	0	0	0	0
788	Alpha-actinin-4 OS=Homo sapiens OX=9606 GN=ACTN4 PE=1 SV=2	ACTN4_HUMAN	ACTN4	105 kDa	0	2	2	0	0	2	0	0	0	0	0	0
789	HLA class I histocompatibility antigen, B-51 alpha chain OS=Homo sapiens OX=9606 GN=HLA-B PE=1 SV=1	1B51_HUMAN (+3)	HLA-B	41 kDa	0	0	0	0	0	0	2	2	0	0	0	2
790	Translin-associated protein X OS=Homo sapiens OX=9606 GN=TSNAX PE=1 SV=1	TSNAX_HUMAN	TSNAX	33 kDa	0	0	0	0	4	0	0	0	0	0	0	0
791	Signal peptidase complex subunit 3 OS=Homo sapiens OX=9606 GN=SPCS3 PE=1 SV=1	SPCS3_HUMAN	SPCS3	20 kDa	0	0	0	0	0	0	0	0	0	0	3	0
792	Translin OS=Homo sapiens OX=9606 GN=TSN PE=1 SV=1	TSN_HUMAN	TSN	26 kDa	0	0	0	0	0	2	0	0	0	0	0	0
793	60S ribosomal protein L30 OS=Homo sapiens OX=9606 GN=RPL30 PE=1 SV=2	RL30_HUMAN	RPL30	13 kDa	0	0	0	0	2	0	0	0	0	0	0	3
794	14-3-3 protein theta OS=Homo sapiens OX=9606 GN=YWHAQ PE=1 SV=1	1433T_HUMAN	YWHAQ	28 kDa	0	0	0	0	0	0	8	0	0	0	0	0
795	DnaJ homolog subfamily B member 11 OS=Homo sapiens OX=9606 GN=DNAJB11 PE=1 SV=1	DJB11_HUMAN	DNAJB11	41 kDa	0	0	0	0	0	0	2	0	0	2	0	0
796	Hemoglobin subunit theta-1 OS=Homo sapiens OX=9606 GN=HBQ1 PE=1 SV=2	HBAT_HUMAN	HBQ1	16 kDa	0	0	0	0	0	0	0	0	0	0	0	2
797	Protein SLFN14 OS=Homo sapiens OX=9606 GN=SLFN14 PE=1 SV=2	SLN14_HUMAN	SLFN14	104 kDa	0	0	2	0	3	2	0	0	0	0	0	0
798	Nascent polypeptide-associated complex subunit alpha OS=Homo sapiens OX=9606 GN=NACA PE=1 SV=1	NACA_HUMAN	NACA	23 kDa	0	0	0	0	5	0	0	0	0	0	0	0
799	Protein argonaute-3 OS=Homo sapiens OX=9606 GN=AGO3 PE=1 SV=2	AGO3_HUMAN	AGO3	97 kDa	0	0	58	0	0	0	0	0	0	0	0	0
800	Serine/threonine-protein kinase mTOR OS=Homo sapiens OX=9606 GN=MTOR PE=1 SV=1	MTOR_HUMAN	MTOR	289 kDa	0	0	0	0	0	0	0	2	0	0	0	0
801	Golgi SNAP receptor complex member 2 OS=Homo sapiens OX=9606 GN=GOSR2 PE=1 SV=2	GOSR2_HUMAN	GOSR2	25 kDa	0	2	0	0	0	0	0	0	0	0	0	0
802	Tyrosine-protein kinase Lyn OS=Homo sapiens OX=9606 GN=LYN PE=1 SV=3	LYN_HUMAN	LYN	59 kDa	0	3	0	0	0	0	0	0	0	0	0	0
803	Keratin, type II cuticular Hb6 OS=Homo sapiens OX=9606 GN=KRT86 PE=1 SV=1	KRT86_HUMAN	KRT86	53 kDa	6	0	0	0	0	0	0	0	0	0	0	0
804	N-terminal kinase-like protein OS=Homo sapiens OX=9606 GN=SCYL1 PE=1 SV=1	SCYL1_HUMAN	SCYL1	90 kDa	2	0	0	0	0	0	0	0	0	0	0	0
805	Nicotinate phosphoribosyltransferase OS=Homo sapiens OX=9606 GN=NAPRT PE=1 SV=2	PNCB_HUMAN	NAPRT	58 kDa	0	0	0	0	0	0	2	0	0	0	0	0
806	ADP-ribose glycohydrolase OARD1 OS=Homo sapiens OX=9606 GN=OARD1 PE=1 SV=2	OARD1_HUMAN	OARD1	17 kDa	0	0	0	0	0	0	0	2	0	0	0	0
807	Plasminogen activator inhibitor 1 RNA-binding protein OS=Homo sapiens OX=9606 GN=SERBP1 PE=1 SV=2	PAIRB_HUMAN	SERBP1	45 kDa	0	0	0	0	0	0	2	0	0	0	0	0
808	Condensin complex subunit 1 OS=Homo sapiens OX=9606 GN=NCAPD2 PE=1 SV=3	CND1_HUMAN	NCAPD2	157 kDa	0	2	2	0	0	0	0	0	0	0	0	0
809	60S ribosomal protein L7 OS=Homo sapiens OX=9606 GN=RPL7 PE=1 SV=1	RL7_HUMAN	RPL7	29 kDa	0	0	0	0	0	2	0	0	0	0	0	0
810	Dolichyl-diphosphooligosaccharide--protein glycosyltransferase 48 kDa subunit OS=Homo sapiens OX=9606 GN=DDOST PE=1 SV=4	OST48_HUMAN	DDOST	51 kDa	0	0	0	0	0	0	0	0	0	0	0	2
811	Immunoglobulin heavy variable 5-51 OS=Homo sapiens OX=9606 GN=IGHV5-51 PE=3 SV=1	HV551_HUMAN	IGHV5-51	13 kDa	0	0	0	0	0	0	0	0	0	0	2	0
812	Transmembrane protein with metallophosphoesterase domain OS=Homo sapiens OX=9606 GN=TMPPE PE=2 SV=2	TMPPE_HUMAN	TMPPE	49 kDa	0	0	0	0	0	0	3	0	0	0	0	0
813	Glutathione reductase, mitochondrial OS=Homo sapiens OX=9606 GN=GSR PE=1 SV=2	GSHR_HUMAN	GSR	56 kDa	0	0	0	0	2	2	0	0	0	0	0	0
814	Proteasomal ATPase-associated factor 1 OS=Homo sapiens OX=9606 GN=PAAF1 PE=1 SV=2	PAAF1_HUMAN	PAAF1	42 kDa	0	0	0	0	0	2	0	0	0	0	0	0
815	CTP synthase 1 OS=Homo sapiens OX=9606 GN=CTPS1 PE=1 SV=2	PYRG1_HUMAN	CTPS1	67 kDa	0	0	0	0	0	5	0	0	0	0	0	0
816	Keratin, type I cuticular Ha1 OS=Homo sapiens OX=9606 GN=KRT31 PE=1 SV=3	K1H1_HUMAN	KRT31	47 kDa	5	0	0	0	0	0	0	0	0	0	0	0
817	Procollagen galactosyltransferase 1 OS=Homo sapiens OX=9606 GN=COLGALT1 PE=1 SV=1	GT251_HUMAN	COLGALT1	72 kDa	0	0	0	0	0	0	2	0	0	0	0	0
818	Proteasome subunit beta type-8 OS=Homo sapiens OX=9606 GN=PSMB8 PE=1 SV=3	PSB8_HUMAN	PSMB8	30 kDa	0	0	0	0	2	0	0	0	0	0	0	0
819	Elongation factor 1-delta OS=Homo sapiens OX=9606 GN=EEF1D PE=1 SV=5	EF1D_HUMAN	EEF1D	31 kDa	0	0	0	0	2	0	0	0	0	0	0	0
820	Ran-specific GTPase-activating protein OS=Homo sapiens OX=9606 GN=RANBP1 PE=1 SV=1	RANG_HUMAN	RANBP1	23 kDa	0	0	0	0	3	0	0	0	0	0	0	0
821	Guanine nucleotide exchange factor for Rab-3A OS=Homo sapiens OX=9606 GN=RAB3IL1 PE=1 SV=1	R3GEF_HUMAN	RAB3IL1	43 kDa	0	0	0	0	0	2	0	0	0	0	0	0
822	Monocarboxylate transporter 2 OS=Homo sapiens OX=9606 GN=SLC16A7 PE=1 SV=2	MOT2_HUMAN	SLC16A7	52 kDa	0	0	0	0	0	0	2	0	0	0	0	0
823	Calcium load-activated calcium channel OS=Homo sapiens OX=9606 GN=TMCO1 PE=1 SV=2	TMCO1_HUMAN	TMCO1	27 kDa	0	0	0	0	0	0	2	0	0	0	0	0
824	Putative heat shock protein HSP 90-beta 4 OS=Homo sapiens OX=9606 GN=HSP90AB4P PE=5 SV=1	H90B4_HUMAN	HSP90AB4P	58 kDa	0	0	0	0	10	0	0	0	0	0	0	0

825	L-xylulose reductase OS=Homo sapiens OX=9606 GN=DCXR PE=1 SV=2	DCXR_HUMAN	DCXR	26 kDa	0	0	0	0	0	2	0	0	0	0	0	0
826	Gelsolin OS=Homo sapiens OX=9606 GN=GSN PE=1 SV=1	GELS_HUMAN	GSN	86 kDa	0	0	0	0	2	0	0	0	0	0	0	0
827	Sodium/hydrogen exchanger 6 OS=Homo sapiens OX=9606 GN=SLC9A6 PE=1 SV=2	SL9A6_HUMAN	SLC9A6	74 kDa	0	0	0	0	0	0	3	0	0	0	0	0
828	CD151 antigen OS=Homo sapiens OX=9606 GN=CD151 PE=1 SV=3	CD151_HUMAN	CD151	28 kDa	0	0	0	0	0	0	2	0	0	0	0	0
829	Immunoglobulin heavy constant delta OS=Homo sapiens OX=9606 GN=IGHD PE=1 SV=3	IGHD_HUMAN	IGHD	42 kDa	0	0	0	2	0	0	0	0	0	0	0	0
830	Endoglin OS=Homo sapiens OX=9606 GN=ENG PE=1 SV=2	EGLN_HUMAN	ENG	71 kDa	0	0	0	0	0	0	0	0	0	2	0	0
831	STE20-like serine/threonine-protein kinase OS=Homo sapiens OX=9606 GN=SLK PE=1 SV=1	SLK_HUMAN	SLK	143 kDa	0	0	0	0	2	0	0	0	0	0	0	0
832	Eukaryotic initiation factor 4A-I OS=Homo sapiens OX=9606 GN=EIF4A1 PE=1 SV=1	IF4A1_HUMAN	EIF4A1	46 kDa	0	0	0	0	2	0	0	0	0	0	0	0
833	Vacuolar protein sorting-associated protein 4A OS=Homo sapiens OX=9606 GN=VPS4A PE=1 SV=1	VPS4A_HUMAN	VPS4A	49 kDa	0	0	0	0	0	3	0	0	0	0	0	0
