# Supplementary material for: Proteome of Stored RBC Membrane and Vesicles from Heterozygous Beta Thalassemia Donors
Source: Int J Mol Sci. 2021 Mar 25;22(7):3369. doi: 10.3390/ijms22073369 (PMC8037027; doi:10.3390/ijms22073369)
Supplement: Supplementary file 1 [file ijms-22-03369-s001.zip › Table S3 - Proteomics analysis of extracellular vesicles.pdf]

					Total Spectrum Count									
#	Identified Proteins (657)	Accession Number	Alternate ID	Molecular Weight	Control					beta-Thalassemia Heterozygotes				
1	Hemoglobin subunit alpha OS=Homo sapiens OX=9606 GN=HBA1 PE=1 SV=2	HBA_HUMAN	HBA1	15 kDa	1136	1019	1171	975	1324	1095	1149	1375	1324	994
2	Hemoglobin subunit beta OS=Homo sapiens OX=9606 GN=HBB PE=1 SV=2	HBB_HUMAN	HBB	16 kDa	989	898	990	857	1154	942	939	1047	1023	924
3	Serum albumin OS=Homo sapiens OX=9606 GN=ALB PE=1 SV=2	ALBU_HUMAN	ALB	69 kDa	495	370	648	559	512	472	231	663	569	344
4	Erythrocyte band 7 integral membrane protein OS=Homo sapiens OX=9606 GN=STOM PE=1 SV=3	STOM_HUMAN	STOM	32 kDa	286	230	404	318	463	274	274	587	402	235
5	Band 3 anion transport protein OS=Homo sapiens OX=9606 GN=SLC4A1 PE=1 SV=3	B3AT_HUMAN	SLC4A1	102 kDa	226	207	302	194	298	213	211	492	382	185
6	Ankyrin-1 OS=Homo sapiens OX=9606 GN=ANK1 PE=1 SV=3	ANK1_HUMAN	ANK1	206 kDa	232	233	230	193	194	223	247	268	255	194
7	Transitional endoplasmic reticulum ATPase OS=Homo sapiens OX=9606 GN=VCP PE=1 SV=4	TERA_HUMAN	VCP	89 kDa	183	173	202	187	193	180	175	220	190	182
8	Carbonic anhydrase 1 OS=Homo sapiens OX=9606 GN=CA1 PE=1 SV=2	CAH1_HUMAN	CA1	29 kDa	140	119	174	135	115	171	194	301	155	143
9	Peroxiredoxin-2 OS=Homo sapiens OX=9606 GN=PRDX2 PE=1 SV=5	PRDX2_HUMAN	PRDX2	22 kDa	87	78	190	120	167	136	107	246	227	87
10	Hemoglobin subunit delta OS=Homo sapiens OX=9606 GN=HBD PE=1 SV=2	HBD_HUMAN	HBD	16 kDa	538	502	533	484	579	558	547	561	544	532
11	Serotransferrin OS=Homo sapiens OX=9606 GN=TF PE=1 SV=3	TRFE_HUMAN	TF	77 kDa	119	99	152	113	87	139	68	123	82	137
12	Catalase OS=Homo sapiens OX=9606 GN=CAT PE=1 SV=3	CATA_HUMAN	CAT	60 kDa	95	87	97	88	159	108	96	143	134	107
13	Protein 4.1 OS=Homo sapiens OX=9606 GN=EPB41 PE=1 SV=4	41_HUMAN	EPB41	97 kDa	68	83	88	67	91	65	62	112	136	57
14	Hemoglobin subunit gamma-1 OS=Homo sapiens OX=9606 GN=HBG1 PE=1 SV=2	HBG1_HUMAN	HBG1	16 kDa	197	160	165	287	246	239	268	253	218	208
15	Flavin reductase (NADPH) OS=Homo sapiens OX=9606 GN=BLVRB PE=1 SV=3	BLVRB_HUMAN	BLVRB	22 kDa	76	65	93	78	78	96	107	149	87	87
16	Erythrocyte membrane protein band 4.2 OS=Homo sapiens OX=9606 GN=EPB42 PE=1 SV=3	EPB42_HUMAN	EPB42	77 kDa	59	58	79	33	80	49	50	136	97	44
17	Immunoglobulin heavy constant mu OS=Homo sapiens OX=9606 GN=IGHM PE=1 SV=4	IGHM_HUMAN	IGHM	49 kDa	87	72	65	111	46	70	112	52	33	78
18	Carbonic anhydrase 2 OS=Homo sapiens OX=9606 GN=CA2 PE=1 SV=2	CAH2_HUMAN	CA2	29 kDa	75	62	82	65	74	82	75	103	77	70
19	Complement C3 OS=Homo sapiens OX=9606 GN=C3 PE=1 SV=2	CO3_HUMAN	C3	187 kDa	114	99	121	47	36	37	18	66	49	49
20	Solute carrier family 2, facilitated glucose transporter member 1 OS=Homo sapiens OX=9606 GN=SLC2A1 PE=1 SV=2	GTR1_HUMAN	SLC2A1	54 kDa	37	37	57	32	110	40	38	141	126	30
21	Keratin, type I cytoskeletal 10 OS=Homo sapiens OX=9606 GN=KRT10 PE=1 SV=6	K1C10_HUMAN	KRT10	59 kDa	123	55	47	69	22	78	73	52	66	68
22	Keratin, type I cytoskeletal 9 OS=Homo sapiens OX=9606 GN=KRT9 PE=1 SV=3	K1C9_HUMAN	KRT9	62 kDa	68	59	40	55	27	69	74	51	102	72
23	55 kDa erythrocyte membrane protein OS=Homo sapiens OX=9606 GN=MPP1 PE=1 SV=2	EM55_HUMAN	MPP1	52 kDa	62	61	68	52	58	58	57	71	88	49
24	Keratin, type II cytoskeletal 1 OS=Homo sapiens OX=9606 GN=KRT1 PE=1 SV=6	K2C1_HUMAN	KRT1	66 kDa	85	67	50	51	22	72	82	47	88	77
25	Heat shock cognate 71 kDa protein OS=Homo sapiens OX=9606 GN=HSPA8 PE=1 SV=1	HSP7C_HUMAN	HSPA8	71 kDa	49	64	59	58	50	57	75	63	51	54
26	Methanethiol oxidase OS=Homo sapiens OX=9606 GN=SELENBP1 PE=1 SV=2	SBP1_HUMAN	SELENBP1	52 kDa	44	42	48	42	58	57	50	46	56	59
27	Fibrinogen gamma chain OS=Homo sapiens OX=9606 GN=FGG PE=1 SV=3	FIBG_HUMAN	FGG	52 kDa	57	60	27	30	23	37	38	18	27	90
28	Immunoglobulin gamma-1 heavy chain OS=Homo sapiens OX=9606 PE=1 SV=2	IGG1_HUMAN		49 kDa	62	48	61	62	34	48	43	40	46	39
29	Peroxiredoxin-6 OS=Homo sapiens OX=9606 GN=PRDX6 PE=1 SV=3	PRDX6_HUMAN	PRDX6	25 kDa	46	37	56	39	64	50	53	33	67	47
30	Apolipoprotein B-100 OS=Homo sapiens OX=9606 GN=APOB PE=1 SV=2	APOB_HUMAN	APOB	516 kDa	77	67	64		28		8	87	68	2
31	Flotillin-1 OS=Homo sapiens OX=9606 GN=FLOT1 PE=1 SV=3	FLOT1_HUMAN	FLOT1	47 kDa	32	30	59	20	37	66	36	57	33	67
32	Spectrin alpha chain, erythrocytic 1 OS=Homo sapiens OX=9606 GN=SPTA1 PE=1 SV=5	SPTA1_HUMAN	SPTA1	280 kDa	62	50	22	9	27	46	40	50	26	79
33	Rab GDP dissociation inhibitor beta OS=Homo sapiens OX=9606 GN=GDI2 PE=1 SV=2	GDIB_HUMAN	GDI2	51 kDa	41	32	45	23	51	45	36	46	52	37
34	Heat shock 70 kDa protein 1A OS=Homo sapiens OX=9606 GN=HSPA1A PE=1 SV=1	HS71A_HUMAN (+1)	HSPA1A	70 kDa	30	46	39	30	24	72	72	50	29	54
35	Glyceraldehyde-3-phosphate dehydrogenase OS=Homo sapiens OX=9606 GN=GAPDH PE=1 SV=3	G3P_HUMAN	GAPDH	36 kDa	27	30	38	30	44	37	38	45	45	40
36	Alpha-1-antitrypsin OS=Homo sapiens OX=9606 GN=SERPINA1 PE=1 SV=3	A1AT_HUMAN	SERPINA1	47 kDa	29	37	58	43	41	31	24	47	39	31
37	Apolipoprotein A-I OS=Homo sapiens OX=9606 GN=APOA1 PE=1 SV=1	APOA1_HUMAN	APOA1	31 kDa	53	44	45	45	29	38	31	36	32	39
38	Ras-related protein Rap-1A OS=Homo sapiens OX=9606 GN=RAP1A PE=1 SV=1	RAP1A_HUMAN	RAP1A	21 kDa	24	24	34	27	25	41	36	90	23	37
39	Haptoglobin OS=Homo sapiens OX=9606 GN=HP PE=1 SV=1	HPT_HUMAN	HP	45 kDa	38	31	51	36	23	37	20	22	39	32
40	Acetylcholinesterase OS=Homo sapiens OX=9606 GN=ACHE PE=1 SV=1	ACES_HUMAN	ACHE	68 kDa	26	30	35	34	46	27	21	38	29	21
41	Keratin, type II cytoskeletal 2 epidermal OS=Homo sapiens OX=9606 GN=KRT2 PE=1 SV=2	K22E_HUMAN	KRT2	65 kDa	72	31	29	37	5	36	37	18	27	40
42	Immunoglobulin heavy constant alpha 1 OS=Homo sapiens OX=9606 GN=IGHA1 PE=1 SV=2	IGHA1_HUMAN	IGHA1	38 kDa	39	43	28	37	18	23	42	23	36	30
43	Purine nucleoside phosphorylase OS=Homo sapiens OX=9606 GN=PNP PE=1 SV=2	PNPH_HUMAN	PNP	32 kDa	22	27	43	27	38	29	42	41	45	32
44	Hemopexin OS=Homo sapiens OX=9606 GN=HPX PE=1 SV=2	HEMO_HUMAN	HPX	52 kDa	35	29	44	32	26	27	15	31	24	29
45	Delta-aminolevulinic acid dehydratase OS=Homo sapiens OX=9606 GN=ALAD PE=1 SV=1	HEM2_HUMAN	ALAD	36 kDa	11	21	29	19	29	35	16	38	38	25
46	Moesin OS=Homo sapiens OX=9606 GN=MSN PE=1 SV=3	MOES_HUMAN	MSN	68 kDa	38	30	26	28	12	33	35	28	18	26
47	Complement C4-B OS=Homo sapiens OX=9606 GN=C4B PE=1 SV=2	CO4B_HUMAN	C4B	193 kDa	37	28	48	30	20	14	23	30	22	26
48	Actin, cytoplasmic 2 OS=Homo sapiens OX=9606 GN=ACTG1 PE=1 SV=1	ACTG_HUMAN	ACTG1	42 kDa	30	41	25	11	17	34	39	29	12	29
49	Phosphoglycerate kinase 1 OS=Homo sapiens OX=9606 GN=PGK1 PE=1 SV=3	PGK1_HUMAN	PGK1	45 kDa	20	26	23	26	21	30	31	27	22	22
50	Alpha-2-macroglobulin OS=Homo sapiens OX=9606 GN=A2M PE=1 SV=3	A2MG_HUMAN	A2M	163 kDa	69	49	56	16	15	7	6	17	20	9
51	Alpha-soluble NSF attachment protein OS=Homo sapiens OX=9606 GN=NAPA PE=1 SV=3	SNAA_HUMAN	NAPA	33 kDa	22	18	30	20	36	36	17	44	26	31
52	Spectrin beta chain, erythrocytic OS=Homo sapiens OX=9606 GN=SPTB PE=1 SV=5	SPTB1_HUMAN	SPTB	246 kDa	26	24	19	2	28	24	12	33	15	39
53	Flotillin-2 OS=Homo sapiens OX=9606 GN=FLOT2 PE=1 SV=2	FLOT2_HUMAN	FLOT2	47 kDa	16	19	33	15	36	37	19	46	30	37
54	ADP-ribosylation factor 1 OS=Homo sapiens OX=9606 GN=ARF1 PE=1 SV=2	ARF1_HUMAN	ARF1	21 kDa	23	25	30	22	40	19	20	39	38	22
55	Peptidyl-prolyl cis-trans isomerase A OS=Homo sapiens OX=9606 GN=PPIA PE=1 SV=2	PPIA_HUMAN	PPIA	18 kDa	27	30	29	19	28	35	26	34	28	23
56	Heat shock protein HSP 90-alpha OS=Homo sapiens OX=9606 GN=HSP90AA1 PE=1 SV=5	HS90A_HUMAN	HSP90AA1	85 kDa	16	22	21	23	14	40	41	26	11	38
57	Triosephosphate isomerase OS=Homo sapiens OX=9606 GN=TPI1 PE=1 SV=3	TPIS_HUMAN	TPI1	31 kDa	22	23	25	26	20	29	29	22	25	32
58	Semaphorin-7A OS=Homo sapiens OX=9606 GN=SEMA7A PE=1 SV=1	SEM7A_HUMAN	SEMA7A	75 kDa	28	17	28	27	24	32	11	22	19	28
59	Immunoglobulin heavy constant gamma 2 OS=Homo sapiens OX=9606 GN=IGHG2 PE=1 SV=2	IGHG2_HUMAN	IGHG2	36 kDa	68	42	67	59	19	46	35	39	23	41
60	Guanine nucleotide-binding protein G(I)/G(S)/G(T) subunit beta-1 OS=Homo sapiens OX=9606 GN=GNB1 PE=1 SV=3	GBB1_HUMAN	GNB1	37 kDa	21	21	22	16	28	23	25	38	21	27
61	Vesicle-fusing ATPase OS=Homo sapiens OX=9606 GN=NSF PE=1 SV=3	NSF_HUMAN	NSF	83 kDa	15	20	36	19	26	19	7	29	20	12
62	Fructose-bisphosphate aldolase A OS=Homo sapiens OX=9606 GN=ALDOA PE=1 SV=2	ALDOA_HUMAN	ALDOA	39 kDa	22	27	24	22	19	30	31	21	18	26
63	14-3-3 protein epsilon OS=Homo sapiens OX=9606 GN=YWHAE PE=1 SV=1	1433E_HUMAN	YWHAE	29 kDa	16	17	15	19	23	31	20	21	26	28
64	Immunoglobulin kappa light chain OS=Homo sapiens OX=9606 PE=1 SV=1	IGK_HUMAN		23 kDa	31	25	25	32	22	17	22	29	26	23
65	Retinal dehydrogenase 1 OS=Homo sapiens OX=9606 GN=ALDH1A1 PE=1 SV=2	AL1A1_HUMAN	ALDH1A1	55 kDa	20	19	24	22	21	27	20	24	23	21
66	Programmed cell death 6-interacting protein OS=Homo sapiens OX=9606 GN=PDCD6IP PE=1 SV=1	PDC6I_HUMAN	PDCD6IP	96 kDa	16	4	32	8	22	34	8	21	11	36
67	L-lactate dehydrogenase B chain OS=Homo sapiens OX=9606 GN=LDHB PE=1 SV=2	LDHB_HUMAN	LDHB	37 kDa	15	18	15	12	33	21	14	26	29	10
68	Beta-2-glycoprotein 1 OS=Homo sapiens OX=9606 GN=APOH PE=1 SV=3	APOH_HUMAN	APOH	38 kDa	22	22	23	22	29	17	15	15	14	15
69	Guanine nucleotide-binding protein G(i) subunit alpha-2 OS=Homo sapiens OX=9606 GN=GNAI2 PE=1 SV=3	GNAI2_HUMAN	GNAI2	40 kDa	11	18	28	13	35	19	12	26	23	19
70	Glycophorin-A OS=Homo sapiens OX=9606 GN=GYPA PE=1 SV=2	GLPA_HUMAN	GYPA	16 kDa	22	18	16	15	16	18	21	17	23	20
71	Disheveled-associated activator of morphogenesis 1 OS=Homo sapiens OX=9606 GN=DAAM1 PE=1 SV=2	DAAM1_HUMAN	DAAM1	123 kDa	10	15	24	8	33	19	10	33	26	18
72	Ras-related protein Rap-2b OS=Homo sapiens OX=9606 GN=RAP2B PE=1 SV=1	RAP2B_HUMAN	RAP2B	21 kDa	16	20	22	18	17	20	16	33	20	17
73	Annexin A7 OS=Homo sapiens OX=9606 GN=ANXA7 PE=1 SV=3	ANXA7_HUMAN	ANXA7	53 kDa	26	12	17	20	11	42	12	17	8	28
74	Bisphosphoglycerate mutase OS=Homo sapiens OX=9606 GN=BPGM PE=1 SV=2	PMGE_HUMAN	BPGM	30 kDa	17	22	18	21	22	21	17	25	24	15
75	UBX domain-containing protein 6 OS=Homo sapiens OX=9606 GN=UBXN6 PE=1 SV=1	UBXN6_HUMAN	UBXN6	50 kDa	9	10	28	14	18	17	20	29	8	34
76	Radixin OS=Homo sapiens OX=9606 GN=RDX PE=1 SV=1	RADI_HUMAN	RDX	69 kDa	29	21	24	29	25	21	30	31	31	25
77	UV excision repair protein RAD23 homolog A OS=Homo sapiens OX=9606 GN=RAD23A PE=1 SV=1	RD23A_HUMAN	RAD23A	40 kDa	6	8	19	13	29	12	13	30	31	11
78	Stress-induced-phosphoprotein 1 OS=Homo sapiens OX=9606 GN=STIP1 PE=1 SV=1	STIP1_HUMAN	STIP1	63 kDa	12	19	16	18	12	22	26	16	12	25
79	Eukaryotic translation initiation factor 5A-1 OS=Homo sapiens OX=9606 GN=EIF5A PE=1 SV=2	IF5A1_HUMAN	EIF5A	17 kDa	13	14	17	13	20	20	19	29	22	14
80	Transferrin receptor protein 1 OS=Homo sapiens OX=9606 GN=TFRC PE=1 SV=2	TFR1_HUMAN	TFRC	85 kDa	12		5			37	23	63		43
81	Cell division control protein 42 homolog OS=Homo sapiens OX=9606 GN=CDC42 PE=1 SV=2	CDC42_HUMAN	CDC42	21 kDa	14	17	19	14	27	19	17	18	20	17
82	Blood group Rh(CE) polypeptide OS=Homo sapiens OX=9606 GN=RHCE PE=1 SV=2	RHCE_HUMAN	RHCE	46 kDa	13	17	20	9	26	16	16	24	32	11
83	Adenylate kinase isoenzyme 1 OS=Homo sapiens OX=9606 GN=AK1 PE=1 SV=3	KAD1_HUMAN	AK1	22 kDa	15	18	18	17	20	21	17	13	21	14
84	Complement decay-accelerating factor OS=Homo sapiens OX=9606 GN=CD55 PE=1 SV=4	DAF_HUMAN	CD55	41 kDa	16	19	19	18	18	22	17	21	17	13
85	CD59 glycoprotein OS=Homo sapiens OX=9606 GN=CD59 PE=1 SV=1	CD59_HUMAN	CD59	14 kDa	23	20	21	20	16	18	14	3	14	25
86	Guanine nucleotide-binding protein G(s) subunit alpha isoforms XLas OS=Homo sapiens OX=9606 GN=GNAS PE=1 SV=2	GNAS1_HUMAN	GNAS	111 kDa	16	15	15	15	24	19	13	25	15	12
87	Ammonium transporter Rh type A OS=Homo sapiens OX=9606 GN=RHAG PE=1 SV=2	RHAG_HUMAN	RHAG	44 kDa	16	19	16	16	9	14	17	31	15	12
88	RuvB-like 2 OS=Homo sapiens OX=9606 GN=RUVBL2 PE=1 SV=3	RUVB2_HUMAN	RUVBL2	51 kDa	10	9	18	9	9	21	30	15	9	20
89	Phosphatidylinositol 5-phosphate 4-kinase type-2 alpha OS=Homo sapiens OX=9606 GN=PIP4K2A PE=1 SV=2	PI42A_HUMAN	PIP4K2A	46 kDa	8	12	8	12	24	17	16	24	16	15
90	C4b-binding protein alpha chain OS=Homo sapiens OX=9606 GN=C4BPA PE=1 SV=2	C4BPA_HUMAN	C4BPA	67 kDa	17	14	20	29	3	9	30	5		26
91	Vitamin D-binding protein OS=Homo sapiens OX=9606 GN=GC PE=1 SV=2	VTDB_HUMAN	GC	53 kDa	18	15	15	20	14	17	9	9	14	22
92	Keratin, type I cytoskeletal 14 OS=Homo sapiens OX=9606 GN=KRT14 PE=1 SV=4	K1C14_HUMAN	KRT14	52 kDa	39	16	5	12	3	19	19	4	16	19
93	Ras-related protein Rab-1B OS=Homo sapiens OX=9606 GN=RAB1B PE=1 SV=1	RAB1B_HUMAN	RAB1B	22 kDa	13	16	22	20	15	20	17	32	15	23
94	Ceruloplasmin OS=Homo sapiens OX=9606 GN=CP PE=1 SV=1	CERU_HUMAN	CP	122 kDa	21	22	32	12	6	7	4	6	9	14
95	Basal cell adhesion molecule OS=Homo sapiens OX=9606 GN=BCAM PE=1 SV=2	BCAM_HUMAN	BCAM	67 kDa	14	9	22	2	9	23	5	22	26	22
96	Superoxide dismutase [Cu-Zn] OS=Homo sapiens OX=9606 GN=SOD1 PE=1 SV=2	SODC_HUMAN	SOD1	16 kDa	21	20	13	18	14	15	17	11	12	18
97	Peroxiredoxin-1 OS=Homo sapiens OX=9606 GN=PRDX1 PE=1 SV=1	PRDX1_HUMAN	PRDX1	22 kDa	17	23	16	19	22	24	18	17	16	23
98	Alpha-1B-glycoprotein OS=Homo sapiens OX=9606 GN=A1BG PE=1 SV=4	A1BG_HUMAN	A1BG	54 kDa	19	15	30	14	11	14	5	8	6	13
99	Acylamino-acid-releasing enzyme OS=Homo sapiens OX=9606 GN=APEH PE=1 SV=4	ACPH_HUMAN	APEH	81 kDa	12	13	10	10	20	13	10	11	16	9

100	Protein/nucleic acid deglycase DJ-1 OS=Homo sapiens OX=9606 GN=PARK7 PE=1 SV=2	PARK7_HUMAN	PARK7	20 kDa	15	12	14	13	15	14	14	15	13	13
101	Golgi-associated plant pathogenesis-related protein 1 OS=Homo sapiens OX=9606 GN=GLIPR2 PE=1 SV=3	GAPR1_HUMAN	GLIPR2	17 kDa	12	11	13	11	16	16	9	13	10	15
102	Alpha-enolase OS=Homo sapiens OX=9606 GN=ENO1 PE=1 SV=2	ENOA_HUMAN	ENO1	47 kDa	10	8	17	10	7	12	17	12	9	16
103	Porphobilinogen deaminase OS=Homo sapiens OX=9606 GN=HMBS PE=1 SV=2	HEM3_HUMAN	HMBS	39 kDa	8	2	11	10	8	21	14	18	6	21
104	Cofilin-1 OS=Homo sapiens OX=9606 GN=CFL1 PE=1 SV=3	COF1_HUMAN	CFL1	19 kDa	15	14	12	8	16	13	14	17	8	15
105	2',3'-cyclic-nucleotide 3'-phosphodiesterase OS=Homo sapiens OX=9606 GN=CNP PE=1 SV=2	CN37_HUMAN	CNP	48 kDa	13	10	21	14	23	14	8	9	16	8
106	Fibrinogen alpha chain OS=Homo sapiens OX=9606 GN=FGA PE=1 SV=2	FIBA_HUMAN	FGA	95 kDa	16	21	21	11	4	5	8	5	7	17
107	Alpha-1-acid glycoprotein 2 OS=Homo sapiens OX=9606 GN=ORM2 PE=1 SV=2	A1AG2_HUMAN	ORM2	24 kDa	18	17	24	17	11	15	7	7	14	19
108	Transforming protein RhoA OS=Homo sapiens OX=9606 GN=RHOA PE=1 SV=1	RHOA_HUMAN	RHOA	22 kDa	13	13	8	15	11	14	11	14	10	18
109	Alpha-2-HS-glycoprotein OS=Homo sapiens OX=9606 GN=AHSG PE=1 SV=2	FETUA_HUMAN	AHSG	39 kDa	17	11	16	6	9	11	8	16	11	13
110	Ribonuclease inhibitor OS=Homo sapiens OX=9606 GN=RNH1 PE=1 SV=2	RINI_HUMAN	RNH1	50 kDa	12	11	14	12	16	12	10	15	13	10
111	Serine/threonine-protein kinase TAO3 OS=Homo sapiens OX=9606 GN=TAOK3 PE=1 SV=2	TAOK3_HUMAN	TAOK3	105 kDa	8	5	13	5	10	19	11	17	8	22
112	Long-chain-fatty-acid--CoA ligase 4 OS=Homo sapiens OX=9606 GN=ACSL4 PE=1 SV=2	ACSL4_HUMAN	ACSL4	79 kDa	11	8	12	11	17	16	8	15	19	9
113	EH domain-containing protein 1 OS=Homo sapiens OX=9606 GN=EHD1 PE=1 SV=2	EHD1_HUMAN	EHD1	61 kDa	4	5	12	5	8	14	9	15	5	21
114	Calpastatin OS=Homo sapiens OX=9606 GN=CAST PE=1 SV=4	ICAL_HUMAN	CAST	77 kDa	9	9	15	12	7	16	14	7	7	27
115	Hsc70-interacting protein OS=Homo sapiens OX=9606 GN=ST13 PE=1 SV=2	F10A1_HUMAN	ST13	41 kDa	14	15	9	13	6	11	18	12	11	17
116	CB1 cannabinoid receptor-interacting protein 1 OS=Homo sapiens OX=9606 GN=CNRIP1 PE=1 SV=1	CNRP1_HUMAN	CNRIP1	19 kDa	11	12	11	12	16	7	12	19	17	9
117	Immunoglobulin kappa constant OS=Homo sapiens OX=9606 GN=IGKC PE=1 SV=2	IGKC_HUMAN	IGKC	12 kDa	40	34	39	43		27	30		38	
118	Antithrombin-III OS=Homo sapiens OX=9606 GN=SERPINC1 PE=1 SV=1	ANT3_HUMAN	SERPINC1	53 kDa	18	16	12	16	4	12	3	14	8	9
119	14-3-3 protein zeta/delta OS=Homo sapiens OX=9606 GN=YWHAZ PE=1 SV=1	1433Z_HUMAN	YWHAZ	28 kDa	20	21	11	12	14	20	22	12	16	19
120	Guanine nucleotide-binding protein G(k) subunit alpha OS=Homo sapiens OX=9606 GN=GNAI3 PE=1 SV=3	GNAI3_HUMAN	GNAI3	41 kDa	16	19	27	22	34	23	15	34	22	14
121	Immunoglobulin lambda-1 light chain OS=Homo sapiens OX=9606 PE=1 SV=1	IGL1_HUMAN (+1)		23 kDa	18	13	10	17	7	11	12	7	9	6
122	Talin-1 OS=Homo sapiens OX=9606 GN=TLN1 PE=1 SV=3	TLN1_HUMAN	TLN1	270 kDa	6	44	4			7	17	14	7	6
123	Alpha-synuclein OS=Homo sapiens OX=9606 GN=SNCA PE=1 SV=1	SYUA_HUMAN	SNCA	14 kDa	15	13	13	15	10	19	14	7	11	15
124	EH domain-binding protein 1-like protein 1 OS=Homo sapiens OX=9606 GN=EHBP1L1 PE=1 SV=2	EH1L1_HUMAN	EHBP1L1	162 kDa	5		12	9	16	7	8	9	12	15
125	Sorcin OS=Homo sapiens OX=9606 GN=SRI PE=1 SV=1	SORCN_HUMAN	SRI	22 kDa	9	6	12	9	10	29	6	17		19
126	Fibrinogen beta chain OS=Homo sapiens OX=9606 GN=FGB PE=1 SV=2	FIBB_HUMAN	FGB	56 kDa	20	21	14	15	4	6	12	5	5	11
127	Clathrin heavy chain 1 OS=Homo sapiens OX=9606 GN=CLTC PE=1 SV=5	CLH1_HUMAN	CLTC	192 kDa	16	3	24	2	43	3		9		10
128	Guanine nucleotide-binding protein G(I)/G(S)/G(T) subunit beta-2 OS=Homo sapiens OX=9606 GN=GNB2 PE=1 SV=3	GBB2_HUMAN	GNB2	37 kDa	20		22		14	32	25	24	12	29
129	CD5 antigen-like OS=Homo sapiens OX=9606 GN=CD5L PE=1 SV=1	CD5L_HUMAN	CD5L	38 kDa	16	13	13	24		16	15	2		14
130	Nucleoside diphosphate kinase A OS=Homo sapiens OX=9606 GN=NME1 PE=1 SV=1	NDKA_HUMAN	NME1	17 kDa	8	9	14	10	9	11	13	4	9	11
131	RuvB-like 1 OS=Homo sapiens OX=9606 GN=RUVBL1 PE=1 SV=1	RUVB1_HUMAN	RUVBL1	50 kDa	7	9	9	7	4	9	23	15	10	16
132	Ubiquitin carboxyl-terminal hydrolase 14 OS=Homo sapiens OX=9606 GN=USP14 PE=1 SV=3	UBP14_HUMAN	USP14	56 kDa	11	9	7	6	14	12	10	13	11	14
133	Hypoxanthine-guanine phosphoribosyltransferase OS=Homo sapiens OX=9606 GN=HPRT1 PE=1 SV=2	HPRT_HUMAN	HPRT1	25 kDa	10	9	11	12	11	15	13	9	12	11
134	Inter-alpha-trypsin inhibitor heavy chain H4 OS=Homo sapiens OX=9606 GN=ITIH4 PE=1 SV=4	ITIH4_HUMAN	ITIH4	103 kDa	17	12	11	8	8	12	16	15	5	5
135	Protein argonaute-2 OS=Homo sapiens OX=9606 GN=AGO2 PE=1 SV=3	AGO2_HUMAN	AGO2	97 kDa	8	3	8	4	5	11	4	10	13	10
136	Phosphatidylethanolamine-binding protein 1 OS=Homo sapiens OX=9606 GN=PEBP1 PE=1 SV=3	PEBP1_HUMAN	PEBP1	21 kDa	13	10	10	13	10	11	12	14	11	7
137	Protein-L-isoaspartate(D-aspartate) O-methyltransferase OS=Homo sapiens OX=9606 GN=PCMT1 PE=1 SV=4	PIMT_HUMAN	PCMT1	25 kDa	17	11	10	15	8	17	15	7	8	13
138	Heme-binding protein 1 OS=Homo sapiens OX=9606 GN=HEBP1 PE=1 SV=1	HEBP1_HUMAN	HEBP1	21 kDa	6	8	14	10	10	15	8	11	8	18
139	Galectin-3 OS=Homo sapiens OX=9606 GN=LGALS3 PE=1 SV=5	LEG3_HUMAN	LGALS3	26 kDa	10	7	15	9	10	21	10	6	3	15
140	Synaptic vesicle membrane protein VAT-1 homolog OS=Homo sapiens OX=9606 GN=VAT1 PE=1 SV=2	VAT1_HUMAN	VAT1	42 kDa	4	4	10		27	9	8	20	2	12
141	Carbonic anhydrase 3 OS=Homo sapiens OX=9606 GN=CA3 PE=1 SV=3	CAH3_HUMAN	CA3	30 kDa	19	5	6	8		4	16	6	7	6
142	Charged multivesicular body protein 4b OS=Homo sapiens OX=9606 GN=CHMP4B PE=1 SV=1	CHM4B_HUMAN	CHMP4B	25 kDa	11	8	8	14	5	18	9	11	3	23
143	Complement factor H OS=Homo sapiens OX=9606 GN=CFH PE=1 SV=4	CFAH_HUMAN	CFH	139 kDa	12	7	8			3			4	
144	Protein DDI1 homolog 2 OS=Homo sapiens OX=9606 GN=DDI2 PE=1 SV=1	DDI2_HUMAN	DDI2	45 kDa	7	10	11	11	10	9	12	9	10	11
145	Malate dehydrogenase, cytoplasmic OS=Homo sapiens OX=9606 GN=MDH1 PE=1 SV=4	MDHC_HUMAN	MDH1	36 kDa	15	10	11	16	8	12	12	12	7	15
146	GTP-binding nuclear protein Ran OS=Homo sapiens OX=9606 GN=RAN PE=1 SV=3	RAN_HUMAN	RAN	24 kDa	8	13	8	10	7	11	14	6	4	9
147	GTPase KRas OS=Homo sapiens OX=9606 GN=KRAS PE=1 SV=1	RASK_HUMAN	KRAS	22 kDa	8	7	11	8	14	11	7	15	7	8
148	Apolipoprotein A-II OS=Homo sapiens OX=9606 GN=APOA2 PE=1 SV=1	APOA2_HUMAN	APOA2	11 kDa	15	13	11	13	9	13		9	7	6
149	Keratin, type II cytoskeletal 6A OS=Homo sapiens OX=9606 GN=KRT6A PE=1 SV=3	K2C6A_HUMAN	KRT6A	60 kDa	25	14	10	14		13	19	6	16	26
150	Aspartate aminotransferase, cytoplasmic OS=Homo sapiens OX=9606 GN=GOT1 PE=1 SV=3	AATC_HUMAN	GOT1	46 kDa	8	8	8	4	5	13	13	9	6	10
151	Complement factor B OS=Homo sapiens OX=9606 GN=CFB PE=1 SV=2	CFAB_HUMAN	CFB	86 kDa	14	6	20	6	4	9	5	13	5	8
152	Ras-related C3 botulinum toxin substrate 1 OS=Homo sapiens OX=9606 GN=RAC1 PE=1 SV=1	RAC1_HUMAN	RAC1	21 kDa	8	5	9	9	22	8	12	12	14	9
153	Thioredoxin OS=Homo sapiens OX=9606 GN=TXN PE=1 SV=3	THIO_HUMAN	TXN	12 kDa	11	9	8	10	9	10	11	16	7	7
154	Calmodulin-1 OS=Homo sapiens OX=9606 GN=CALM1 PE=1 SV=1	CALM1_HUMAN (+2)	CALM1	17 kDa	4	6	9	10	3	10	5	11		20
155	Alpha-1-acid glycoprotein 1 OS=Homo sapiens OX=9606 GN=ORM1 PE=1 SV=1	A1AG1_HUMAN	ORM1	24 kDa	20	21	23	15	9	15	6	8	13	22
156	Obg-like ATPase 1 OS=Homo sapiens OX=9606 GN=OLA1 PE=1 SV=2	OLA1_HUMAN	OLA1	45 kDa	5	5	6	4	5	14	12	9	5	8
157	Ras-related protein Rab-35 OS=Homo sapiens OX=9606 GN=RAB35 PE=1 SV=1	RAB35_HUMAN	RAB35	23 kDa	8	7	18	10	17	9	12	8	12	12
158	ATP-citrate synthase OS=Homo sapiens OX=9606 GN=ACLY PE=1 SV=3	ACLY_HUMAN	ACLY	121 kDa	3	4	7	3	13	17	5	17	8	9
159	Calpain small subunit 1 OS=Homo sapiens OX=9606 GN=CAPNS1 PE=1 SV=1	CPNS1_HUMAN	CAPNS1	28 kDa	10	7	7	6	6	11	12	9	10	9
160	Protein diaphanous homolog 1 OS=Homo sapiens OX=9606 GN=DIAPH1 PE=1 SV=2	DIAP1_HUMAN	DIAPH1	141 kDa	8	8	13	5	19	5	5	9	8	3
161	Glutathione S-transferase omega-1 OS=Homo sapiens OX=9606 GN=GSTO1 PE=1 SV=2	GSTO1_HUMAN	GSTO1	28 kDa	6	8	7	7	10	7	7	9	7	10
162	Alpha-hemoglobin-stabilizing protein OS=Homo sapiens OX=9606 GN=AHSP PE=1 SV=1	AHSP_HUMAN	AHSP	12 kDa	11	8	4	9	3	13	4	8		23
163	Ubiquitin-conjugating enzyme E2 N OS=Homo sapiens OX=9606 GN=UBE2N PE=1 SV=1	UBE2N_HUMAN	UBE2N	17 kDa	9	9	6	9	4	10	9	6	8	12
164	Serine/threonine-protein phosphatase 2A 65 kDa regulatory subunit A alpha isoform OS=Homo sapiens OX=9606 GN=PPP2R1A PE=1 SV=4	2AAA_HUMAN	PPP2R1A	65 kDa	3	2	7		8	10	5	7	12	8
165	Elongation factor 1-alpha 1 OS=Homo sapiens OX=9606 GN=EEF1A1 PE=1 SV=1	EF1A1_HUMAN	EEF1A1	50 kDa	6	6	8	4	7	8	4	11	3	14
166	Protein-glutamine gamma-glutamyltransferase 2 OS=Homo sapiens OX=9606 GN=TGM2 PE=1 SV=2	TGM2_HUMAN	TGM2	77 kDa		7	9	7	6	8	6	15	9	9
167	AP-2 complex subunit beta OS=Homo sapiens OX=9606 GN=AP2B1 PE=1 SV=1	AP2B1_HUMAN	AP2B1	105 kDa	3	2	6		6	5	6	12	9	11
168	Clusterin OS=Homo sapiens OX=9606 GN=CLU PE=1 SV=1	CLUS_HUMAN	CLU	52 kDa	11	9	9	9	3	11	7	5	4	10
169	Fibronectin OS=Homo sapiens OX=9606 GN=FN1 PE=1 SV=4	FINC_HUMAN	FN1	263 kDa		48	7	3	3			2	2	
170	Proliferation-associated protein 2G4 OS=Homo sapiens OX=9606 GN=PA2G4 PE=1 SV=3	PA2G4_HUMAN	PA2G4	44 kDa	3	6	11	4	3	13	7	8	9	9
171	Small integral membrane protein 1 OS=Homo sapiens OX=9606 GN=SMIM1 PE=1 SV=1	SMIM1_HUMAN	SMIM1	9 kDa	9	10	8		11	14	10	7		16
172	Protein XRP2 OS=Homo sapiens OX=9606 GN=RP2 PE=1 SV=4	XRP2_HUMAN	RP2	40 kDa	6	8	12	9	7	5	5	10	13	5
173	S-formylglutathione hydrolase OS=Homo sapiens OX=9606 GN=ESD PE=1 SV=2	ESTD_HUMAN	ESD	31 kDa	9	8	7	3	8	6	7	6	5	10
174	6-phosphogluconate dehydrogenase, decarboxylating OS=Homo sapiens OX=9606 GN=PGD PE=1 SV=3	6PGD_HUMAN	PGD	53 kDa	5	6	9	4	9	5	9	10	9	4
175	Ribose-phosphate pyrophosphokinase 1 OS=Homo sapiens OX=9606 GN=PRPS1 PE=1 SV=2	PRPS1_HUMAN	PRPS1	35 kDa	12	8	8	9	9	8	7	3	8	6
176	Filamin-A OS=Homo sapiens OX=9606 GN=FLNA PE=1 SV=4	FLNA_HUMAN	FLNA	281 kDa	6	36					15			
177	Adenosylhomocysteinase OS=Homo sapiens OX=9606 GN=AHCY PE=1 SV=4	SAHH_HUMAN	AHCY	48 kDa	9	11	7	10	8	11	6	5	6	9
178	Proteasome activator complex subunit 2 OS=Homo sapiens OX=9606 GN=PSME2 PE=1 SV=4	PSME2_HUMAN	PSME2	27 kDa		3	8		9	7	6	10	10	5
179	Apolipoprotein E OS=Homo sapiens OX=9606 GN=APOE PE=1 SV=1	APOE_HUMAN	APOE	36 kDa	13	7	7	10	4	7	11	10	4	4
180	Ubiquitin-like modifier-activating enzyme 1 OS=Homo sapiens OX=9606 GN=UBA1 PE=1 SV=3	UBA1_HUMAN	UBA1	118 kDa	4	2	8		15	5	6	12	14	8
181	14-3-3 protein beta/alpha OS=Homo sapiens OX=9606 GN=YWHAB PE=1 SV=3	1433B_HUMAN	YWHAB	28 kDa	10	10	8	10	12	12	14	15	12	12
182	Ubiquitin carboxyl-terminal hydrolase 5 OS=Homo sapiens OX=9606 GN=USP5 PE=1 SV=2	UBP5_HUMAN	USP5	96 kDa	6	3	7	4	9	11	6	3	6	5
183	Coagulation factor V OS=Homo sapiens OX=9606 GN=F5 PE=1 SV=4	FA5_HUMAN	F5	252 kDa	13	24	8	13			4	2	7	
184	Lactoylglutathione lyase OS=Homo sapiens OX=9606 GN=GLO1 PE=1 SV=4	LGUL_HUMAN	GLO1	21 kDa			10		5	2	5	8	5	6
185	Prothrombin OS=Homo sapiens OX=9606 GN=F2 PE=1 SV=2	THRB_HUMAN	F2	70 kDa	13	14	15	6	2	3		10	5	4
186	Elongation factor 2 OS=Homo sapiens OX=9606 GN=EEF2 PE=1 SV=4	EF2_HUMAN	EEF2	95 kDa			3		5	11	3	19		11
187	Hemoglobin subunit theta-1 OS=Homo sapiens OX=9606 GN=HBQ1 PE=1 SV=2	HBAT_HUMAN	HBQ1	16 kDa	7	4	9	9	12	8	7	9	8	5
188	Transketolase OS=Homo sapiens OX=9606 GN=TKT PE=1 SV=3	TKT_HUMAN	TKT	68 kDa	11	5	12	5	5	4	6	4	4	10
189	Guanine nucleotide-binding protein G(q) subunit alpha OS=Homo sapiens OX=9606 GN=GNAQ PE=1 SV=4	GNAQ_HUMAN	GNAQ	42 kDa	3	7	7	3	6	10	4	9		8
190	Ficolin-2 OS=Homo sapiens OX=9606 GN=FCN2 PE=1 SV=2	FCN2_HUMAN	FCN2	34 kDa		9	12	5		14	16		4	9
191	TBC1 domain family member 24 OS=Homo sapiens OX=9606 GN=TBC1D24 PE=1 SV=2	TBC24_HUMAN	TBC1D24	63 kDa	8	6	8	5	5	4	7	3	8	
192	Band 4.1-like protein 2 OS=Homo sapiens OX=9606 GN=EPB41L2 PE=1 SV=1	E41L2_HUMAN	EPB41L2	113 kDa	3	3	15	2	4		9	4	4	6
193	Vitronectin OS=Homo sapiens OX=9606 GN=VTN PE=1 SV=1	VTNC_HUMAN	VTN	54 kDa	7	6	8	6		4	2	7	8	5
194	Polyubiquitin-B OS=Homo sapiens OX=9606 GN=UBB PE=1 SV=1	UBB_HUMAN (+1)	UBB	26 kDa	8	7	5	8	3	11	7	4	3	11
195	Histidine triad nucleotide-binding protein 1 OS=Homo sapiens OX=9606 GN=HINT1 PE=1 SV=2	HINT1_HUMAN	HINT1	14 kDa		3		6	3	6	5	6	2	8
196	Alpha-1-antichymotrypsin OS=Homo sapiens OX=9606 GN=SERPINA3 PE=1 SV=2	AACT_HUMAN	SERPINA3	48 kDa	9	7	11	5	4	9	2	7	8	7
197	Proteasome activator complex subunit 1 OS=Homo sapiens OX=9606 GN=PSME1 PE=1 SV=1	PSME1_HUMAN	PSME1	29 kDa	4	6	9	6	3	6	6	6	5	4
198	Ras-related protein Ral-A OS=Homo sapiens OX=9606 GN=RALA PE=1 SV=1	RALA_HUMAN	RALA	24 kDa	4	5	6	4	7	6	6	7	7	6
199	Keratin, type I cytoskeletal 16 OS=Homo sapiens OX=9606 GN=KRT16 PE=1 SV=4	K1C16_HUMAN	KRT16	51 kDa	13	21		10		14	10	4	12	17
200	T-complex protein 1 subunit beta OS=Homo sapiens OX=9606 GN=CCT2 PE=1 SV=4	TCPB_HUMAN	CCT2	57 kDa	7	2	5		3	15	5	8		12

201	Proteasome subunit alpha type-5 OS=Homo sapiens OX=9606 GN=PSMA5 PE=1 SV=3	PSA5_HUMAN	PSMA5	26 kDa	9	7	5	6	5	12	11	3	6	11
202	Apolipoprotein A-IV OS=Homo sapiens OX=9606 GN=APOA4 PE=1 SV=3	APOA4_HUMAN	APOA4	45 kDa	7	8	11	8	4	7	4	4	2	5
203	Immunoglobulin heavy constant gamma 3 OS=Homo sapiens OX=9606 GN=IGHG3 PE=1 SV=2	IGHG3_HUMAN	IGHG3	41 kDa	42	25	37	36	10	24	25	14	14	18
204	Serine/threonine-protein kinase OSR1 OS=Homo sapiens OX=9606 GN=OXSR1 PE=1 SV=1	OXSR1_HUMAN	OXSR1	58 kDa	3	4	8		10	8	7	9	10	2
205	cAMP-dependent protein kinase type I-alpha regulatory subunit OS=Homo sapiens OX=9606 GN=PRKAR1A PE=1 SV=1	KAP0_HUMAN	PRKAR1A	43 kDa		3	2		4	9	8	3	6	7
206	Basigin OS=Homo sapiens OX=9606 GN=BSG PE=1 SV=2	BASI_HUMAN	BSG	42 kDa	4	7		4		5	7	14	3	10
207	T-complex protein 1 subunit zeta OS=Homo sapiens OX=9606 GN=CCT6A PE=1 SV=3	TCPZ_HUMAN	CCT6A	58 kDa	5		8	3	2	10	6	10		9
208	Ubiquitin-conjugating enzyme E2 L3 OS=Homo sapiens OX=9606 GN=UBE2L3 PE=1 SV=1	UB2L3_HUMAN	UBE2L3	18 kDa	3	3	6	6	7	4	4	3	8	7
209	Ras-related protein Rab-11B OS=Homo sapiens OX=9606 GN=RAB11B PE=1 SV=4	RB11B_HUMAN	RAB11B	24 kDa	6		5	4	4	9	7	3	2	16
210	Cullin-associated NEDD8-dissociated protein 1 OS=Homo sapiens OX=9606 GN=CAND1 PE=1 SV=2	CAND1_HUMAN	CAND1	136 kDa		5	10	2	10	2		10	9	
211	Glutathione S-transferase P OS=Homo sapiens OX=9606 GN=GSTP1 PE=1 SV=2	GSTP1_HUMAN	GSTP1	23 kDa	4	5	3		9	4	3	9	15	8
212	Nucleosome assembly protein 1-like 4 OS=Homo sapiens OX=9606 GN=NAP1L4 PE=1 SV=1	NP1L4_HUMAN	NAP1L4	43 kDa		3	7		3	10	8	12		9
213	Kell blood group glycoprotein OS=Homo sapiens OX=9606 GN=KEL PE=1 SV=2	KELL_HUMAN	KEL	83 kDa	4	3	8		7	4	3	7	9	2
214	Complement C1q subcomponent subunit B OS=Homo sapiens OX=9606 GN=C1QB PE=1 SV=3	C1QB_HUMAN	C1QB	27 kDa	5		4	32			6			8
215	CD44 antigen OS=Homo sapiens OX=9606 GN=CD44 PE=1 SV=3	CD44_HUMAN	CD44	82 kDa	7	6	6	6	5	8	10	6	6	9
216	Uroporphyrinogen decarboxylase OS=Homo sapiens OX=9606 GN=UROD PE=1 SV=2	DCUP_HUMAN	UROD	41 kDa	4	3	5	2	7	6	3	7	8	5
217	Ribose-5-phosphate isomerase OS=Homo sapiens OX=9606 GN=RPIA PE=1 SV=3	RPIA_HUMAN	RPIA	33 kDa	4	7	5	5	6	8	3	11	4	10
218	GMP reductase 1 OS=Homo sapiens OX=9606 GN=GMPR PE=1 SV=1	GMPR1_HUMAN	GMPR	37 kDa	2	3	4	3	8	6	4	9	4	5
219	Protein S100-A4 OS=Homo sapiens OX=9606 GN=S100A4 PE=1 SV=1	S10A4_HUMAN	S100A4	12 kDa	6	6	7	7	5	6	7	5	5	7
220	Nucleoside diphosphate kinase B OS=Homo sapiens OX=9606 GN=NME2 PE=1 SV=1	NDKB_HUMAN	NME2	17 kDa			13		10	14	11	7	14	
221	Vacuolar protein sorting-associated protein VTA1 homolog OS=Homo sapiens OX=9606 GN=VTA1 PE=1 SV=1	VTA1_HUMAN	VTA1	34 kDa	5		7	5	3	12	4	8	2	10
222	Programmed cell death protein 6 OS=Homo sapiens OX=9606 GN=PDCD6 PE=1 SV=1	PDCD6_HUMAN	PDCD6	22 kDa	5		5	6	2	15		6		13
223	Immunoglobulin J chain OS=Homo sapiens OX=9606 GN=JCHAIN PE=1 SV=4	IGJ_HUMAN	JCHAIN	18 kDa	9	6	6	13	2	6	12	4		8
224	Hydroxyacylglutathione hydrolase, mitochondrial OS=Homo sapiens OX=9606 GN=HAGH PE=1 SV=2	GLO2_HUMAN	HAGH	34 kDa	6	7	7	7	5	5	5	4	6	9
225	Ecto-ADP-ribosyltransferase 4 OS=Homo sapiens OX=9606 GN=ART4 PE=2 SV=2	NAR4_HUMAN	ART4	36 kDa	4	4	5	6	4	6	6	8	6	8
226	Transaldolase OS=Homo sapiens OX=9606 GN=TALDO1 PE=1 SV=2	TALDO_HUMAN	TALDO1	38 kDa		3	7	5	8	12	5	5	5	4
227	Pyruvate kinase PKLR OS=Homo sapiens OX=9606 GN=PKLR PE=1 SV=2	KPYR_HUMAN	PKLR	62 kDa	2	2	7		6	10	2	8		7
228	Zinc-alpha-2-glycoprotein OS=Homo sapiens OX=9606 GN=AZGP1 PE=1 SV=2	ZA2G_HUMAN	AZGP1	34 kDa	10	6	3	7	5		4	4	5	5
229	Proteasome subunit alpha type-2 OS=Homo sapiens OX=9606 GN=PSMA2 PE=1 SV=2	PSA2_HUMAN	PSMA2	26 kDa	4	2	4		5	8	6	11	8	3
230	Protein S100-A6 OS=Homo sapiens OX=9606 GN=S100A6 PE=1 SV=1	S10A6_HUMAN	S100A6	10 kDa	4	2	3	3	4		3	5	7	2
231	Acetyl-CoA acetyltransferase, cytosolic OS=Homo sapiens OX=9606 GN=ACAT2 PE=1 SV=2	THIC_HUMAN	ACAT2	41 kDa	2		5		7	5	3	4	12	4
232	Hemoglobin subunit zeta OS=Homo sapiens OX=9606 GN=HBZ PE=1 SV=2	HBAZ_HUMAN	HBZ	16 kDa	10	3		3	9	3	17	3	16	
233	Lymphocyte function-associated antigen 3 OS=Homo sapiens OX=9606 GN=CD58 PE=1 SV=1	LFA3_HUMAN	CD58	28 kDa	5	4	4	6	9	5	3	10	5	5
234	Glutamate--cysteine ligase catalytic subunit OS=Homo sapiens OX=9606 GN=GCLC PE=1 SV=2	GSH1_HUMAN	GCLC	73 kDa	5	4	3	3	9	6	2	7	7	3
235	Transthyretin OS=Homo sapiens OX=9606 GN=TTR PE=1 SV=1	TTHY_HUMAN	TTR	16 kDa	6	8	4	9	2	9	3	9	7	9
236	Inter-alpha-trypsin inhibitor heavy chain H2 OS=Homo sapiens OX=9606 GN=ITIH2 PE=1 SV=2	ITIH2_HUMAN	ITIH2	106 kDa	10	12	4	9		4	2	2	6	2
237	T-complex protein 1 subunit theta OS=Homo sapiens OX=9606 GN=CCT8 PE=1 SV=4	TCPQ_HUMAN	CCT8	60 kDa	5	3	6		3	12	4	5		6
238	Calpain-1 catalytic subunit OS=Homo sapiens OX=9606 GN=CAPN1 PE=1 SV=1	CAN1_HUMAN	CAPN1	82 kDa	5	4	2		7	2	5	8	4	6
239	T-complex protein 1 subunit epsilon OS=Homo sapiens OX=9606 GN=CCT5 PE=1 SV=1	TCPE_HUMAN	CCT5	60 kDa	3		4		6	9	5	14		7
240	Plasma membrane calcium-transporting ATPase 4 OS=Homo sapiens OX=9606 GN=ATP2B4 PE=1 SV=2	AT2B4_HUMAN	ATP2B4	138 kDa	5	5	4	3	6	10	5	7	4	4
241	Platelet-activating factor acetylhydrolase IB subunit gamma OS=Homo sapiens OX=9606 GN=PAFAH1B3 PE=1 SV=1	PA1B3_HUMAN	PAFAH1B3	26 kDa	3	5	6	2	4	5	6	5	5	5
242	Adenylosuccinate lyase OS=Homo sapiens OX=9606 GN=ADSL PE=1 SV=2	PUR8_HUMAN	ADSL	55 kDa	3	2	6	2	7	5	3	9	8	2
243	D-dopachrome decarboxylase OS=Homo sapiens OX=9606 GN=DDT PE=1 SV=3	DOPD_HUMAN	DDT	13 kDa	5	6	6	7	6	5	4	8	8	6
244	Plasma protease C1 inhibitor OS=Homo sapiens OX=9606 GN=SERPING1 PE=1 SV=2	IC1_HUMAN	SERPING1	55 kDa	5	7	9	5		7		3	6	5
245	Integrin alpha-IIb OS=Homo sapiens OX=9606 GN=ITGA2B PE=1 SV=3	ITA2B_HUMAN	ITGA2B	113 kDa	7	19					10		3	
246	Plasminogen OS=Homo sapiens OX=9606 GN=PLG PE=1 SV=2	PLMN_HUMAN	PLG	91 kDa	3	7	8			2		3		5
247	PITH domain-containing protein 1 OS=Homo sapiens OX=9606 GN=PITHD1 PE=1 SV=1	PITH1_HUMAN	PITHD1	24 kDa	2	5	6	8	3	5	4	3	3	7
248	Proteasome subunit alpha type-7 OS=Homo sapiens OX=9606 GN=PSMA7 PE=1 SV=1	PSA7_HUMAN	PSMA7	28 kDa	3	3	9	3	4	2	8		4	7
249	Ras-related protein Rab-7a OS=Homo sapiens OX=9606 GN=RAB7A PE=1 SV=1	RAB7A_HUMAN	RAB7A	23 kDa	3		3	2	2	13	5	7		10
250	Low molecular weight phosphotyrosine protein phosphatase OS=Homo sapiens OX=9606 GN=ACP1 PE=1 SV=3	PPAC_HUMAN	ACP1	18 kDa	5	4	7	2		9	9	5	4	9
251	Angiotensinogen OS=Homo sapiens OX=9606 GN=AGT PE=1 SV=1	ANGT_HUMAN	AGT	53 kDa	7	3	10	8	4	6		4	4	2
252	Arginase-1 OS=Homo sapiens OX=9606 GN=ARG1 PE=1 SV=2	ARGI1_HUMAN	ARG1	35 kDa	3	2	3	2		4	7	9	12	5
253	Guanine nucleotide-binding protein subunit alpha-13 OS=Homo sapiens OX=9606 GN=GNA13 PE=1 SV=2	GNA13_HUMAN	GNA13	44 kDa	4		5		4	8	5	7	3	16
254	Immunoglobulin heavy constant gamma 4 OS=Homo sapiens OX=9606 GN=IGHG4 PE=1 SV=1	IGHG4_HUMAN	IGHG4	36 kDa		43	59					22	22	
255	Keratin, type II cytoskeletal 5 OS=Homo sapiens OX=9606 GN=KRT5 PE=1 SV=3	K2C5_HUMAN	KRT5	62 kDa	28	16	10	11		17	17		14	27
256	Tubulin beta-4B chain OS=Homo sapiens OX=9606 GN=TUBB4B PE=1 SV=1	TBB4B_HUMAN	TUBB4B	50 kDa	3		2		2	9		3		13
257	Syntaxin-7 OS=Homo sapiens OX=9606 GN=STX7 PE=1 SV=4	STX7_HUMAN	STX7	30 kDa	4	5	8	5	4	6	5	9	3	6
258	L-lactate dehydrogenase A chain OS=Homo sapiens OX=9606 GN=LDHA PE=1 SV=2	LDHA_HUMAN	LDHA	37 kDa	5		2	2	7	6	4	4	6	4
259	Ras-related protein Rab-14 OS=Homo sapiens OX=9606 GN=RAB14 PE=1 SV=4	RAB14_HUMAN	RAB14	24 kDa			5		2	7	7	12	3	9
260	Actin, alpha cardiac muscle 1 OS=Homo sapiens OX=9606 GN=ACTC1 PE=1 SV=1	ACTC_HUMAN (+1)	ACTC1	42 kDa			12							
261	Histidine-rich glycoprotein OS=Homo sapiens OX=9606 GN=HRG PE=1 SV=1	HRG_HUMAN	HRG	60 kDa	11	5		5			5	6		5
262	Tubulin alpha-1B chain OS=Homo sapiens OX=9606 GN=TUBA1B PE=1 SV=1	TBA1B_HUMAN	TUBA1B	50 kDa		3	7		2	3		12		17
263	Heat shock 70 kDa protein 4 OS=Homo sapiens OX=9606 GN=HSPA4 PE=1 SV=4	HSP74_HUMAN	HSPA4	94 kDa	5	2	6	2	6	7	4	5	5	3
264	Glutathione peroxidase 1 OS=Homo sapiens OX=9606 GN=GPX1 PE=1 SV=4	GPX1_HUMAN	GPX1	22 kDa	6	2	5	5		7	3	5	7	7
265	IST1 homolog OS=Homo sapiens OX=9606 GN=IST1 PE=1 SV=1	IST1_HUMAN	IST1	40 kDa	5	2	5	5		11	5			11
266	Ras-related protein Rab-5C OS=Homo sapiens OX=9606 GN=RAB5C PE=1 SV=2	RAB5C_HUMAN	RAB5C	23 kDa	5	5	4	5	7	3	3	6	5	5
267	Phosphoglycerate mutase 1 OS=Homo sapiens OX=9606 GN=PGAM1 PE=1 SV=2	PGAM1_HUMAN	PGAM1	29 kDa	5	3	2	3	2	5	2	4	4	3
268	Biliverdin reductase A OS=Homo sapiens OX=9606 GN=BLVRA PE=1 SV=2	BIEA_HUMAN	BLVRA	33 kDa	2	2	6	2	8	3		8	7	4
269	Kininogen-1 OS=Homo sapiens OX=9606 GN=KNG1 PE=1 SV=2	KNG1_HUMAN	KNG1	72 kDa	7	8	6	7	2	4		5	3	3
270	Rho GDP-dissociation inhibitor 1 OS=Homo sapiens OX=9606 GN=ARHGDIA PE=1 SV=3	GDIR1_HUMAN	ARHGDIA	23 kDa	3	4	5	2	5	7	4	2	5	5
271	Glutamate--cysteine ligase regulatory subunit OS=Homo sapiens OX=9606 GN=GCLM PE=1 SV=1	GSH0_HUMAN	GCLM	31 kDa	5	6	5	5	3	6	3			6
272	GDP-L-fucose synthase OS=Homo sapiens OX=9606 GN=TSTA3 PE=1 SV=1	FCL_HUMAN	TSTA3	36 kDa	2	2	5	3	3	4	4		2	4
273	Leucine-rich repeat-containing protein 57 OS=Homo sapiens OX=9606 GN=LRRC57 PE=1 SV=1	LRC57_HUMAN	LRRC57	27 kDa	2		3	3	4	6	3	6	5	3
274	Uncharacterized protein C1orf198 OS=Homo sapiens OX=9606 GN=C1orf198 PE=1 SV=1	CA198_HUMAN	C1orf198	36 kDa	2	2	5	3	4	5	3	6		5
275	Proteasome inhibitor PI31 subunit OS=Homo sapiens OX=9606 GN=PSMF1 PE=1 SV=2	PSMF1_HUMAN	PSMF1	30 kDa	5	5	5	6	5	4	4	3	2	4
276	F-box only protein 7 OS=Homo sapiens OX=9606 GN=FBXO7 PE=1 SV=1	FBX7_HUMAN	FBXO7	59 kDa	4	4	4	3	4	4	3	4	4	5
277	Myotrophin OS=Homo sapiens OX=9606 GN=MTPN PE=1 SV=2	MTPN_HUMAN	MTPN	13 kDa	6		4	3	4	5	5	3	6	
278	26S proteasome non-ATPase regulatory subunit 2 OS=Homo sapiens OX=9606 GN=PSMD2 PE=1 SV=3	PSMD2_HUMAN	PSMD2	100 kDa	3	4	5		6	5		4	7	3
279	T-complex protein 1 subunit delta OS=Homo sapiens OX=9606 GN=CCT4 PE=1 SV=4	TCPD_HUMAN	CCT4	58 kDa	3	2	3	3	4	10	4	6		6
280	Keratin, type II cuticular Hb6 OS=Homo sapiens OX=9606 GN=KRT86 PE=1 SV=1	KRT86_HUMAN	KRT86	53 kDa	34			2						
281	3-mercaptopyruvate sulfurtransferase OS=Homo sapiens OX=9606 GN=MPST PE=1 SV=3	THTM_HUMAN	MPST	33 kDa	5		6		3	5	5	2	2	4
282	Serine/threonine-protein phosphatase 2A activator OS=Homo sapiens OX=9606 GN=PTPA PE=1 SV=3	PTPA_HUMAN	PTPA	41 kDa	3		5		4	3		4	3	4
283	Glutaredoxin-1 OS=Homo sapiens OX=9606 GN=GLRX PE=1 SV=2	GLRX1_HUMAN	GLRX	12 kDa	6		4	2	3		3	7	6	6
284	Serum paraoxonase/arylesterase 1 OS=Homo sapiens OX=9606 GN=PON1 PE=1 SV=3	PON1_HUMAN	PON1	40 kDa	7	6	7	3		2		7		
285	Charged multivesicular body protein 6 OS=Homo sapiens OX=9606 GN=CHMP6 PE=1 SV=3	CHMP6_HUMAN	CHMP6	23 kDa	3	4	5	4	8	5	3	2	5	6
286	Axin interactor, dorsalization-associated protein OS=Homo sapiens OX=9606 GN=AIDA PE=1 SV=1	AIDA_HUMAN	AIDA	35 kDa		5	5	3	4	3	7	7	4	4
287	Proteasome subunit beta type-4 OS=Homo sapiens OX=9606 GN=PSMB4 PE=1 SV=4	PSB4_HUMAN	PSMB4	29 kDa	4	3	3	3		3	6	2	2	4
288	Hemoglobin subunit mu OS=Homo sapiens OX=9606 GN=HBM PE=1 SV=1	HBM_HUMAN	HBM	16 kDa			3	2	7	6	3	7	3	7
289	Osteoclast-stimulating factor 1 OS=Homo sapiens OX=9606 GN=OSTF1 PE=1 SV=2	OSTF1_HUMAN	OSTF1	24 kDa	3	3	4	2	5	6	3	2	3	6
290	14-3-3 protein theta OS=Homo sapiens OX=9606 GN=YWHAQ PE=1 SV=1	1433T_HUMAN	YWHAQ	28 kDa			8			11	7	9		11
291	Aquaporin-1 OS=Homo sapiens OX=9606 GN=AQP1 PE=1 SV=3	AQP1_HUMAN	AQP1	29 kDa				2	9	3	3	8	8	
292	Thioredoxin-like protein 1 OS=Homo sapiens OX=9606 GN=TXNL1 PE=1 SV=3	TXNL1_HUMAN	TXNL1	32 kDa	4	3	6		2	6	5	2		11
293	Calcium-binding protein 39 OS=Homo sapiens OX=9606 GN=CAB39 PE=1 SV=1	CAB39_HUMAN	CAB39	40 kDa			2	3	3	4			2	5
294	Bifunctional purine biosynthesis protein PURH OS=Homo sapiens OX=9606 GN=ATIC PE=1 SV=3	PUR9_HUMAN	ATIC	65 kDa			4		7	5	5	3	3	3
295	Nicotinate phosphoribosyltransferase OS=Homo sapiens OX=9606 GN=NAPRT PE=1 SV=2	PNCB_HUMAN	NAPRT	58 kDa	2	5	3			4		6	2	
296	NSFL1 cofactor p47 OS=Homo sapiens OX=9606 GN=NSFL1C PE=1 SV=2	NSF1C_HUMAN	NSFL1C	41 kDa		6	3	2	2	9	5	3		3
297	Gamma-soluble NSF attachment protein OS=Homo sapiens OX=9606 GN=NAPG PE=1 SV=1	SNAG_HUMAN	NAPG	35 kDa	6	3	7	3	2	8	3			8
298	ATP-binding cassette sub-family B member 6, mitochondrial OS=Homo sapiens OX=9606 GN=ABCB6 PE=1 SV=1	ABCB6_HUMAN	ABCB6	94 kDa	2		6		5	3	3	8	4	7
299	Copper chaperone for superoxide dismutase OS=Homo sapiens OX=9606 GN=CCS PE=1 SV=1	CCS_HUMAN	CCS	29 kDa	3	3			3	3		4	3	3
300	Fumarate hydratase, mitochondrial OS=Homo sapiens OX=9606 GN=FH PE=1 SV=3	FUMH_HUMAN	FH	55 kDa	2		2			5	4	4	3	4
301	Ras-related protein Rab-10 OS=Homo sapiens OX=9606 GN=RAB10 PE=1 SV=1	RAB10_HUMAN	RAB10	23 kDa	7	8	14	7		10	9	10		10

302	T-complex protein 1 subunit alpha OS=Homo sapiens OX=9606 GN=TCP1 PE=1 SV=1	TCPA_HUMAN	TCP1	60 kDa	2		6			9				5
303	Afamin OS=Homo sapiens OX=9606 GN=AFM PE=1 SV=1	AFAM_HUMAN	AFM	69 kDa	2	4	9	3				6		
304	Nuclear transport factor 2 OS=Homo sapiens OX=9606 GN=NUTF2 PE=1 SV=1	NTF2_HUMAN	NUTF2	14 kDa	2	4	4	4	6	5	2	4	5	3
305	Ras-related protein Rap-1b OS=Homo sapiens OX=9606 GN=RAP1B PE=1 SV=1	RAP1B_HUMAN	RAP1B	21 kDa		22	30	24	24	36	31	77		33
306	Junctional adhesion molecule A OS=Homo sapiens OX=9606 GN=F11R PE=1 SV=1	JAM1_HUMAN	F11R	33 kDa			2	3	5	3		3	6	
307	Proteasome subunit beta type-2 OS=Homo sapiens OX=9606 GN=PSMB2 PE=1 SV=1	PSB2_HUMAN	PSMB2	23 kDa	2			2	3	3	2		2	
308	Charged multivesicular body protein 4a OS=Homo sapiens OX=9606 GN=CHMP4A PE=1 SV=3	CHM4A_HUMAN	CHMP4A	25 kDa			4			9	3			11
309	F-actin-capping protein subunit beta OS=Homo sapiens OX=9606 GN=CAPZB PE=1 SV=4	CAPZB_HUMAN	CAPZB	31 kDa	3	3	2	4		7	3	3		6
310	Proteasome subunit beta type-1 OS=Homo sapiens OX=9606 GN=PSMB1 PE=1 SV=2	PSB1_HUMAN	PSMB1	26 kDa		2	4		6	3	3		5	
311	F-actin-capping protein subunit alpha-1 OS=Homo sapiens OX=9606 GN=CAPZA1 PE=1 SV=3	CAZA1_HUMAN	CAPZA1	33 kDa	3		2	2		4	3	3		8
312	Rho GTPase-activating protein 1 OS=Homo sapiens OX=9606 GN=ARHGAP1 PE=1 SV=1	RHG01_HUMAN	ARHGAP1	50 kDa			4		2	9	3	4		10
313	DnaJ homolog subfamily B member 2 OS=Homo sapiens OX=9606 GN=DNAJB2 PE=1 SV=3	DNJB2_HUMAN	DNAJB2	36 kDa	2	6	7	6		3	3			4
314	Ubiquitin-conjugating enzyme E2 variant 2 OS=Homo sapiens OX=9606 GN=UBE2V2 PE=1 SV=4	UB2V2_HUMAN	UBE2V2	16 kDa		2	4	2	4	4	4			6
315	Fermitin family homolog 3 OS=Homo sapiens OX=9606 GN=FERMT3 PE=1 SV=1	URP2_HUMAN	FERMT3	76 kDa	2	11					2	5		2
316	Proteasome subunit alpha type-4 OS=Homo sapiens OX=9606 GN=PSMA4 PE=1 SV=1	PSA4_HUMAN	PSMA4	29 kDa	2	2		2	5	4	2	7	4	3
317	Ubiquitin thioesterase OTU1 OS=Homo sapiens OX=9606 GN=YOD1 PE=1 SV=1	OTU1_HUMAN	YOD1	38 kDa			3	3	4	4	3	4	6	2
318	Equilibrative nucleoside transporter 1 OS=Homo sapiens OX=9606 GN=SLC29A1 PE=1 SV=3	S29A1_HUMAN	SLC29A1	50 kDa	4	4	5		6	3		5	7	3
319	Nectin-1 OS=Homo sapiens OX=9606 GN=NECTIN1 PE=1 SV=3	NECT1_HUMAN	NECTIN1	57 kDa	4	3	2		7	3		3	6	3
320	26S proteasome regulatory subunit 6A OS=Homo sapiens OX=9606 GN=PSMC3 PE=1 SV=3	PRS6A_HUMAN	PSMC3	49 kDa			5		5	2		4	8	6
321	Importin subunit beta-1 OS=Homo sapiens OX=9606 GN=KPNB1 PE=1 SV=2	IMB1_HUMAN	KPNB1	97 kDa			4		4	2			2	6
322	NADH-cytochrome b5 reductase 3 OS=Homo sapiens OX=9606 GN=CYB5R3 PE=1 SV=3	NB5R3_HUMAN	CYB5R3	34 kDa	3	3	3		7		3		3	2
323	Calpain-5 OS=Homo sapiens OX=9606 GN=CAPN5 PE=1 SV=2	CAN5_HUMAN	CAPN5	73 kDa	5	3			8			2		
324	Fumarylacetoacetase OS=Homo sapiens OX=9606 GN=FAH PE=1 SV=2	FAAA_HUMAN	FAH	46 kDa		2	6		3			6	5	4
325	Vacuolar protein sorting-associated protein 4A OS=Homo sapiens OX=9606 GN=VPS4A PE=1 SV=1	VPS4A_HUMAN	VPS4A	49 kDa						4	2			15
326	Proteasome subunit alpha type-6 OS=Homo sapiens OX=9606 GN=PSMA6 PE=1 SV=1	PSA6_HUMAN	PSMA6	27 kDa	2		3	3	3	4	3		6	6
327	Ubiquitin-associated domain-containing protein 1 OS=Homo sapiens OX=9606 GN=UBAC1 PE=1 SV=1	UBAC1_HUMAN	UBAC1	45 kDa			7	3	4	2		3		5
328	Malignant T-cell-amplified sequence 1 OS=Homo sapiens OX=9606 GN=MCTS1 PE=1 SV=1	MCTS1_HUMAN	MCTS1	21 kDa			5		2	4		3	4	4
329	14 kDa phosphohistidine phosphatase OS=Homo sapiens OX=9606 GN=PHPT1 PE=1 SV=1	PHP14_HUMAN	PHPT1	14 kDa	2		4		3	5			6	4
330	Synaptosomal-associated protein 23 OS=Homo sapiens OX=9606 GN=SNAP23 PE=1 SV=1	SNP23_HUMAN	SNAP23	23 kDa			4		3	3	2	3	3	
331	BRO1 domain-containing protein BROX OS=Homo sapiens OX=9606 GN=BROX PE=1 SV=1	BROX_HUMAN	BROX	46 kDa	3		3	2		7	3	3	4	6
332	Calcium and integrin-binding protein 1 OS=Homo sapiens OX=9606 GN=CIB1 PE=1 SV=4	CIB1_HUMAN	CIB1	22 kDa	5	2	5			6	2	2	2	9
333	Glyoxalase domain-containing protein 4 OS=Homo sapiens OX=9606 GN=GLOD4 PE=1 SV=1	GLOD4_HUMAN	GLOD4	35 kDa			4	2	4	5			5	3
334	Glutathione reductase, mitochondrial OS=Homo sapiens OX=9606 GN=GSR PE=1 SV=2	GSHR_HUMAN	GSR	56 kDa			3		4			5	8	2
335	Phosphatidylinositol transfer protein alpha isoform OS=Homo sapiens OX=9606 GN=PITPNA PE=1 SV=2	PIPNA_HUMAN	PITPNA	32 kDa		5	3	3		2	3			4
336	Solute carrier family 40 member 1 OS=Homo sapiens OX=9606 GN=SLC40A1 PE=1 SV=1	S40A1_HUMAN	SLC40A1	63 kDa	5	5	4	3		6			3	3
337	Proteasome subunit beta type-3 OS=Homo sapiens OX=9606 GN=PSMB3 PE=1 SV=2	PSB3_HUMAN	PSMB3	23 kDa		4		4		4	5		3	
338	Dipeptidyl peptidase 3 OS=Homo sapiens OX=9606 GN=DPP3 PE=1 SV=2	DPP3_HUMAN	DPP3	83 kDa						2	4	4	3	5
339	AP-2 complex subunit mu OS=Homo sapiens OX=9606 GN=AP2M1 PE=1 SV=2	AP2M1_HUMAN	AP2M1	50 kDa			3		3	4	3	7		3
340	Protein phosphatase 1 regulatory subunit 7 OS=Homo sapiens OX=9606 GN=PPP1R7 PE=1 SV=1	PP1R7_HUMAN	PPP1R7	42 kDa			5		4			5	4	4
341	Syntenin-1 OS=Homo sapiens OX=9606 GN=SDCBP PE=1 SV=1	SDCB1_HUMAN	SDCBP	32 kDa			4			2		12		6
342	Macrophage migration inhibitory factor OS=Homo sapiens OX=9606 GN=MIF PE=1 SV=4	MIF_HUMAN	MIF	12 kDa	3		4	2	3		2	3	4	
343	Haptoglobin-related protein OS=Homo sapiens OX=9606 GN=HPR PE=2 SV=2	HPTR_HUMAN	HPR	39 kDa	18	14	16	17		26	10	7		14
344	Proteasome subunit beta type-5 OS=Homo sapiens OX=9606 GN=PSMB5 PE=1 SV=3	PSB5_HUMAN	PSMB5	28 kDa	3	4	3	3	3	4				4
345	Monocarboxylate transporter 1 OS=Homo sapiens OX=9606 GN=SLC16A1 PE=1 SV=3	MOT1_HUMAN	SLC16A1	54 kDa			3			3		6		5
346	Tubulin-specific chaperone A OS=Homo sapiens OX=9606 GN=TBCA PE=1 SV=3	TBCA_HUMAN	TBCA	13 kDa	2				4	3	3			5
347	Calcium-regulated heat-stable protein 1 OS=Homo sapiens OX=9606 GN=CARHSP1 PE=1 SV=2	CHSP1_HUMAN	CARHSP1	16 kDa	2	2				5				6
348	Integrin beta-3 OS=Homo sapiens OX=9606 GN=ITGB3 PE=1 SV=2	ITB3_HUMAN	ITGB3	87 kDa	5	15					5			
349	Small VCP/p97-interacting protein OS=Homo sapiens OX=9606 GN=SVIP PE=1 SV=1	SVIP_HUMAN	SVIP	8 kDa	5	4	4	5	2	5	3		4	4
350	CD2-associated protein OS=Homo sapiens OX=9606 GN=CD2AP PE=1 SV=1	CD2AP_HUMAN	CD2AP	71 kDa						6		2		14
351	Ras-related protein Rab-2B OS=Homo sapiens OX=9606 GN=RAB2B PE=1 SV=1	RAB2B_HUMAN	RAB2B	24 kDa	4	2	4	3	3	3		4	5	2
352	Inter-alpha-trypsin inhibitor heavy chain H1 OS=Homo sapiens OX=9606 GN=ITIH1 PE=1 SV=3	ITIH1_HUMAN	ITIH1	101 kDa	4	3	7	3				2	2	3
353	Ubiquitin carboxyl-terminal hydrolase 15 OS=Homo sapiens OX=9606 GN=USP15 PE=1 SV=3	UBP15_HUMAN	USP15	112 kDa	3				4	4	3	5	3	4
354	Copper-transporting ATPase 1 OS=Homo sapiens OX=9606 GN=ATP7A PE=1 SV=4	ATP7A_HUMAN	ATP7A	163 kDa			2		9			3	4	
355	STAM-binding protein OS=Homo sapiens OX=9606 GN=STAMBP PE=1 SV=1	STABP_HUMAN	STAMBP	48 kDa			4			6		5		7
356	T-complex protein 1 subunit gamma OS=Homo sapiens OX=9606 GN=CCT3 PE=1 SV=4	TCPG_HUMAN	CCT3	61 kDa			3		6	4		6		
357	Peflin OS=Homo sapiens OX=9606 GN=PEF1 PE=1 SV=1	PEF1_HUMAN	PEF1	30 kDa			5	3	3	6				
358	Complement receptor type 1 OS=Homo sapiens OX=9606 GN=CR1 PE=1 SV=3	CR1_HUMAN	CR1	224 kDa	8		3			9		4		2
359	SH3 domain-binding protein 5-like OS=Homo sapiens OX=9606 GN=SH3BP5L PE=1 SV=1	3BP5L_HUMAN	SH3BP5L	43 kDa						5	4	3		9
360	Keratin, type I cuticular Ha1 OS=Homo sapiens OX=9606 GN=KRT31 PE=1 SV=3	K1H1_HUMAN	KRT31	47 kDa	25			4			5			
361	Glutaredoxin-3 OS=Homo sapiens OX=9606 GN=GLRX3 PE=1 SV=2	GLRX3_HUMAN	GLRX3	37 kDa	3	2	2	3	2	3	3			3
362	Guanine nucleotide-binding protein subunit beta-4 OS=Homo sapiens OX=9606 GN=GNB4 PE=1 SV=3	GBB4_HUMAN	GNB4	38 kDa	4	6	8		11	4	5	14	8	
363	Proteasome subunit alpha type-1 OS=Homo sapiens OX=9606 GN=PSMA1 PE=1 SV=1	PSA1_HUMAN	PSMA1	30 kDa		3		2	2	6		5		3
364	Heat shock protein HSP 90-beta OS=Homo sapiens OX=9606 GN=HSP90AB1 PE=1 SV=4	HS90B_HUMAN	HSP90AB1	83 kDa		12				18	17	12		17
365	S-phase kinase-associated protein 1 OS=Homo sapiens OX=9606 GN=SKP1 PE=1 SV=2	SKP1_HUMAN	SKP1	19 kDa	2	3	3	3				2	4	
366	Latexin OS=Homo sapiens OX=9606 GN=LXN PE=1 SV=2	LXN_HUMAN	LXN	26 kDa		4		3		4	3	6		5
367	Proteasome subunit alpha type-3 OS=Homo sapiens OX=9606 GN=PSMA3 PE=1 SV=2	PSA3_HUMAN	PSMA3	28 kDa	2	2	5	3		2	3	4		4
368	Erythroid membrane-associated protein OS=Homo sapiens OX=9606 GN=ERMAP PE=1 SV=1	ERMAP_HUMAN	ERMAP	53 kDa	4	2	2		3	2	3	5	2	3
369	Transport and Golgi organization protein 2 homolog OS=Homo sapiens OX=9606 GN=TANGO2 PE=1 SV=1	TNG2_HUMAN	TANGO2	31 kDa			2		5	3	2	4	3	
370	Ezrin OS=Homo sapiens OX=9606 GN=EZR PE=1 SV=4	EZRI_HUMAN	EZR	69 kDa	13	9					14	10		
371	Charged multivesicular body protein 2a OS=Homo sapiens OX=9606 GN=CHMP2A PE=1 SV=1	CHM2A_HUMAN	CHMP2A	25 kDa	2			2		4				10
372	Gelsolin OS=Homo sapiens OX=9606 GN=GSN PE=1 SV=1	GELS_HUMAN	GSN	86 kDa	8	9	2	2		4	2			
373	Fructosamine-3-kinase OS=Homo sapiens OX=9606 GN=FN3K PE=1 SV=1	FN3K_HUMAN	FN3K	35 kDa	2	4	3		3	3			3	3
374	Serine/threonine-protein phosphatase 2A catalytic subunit alpha isoform OS=Homo sapiens OX=9606 GN=PPP2CA PE=1 SV=1	PP2AA_HUMAN	PPP2CA	36 kDa			2		4	4	4	3	3	4
375	Immunoglobulin alpha-2 heavy chain OS=Homo sapiens OX=9606 PE=1 SV=2	IGA2_HUMAN		49 kDa		17	16	14		10	11			8
376	Carbonyl reductase [NADPH] 1 OS=Homo sapiens OX=9606 GN=CBR1 PE=1 SV=3	CBR1_HUMAN	CBR1	30 kDa			3	2		4	2		5	2
377	Eukaryotic initiation factor 4A-I OS=Homo sapiens OX=9606 GN=EIF4A1 PE=1 SV=1	IF4A1_HUMAN	EIF4A1	46 kDa			2			6				6
378	6-phosphogluconolactonase OS=Homo sapiens OX=9606 GN=PGLS PE=1 SV=2	6PGL_HUMAN	PGLS	28 kDa	2			2	2	2		2	3	2
379	2'-deoxynucleoside 5'-phosphate N-hydrolase 1 OS=Homo sapiens OX=9606 GN=DNPH1 PE=1 SV=1	DNPH1_HUMAN	DNPH1	19 kDa			2					3	3	
380	Nucleosome assembly protein 1-like 1 OS=Homo sapiens OX=9606 GN=NAP1L1 PE=1 SV=1	NP1L1_HUMAN	NAP1L1	45 kDa						6	7			7
381	14-3-3 protein gamma OS=Homo sapiens OX=9606 GN=YWHAG PE=1 SV=2	1433G_HUMAN	YWHAG	28 kDa	6		6		8		6	7	9	6
382	Small glutamine-rich tetratricopeptide repeat-containing protein alpha OS=Homo sapiens OX=9606 GN=SGTA PE=1 SV=1	SGTA_HUMAN	SGTA	34 kDa	2					6		3		5
383	CD99 antigen OS=Homo sapiens OX=9606 GN=CD99 PE=1 SV=1	CD99_HUMAN	CD99	19 kDa								4		
384	Exportin-7 OS=Homo sapiens OX=9606 GN=XPO7 PE=1 SV=3	XPO7_HUMAN	XPO7	124 kDa			3			2		4		4
385	Heparin cofactor 2 OS=Homo sapiens OX=9606 GN=SERPIND1 PE=1 SV=3	HEP2_HUMAN	SERPIND1	57 kDa			3	4	2				2	
386	Ras-related protein Rab-6A OS=Homo sapiens OX=9606 GN=RAB6A PE=1 SV=3	RAB6A_HUMAN	RAB6A	24 kDa		5		5		7	6			10
387	AP-2 complex subunit alpha-1 OS=Homo sapiens OX=9606 GN=AP2A1 PE=1 SV=3	AP2A1_HUMAN	AP2A1	108 kDa			3				2	6		2
388	Immunoglobulin kappa variable 4-1 OS=Homo sapiens OX=9606 GN=IGKV4-1 PE=1 SV=1	KV401_HUMAN	IGKV4-1	13 kDa	2	4	2	6		4	4	2		3
389	Protein TFG OS=Homo sapiens OX=9606 GN=TFG PE=1 SV=2	TFG_HUMAN	TFG	43 kDa			3	2	2	7				6
390	Ras-related protein Rab-1A OS=Homo sapiens OX=9606 GN=RAB1A PE=1 SV=3	RAB1A_HUMAN	RAB1A	23 kDa					12	16				18
391	SLIT-ROBO Rho GTPase-activating protein 2 OS=Homo sapiens OX=9606 GN=SRGAP2 PE=1 SV=3	SRGP2_HUMAN	SRGAP2	121 kDa		3	2			3		8		6
392	Complement component C9 OS=Homo sapiens OX=9606 GN=C9 PE=1 SV=2	CO9_HUMAN	C9	63 kDa	2	4	2	2	2					5
393	Glutathione synthetase OS=Homo sapiens OX=9606 GN=GSS PE=1 SV=1	GSHB_HUMAN	GSS	52 kDa	2	2		2	3	3			2	
394	Complement C1q subcomponent subunit A OS=Homo sapiens OX=9606 GN=C1QA PE=1 SV=2	C1QA_HUMAN	C1QA	26 kDa	3			8			3			3
395	Desmoplakin OS=Homo sapiens OX=9606 GN=DSP PE=1 SV=3	DESP_HUMAN	DSP	332 kDa	9								6	
396	Vinculin OS=Homo sapiens OX=9606 GN=VCL PE=1 SV=4	VINC_HUMAN	VCL	124 kDa	2	11					2			
397	Protein ABHD14B OS=Homo sapiens OX=9606 GN=ABHD14B PE=1 SV=1	ABHEB_HUMAN	ABHD14B	22 kDa			2		2	4	4		3	
398	Ran-specific GTPase-activating protein OS=Homo sapiens OX=9606 GN=RANBP1 PE=1 SV=1	RANG_HUMAN	RANBP1	23 kDa		2	3	2	3	3		4	5	3
399	Phosphoglucomutase-2 OS=Homo sapiens OX=9606 GN=PGM2 PE=1 SV=4	PGM2_HUMAN	PGM2	68 kDa	2		2		3			4	3	
400	Apolipoprotein D OS=Homo sapiens OX=9606 GN=APOD PE=1 SV=1	APOD_HUMAN	APOD	21 kDa	5	6		3		2				
401	Profilin-1 OS=Homo sapiens OX=9606 GN=PFN1 PE=1 SV=2	PROF1_HUMAN	PFN1	15 kDa	2	4				5	3	5	2	6
402	Rabankyrin-5 OS=Homo sapiens OX=9606 GN=ANKFY1 PE=1 SV=2	ANFY1_HUMAN	ANKFY1	128 kDa			5		7			3	4	

403	Apolipoprotein L1 OS=Homo sapiens OX=9606 GN=APOL1 PE=1 SV=5	APOL1_HUMAN	APOL1	44 kDa			3	2				2		
404	Intercellular adhesion molecule 4 OS=Homo sapiens OX=9606 GN=ICAM4 PE=1 SV=1	ICAM4_HUMAN	ICAM4	29 kDa	4	5	4			3	2		4	
405	Apolipoprotein C-I OS=Homo sapiens OX=9606 GN=APOC1 PE=1 SV=1	APOC1_HUMAN	APOC1	9 kDa	4	5		2						3
406	Glucose-6-phosphate 1-dehydrogenase OS=Homo sapiens OX=9606 GN=G6PD PE=1 SV=4	G6PD_HUMAN	G6PD	59 kDa					2	3				6
407	Complement C1q subcomponent subunit C OS=Homo sapiens OX=9606 GN=C1QC PE=1 SV=3	C1QC_HUMAN	C1QC	26 kDa	2			8			3	3		3
408	T-complex protein 1 subunit eta OS=Homo sapiens OX=9606 GN=CCT7 PE=1 SV=2	TCPH_HUMAN	CCT7	59 kDa					5	2		6		
409	AP-2 complex subunit sigma OS=Homo sapiens OX=9606 GN=AP2S1 PE=1 SV=2	AP2S1_HUMAN	AP2S1	17 kDa								5		4
410	Adenylyl cyclase-associated protein 1 OS=Homo sapiens OX=9606 GN=CAP1 PE=1 SV=5	CAP1_HUMAN	CAP1	52 kDa	2	5				4	4			
411	Myosin-9 OS=Homo sapiens OX=9606 GN=MYH9 PE=1 SV=4	MYH9_HUMAN	MYH9	227 kDa						4		4		
412	WD repeat-containing protein 91 OS=Homo sapiens OX=9606 GN=WDR91 PE=1 SV=2	WDR91_HUMAN	WDR91	83 kDa						6		2		5
413	Guanine nucleotide-binding protein G(I)/G(S)/G(O) subunit gamma-5 OS=Homo sapiens OX=9606 GN=GNG5 PE=1 SV=3	GBG5_HUMAN	GNG5	7 kDa					3	3	2	3	3	4
414	Clathrin light chain A OS=Homo sapiens OX=9606 GN=CLTA PE=1 SV=1	CLCA_HUMAN	CLTA	27 kDa	2	2		2		2				3
415	Retinol-binding protein 4 OS=Homo sapiens OX=9606 GN=RBP4 PE=1 SV=3	RET4_HUMAN	RBP4	23 kDa	7	5		2		2				2
416	Neutral amino acid transporter B(0) OS=Homo sapiens OX=9606 GN=SLC1A5 PE=1 SV=2	AAAT_HUMAN	SLC1A5	57 kDa								14		4
417	Ras-related protein Rab-5B OS=Homo sapiens OX=9606 GN=RAB5B PE=1 SV=1	RAB5B_HUMAN	RAB5B	24 kDa	5			6	6	6		4		4
418	WW domain-binding protein 2 OS=Homo sapiens OX=9606 GN=WBP2 PE=1 SV=1	WBP2_HUMAN	WBP2	28 kDa	3					4	2	2		5
419	S-methyl-5'-thioadenosine phosphorylase OS=Homo sapiens OX=9606 GN=MTAP PE=1 SV=2	MTAP_HUMAN	MTAP	31 kDa							3			4
420	Blood group Rh(D) polypeptide OS=Homo sapiens OX=9606 GN=RHD PE=1 SV=3	RHD_HUMAN	RHD	45 kDa								14	10	
421	Sodium/potassium-transporting ATPase subunit alpha-1 OS=Homo sapiens OX=9606 GN=ATP1A1 PE=1 SV=1	AT1A1_HUMAN	ATP1A1	113 kDa	2				3	5				3
422	Xaa-Pro dipeptidase OS=Homo sapiens OX=9606 GN=PEPD PE=1 SV=3	PEPD_HUMAN	PEPD	55 kDa								5		3
423	WD repeat-containing protein 1 OS=Homo sapiens OX=9606 GN=WDR1 PE=1 SV=4	WDR1_HUMAN	WDR1	66 kDa	2	3	2		2		3			
424	Annexin A4 OS=Homo sapiens OX=9606 GN=ANXA4 PE=1 SV=4	ANXA4_HUMAN	ANXA4	36 kDa						8				10
425	Protein lifeguard 3 OS=Homo sapiens OX=9606 GN=TMBIM1 PE=1 SV=2	LFG3_HUMAN	TMBIM1	35 kDa					3		2	4	3	3
426	Chloride intracellular channel protein 1 OS=Homo sapiens OX=9606 GN=CLIC1 PE=1 SV=4	CLIC1_HUMAN	CLIC1	27 kDa	3	4				4	2		2	
427	L-xylulose reductase OS=Homo sapiens OX=9606 GN=DCXR PE=1 SV=2	DCXR_HUMAN	DCXR	26 kDa					4			3	3	
428	Guanine nucleotide-binding protein G(I)/G(S)/G(O) subunit gamma-2 OS=Homo sapiens OX=9606 GN=GNG2 PE=1 SV=2	GBG2_HUMAN	GNG2	8 kDa										2
429	Protein CutA OS=Homo sapiens OX=9606 GN=CUTA PE=1 SV=2	CUTA_HUMAN	CUTA	19 kDa			2			4		3		
430	Apolipoprotein C-III OS=Homo sapiens OX=9606 GN=APOC3 PE=1 SV=1	APOC3_HUMAN	APOC3	11 kDa			5					5		
431	Alpha-2-antiplasmin OS=Homo sapiens OX=9606 GN=SERPINF2 PE=1 SV=3	A2AP_HUMAN	SERPINF2	55 kDa			2		2			4	3	
432	14-3-3 protein eta OS=Homo sapiens OX=9606 GN=YWHAH PE=1 SV=4	1433F_HUMAN	YWHAH	28 kDa			5				7	5		10
433	Heat shock protein beta-1 OS=Homo sapiens OX=9606 GN=HSPB1 PE=1 SV=2	HSPB1_HUMAN	HSPB1	23 kDa						7				13
434	26S proteasome non-ATPase regulatory subunit 9 OS=Homo sapiens OX=9606 GN=PSMD9 PE=1 SV=3	PSMD9_HUMAN	PSMD9	25 kDa		2		2	2		2			
435	Ras-related protein Rap-2a OS=Homo sapiens OX=9606 GN=RAP2A PE=1 SV=1	RAP2A_HUMAN	RAP2A	21 kDa			15		14	13		28		8
436	Fascin OS=Homo sapiens OX=9606 GN=FSCN1 PE=1 SV=3	FSCN1_HUMAN	FSCN1	55 kDa						3				3
437	SEC14-like protein 4 OS=Homo sapiens OX=9606 GN=SEC14L4 PE=1 SV=1	S14L4_HUMAN	SEC14L4	47 kDa					2					4
438	HLA class I histocompatibility antigen, B-41 alpha chain OS=Homo sapiens OX=9606 GN=HLA-B PE=1 SV=1	1B41_HUMAN	HLA-B	41 kDa	5	2		4			3			
439	Aldehyde dehydrogenase family 16 member A1 OS=Homo sapiens OX=9606 GN=ALDH16A1 PE=1 SV=2	A16A1_HUMAN	ALDH16A1	85 kDa	2		3	2	4					
440	Ras suppressor protein 1 OS=Homo sapiens OX=9606 GN=RSU1 PE=1 SV=3	RSU1_HUMAN	RSU1	32 kDa		3	3					2		
441	40S ribosomal protein SA OS=Homo sapiens OX=9606 GN=RPSA PE=1 SV=4	RSSA_HUMAN	RPSA	33 kDa						6	3	2		8
442	Unconventional myosin-Ic OS=Homo sapiens OX=9606 GN=MYO1C PE=1 SV=4	MYO1C_HUMAN	MYO1C	122 kDa			3		11				3	
443	Complement C1s subcomponent OS=Homo sapiens OX=9606 GN=C1S PE=1 SV=1	C1S_HUMAN	C1S	77 kDa				4			6			
444	Vacuolar protein sorting-associated protein 28 homolog OS=Homo sapiens OX=9606 GN=VPS28 PE=1 SV=1	VPS28_HUMAN	VPS28	25 kDa			5			5		2		3
445	E2 ubiquitin-conjugating enzyme OS=Homo sapiens OX=9606 GN=UBE2O PE=1 SV=3	sp|Q9C0C9|UBE2O_HUMAN	UBE2O	141 kDa					2	4		3		7
446	Pleckstrin-2 OS=Homo sapiens OX=9606 GN=PLEK2 PE=1 SV=1	PLEK2_HUMAN	PLEK2	40 kDa								9		7
447	26S proteasome regulatory subunit 7 OS=Homo sapiens OX=9606 GN=PSMC2 PE=1 SV=3	PRS7_HUMAN	PSMC2	49 kDa		2	2	2	2		3		2	2
448	Rab GDP dissociation inhibitor alpha OS=Homo sapiens OX=9606 GN=GDI1 PE=1 SV=2	GDIA_HUMAN	GDI1	51 kDa					20	15			23	
449	26S proteasome regulatory subunit 10B OS=Homo sapiens OX=9606 GN=PSMC6 PE=1 SV=1	PRS10_HUMAN	PSMC6	44 kDa			3			2		3	4	
450	Peptidyl-prolyl cis-trans isomerase FKBP1A OS=Homo sapiens OX=9606 GN=FKBP1A PE=1 SV=2	FKB1A_HUMAN	FKBP1A	12 kDa			6						5	4
451	Immunoglobulin heavy variable 5-51 OS=Homo sapiens OX=9606 GN=IGHV5-51 PE=3 SV=1	HV551_HUMAN	IGHV5-51	13 kDa	2	2	3	6		3				
452	TIP41-like protein OS=Homo sapiens OX=9606 GN=TIPRL PE=1 SV=2	TIPRL_HUMAN	TIPRL	31 kDa										4
453	Guanine nucleotide-binding protein subunit alpha-11 OS=Homo sapiens OX=9606 GN=GNA11 PE=1 SV=2	GNA11_HUMAN	GNA11	42 kDa					3	7		8		
454	C-1-tetrahydrofolate synthase, cytoplasmic OS=Homo sapiens OX=9606 GN=MTHFD1 PE=1 SV=3	C1TC_HUMAN	MTHFD1	102 kDa						2		10		2
455	Immunoglobulin heavy variable 3-7 OS=Homo sapiens OX=9606 GN=IGHV3-7 PE=1 SV=2	HV307_HUMAN	IGHV3-7	13 kDa	3	7	5	4		3	3			
456	Leukocyte surface antigen CD47 OS=Homo sapiens OX=9606 GN=CD47 PE=1 SV=1	CD47_HUMAN	CD47	35 kDa	2	3		3		2	2	2		2
457	Cytochrome b5 OS=Homo sapiens OX=9606 GN=CYB5A PE=1 SV=2	CYB5_HUMAN	CYB5A	15 kDa								3		
458	Protein AMBP OS=Homo sapiens OX=9606 GN=AMBP PE=1 SV=1	AMBP_HUMAN	AMBP	39 kDa	3	4	2	2				2		2
459	DnaJ homolog subfamily A member 4 OS=Homo sapiens OX=9606 GN=DNAJA4 PE=1 SV=1	DNJA4_HUMAN	DNAJA4	45 kDa			3			3	3			
460	AP-1 complex subunit beta-1 OS=Homo sapiens OX=9606 GN=AP1B1 PE=1 SV=2	AP1B1_HUMAN	AP1B1	105 kDa			4				3	4	4	5
461	Gamma-aminobutyric acid receptor-associated protein-like 2 OS=Homo sapiens OX=9606 GN=GABARAPL2 PE=1 SV=1	GBRL2_HUMAN	GABARAPL2	14 kDa						3				3
462	BolA-like protein 2 OS=Homo sapiens OX=9606 GN=BOLA2 PE=1 SV=1	BOLA2_HUMAN	BOLA2	10 kDa		3					2			3
463	Desmoglein-1 OS=Homo sapiens OX=9606 GN=DSG1 PE=1 SV=2	DSG1_HUMAN	DSG1	114 kDa		3								
464	Transgelin-2 OS=Homo sapiens OX=9606 GN=TAGLN2 PE=1 SV=3	TAGL2_HUMAN	TAGLN2	22 kDa						2				4
465	Protein DPCD OS=Homo sapiens OX=9606 GN=DPCD PE=1 SV=2	DPCD_HUMAN	DPCD	23 kDa						3	4			
466	Poly(rC)-binding protein 1 OS=Homo sapiens OX=9606 GN=PCBP1 PE=1 SV=2	PCBP1_HUMAN	PCBP1	37 kDa						2		5		4
467	Complement C1r subcomponent OS=Homo sapiens OX=9606 GN=C1R PE=1 SV=2	C1R_HUMAN	C1R	80 kDa				3			8			
468	Charged multivesicular body protein 3 OS=Homo sapiens OX=9606 GN=CHMP3 PE=1 SV=3	CHMP3_HUMAN	CHMP3	25 kDa						5				5
469	SH3 domain-binding glutamic acid-rich-like protein 3 OS=Homo sapiens OX=9606 GN=SH3BGRL3 PE=1 SV=1	SH3L3_HUMAN	SH3BGRL3	10 kDa						3				
470	Sorbitol dehydrogenase OS=Homo sapiens OX=9606 GN=SORD PE=1 SV=4	DHSO_HUMAN	SORD	38 kDa						2		2	2	
471	Ubiquitin thioesterase OTUB1 OS=Homo sapiens OX=9606 GN=OTUB1 PE=1 SV=2	OTUB1_HUMAN	OTUB1	31 kDa	2		2	2		2		2		2
472	Ras-related protein Rab-2A OS=Homo sapiens OX=9606 GN=RAB2A PE=1 SV=1	RAB2A_HUMAN	RAB2A	24 kDa					5			7		
473	Galectin-9 OS=Homo sapiens OX=9606 GN=LGALS9 PE=1 SV=2	LEG9_HUMAN	LGALS9	40 kDa		2		3						5
474	Platelet-activating factor acetylhydrolase IB subunit alpha OS=Homo sapiens OX=9606 GN=PAFAH1B1 PE=1 SV=2	LIS1_HUMAN	PAFAH1B1	47 kDa			2					3		3
475	Immunoglobulin heavy variable 4-34 OS=Homo sapiens OX=9606 GN=IGHV4-34 PE=1 SV=2	HV434_HUMAN	IGHV4-34	14 kDa		2				4		2		2
476	DnaJ homolog subfamily C member 5 OS=Homo sapiens OX=9606 GN=DNAJC5 PE=1 SV=1	DNJC5_HUMAN	DNAJC5	22 kDa			4			4				
477	UTP--glucose-1-phosphate uridylyltransferase OS=Homo sapiens OX=9606 GN=UGP2 PE=1 SV=5	UGPA_HUMAN	UGP2	57 kDa			2		2	3		3	2	
478	Leukotriene A-4 hydrolase OS=Homo sapiens OX=9606 GN=LTA4H PE=1 SV=2	LKHA4_HUMAN	LTA4H	69 kDa			3		2				2	
479	Myosin regulatory light chain 12A OS=Homo sapiens OX=9606 GN=MYL12A PE=1 SV=2	ML12A_HUMAN (+1)	MYL12A	20 kDa						7				2
480	Importin-5 OS=Homo sapiens OX=9606 GN=IPO5 PE=1 SV=4	IPO5_HUMAN	IPO5	124 kDa						4		5		
481	Proteasome subunit beta type-6 OS=Homo sapiens OX=9606 GN=PSMB6 PE=1 SV=4	PSB6_HUMAN	PSMB6	25 kDa				2		3				2
482	Dermcidin OS=Homo sapiens OX=9606 GN=DCD PE=1 SV=2	DCD_HUMAN	DCD	11 kDa		2		2				5	2	
483	Vacuolar protein sorting-associated protein 37C OS=Homo sapiens OX=9606 GN=VPS37C PE=1 SV=2	VP37C_HUMAN	VPS37C	39 kDa								2		4
484	Complement factor I OS=Homo sapiens OX=9606 GN=CFI PE=1 SV=2	CFAI_HUMAN	CFI	66 kDa	5	2								
485	Ubiquitin carboxyl-terminal hydrolase isozyme L3 OS=Homo sapiens OX=9606 GN=UCHL3 PE=1 SV=1	UCHL3_HUMAN	UCHL3	26 kDa						3				
486	cAMP-dependent protein kinase catalytic subunit alpha OS=Homo sapiens OX=9606 GN=PRKACA PE=1 SV=2	KAPCA_HUMAN	PRKACA	41 kDa						3		3		2
487	26S proteasome non-ATPase regulatory subunit 10 OS=Homo sapiens OX=9606 GN=PSMD10 PE=1 SV=1	PSD10_HUMAN	PSMD10	24 kDa						2				
488	DNA damage-binding protein 1 OS=Homo sapiens OX=9606 GN=DDB1 PE=1 SV=1	DDB1_HUMAN	DDB1	127 kDa								3		3
489	Cytochrome b reductase 1 OS=Homo sapiens OX=9606 GN=CYBRD1 PE=1 SV=1	CYBR1_HUMAN	CYBRD1	32 kDa								4		
490	Peptidyl-prolyl cis-trans isomerase NIMA-interacting 1 OS=Homo sapiens OX=9606 GN=PIN1 PE=1 SV=1	PIN1_HUMAN	PIN1	18 kDa						2	3			2
491	Ras-related protein Rab-21 OS=Homo sapiens OX=9606 GN=RAB21 PE=1 SV=3	RAB21_HUMAN	RAB21	24 kDa			5			2				4
492	Complement C5 OS=Homo sapiens OX=9606 GN=C5 PE=1 SV=4	CO5_HUMAN	C5	188 kDa	4		2		2			3		
493	Histone-arginine methyltransferase CARM1 OS=Homo sapiens OX=9606 GN=CARM1 PE=1 SV=3	CARM1_HUMAN	CARM1	66 kDa			4			5		3		
494	Deoxyribonuclease-1-like 1 OS=Homo sapiens OX=9606 GN=DNASE1L1 PE=1 SV=1	DNSL1_HUMAN	DNASE1L1	34 kDa			3		2					
495	26S proteasome regulatory subunit 4 OS=Homo sapiens OX=9606 GN=PSMC1 PE=1 SV=1	PRS4_HUMAN	PSMC1	49 kDa			3		2	4			2	2
496	UV excision repair protein RAD23 homolog B OS=Homo sapiens OX=9606 GN=RAD23B PE=1 SV=1	RD23B_HUMAN	RAD23B	43 kDa			4							
497	Serine/threonine-protein kinase mTOR OS=Homo sapiens OX=9606 GN=MTOR PE=1 SV=1	MTOR_HUMAN	MTOR	289 kDa						2				
498	Serum amyloid A-4 protein OS=Homo sapiens OX=9606 GN=SAA4 PE=1 SV=2	SAA4_HUMAN	SAA4	15 kDa			3							
499	5'-AMP-activated protein kinase subunit gamma-1 OS=Homo sapiens OX=9606 GN=PRKAG1 PE=1 SV=1	AAKG1_HUMAN	PRKAG1	38 kDa			3					6		
500	ATPase ASNA1 OS=Homo sapiens OX=9606 GN=ASNA1 PE=1 SV=2	ASNA_HUMAN	ASNA1	39 kDa					2	2			2	2
501	Alpha-adducin OS=Homo sapiens OX=9606 GN=ADD1 PE=1 SV=2	ADDA_HUMAN	ADD1	81 kDa								3		2
502	Proteasome subunit beta type-7 OS=Homo sapiens OX=9606 GN=PSMB7 PE=1 SV=1	PSB7_HUMAN	PSMB7	30 kDa								5		2
503	Mitogen-activated protein kinase kinase kinase kinase 4 OS=Homo sapiens OX=9606 GN=MAP4K4 PE=1 SV=2	M4K4_HUMAN	MAP4K4	142 kDa						2				5

504	CD82 antigen OS=Homo sapiens OX=9606 GN=CD82 PE=1 SV=1	CD82_HUMAN	CD82	30 kDa								5		
505	Immunoglobulin heavy variable 3-72 OS=Homo sapiens OX=9606 GN=IGHV3-72 PE=3 SV=1	HV372_HUMAN	IGHV3-72	13 kDa				3						
506	Multifunctional protein ADE2 OS=Homo sapiens OX=9606 GN=PAICS PE=1 SV=3	PUR6_HUMAN	PAICS	47 kDa					3			2		2
507	Beta-adducin OS=Homo sapiens OX=9606 GN=ADD2 PE=1 SV=3	ADDB_HUMAN	ADD2	81 kDa						2				4
508	V-type proton ATPase subunit B, brain isoform OS=Homo sapiens OX=9606 GN=ATP6V1B2 PE=1 SV=3	VATB2_HUMAN	ATP6V1B2	57 kDa	3		2					2		
509	Immunoglobulin kappa variable 3-20 OS=Homo sapiens OX=9606 GN=IGKV3-20 PE=1 SV=2	KV320_HUMAN	IGKV3-20	13 kDa	3		3	2		2				
510	Syntaxin-binding protein 2 OS=Homo sapiens OX=9606 GN=STXBP2 PE=1 SV=2	STXB2_HUMAN	STXBP2	66 kDa			3		4					
511	Platelet glycoprotein 4 OS=Homo sapiens OX=9606 GN=CD36 PE=1 SV=2	CD36_HUMAN	CD36	53 kDa		2				6				
512	Synaptosomal-associated protein 29 OS=Homo sapiens OX=9606 GN=SNAP29 PE=1 SV=1	SNP29_HUMAN	SNAP29	29 kDa						5	3			3
513	Ragulator complex protein LAMTOR2 OS=Homo sapiens OX=9606 GN=LAMTOR2 PE=1 SV=1	LTOR2_HUMAN	LAMTOR2	14 kDa						2				7
514	Keratin, type II cuticular Hb5 OS=Homo sapiens OX=9606 GN=KRT85 PE=1 SV=1	KRT85_HUMAN	KRT85	56 kDa	28									
515	LIM and SH3 domain protein 1 OS=Homo sapiens OX=9606 GN=LASP1 PE=1 SV=2	LASP1_HUMAN	LASP1	30 kDa		2		2						
516	Transmembrane protein 222 OS=Homo sapiens OX=9606 GN=TMEM222 PE=1 SV=2	TM222_HUMAN	TMEM222	23 kDa					2			2	2	
517	Pyridoxal phosphate phosphatase OS=Homo sapiens OX=9606 GN=PDXP PE=1 SV=2	PLPP_HUMAN	PDXP	32 kDa	2					2				4
518	Platelet factor 4 OS=Homo sapiens OX=9606 GN=PF4 PE=1 SV=2	PLF4_HUMAN	PF4	11 kDa	4			4						
519	Heterogeneous nuclear ribonucleoprotein K OS=Homo sapiens OX=9606 GN=HNRNPK PE=1 SV=1	HNRPK_HUMAN	HNRNPK	51 kDa			2			4	3			3
520	Tumor susceptibility gene 101 protein OS=Homo sapiens OX=9606 GN=TSG101 PE=1 SV=2	TS101_HUMAN	TSG101	44 kDa			3			3		2		
521	Copine-3 OS=Homo sapiens OX=9606 GN=CPNE3 PE=1 SV=1	CPNE3_HUMAN	CPNE3	60 kDa						4				4
522	Microtubule-associated protein RP/EB family member 1 OS=Homo sapiens OX=9606 GN=MAPRE1 PE=1 SV=3	MARE1_HUMAN	MAPRE1	30 kDa										4
523	E3 ubiquitin-protein ligase RNF123 OS=Homo sapiens OX=9606 GN=RNF123 PE=1 SV=1	RN123_HUMAN	RNF123	149 kDa			2		3				4	
524	BTB/POZ domain-containing protein KCTD12 OS=Homo sapiens OX=9606 GN=KCTD12 PE=1 SV=1	KCD12_HUMAN	KCTD12	36 kDa	2			2			5			
525	Adiponectin OS=Homo sapiens OX=9606 GN=ADIPOQ PE=1 SV=1	ADIPO_HUMAN	ADIPOQ	26 kDa				6			7			
526	Rieske domain-containing protein OS=Homo sapiens OX=9606 GN=RFESD PE=1 SV=1	RFESD_HUMAN	RFESD	18 kDa		2								2
527	Dihydropteridine reductase OS=Homo sapiens OX=9606 GN=QDPR PE=1 SV=2	DHPR_HUMAN	QDPR	26 kDa		3							2	
528	Bleomycin hydrolase OS=Homo sapiens OX=9606 GN=BLMH PE=1 SV=1	BLMH_HUMAN	BLMH	53 kDa			2							
529	Gamma-glutamylcyclotransferase OS=Homo sapiens OX=9606 GN=GGCT PE=1 SV=1	GGCT_HUMAN	GGCT	21 kDa			3						2	
530	Gamma-enolase OS=Homo sapiens OX=9606 GN=ENO2 PE=1 SV=3	ENOG_HUMAN	ENO2	47 kDa								5		
531	COP9 signalosome complex subunit 3 OS=Homo sapiens OX=9606 GN=COPS3 PE=1 SV=3	CSN3_HUMAN	COPS3	48 kDa			2		3					
532	Immunoglobulin lambda variable 3-10 OS=Homo sapiens OX=9606 GN=IGLV3-10 PE=3 SV=2	LV310_HUMAN	IGLV3-10	12 kDa							2			3
533	F-actin-capping protein subunit alpha-2 OS=Homo sapiens OX=9606 GN=CAPZA2 PE=1 SV=3	CAZA2_HUMAN	CAPZA2	33 kDa						4		3		5
534	Hornerin OS=Homo sapiens OX=9606 GN=HRNR PE=1 SV=2	HORN_HUMAN	HRNR	282 kDa	2								5	
535	Junction plakoglobin OS=Homo sapiens OX=9606 GN=JUP PE=1 SV=3	PLAK_HUMAN	JUP	82 kDa	7									
536	Elongation factor 1-delta OS=Homo sapiens OX=9606 GN=EEF1D PE=1 SV=5	EF1D_HUMAN	EEF1D	31 kDa						3		3		2
537	Ketosamine-3-kinase OS=Homo sapiens OX=9606 GN=FN3KRP PE=1 SV=2	KT3K_HUMAN	FN3KRP	34 kDa		2								2
538	Phospholipid scramblase 4 OS=Homo sapiens OX=9606 GN=PLSCR4 PE=1 SV=2	PLS4_HUMAN	PLSCR4	37 kDa										2
539	Immunoglobulin kappa variable 3-15 OS=Homo sapiens OX=9606 GN=IGKV3-15 PE=1 SV=2	KV315_HUMAN	IGKV3-15	12 kDa			2							
540	Carboxymethylenebutenolidase homolog OS=Homo sapiens OX=9606 GN=CMBL PE=1 SV=1	CMBL_HUMAN	CMBL	28 kDa			3		2					
541	Apolipoprotein(a) OS=Homo sapiens OX=9606 GN=LPA PE=1 SV=1	APOA_HUMAN	LPA	501 kDa		2	3						2	
542	Myosin light chain 4 OS=Homo sapiens OX=9606 GN=MYL4 PE=1 SV=3	MYL4_HUMAN	MYL4	22 kDa						4				3
543	Leucyl-cystinyl aminopeptidase OS=Homo sapiens OX=9606 GN=LNPEP PE=1 SV=3	LCAP_HUMAN	LNPEP	117 kDa								6		
544	GTPase NRas OS=Homo sapiens OX=9606 GN=NRAS PE=1 SV=1	RASN_HUMAN	NRAS	21 kDa			11					14		
545	26S proteasome non-ATPase regulatory subunit 5 OS=Homo sapiens OX=9606 GN=PSMD5 PE=1 SV=3	PSMD5_HUMAN	PSMD5	56 kDa								2		
546	Protein S100-A8 OS=Homo sapiens OX=9606 GN=S100A8 PE=1 SV=1	S10A8_HUMAN	S100A8	11 kDa										2
547	Keratin, type I cytoskeletal 17 OS=Homo sapiens OX=9606 GN=KRT17 PE=1 SV=2	K1C17_HUMAN	KRT17	48 kDa				6						
548	Fatty acid-binding protein 5 OS=Homo sapiens OX=9606 GN=FABP5 PE=1 SV=3	FABP5_HUMAN	FABP5	15 kDa						2				2
549	Ubiquitin-conjugating enzyme E2 K OS=Homo sapiens OX=9606 GN=UBE2K PE=1 SV=3	UBE2K_HUMAN	UBE2K	22 kDa						2				3
550	Ras-related protein Rab-8A OS=Homo sapiens OX=9606 GN=RAB8A PE=1 SV=1	RAB8A_HUMAN	RAB8A	24 kDa								6		
551	Vitamin K-dependent protein S OS=Homo sapiens OX=9606 GN=PROS1 PE=1 SV=1	PROS_HUMAN	PROS1	75 kDa				4						
552	Disks large homolog 1 OS=Homo sapiens OX=9606 GN=DLG1 PE=1 SV=2	DLG1_HUMAN	DLG1	100 kDa								3		3
553	Polymeric immunoglobulin receptor OS=Homo sapiens OX=9606 GN=PIGR PE=1 SV=4	PIGR_HUMAN	PIGR	83 kDa				3						
554	UPF0687 protein C20orf27 OS=Homo sapiens OX=9606 GN=C20orf27 PE=1 SV=3	CT027_HUMAN	C20orf27	19 kDa	2					3				
555	C4b-binding protein beta chain OS=Homo sapiens OX=9606 GN=C4BPB PE=1 SV=1	C4BPB_HUMAN	C4BPB	28 kDa				3						
556	Adenosine kinase OS=Homo sapiens OX=9606 GN=ADK PE=1 SV=2	ADK_HUMAN	ADK	41 kDa									2	
557	C2 domain-containing protein 5 OS=Homo sapiens OX=9606 GN=C2CD5 PE=1 SV=1	C2CD5_HUMAN	C2CD5	110 kDa								2		
558	Clathrin light chain B OS=Homo sapiens OX=9606 GN=CLTB PE=1 SV=1	CLCB_HUMAN	CLTB	25 kDa										4
559	WD repeat-containing protein 81 OS=Homo sapiens OX=9606 GN=WDR81 PE=1 SV=2	WDR81_HUMAN	WDR81	212 kDa						4				
560	Density-regulated protein OS=Homo sapiens OX=9606 GN=DENR PE=1 SV=2	DENR_HUMAN	DENR	22 kDa										4
561	Glucose-6-phosphate isomerase OS=Homo sapiens OX=9606 GN=GPI PE=1 SV=4	G6PI_HUMAN	GPI	63 kDa						2				
562	Protein kinase C and casein kinase substrate in neurons protein 2 OS=Homo sapiens OX=9606 GN=PACSIN2 PE=1 SV=2	PACN2_HUMAN	PACSIN2	56 kDa						3				2
563	Syntaxin-4 OS=Homo sapiens OX=9606 GN=STX4 PE=1 SV=2	STX4_HUMAN	STX4	34 kDa						3				
564	Prolactin-inducible protein OS=Homo sapiens OX=9606 GN=PIP PE=1 SV=1	PIP_HUMAN	PIP	17 kDa	2	3					2			
565	Translin OS=Homo sapiens OX=9606 GN=TSN PE=1 SV=1	TSN_HUMAN	TSN	26 kDa						3				
566	ADP-ribosylation factor 6 OS=Homo sapiens OX=9606 GN=ARF6 PE=1 SV=2	ARF6_HUMAN	ARF6	20 kDa						2				3
567	N-acetylmuramoyl-L-alanine amidase OS=Homo sapiens OX=9606 GN=PGLYRP2 PE=1 SV=1	PGRP2_HUMAN	PGLYRP2	62 kDa			3							
568	Immunoglobulin heavy variable 3-23 OS=Homo sapiens OX=9606 GN=IGHV3-23 PE=1 SV=2	HV323_HUMAN	IGHV3-23	13 kDa		2		2			2			
569	UMP-CMP kinase OS=Homo sapiens OX=9606 GN=CMPK1 PE=1 SV=3	KCY_HUMAN	CMPK1	22 kDa						2				2
570	Immunoglobulin delta heavy chain OS=Homo sapiens OX=9606 PE=1 SV=1	IGD_HUMAN		56 kDa						2				
571	Vacuolar protein sorting-associated protein 29 OS=Homo sapiens OX=9606 GN=VPS29 PE=1 SV=1	VPS29_HUMAN	VPS29	21 kDa						3				2
572	Prostaglandin E synthase 3 OS=Homo sapiens OX=9606 GN=PTGES3 PE=1 SV=1	TEBP_HUMAN	PTGES3	19 kDa										6
573	Pyrroline-5-carboxylate reductase 3 OS=Homo sapiens OX=9606 GN=PYCR3 PE=1 SV=3	P5CR3_HUMAN	PYCR3	29 kDa		2								2
574	Tryptophan--tRNA ligase, cytoplasmic OS=Homo sapiens OX=9606 GN=WARS PE=1 SV=2	SYWC_HUMAN	WARS	53 kDa						2		2		
575	26S proteasome non-ATPase regulatory subunit 13 OS=Homo sapiens OX=9606 GN=PSMD13 PE=1 SV=2	PSD13_HUMAN	PSMD13	43 kDa					3					
576	Leukocyte elastase inhibitor OS=Homo sapiens OX=9606 GN=SERPINB1 PE=1 SV=1	ILEU_HUMAN	SERPINB1	43 kDa									2	
577	Stathmin OS=Homo sapiens OX=9606 GN=STMN1 PE=1 SV=3	STMN1_HUMAN	STMN1	17 kDa						2		2		
578	Nascent polypeptide-associated complex subunit alpha, muscle-specific form OS=Homo sapiens OX=9606 GN=NACA PE=1 SV=1	NACAM_HUMAN	NACA	205 kDa						4	2			
579	Merlin OS=Homo sapiens OX=9606 GN=NF2 PE=1 SV=1	MERL_HUMAN	NF2	70 kDa			2					2		
580	Ferritin heavy chain OS=Homo sapiens OX=9606 GN=FTH1 PE=1 SV=2	FRIH_HUMAN	FTH1	21 kDa						3		2		
581	Eukaryotic translation initiation factor 2 subunit 1 OS=Homo sapiens OX=9606 GN=EIF2S1 PE=1 SV=3	IF2A_HUMAN	EIF2S1	36 kDa						2				
582	Syntaxin-binding protein 3 OS=Homo sapiens OX=9606 GN=STXBP3 PE=1 SV=2	STXB3_HUMAN	STXBP3	68 kDa								2		
583	Septin-2 OS=Homo sapiens OX=9606 GN=SEPT2 PE=1 SV=1	SEPT2_HUMAN	2-Sep	41 kDa								2		3
584	Thrombospondin-1 OS=Homo sapiens OX=9606 GN=THBS1 PE=1 SV=2	TSP1_HUMAN	THBS1	129 kDa	3									
585	Charged multivesicular body protein 5 OS=Homo sapiens OX=9606 GN=CHMP5 PE=1 SV=1	CHMP5_HUMAN	CHMP5	25 kDa								3		
586	Keratin, type II cytoskeletal 78 OS=Homo sapiens OX=9606 GN=KRT78 PE=1 SV=2	K2C78_HUMAN	KRT78	57 kDa	4									
587	E3 ubiquitin-protein ligase TRIM58 OS=Homo sapiens OX=9606 GN=TRIM58 PE=2 SV=2	TRI58_HUMAN	TRIM58	55 kDa										3
588	Urea transporter 1 OS=Homo sapiens OX=9606 GN=SLC14A1 PE=1 SV=2	UT1_HUMAN	SLC14A1	43 kDa	2									
589	Polypyrimidine tract-binding protein 1 OS=Homo sapiens OX=9606 GN=PTBP1 PE=1 SV=1	PTBP1_HUMAN	PTBP1	57 kDa						3				
590	Poly(rC)-binding protein 2 OS=Homo sapiens OX=9606 GN=PCBP2 PE=1 SV=1	PCBP2_HUMAN	PCBP2	39 kDa										6
591	Rho GDP-dissociation inhibitor 2 OS=Homo sapiens OX=9606 GN=ARHGDIB PE=1 SV=3	GDIR2_HUMAN	ARHGDIB	23 kDa									2	
592	ATP-dependent (S)-NAD(P)H-hydrate dehydratase OS=Homo sapiens OX=9606 GN=NAXD PE=1 SV=1	NNRD_HUMAN	NAXD	37 kDa						2				
593	Synembryn-A OS=Homo sapiens OX=9606 GN=RIC8A PE=1 SV=3	RIC8A_HUMAN	RIC8A	60 kDa			2			2				
594	DnaJ homolog subfamily B member 1 OS=Homo sapiens OX=9606 GN=DNAJB1 PE=1 SV=4	DNJB1_HUMAN	DNAJB1	38 kDa										3
595	60S acidic ribosomal protein P0 OS=Homo sapiens OX=9606 GN=RPLP0 PE=1 SV=1	RLA0_HUMAN	RPLP0	34 kDa								4		
596	E3 ubiquitin-protein ligase NEDD4-like OS=Homo sapiens OX=9606 GN=NEDD4L PE=1 SV=2	NED4L_HUMAN	NEDD4L	112 kDa						4				
597	Receptor-type tyrosine-protein phosphatase alpha OS=Homo sapiens OX=9606 GN=PTPRA PE=1 SV=3	PTPRA_HUMAN	PTPRA	91 kDa								3		
598	Collagen alpha-3(VI) chain OS=Homo sapiens OX=9606 GN=COL6A3 PE=1 SV=5	CO6A3_HUMAN	COL6A3	344 kDa		2								
599	Integrin-linked protein kinase OS=Homo sapiens OX=9606 GN=ILK PE=1 SV=2	ILK_HUMAN	ILK	51 kDa		2								
600	Lipopolysaccharide-binding protein OS=Homo sapiens OX=9606 GN=LBP PE=1 SV=3	LBP_HUMAN	LBP	53 kDa										3
601	Keratin, type I cuticular Ha3-II OS=Homo sapiens OX=9606 GN=KRT33B PE=1 SV=3	KT33B_HUMAN	KRT33B	46 kDa	20									
602	Acidic fibroblast growth factor intracellular-binding protein OS=Homo sapiens OX=9606 GN=FIBP PE=1 SV=3	FIBP_HUMAN	FIBP	42 kDa					2					
603	Zinc transporter 1 OS=Homo sapiens OX=9606 GN=SLC30A1 PE=1 SV=3	ZNT1_HUMAN	SLC30A1	55 kDa	2									
604	Eukaryotic translation initiation factor 3 subunit J OS=Homo sapiens OX=9606 GN=EIF3J PE=1 SV=2	EIF3J_HUMAN	EIF3J	29 kDa										2

605	WD repeat-containing protein 48 OS=Homo sapiens OX=9606 GN=WDR48 PE=1 SV=1	WDR48_HUMAN	WDR48	76 kDa					2					
606	Eukaryotic peptide chain release factor GTP-binding subunit ERF3A OS=Homo sapiens OX=9606 GN=GSPT1 PE=1 SV=1	ERF3A_HUMAN	GSPT1	56 kDa						2				2
607	Mitogen-activated protein kinase 1 OS=Homo sapiens OX=9606 GN=MAPK1 PE=1 SV=3	MK01_HUMAN	MAPK1	41 kDa										3
608	Serine/threonine-protein kinase 10 OS=Homo sapiens OX=9606 GN=STK10 PE=1 SV=1	STK10_HUMAN	STK10	112 kDa					3					
609	Charged multivesicular body protein 1a OS=Homo sapiens OX=9606 GN=CHMP1A PE=1 SV=1	CHM1A_HUMAN	CHMP1A	22 kDa										2
610	Thioredoxin reductase 1, cytoplasmic OS=Homo sapiens OX=9606 GN=TXNRD1 PE=1 SV=3	TRXR1_HUMAN	TXNRD1	71 kDa								3		
611	Vacuolar protein sorting-associated protein 4B OS=Homo sapiens OX=9606 GN=VPS4B PE=1 SV=2	VPS4B_HUMAN	VPS4B	49 kDa										4
612	MAGUK p55 subfamily member 7 OS=Homo sapiens OX=9606 GN=MPP7 PE=1 SV=1	MPP7_HUMAN	MPP7	66 kDa										2
613	60S acidic ribosomal protein P2 OS=Homo sapiens OX=9606 GN=RPLP2 PE=1 SV=1	RLA2_HUMAN	RPLP2	12 kDa						2				
614	RING finger protein 11 OS=Homo sapiens OX=9606 GN=RNF11 PE=1 SV=1	RNF11_HUMAN	RNF11	17 kDa										3
615	Activator of 90 kDa heat shock protein ATPase homolog 1 OS=Homo sapiens OX=9606 GN=AHSA1 PE=1 SV=1	AHSA1_HUMAN	AHSA1	38 kDa										3
616	Ragulator complex protein LAMTOR1 OS=Homo sapiens OX=9606 GN=LAMTOR1 PE=1 SV=2	LTOR1_HUMAN	LAMTOR1	18 kDa										2
617	Caspase-14 OS=Homo sapiens OX=9606 GN=CASP14 PE=1 SV=2	CASPE_HUMAN	CASP14	28 kDa	2									
618	Heterogeneous nuclear ribonucleoprotein H OS=Homo sapiens OX=9606 GN=HNRNPH1 PE=1 SV=4	HNRH1_HUMAN	HNRNPH1	49 kDa										5
619	Niban-like protein 1 OS=Homo sapiens OX=9606 GN=FAM129B PE=1 SV=3	NIBL1_HUMAN	FAM129B	84 kDa					4					
620	Mannan-binding lectin serine protease 2 OS=Homo sapiens OX=9606 GN=MASP2 PE=1 SV=4	MASP2_HUMAN	MASP2	76 kDa						2				
621	Eukaryotic translation initiation factor 2 subunit 3 OS=Homo sapiens OX=9606 GN=EIF2S3 PE=1 SV=3	IF2G_HUMAN	EIF2S3	51 kDa										2
622	Serine/threonine-protein phosphatase CPPED1 OS=Homo sapiens OX=9606 GN=CPPED1 PE=1 SV=3	CPPED_HUMAN	CPPED1	36 kDa										2
623	Protein EFR3 homolog A OS=Homo sapiens OX=9606 GN=EFR3A PE=1 SV=2	EFR3A_HUMAN	EFR3A	93 kDa								2		
624	60 kDa heat shock protein, mitochondrial OS=Homo sapiens OX=9606 GN=HSPD1 PE=1 SV=2	CH60_HUMAN	HSPD1	61 kDa										3
625	Dynactin subunit 2 OS=Homo sapiens OX=9606 GN=DCTN2 PE=1 SV=4	DCTN2_HUMAN	DCTN2	44 kDa				3						2
626	Plexin-A1 OS=Homo sapiens OX=9606 GN=PLXNA1 PE=1 SV=3	PLXA1_HUMAN	PLXNA1	211 kDa			3							
627	Coagulation factor XIII A chain OS=Homo sapiens OX=9606 GN=F13A1 PE=1 SV=4	F13A_HUMAN	F13A1	83 kDa		2								
628	Mannan-binding lectin serine protease 1 OS=Homo sapiens OX=9606 GN=MASP1 PE=1 SV=3	MASP1_HUMAN	MASP1	79 kDa			2							
629	Coronin-1A OS=Homo sapiens OX=9606 GN=CORO1A PE=1 SV=4	COR1A_HUMAN	CORO1A	51 kDa							2			
630	Protein S100-A7 OS=Homo sapiens OX=9606 GN=S100A7 PE=1 SV=4	S10A7_HUMAN	S100A7	11 kDa										2
631	Dematin OS=Homo sapiens OX=9606 GN=DMTN PE=1 SV=3	DEMA_HUMAN	DMTN	46 kDa						2				
632	Endoplasmic reticulum chaperone BiP OS=Homo sapiens OX=9606 GN=HSPA5 PE=1 SV=2	BIP_HUMAN	HSPA5	72 kDa										6
633	Filaggrin-2 OS=Homo sapiens OX=9606 GN=FLG2 PE=1 SV=1	FILA2_HUMAN	FLG2	248 kDa	4									
634	UBAP1-MVB12-associated (UMA)-domain containing protein 1 OS=Homo sapiens OX=9606 GN=UMAD1 PE=2 SV=2	UMAD1_HUMAN	UMAD1	15 kDa										3
635	5'-AMP-activated protein kinase catalytic subunit alpha-1 OS=Homo sapiens OX=9606 GN=PRKAA1 PE=1 SV=4	AAPK1_HUMAN	PRKAA1	64 kDa								3		
636	Thioredoxin-dependent peroxide reductase, mitochondrial OS=Homo sapiens OX=9606 GN=PRDX3 PE=1 SV=3	PRDX3_HUMAN	PRDX3	28 kDa										4
637	Peroxiredoxin-5, mitochondrial OS=Homo sapiens OX=9606 GN=PRDX5 PE=1 SV=4	PRDX5_HUMAN	PRDX5	22 kDa						4				
638	Phosphatidylinositol 4-phosphate 5-kinase type-1 alpha OS=Homo sapiens OX=9606 GN=PIP5K1A PE=1 SV=1	PI51A_HUMAN	PIP5K1A	63 kDa								2		
639	Fibulin-1 OS=Homo sapiens OX=9606 GN=FBLN1 PE=1 SV=4	FBLN1_HUMAN	FBLN1	77 kDa	5									
640	Voltage-dependent anion-selective channel protein 3 OS=Homo sapiens OX=9606 GN=VDAC3 PE=1 SV=1	VDAC3_HUMAN	VDAC3	31 kDa										5
641	Pleckstrin OS=Homo sapiens OX=9606 GN=PLEK PE=1 SV=3	PLEK_HUMAN	PLEK	40 kDa		2								
642	Tumor protein D54 OS=Homo sapiens OX=9606 GN=TPD52L2 PE=1 SV=2	TPD54_HUMAN	TPD52L2	22 kDa										2
643	MAP/microtubule affinity-regulating kinase 3 OS=Homo sapiens OX=9606 GN=MARK3 PE=1 SV=5	MARK3_HUMAN	MARK3	84 kDa						2				
644	Endophilin-B2 OS=Homo sapiens OX=9606 GN=SH3GLB2 PE=1 SV=1	SHLB2_HUMAN	SH3GLB2	44 kDa						2				
645	Epidermal growth factor receptor substrate 15-like 1 OS=Homo sapiens OX=9606 GN=EPS15L1 PE=1 SV=1	EP15R_HUMAN	EPS15L1	94 kDa										3
646	Switch-associated protein 70 OS=Homo sapiens OX=9606 GN=SWAP70 PE=1 SV=1	SWP70_HUMAN	SWAP70	69 kDa								2		
647	Protein TMED8 OS=Homo sapiens OX=9606 GN=TMED8 PE=1 SV=1	TMED8_HUMAN	TMED8	36 kDa										2
648	Coagulation factor XII OS=Homo sapiens OX=9606 GN=F12 PE=1 SV=3	FA12_HUMAN	F12	68 kDa			2							
649	Apolipoprotein F OS=Homo sapiens OX=9606 GN=APOF PE=1 SV=2	APOF_HUMAN	APOF	35 kDa								2		
650	Cullin-2 OS=Homo sapiens OX=9606 GN=CUL2 PE=1 SV=2	CUL2_HUMAN	CUL2	87 kDa		2								
651	Metalloreductase STEAP3 OS=Homo sapiens OX=9606 GN=STEAP3 PE=1 SV=2	STEA3_HUMAN	STEAP3	55 kDa								2		
652	Importin subunit alpha-3 OS=Homo sapiens OX=9606 GN=KPNA4 PE=1 SV=1	IMA3_HUMAN	KPNA4	58 kDa								2		
653	G protein-coupled receptor kinase 6 OS=Homo sapiens OX=9606 GN=GRK6 PE=1 SV=2	GRK6_HUMAN	GRK6	66 kDa					2					
654	Heterogeneous nuclear ribonucleoprotein A1-like 2 OS=Homo sapiens OX=9606 GN=HNRNPA1L2 PE=2 SV=2	RA1L2_HUMAN (+1)	HNRNPA1L2	34 kDa						2				
655	NIF3-like protein 1 OS=Homo sapiens OX=9606 GN=NIF3L1 PE=1 SV=2	NIF3L_HUMAN	NIF3L1	42 kDa	2									
656	Palmitoyltransferase ZDHHC5 OS=Homo sapiens OX=9606 GN=ZDHHC5 PE=1 SV=2	ZDHC5_HUMAN	ZDHHC5	78 kDa			2							
657	Platelet glycoprotein Ib alpha chain OS=Homo sapiens OX=9606 GN=GP1BA PE=1 SV=2	GP1BA_HUMAN	GP1BA	72 kDa		2								
